# Exploring MicroRNA-Like Small RNAs in the Filamentous Fungus *Fusarium oxysporum*

**DOI:** 10.1371/journal.pone.0104956

**Published:** 2014-08-20

**Authors:** Rui Chen, Nan Jiang, Qiyan Jiang, Xianjun Sun, Yong Wang, Hui Zhang, Zheng Hu

**Affiliations:** 1 Tianjin Institute of Agricultural Quality Standard and Testing Technology, Tianjin Academy of Agricultural Sciences, Tianjin, China; 2 The National Key Facilities for Crop Genetic Resources and Improvement, Institute of crop sciences, Chinese Academy of Agricultural Sciences, Beijing, China; 3 Institute of Applied Ecology, Chinese Academy of Sciences, Shenyang, China; Woosuk University, Republic of Korea

## Abstract

RNA silencing such as quelling and meiotic silencing by unpaired DNA (MSUD) and several other classes of special small RNAs have been discovered in filamentous fungi recently. More than four different mechanisms of microRNA-like RNAs (milRNAs) production have been illustrated in the model fungus *Neurospora crassa* including a dicer-independent pathway. To date, very little work focusing on small RNAs in fungi has been reported and no universal or particular characteristic of milRNAs were defined clearly. In this study, small RNA and degradome libraries were constructed and subsequently deep sequenced for investigating milRNAs and their potential cleavage targets on the genome level in the filamentous fungus *F. oxysporum* f. sp. *lycopersici*. As a result, there is no intersection of conserved miRNAs found by BLASTing against the miRBase. Further analysis showed that the small RNA population of *F. oxysporum* shared many common features with the small RNAs from *N. crassa* and other fungi. According to the known standards of miRNA prediction in plants and animals, milRNA candidates from 8 families (comprising 19 members) were screened out and identified. However, none of them could trigger target cleavage based on the degradome data. Moreover, most major signals of cleavage in transcripts could not match appropriate complementary small RNAs, suggesting that other predominant modes for milRNA-mediated gene regulation could exist in *F. oxysporum*. In addition, the PAREsnip program was utilized for comprehensive analysis and 3 families of small RNAs leading to transcript cleavage were experimentally validated. Altogether, our findings provided valuable information and important hints for better understanding the functions of the small RNAs and milRNAs in the fungal kingdom.

## Introduction

Small RNAs and their regulatory roles in plants and animals have aroused great interest in molecular biology during the last decade. Various small non-coding RNAs (ncRNAs), about 20∼30 nucleotides (nt) long, deriving from endogenous or extrinsic pathways could regulate genes and genomes at different levels, which is known as post-transcriptional gene silencing (PTGS) [1], quelling [2] or RNA interference (RNAi) [3]. Current research has demonstrated that similar regulatory mechanisms exist in most eukaryotes, even in some eubacteria, archea [4] and unicellular organisms [5]–[7], which play fundamental roles in growth, development and stress responses.

MicroRNAs (miRNAs) [8]–[10], short interfering RNAs (siRNAs) [11] and piwi-interacting RNAs (piRNAs) [12] represent three major categories of functional small RNAs. In recent years, many other types of small RNAs with certain features have been constantly described and classified according to their origins, binding proteins or secondary structures, such as trans-acting siRNAs (tasiRNAs) [13], repeat associated siRNAs (rasiRNAs) [14], natural antisense-siRNAs (natsiRNAs) [15], tiny non-coding RNAs (tncRNAs) [16], heterochromatic small RNAs (hcRNAs) [17] and small scan RNAs (scnRNAs) [18], *etc.*, which sufficiently exhibited a more complex and diverse roles of small RNAs.

Quelling, the first fungal RNA silencing phenomenon, was found in *Neurospora crassa* through the fact that the expression of an endogenous gene was attenuated by transformation with homologous sequences [2], [19], [20]. The following reports on meiotic silencing by unpaired DNA (MSUD) and repeat-induced point mutation (RIP) [21], [22], fungal-specific RNA silencing mechanisms, further indicated that fungi possessed sophisticated RNA regulatory networks. However, the key components of RNA silencing, like RNA-dependent RNA polymerase (RdRP), AGO and Dicer proteins, are not universally present in fungi and lost in a considerable amount of fungal species including the model budding yeast *Saccharomyces cerevisiae* [23]. In some cases, the absent hall-markers could be found in its close relative [24], leading to the hypothesis that the inherent mechanisms of fungal small RNAs are undergoing different stages and levels due to their diverse directions and rate of evolution.

In perspective of miRNAs, at least four kinds of miRNA-like RNAs (milRNAs) have been identified in the model fungus *N. crassa*, including a dicer-independent pathway. The production of these milRNAs didn't require QDE-1 (QUELLING DEFICIENT-1, an RdRP) and QDE-3 (a RecQ DNA helicase) but depended on Dicer, QDE-2 (an AGO protein), the exonuclease QIP and an RNase III domain-containing protein (MRPL3) [25]. Characteristics of these known milRNAs cannot meet the criteria for miRNA prediction in plants or animals exactly. So, to some extent, study of fungal milRNAs would offer a new angle to better understand miRNAs and their regulatory roles in eukaryotic organisms. In recent years, the deep sequencing technology is being applied for fungal milRNAs investigation. Although many research works about small RNAs and/or their roles in PTGS have been reported in basidiomycetous fungus *Cryptococcus neoformans* [26], [27] and filamentous fungi *Metarhizium anisopliae* [28], *Trichoderma reesei* [29], *Penicillium marneffei* [30], and *Sclerotinia sclerotiorum* [31], the milRNA pathway is still poorly understood in most fungal species.

*Fusarium oxysporum*, a widespread soil-borne fungus, is the causal agent of root rots or wilt diseases in many commercial crops and ornamental plants [32]. It consists of non-pathogenic and pathogenic isolates, while the latter is grouped into forma specialis (f. sp.) based on its specificity to a certain range of host species [33]. Because of its strong adaptability and multiple infection strategies, the hosts of *F. oxysporum* are extremely diverse, including human that causes infections [34], [35]. Taxonomically, *F. oxysporum* belongs to the subdivision Deuteromycotina while sexual stages of *Fusarium* species are classified into the subdivision Ascomycotina. It lacks the morphology of sexual reproduction and produces three kinds of asexual spores, macroconidia, microconidia, and chlamydospores [36]. As a model organism for biological and pathogenic research, *F. oxysporum* has been extensively studied in respects of gene transfer system, pathogenicity factor, infection process and signal transduction.

Genome sequencing of *Fusarium* species and comparative genomic analysis provided a wealth of information and greatly enhanced the studies on genetic basis and molecular mechanisms of fungal pathogenicity [37]. *F. oxysporum* f. sp. *lycopersici* genome, about 60 Mb in size, encompasses more than 17,000 genes and contains a ∼19 Mb lineage-specific region. The availability of genome data provided convenience for genome-scale investigation of milRNAs where context sequences around small RNA loci were needed for secondary structure prediction. Additionally, five AGOs, two Dicers and four RdRPs encoded by genome gave tangible evidence to believe that a sophisticated system for small RNA production exists in *F. oxysporum* [23].

Degradome sequencing, also known as parallel analysis of RNA ends (PARE) or genome-wide mapping of uncapped and cleaved transcripts (GMUCT), is a global strategy to experimentally identify degraded transcripts with 5′ monophosphate based on the high-throughput sequencing technology [38]. This approach has been proved to be an effective way to fully analyze miRNA-mediated cleavages in *Arabidopsis*. It is well-known that miRNAs regulate target genes through guiding cleavage or suppressing translation in plants and animals. However, the roles and regulatory patterns of fungal milRNAs are still very ambiguous although the groundbreaking works in *N. crassa* and increasing researches have been reported recently. Herein, we combined degradome and small RNA deep sequencing strategies to entirely investigate milRNAs and truncated targets in *F. oxysporum*, which would yield valuable clues for insightful understanding of small RNAs in fungi.

## Results

### Small RNA library analysis

**Figure 1:**
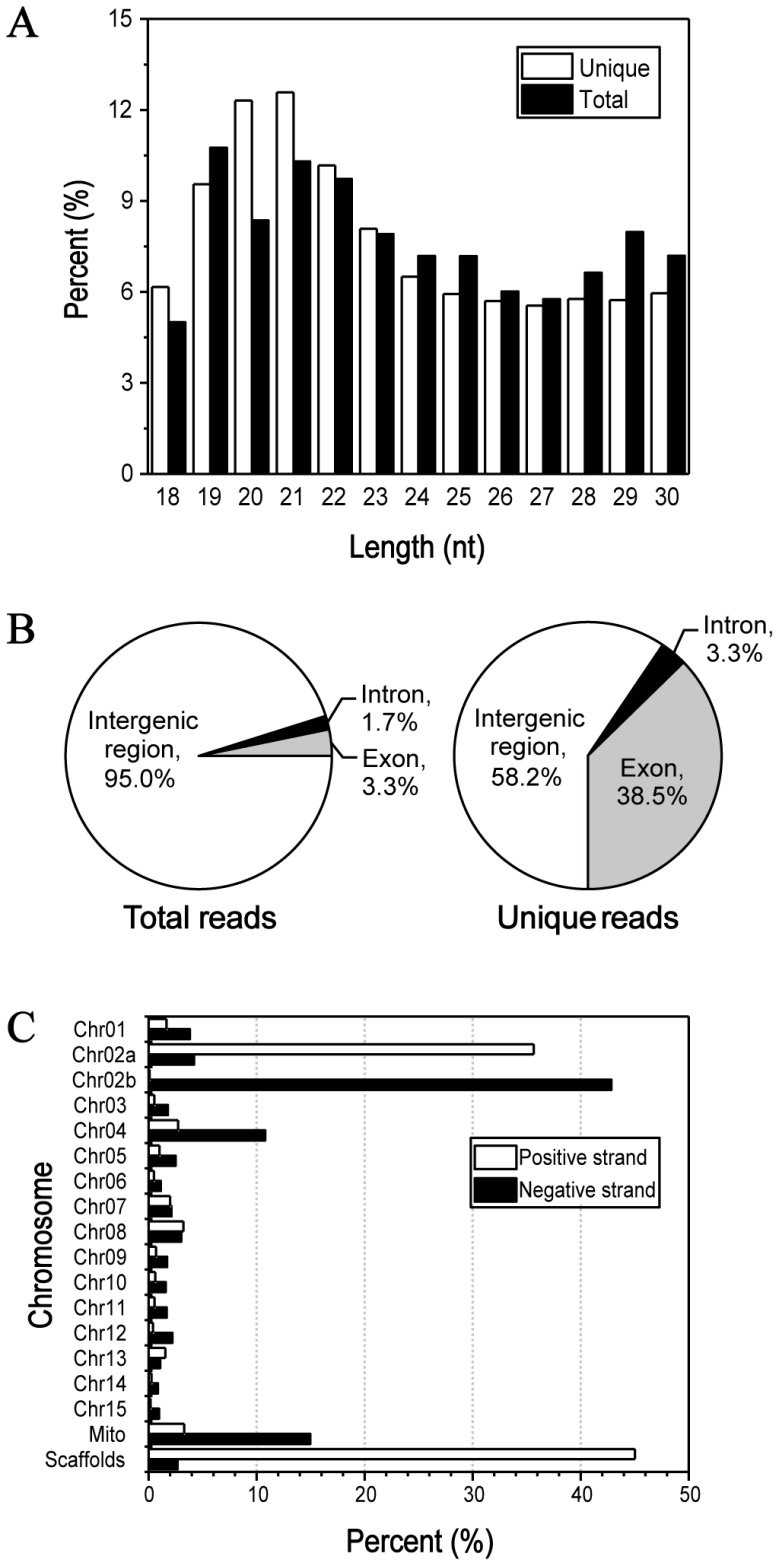
Characterization of small RNAs in *F. oxysporum*. A: Size distribution of small RNAs. White and black columns represent unique and total reads of the small RNAs, respectively. B: Annotation of small RNA loci. Pie graphs show the proportions of small RNAs located in intergenic, exonic and intronic regions, respectively. C: Small RNA distribution on both strands of chromosomes.

To comprehensively survey small RNAs in *F. oxysporum*, total low molecular weight RNA (18∼30 nt) was isolated from seven-day-old mycelia and a small RNA library was constructed. Benefiting from the deep sequencing technology, a total of 45,723,756 raw reads were generated. After removal of low quality reads and 3′ adaptor sequence, 9,985,176 (100%) clean reads, corresponding to 896,322 (100%) unique reads, were obtained for subsequent analysis. Length distribution of clean reads showed that 19 nt∼22 nt small RNAs occupied the major proportion with weak superiority. The 19 nt and 21 nt classes were the most abundant groups in total and unique reads, respectively (**Fig. 1A**).

For determining the origins of small RNAs, clean reads were aligned against *F. oxysporum* genome assembly (117 supercontigs) and only perfectly matched reads were maintained. As a result, 8,435,010 (84.48%) mapped reads, representing 493,304 (55.04%) unique, could be found at least one locus on genome. Annotation of these loci indicated that intergenic region was the major source for small RNA production, accounting for 95.0%, while only 3.3% and 1.7% were associated with exons and introns, respectively (**Fig. 1B**). However, a considerable fraction of unique reads with low abundance were matched on exons (38.5%), which could be degraded products of message RNAs or specific exon-derived small RNAs. Chromosomal distribution analysis showed that small RNAs were intensively generated form Chr02a, Chr02b, Chr04, scaffolds and mitochondrion with preference on sense or antisense strand (**Fig. 1C**).

Before miRNA prediction, mapped reads were screened against ncRNAs, inculding rRNA, tRNA and snoRNA. Notably, 5,867,889 (58.77%) mapped reads were perfectly matched to ribosomal RNA, whereas tiny components come from tRNA and snoRNA (**Table. 1**). The remaining 2,563,874 (25.68%) reads, designated as unknown reads, were used for miRNA prediction pipelines. Chromosomal distribution of unknown reads indicated that, Chr04, Chr08 and mitochondrion were major origins while Chr04, Chr07, Chr08 and Chr13 showed serious strand preference for small RNA generation (**Fig. S2A**). In the rRNA-derived small RNAs, 19 nt, 24 nt and 25 nt were abundant in total and unique reads showed relatively even distribution throughout 18 nt∼30 nt (**Fig. S2B**). No obvious nucleotide bias was observed at the first nucleotide at the 5′ or 3′ end. Also, clean reads, mapped reads and unknown reads shared similar nucleotide bias to that of rRNA-derived small RNAs (**Fig. S2C, D, E, F**).

**Table 1:** Composition of the small RNA library.

	Unique Reads		Total Reads	
	Number	(%)	Number	(%)
Raw reads	—	—	45,723,756	—
High quality reads	—	—	30,279,668	—
Clean reads (18–30 nt)	896,322	(100.00)	9,985,176	(100.00)
Mapped reads^a^	493,304	(55.04)	8,435,010	(84.48)
rRNA	61,967	(6.91)	5,867,889	(58.77)
tRNA	173	(0.02)	862	(0.01)
snoRNA	622	(0.07)	2,385	(0.02)
Unknown reads	430,542	(48.03)	2,563,874	(25.68)

a The 117 supercontigs of *F. oxysporum* genome assembly was generated from BROAD institute. Perfectly matching reads with more than one locus were regarded as mapped reads.

### Conserved miRNAs screening

To identify miRNA homologs, unknown reads were searched against miRBase (Release 19.0) using BLAST and Perl scripts. Two mismatches or length difference were allowed for homolog determination. Although 21,264 hairpins and 25,141 mature miRNAs derived from 193 species were registered in miRBase, only three homologs with low abundance were finally confirmed, which were failed to be defined as milRNA candidates in *F. oxysporum* (fox-milRNAs) during subsequent analysis (**Table S1**). Because of the evolutionary divergence of miRNA genes, there is no common miRNA between plants and animals despite similar mechanisms for miRNA generation co-existed [39]. So, it makes sense that no miRNA orthologous was found between filamentous fungi and other eukaryotic species.

### Prediction and identification of milRNAs

**Figure 2:**
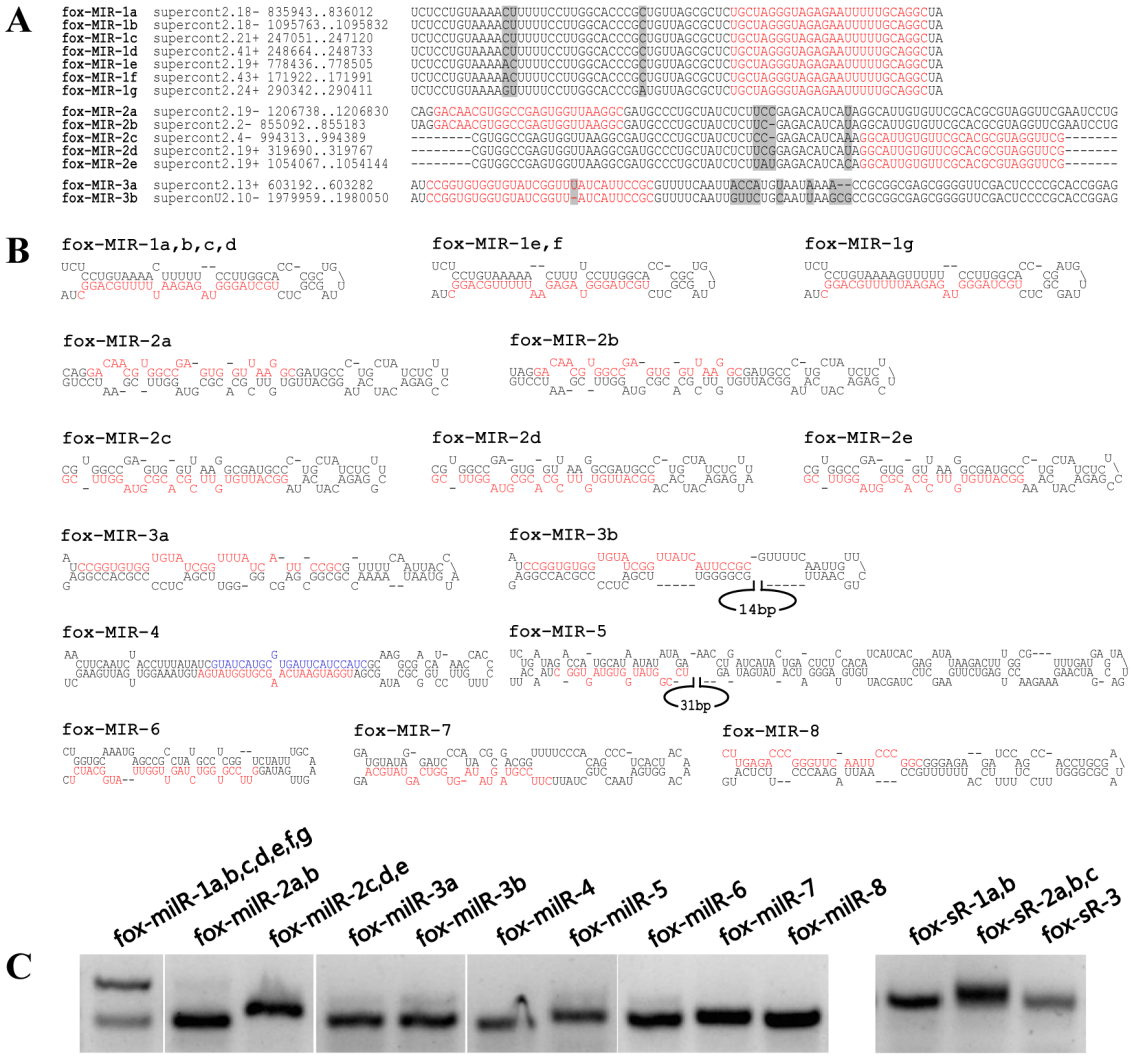
milRNAs identified in this study. A: Multiple sequence alignment of fox-milRNA precursors. Red sequences represent mature milRNAs and gray parts indicate low similarity. B: Secondary structures of fox-milRNA precursors. The red and blue sequences represent mature and star (milRNA*) sequences, respectively. C: Expression analysis of fox-milRNAs and small RNAs by RT-PCR experiments. Total RNA was extracted for seven-day-old mycelia and directly ligated with universal oligos at the 3′ end. After reverse transcription, specific forward primer and common reverse primer could detect a given small RNA. Small RNAs with high abundance are more apt to get positive results. Two bands of fox-milR-1 suggested that the precursor and mature sequences were both amplified and detected.

Due to the fact there is no commonly accepted standards for fungal milRNA prediction, both criteria of plants and animals were employed to comprehensively investigate milRNAs in *F. oxysporum*. miRDeep2 program was carried out with no reference option to predict milRNAs according to animal standards. As a result, 15 milRNA candidates were acquired. 13 of them passed significant randfold p-value test, and 6 of them were not classified as known ncRNAs (**Table S2**). Based on the features of plant miRNA precursors, a stringent pipeline integrating miRCheck was developed and performed for milRNA analysis. After contexts extraction, miRCheck judgment, single-strand evaluation and ortholog clustering, 35 families comprising 66 members were designated as fox-milRNAs, in which 6 families contained multiple members and 9 families were characterized by star strands (miRNA*) (**Table S3**). Subsequently, outputs of miRDeep2 and miRCheck were combined and manually confirmed according to their hairpin structures while fox-milRNA-4 was the only milRNA candidate co-existed in the two groups of results. At last, 15 milRNA candidates were subjected to experimental verification by RT-PCR, in which 10 of them showed positive results (**Fig. 2C** **&** **Table 2**). 11 putative targets of 7 fox-milRNAs were also predicted using an online miRNA target search program, psRNATarget [40] (**Table S4**).

**Table 2:** milRNAs of *F. oxysporum* identified in this study.

Name	ID in this study	Sequence(5′-3′)	Abundance	Length (nt)	Precursors or 5′-end position^a^
fox_milRNA_1a	fox_26 nt_0002525_0000015	UGCUAGGGUAGAGAAUUUUUGCAGGC	2525	26	supercont2.18_−_835943_836012
fox_milRNA_1b	fox_26 nt_0002525_0000015	UGCUAGGGUAGAGAAUUUUUGCAGGC	2525	31	supercont2.18_−_1095763_1095832
fox_milRNA_1c	fox_26 nt_0002525_0000015	UGCUAGGGUAGAGAAUUUUUGCAGGC	2525	26	supercont2.21_+_247051_247120
fox_milRNA_1d	fox_26 nt_0002525_0000015	UGCUAGGGUAGAGAAUUUUUGCAGGC	2525	26	supercont2.41_+_248664_248733
fox_milRNA_1e	fox_26 nt_0002525_0000015	UGCUAGGGUAGAGAAUUUUUGCAGGC	2525	31	supercont2.19_+_778436_778505
fox_milRNA_1f	fox_26 nt_0002525_0000015	UGCUAGGGUAGAGAAUUUUUGCAGGC	2525	26	supercont2.43_+_171922_171991
fox_milRNA_1g	fox_26 nt_0002525_0000015	UGCUAGGGUAGAGAAUUUUUGCAGGC	2525	31	supercont2.24_+_290342_290411
fox_milRNA_2a	fox_25 nt_0000112_0000705	GACAACGUGGCCGAGUGGUUAAGGC	705	25	supercont2.19_−_1206738_1206830
fox_milRNA_2b	fox_25 nt_0000112_0000705	GACAACGUGGCCGAGUGGUUAAGGC	705	25	supercont2.2_−_855092_855183
fox_milRNA_2c	fox_27 nt_0000756_0000077	GGCAUUGUGUUCGCACGCGUAGGUUCG	77	27	supercont2.19_+_319690_319767
fox_milRNA_2d	fox_27 nt_0000756_0000077	GGCAUUGUGUUCGCACGCGUAGGUUCG	77	27	supercont2.19_+_1054067_1054144
fox_milRNA_2e	fox_27 nt_0000756_0000077	GGCAUUGUGUUCGCACGCGUAGGUUCG	77	27	supercont2.4_−_994313_994389
fox_milRNA_3a	fox_30 nt_0000004_0010765	CCGGUGUGGUGUAUCGGUUUAUCAUUCCGC	10765	30	supercont2.13_+_603193_603281
fox_milRNA_3b	fox_29 nt_0000010_0008325	CCGGUGUGGUGUAUCGGUUAUCAUUCCGC	8325	29	supercont2.10_−_1979960_1980049
fox_milRNA_4	fox_23 nt_0000002_0044137	UGGAUGAAUCAAGCGUGGUAUGA	44137	23	supercont2.39_−_99911_100039
fox_milRNA_5	fox_19 nt_0000004_0016753	UCCGGUAUGGUGUAGUGGC	16753	19	supercont2.3_−_3100467_3100674
fox_milRNA_6	fox_29 nt_0000026_0003117	GUUCCGUGGUCUAGUUGGUUAUGGCAUCU	3117	29	supercont2.5_+_2446463_2446540
fox_milRNA_7	fox_27 nt_0000045_0002322	CUUCCGUAGUAUAGUGGUCAGUAUGCA	2322	27	supercont2.14_−_769284_769380
fox_milRNA_8	fox_26 nt_0000088_0000823	CUUGAGACCCGGGUUCAAUUCCCGGC	823	26	supercont2.19_+_73418_73515
fox_sRNA_1a	fox_20 nt_0008904_0000006	CAUCACCACCCUAUGACGGA	6	20	supercont2.2_−_1967219
fox_sRNA_1b	fox_21 nt_0006152_0000010	CAUCACCACCCUAUGACGGAC	10	21	supercont2.2_−_1967218
fox_sRNA_2a	fox_19 nt_0002348_0000022	CAGGACUGAAUGCUUUAUC	22	19	supercont2.115_−_5723
fox_sRNA_2b	fox_20 nt_0009384_0000005	CAGGACUGAAUGCUUUAUCG	5	20	supercont2.115_−_5722
fox_sRNA_2c	fox_21 nt_0001540_0000049	CAGGACUGAAUGCUUUAUCGU	49	21	supercont2.115_−_5721
fox_sRNA_3	fox_19 nt_0004368_0000010	CGCAGAAGGUCCCGAGUUC	10	19	supercont2.2_−_2313383

a The precursor sequences of milRNAs and 5′-end positions of small RNAs loci were indicated, respectively.

The sequence alignment (**Fig. 2A**), secondary structures (**Fig. 2B**) and coverage histograms of fox-milRNA precursors (**Fig. S2G**) were figured out with mature milRNAs indicated. A considerable conservation of the fox-milRNA precursors' sequences was detected when blasted against another 13 genomes of *Fusarium* species, including 11 *F. oxysporum* strains, one *F. graminearum*, and one *F. verticillioides* (**Table S5**). Further alignments of fox-milRNA-4 precursors confirmed the conservation of the hairpin loop structure in *F. oxysporum* (**Fig. S3**).

### Degradome library analysis

**Figure 3:**
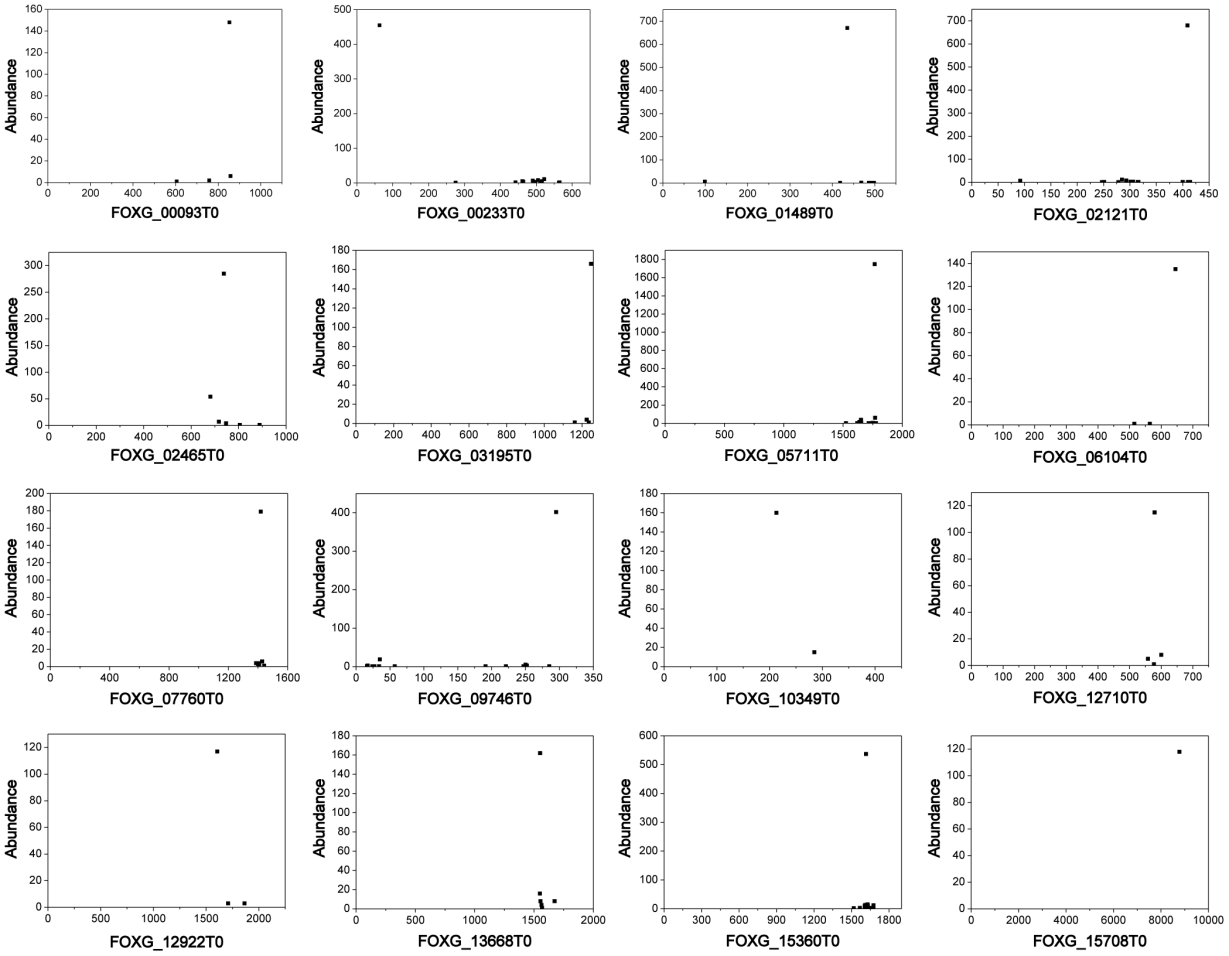
Scatter plot diagrams of degradome data on transcripts. The 16 most abundant signals with high peak-to-total ratio (>0.8) were selected and illustrated. The x and y axis of each diagram represent position of transcripts and the frequency of tags, respectively. Each tag perfectly matching the transcripts was plotted. However, none of these peak signals correspond to predicted cleavage sites of any small RNAs of *F. oxysporum*. These truncated transcripts might be particularly stable decay intermediates or products of special endonuclease.

A degradome library deriving from RNA fragments with 5' monophosphate was constructed and deep sequenced using polyA-enriched RNAs. Illumina/Solexa sequencing produced a total of 13,428,134 single-end reads. Quality screening was performed and 12,468,839 reads were selected for further analysis. Considering the relative low quality in the tail of reads, only front 26 nt were cut out and used for mapping analysis to ensure the specificity and minimize the false positive. Exactly, 454,679 (3.66%), 297,873 (2.40%) and 112,310 (0.90%) of the trimmed tags perfectly matched one or more positions in the genome, genes and transcripts, respectively (**Table 3**). When mapping transcripts, tag abundance, 5′ end alignment positions and cleavage site numbers on each single transcript were recorded and categorized as previously reports [41]. Generally, higher abundance and fewer numbers of cleavage sites are more likely to be the result of endonucleolytic cleavage rather than random degradation products. 16 transcripts with highest peak-to-total ratio were illustrated in **Fig. 3**, in which most signals were located in the 3′ UTR. However, these predominant cleavage sites do not have pairing small RNAs among unknown reads under conditions that no mismatch occurred at positions 10 and 11 of small RNAs. Other top 24 transcripts with strong signals also cannot match appropriate small RNAs either (**Fig. S4**).

**Table 3:** Summary of the degradome library.

	Unique Reads		Total Reads	
	Number	(%)	Number	(%)
Raw reads (35 nt)	—	—	13,428,134	—
High quality reads (35 nt)	—	—	12,468,839	—
Clean reads (26 nt)	1,936,586	(100.00)	12,417,234	(100.00)
Map genome	50,830	(2.62)	454,679	(3.66)
Map genes	34,057	(1.76)	297,873	(2.40)
Map transcripts	18,780	(0.97)	112,310	(0.90)
Transcripts (Total)	—	—	17708	(100.00)
Transcripts (Covered)^a^	—	—	5344	(30.18)

a Transcripts with more than one cleans reads mapping were included.

### Cleavage regulation of small RNAs in *F. oxysporum*

**Figure 4:**
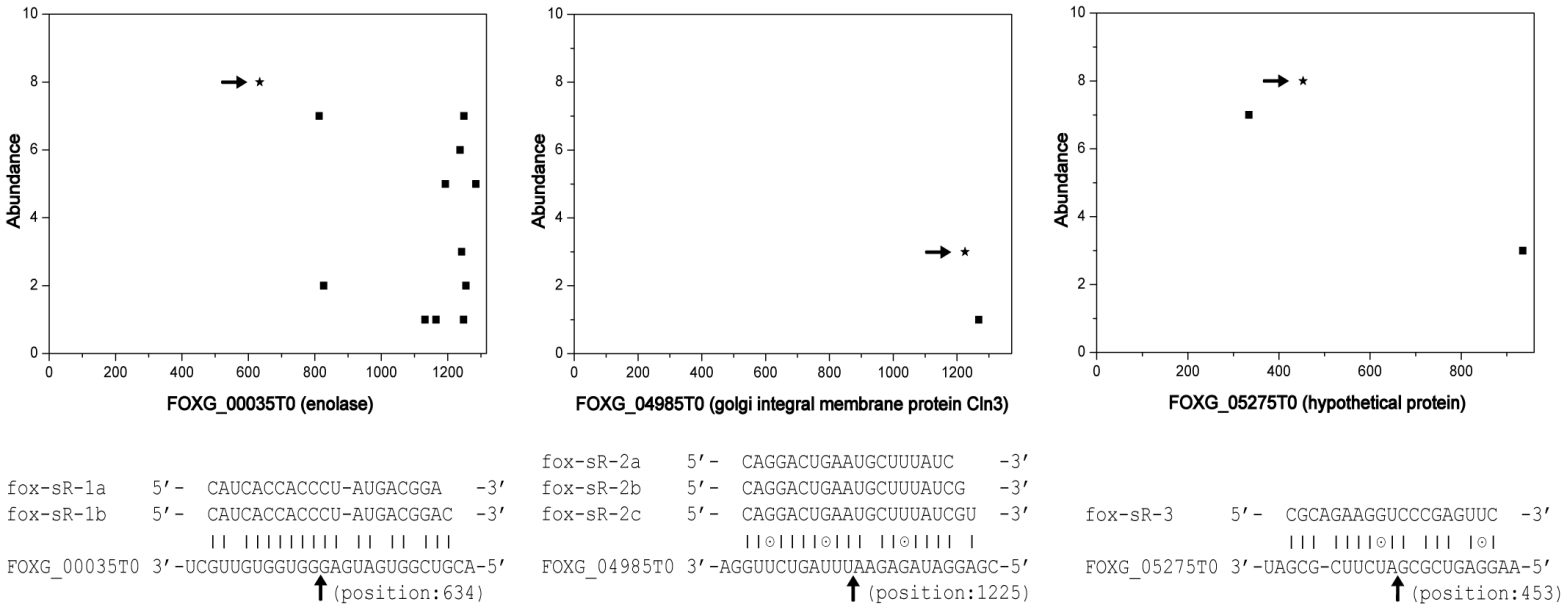
Illustration of three pairs of small RNAs and target transcripts. Scatter plot diagrams show the frequency of tags and their positions on transcripts. The inferred cleavage sites were indicated by the asterisks and arrows. Although the abundance of tags and small RNAs was relative low, the corresponding small RNAs were experimentally validated by RT-PCR (**Fig. 2C**).

PAREsnip program was utilized to make an in-depth survey of cleavage sites in transcripts and corresponding small RNAs in *F. oxysporum* [42]. For each signal found, the alignment between small RNA and target was scored and the credibility was classified into five categories as defined in CleaveLand [43]. In total, 5,344 transcripts were found matching various amounts of degradome tags. Under strict parameters, 147 transcripts were finally identified as cleavaged targets in which 39, 20, 77 and 11 transcripts were belonged to categories 0, I, II and III, respectively (**Fig. S5**). Among category 0, 3 families of small RNAs comprising 6 members were experimentally validated (**Fig. 2C**). The corresponding transcripts, FOXG00035T0, FOXG04985T0 and FOXG05275T0, encoded an enolase, a golgi integral membrane protein (Cln3) and a hypothetical protein, respectively (**Fig. 4**). These results reduced the likelihood of extensive small RNA-guided target cleavage as previously expected.

## Discussion

Different biogenesis pathways of milRNAs identified in *N. crassa* and diverse sets of core proteins of RNA silencing uncovered in different fungi gave a hint that the regulatory functions of fungal milRNAs are ubiquitous but complicated [25], [44]. Recently, deep sequencing technology has been proven to be an effective strategy for discovering novel miRNAs by generating more than ten millions of reads which was believed to cover every single RNA molecular expressed in the studied sample. Besides, the available genome data and the redundant *Ago*, *Dicer* and *RdRP* genes provided prerequisites and foundations for global discovery of milRNAs in *F. oxysporum*. In this work, small RNAs and tags of truncated transcripts were deep sequenced for exploring milRNAs and their potential targets in the filamentous fungus *F. oxysporum*, which would provide valuable evidence for better understanding milRNAs in fungi.

Small RNA populations of *F. oxysporum* was obviously different from plants and animals but shared many similarities with fungal species. First, the type of length distribution of small RNAs was similar with other fungi reported, including *Metarhizium anisopliae* [28], *Trichoderma reesei* [29], *Penicillium marneffei* [30], and *Sclerotinia sclerotiorum* [31], but obviously distinguished from higher eukaryotes in which 21 nt and 24 nt classes were dominant in plants [45] and 22 nt class was abundant in animals [46]. Second, a large proportion of small RNA (nearly 60%) perfectly matching to the rRNA suggested that qiRNAs, a class of DNA damage-induced and rDNA locus derived small RNAs which were also identified in rice (*Oryza sativa*) [47], might be also exist in *F. oxysporum*. The high percentage of rRNA derived small RNA was also found in *P. marneffei* [30], but opposite to other fungi reported previously, ranging from 4.4 to 24.2% [27]–[29], [31]. Moreover, these rRNA-derived small RNAs in *F. oxysporum* with no defined length and strong nucleotide preference at both ends were different from qiRNAs in *N. crassa* which have a strong preference for uridine at the 5′ end (**Fig. S2F**). Next, 14.9% of total reads and 41.1% of unknown reads were located in mitochondrion, sharing similar percentage in *N. crassa* and indicating that the mitochondrion might be a major source for small RNA production in filamentous fungi. Forth, a class of abundant small RNAs equally matching two strands of particular regions was observed during miRCheck prediction, which was similar with the observation in fungus *S. sclerotiorum* [31]. Also like disiRNA (Dicer-independent small interfering RNA) in *N. crassa*, these small RNAs appeared to originate from loci producing overlapping sense and antisense transcripts (data not shown). Last, a large fraction of unique reads matching to exonic regions, which seemed to be ex-siRNA (exonic-siRNAs) reported in *Mucor circinelloides* [48], [49], were much more like degraded products of message RNAs for their low abundance and even distribution pattern. Additionally, the *Fusarium oxysporum* f. sp. *lycopersici* (Fol) lineage-specific regions, including total chromosomes 3, 6, 14, 15 and parts of chromosome 1 and 2, were calculated for small RNA mapping and it's no obvious that they were major origins for small RNA generation. In short, these results confirmed the hypothesis that various types of small RNAs exist in fungi and mechanisms for their production might be universal or alternative among filamentous fungi.

No homologs of conserved miRNAs found in *F. oxysporum* by BLASTing against miRNA database suggested that fungal milRNAs have evolved independently from plants and animals. Given that there is no available standards for distinguishing milRNAs, criteria for miRNA identification in plants and animals were both employed in this study. After multiple rounds of data filtering, 15 milRNA candidates were screened out and 10 of them were finally identified as fox-milRNAs. Comparative analyses of the fox-milRNA precursors among 14 *Fusarium* species, indicated a considerable conservation of the sequence and secondary structure between different *F. oxysporum* strains and also, although to a low extent, between different *Fusarium* species.

Unfortunately, all expected cleavage sites triggered by these fox-milRNAs were not validated by degradome data, suggesting that gene silencing mediated by fox-milRNAs might be independent, at least not completely dependent, on guiding transcripts cleavage [50]. Further analysis of degradome data showed that most strong signals were located in the 3′-UTR of target transcripts and no complementary small RNAs could match these coordinates appropriately. In category 0 of PAREsnip results, the majority of small RNAs were low abundant and failed to be experimentally verified by RT-PCR. Only 6 small RNAs corresponding to three truncated targets were identified but their secondary structures could not form typical hairpins to be classified as milRNAs. These evidences indicated that small RNAs are not major factors causing mRNA cleavage in *F. oxysporum*. Like some animal and plant miRNAs as well as *milRNA-1* in *N. crassa* [25], fox-milRNAs might regulate target gene expression by other modes, such as translational repression [51] or DNA methylation [52], which need further investigations.

Taken together, this study made a genome-wide survey on milRNAs and their potential targets in *F. oxysporum*. 8 families of milRNAs were identified referring to the standards of miRNA prediction in plants and animals. Nevertheless, the newly identified fox-milRNAs could not direct cleavage of predicted targets. In-depth analysis of small RNA and degradome libraries demonstrated many particularities involved in fungal small RNAs which were quite different from that of plants and animals. Our discovery provided significant amount of data and offered a new perspective for better understanding of the biogenesis and functions of small RNAs and miRNAs in eukaryotic kingdoms.

## Conclusions

In this study, small RNA and degradome libraries were constructed and subsequently deep sequenced for investigating milRNAs and their potential cleavage targets on the genome level in the filamentous fungus *Fusarium oxysporum* f. sp. *lycopersici.* As a result, no conserved miRNAs was found by a cross check against miRBase. Further analysis showed that the small RNA population of *F. oxysporum* shared some common features with the small RNAs from *N. crassa* and some other fungi. According to the known standards of miRNA prediction in plants and animals, milRNA candidates from 8 families (comprising 19 members) were screened out and identified. However, none of them could trigger target cleavage based on the degradome data. Moreover, most major signals of cleavage in transcripts could not match appropriate complementary small RNAs, suggesting that other modes for milRNA-mediated gene regulation could exist in *F. oxysporum*. In addition, the PAREsnip program was utilized for comprehensive analysis and 3 families of small RNAs leading to transcript cleavage were experimentally validated. Altogether, our findings provided direct evidence for milRNAs research and implied a more sophisticated system for small RNA generation and regulatory pathways in filamentous fungi.

## Materials and Methods

### *Fusarium oxysporum* strain and RNA isolation

The *Fusarium oxysporum* f. sp. *lycopersici* strain used in this study was kindly offered by Dr. Zhenchuan Mao (Institute of Vegetables and Flowers, Chinese Academy of Agricultural Sciences). The virulence of this strain was confirmed by pathogenicity tests. The strain was taken to be activated and cultivated on PDA plates for 7 days. Mycelium was collected for RNA extraction using TRIzol reagent (Invitrogen) according to the manufacturer's instructions. The quality and quantity of RNA were determined by formaldehyde denaturing agarose gel electrophoresis and NanoDrop 1000 Spectrophotometer (NanoDrop Technologies Inc., Wilmington, DE). Total RNA was used for small RNA and degradome libraries construction as well as RT-PCR experiments (see below).

### Small RNA deep sequencing

Small RNA library was constructed using Small RNA Sample Prep Kit v1.5 (Illumina) according to manufacturer's instructions. Briefly, low molecular weight RNAs (15∼30 nt) were isolated from 100 ug total RNA by a 15% TBE-urea denaturing polyacrylamide gel electrophoresis (PAGE) and ligated sequentially to specific adaptors at the 5′ and 3′ ends. After reverse transcription, appropriate amplification and purification, the cDNA products were checked for amount and quality by Agilent 2100 BioAnalyzer and deep sequenced through Illumina/Solexa GAII platform.

### Degradome library construction

The degradome library was constructed following the previously published work as described by Ma *et al* [53]. Different from the conventional protocol for PARE [54], a method similar to that used for transcriptome sequencing was utilized instead of the restriction endonuclease strategy. Approximately 200 ng of poly(A) RNA containing the 5′-monophosphates was used for adaptor ligation directly skipping the initial RNaseIII fragmentation step. Single-end deep sequencing read the first 35 nt that represented the 5′ends of the original truncate RNA fragments. Sequencing raw data of small RNA and degradome libraries was deposited at the NCBI Sequence Read Archive (SRA) under accession no. SRP034883.

### Data analysis of small RNA and miRNA prediction

Perl programs were designed and written for data analysis in this section. For data preprocessing, the raw reads were firstly screened according to sequencing quality. The reads with more than four Phred scores below 15 (q15< = 4) were removed. High quality reads were subsequently processed by triming adaptors, collapsing repeats and recording abundance. Only reads with reliable 3′ adaptor tail but no ‘N’ or adaptor contaminants ahead were cut out to generate clean reads. Clean reads ranging from 18∼30 nt were mapped to *F. oxysporum* genome using SOAP [55] and only perfectly matched reads were subjected to further analysis. To perform chromosomal distribution analysis and small RNA loci annotation, average abundance was defined and calculated for reads with multiple loci. After removing rRNA, tRNA and snoRNA based on Rfam [56], SILVA [57] and GtRNAdb [58] databases, the remaining reads were designated as unknown reads and used for miRNA prediction. For conserved miRNA analysis, unknown reads were searched against the mature miRNAs in miRBase (Release 19.0) [59] using BLAST (-m 8). Customized Perl programs were used to find homologs under two mismatches or difference in length. For novel miRNA prediction, miRDeep2 [60] and miRCheck [61] were both used according to the criteria of animals and plants, respectively. To reduce the computation, unknown reads with abundance less than 3 times or loci number larger than 20 were excluded. miRDeep2 software was implemented with default and no reference miRNA options. In miRCheck approach, 100 bp flanks around each locus were extracted where adjacent loci less than 50 bp were combined and regarded as one. Then, each context was predicted by RNAfold [62] for secondary structure and examined by miRCheck with default parameters. Given that miRNAs were derived from single strand precursors relative to siRNAs, an additional step was performed for single-strand verification. Only hairpins with coverage rate more than 10-fold between sense and antisense were retained (**Fig. S6**). Finally, outputted hairpins were clustered and mature miRNAs were manually checked for identification of *F. oxysporum* milRNA candidates. psRNATarget online was used for target prediction [40]. Detailed procedures for small RNA analysis and milRNA prediction can be seen in **Fig. S1**.

### Bioinformatic analysis of degradome

Quality screening step was performed initially and low-quality nucleotides were trimmed at the tails of raw reads. To improve the mapping accuracy and keep the specificity, only first 26 nt of reads were extracted and collapsed as unique tags. PAREsnip program was used with stringent parameters to comprehensively analyze regulatory interactions leading to mRNA cleavage [42]. Draft genome assembly and annotated file of *F. oxysporum* were produced by the Broad Institute (http://www.broad.mit.edu/).

### RT-PCR assay for miRNAs

The experimental validation of small RNAs was performed using miRcute miRNA First-Strand cDNA synthesis kit (Tiangen, KR201) and miRcute miRNA qPCR detection kit (Tiangen, FP401). All reactions were repeated twice with two biological samples and detected by 2% (w/v) agarose gel electrophoresis. Primers used in this section were listed in **Table S6**.

## Supporting Information

Figure S1 **Flow chart of small RNA analysis and milRNA prediction.**
(TIF)

Figure S2 **Chromosomal distribution and nucleotide bias of small RNAs.** A: Chromosomal distribution of unknown reads. B: Length distribution of rRNA-derived small RNAs. Nucleotide biases at both ends of clean reads (C), mapped reads (D), unknown reads (E) and rRNA-derived small RNAs (F). G: Histograms of small RNA coverage on fox-milRNA precursors. Base coverage was counted and recorded when mapping small RNAs onto genome. Short bars indicate the location of mature milRNAs. The asterisk represents the milRNA* sequence.
(TIF)

Figure S3 **Alignment of the fox-milRNA-4 precursors in 11** ***Fusarium*** **species.**
(TIF)

Figure S4 **Scatter plot diagrams of cleavage sites on transcripts with high abundance.** Based on the analysis results of degradome data, 24 transcripts with high mapping abundance but no obvious single peak (peak-to-total ratio <0.8) were listed.
(TIF)

Figure S5 **Output of PAREsnip program under stringent parameters.**
(PDF)

Figure S6 **Alignments between milRNA candidates and precursors passing miRCheck.**
(DOC)

Table S1 **MicroRNA homologs found in** ***F. oxysporum*** **by BLASTing against miRBase 19.0.**
(DOCX)

Table S2 **Results of milRNA prediction using miRDeep2 software.**
(DOCX)

Table S3 **Results of milRNA prediction through miRCheck pipeline.**
(DOCX)

Table S4 **Target prediction of fox-milRNAs using psRNATarget online.**
(DOCX)

Table S5 **Conservation analysis of fox-milRNA precursors in closely related** ***Fusarium*** **species.** The precursor sequences of milRNAs were BLAST against related genomes. Alignment length more than 95% was calculated.
(DOCX)

Table S6 **Primers used for RT-PCR experiments in this study.**
(DOCX)
