# Supplementary figures and images for: Exploring MicroRNA-Like Small RNAs in the Filamentous Fungus Fusarium oxysporum
Source: PLoS One. 2014 Aug 20;9(8):e104956. doi: 10.1371/journal.pone.0104956 (PMC4139310; doi:10.1371/journal.pone.0104956)

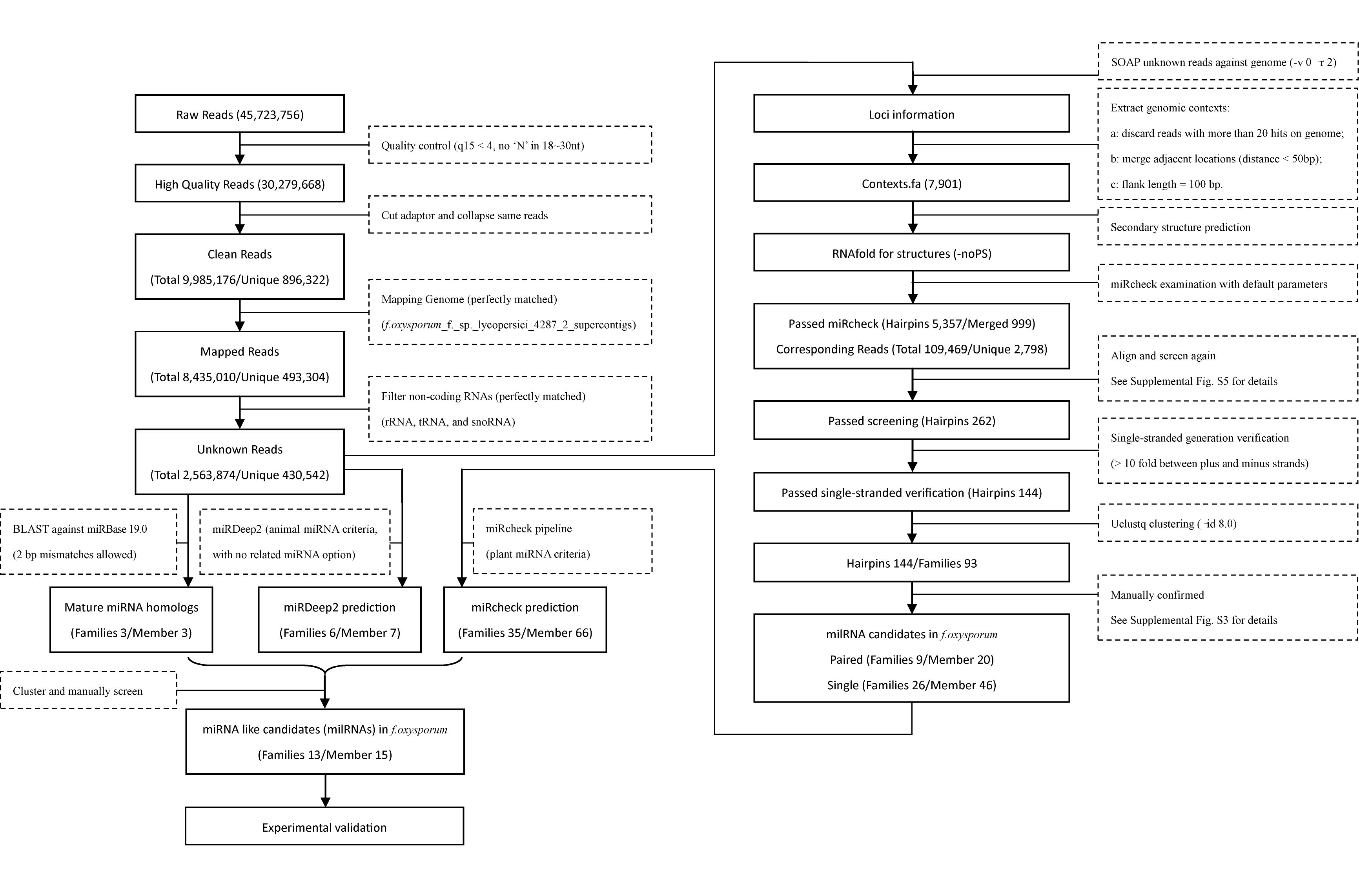

Supplement: Figure S1 — Flow chart of small RNA analysis and milRNA prediction. (TIF) [file pone.0104956.s001.tif]

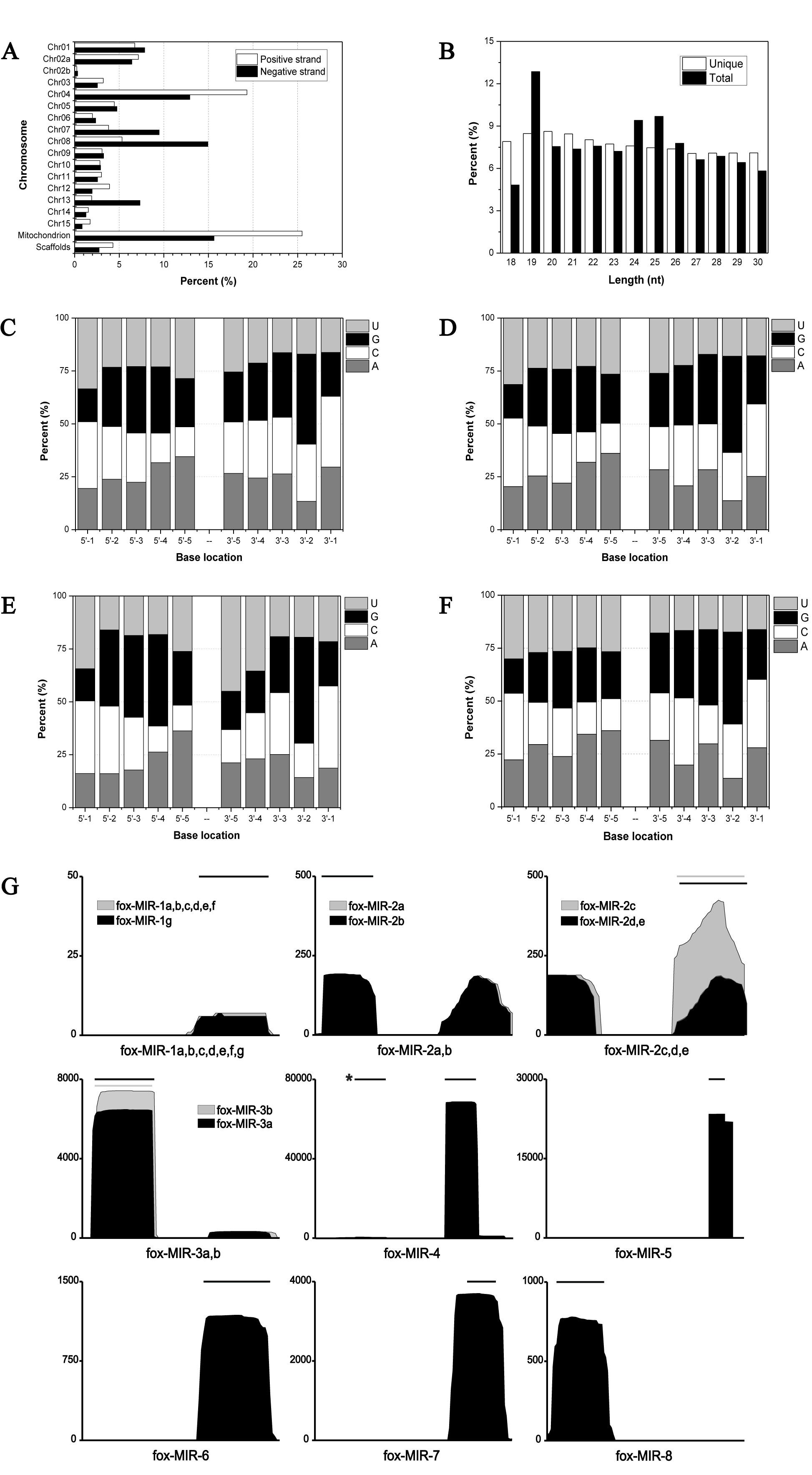

Supplement: Figure S2 — Chromosomal distribution and nucleotide bias of small RNAs. A: Chromosomal distribution of unknown reads. B: Length distribution of rRNA-derived small RNAs. Nucleotide biases at both ends of clean reads (C), mapped reads (D), unknown reads (E) and rRNA-derived small RNAs (F). G: Histograms of small RNA coverage on fox-milRNA precursors. Base coverage was counted and recorded when mapping small RNAs onto genome. Short bars indicate the location of mature milRNAs. The asterisk represents the milRNA* sequence. (TIF) [file pone.0104956.s002.tif]

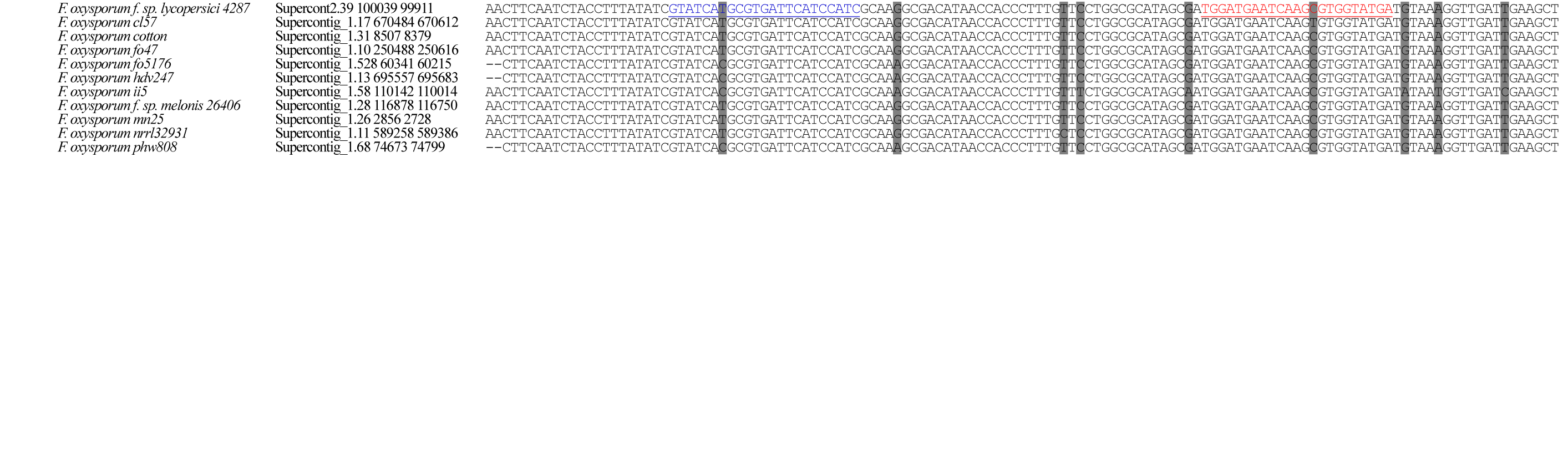

Supplement: Figure S3 — Alignment of the fox-milRNA-4 precursors in 11 Fusarium species. (TIF) [file pone.0104956.s003.tif]

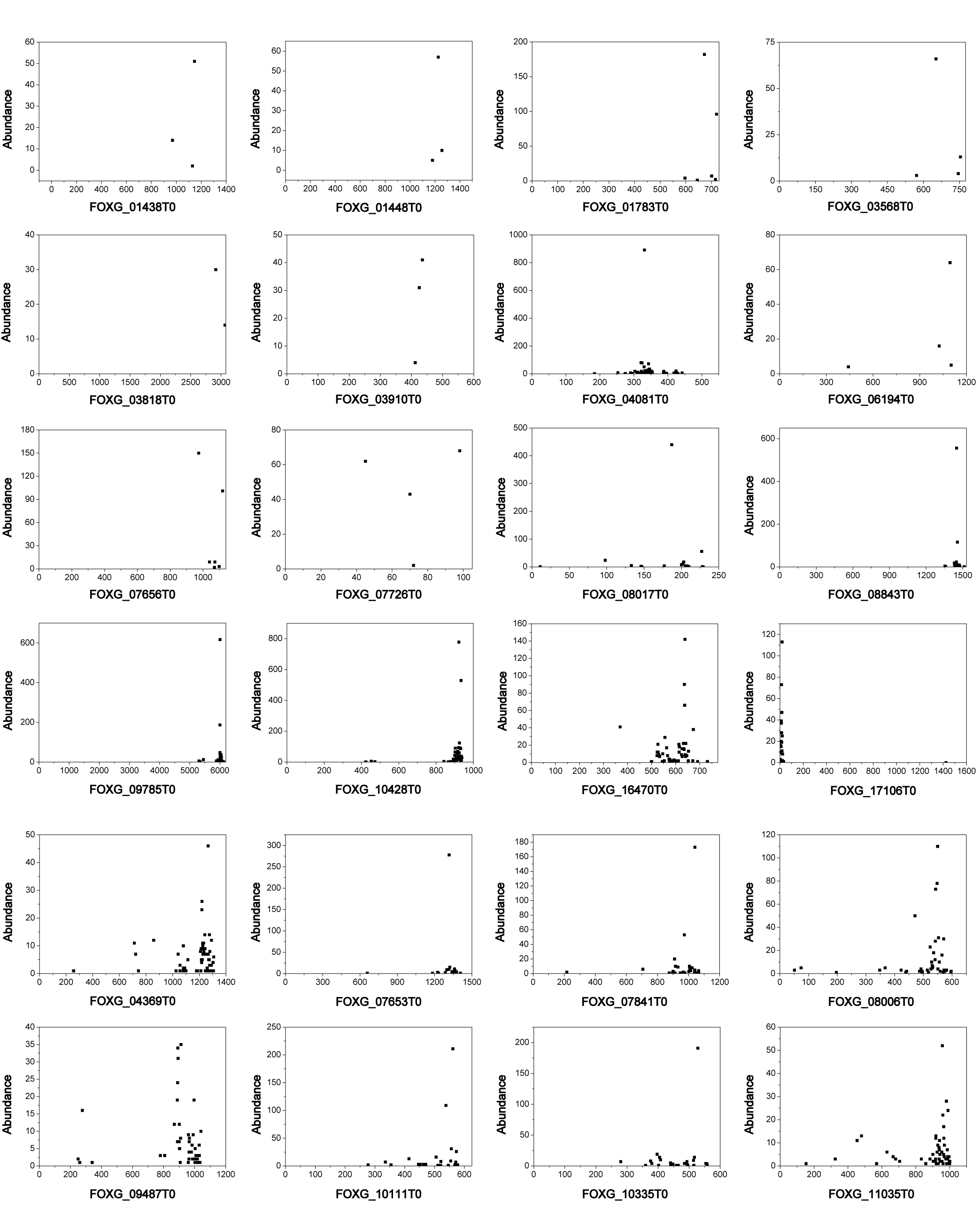

Supplement: Figure S4 — Scatter plot diagrams of cleavage sites on transcripts with high abundance. Based on the analysis results of degradome data, 24 transcripts with high mapping abundance but no obvious single peak (peak-to-total ratio <0.8) were listed. (TIF) [file pone.0104956.s004.tif]
