# Supplementary material for: Exploring MicroRNA-Like Small RNAs in the Filamentous Fungus Fusarium oxysporum
Source: PLoS One. 2014 Aug 20;9(8):e104956. doi: 10.1371/journal.pone.0104956 (PMC4139310; doi:10.1371/journal.pone.0104956)

FOXG_03195T0 | Fusarium oxysporum f. sp. lycopersici 4287 glucosamine-6-phosphate isomerase (1281 nt)

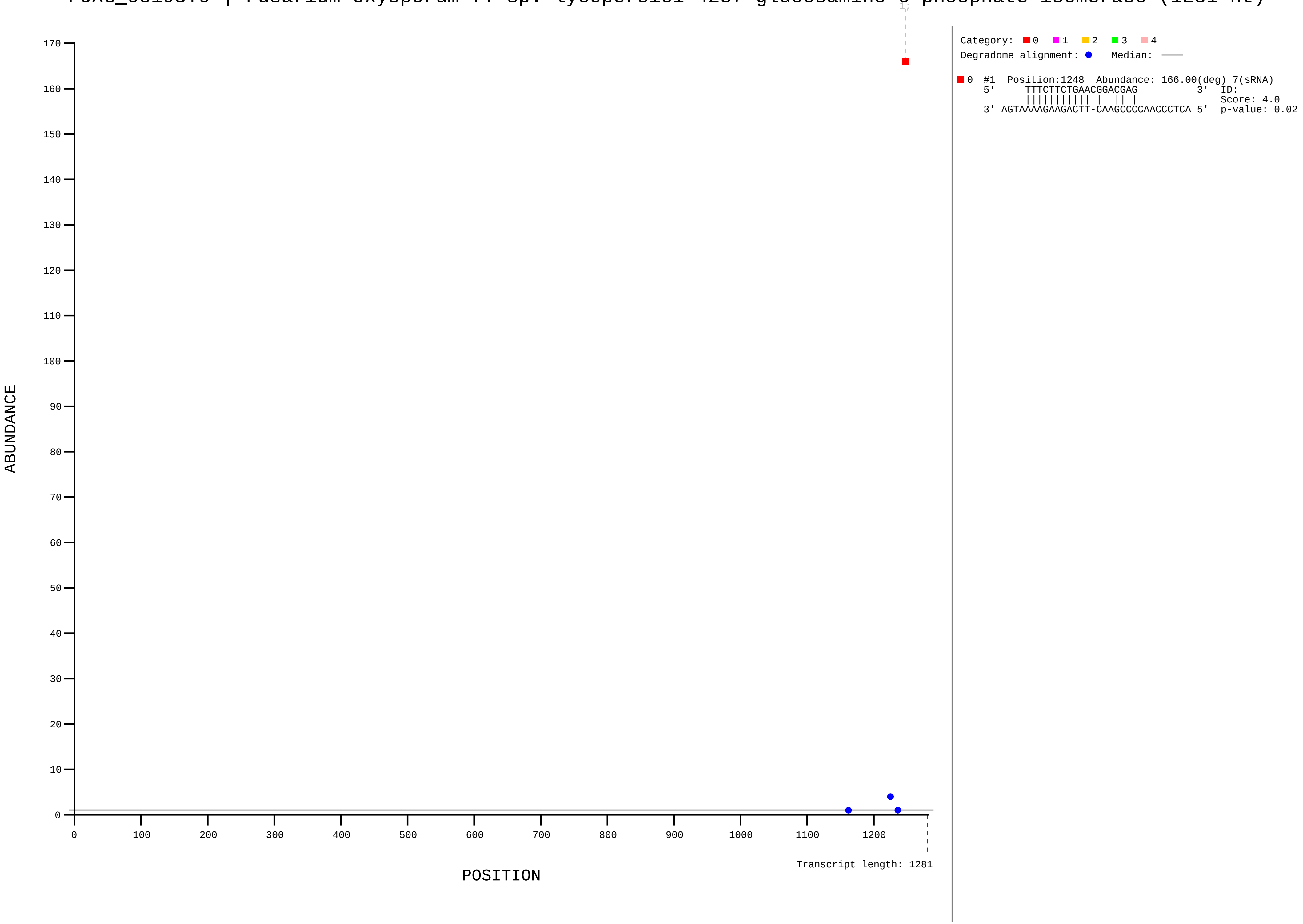

FOXG_10349T0 | Fusarium oxysporum f. sp. lycopersici 4287 hypothetical protein (405 nt)

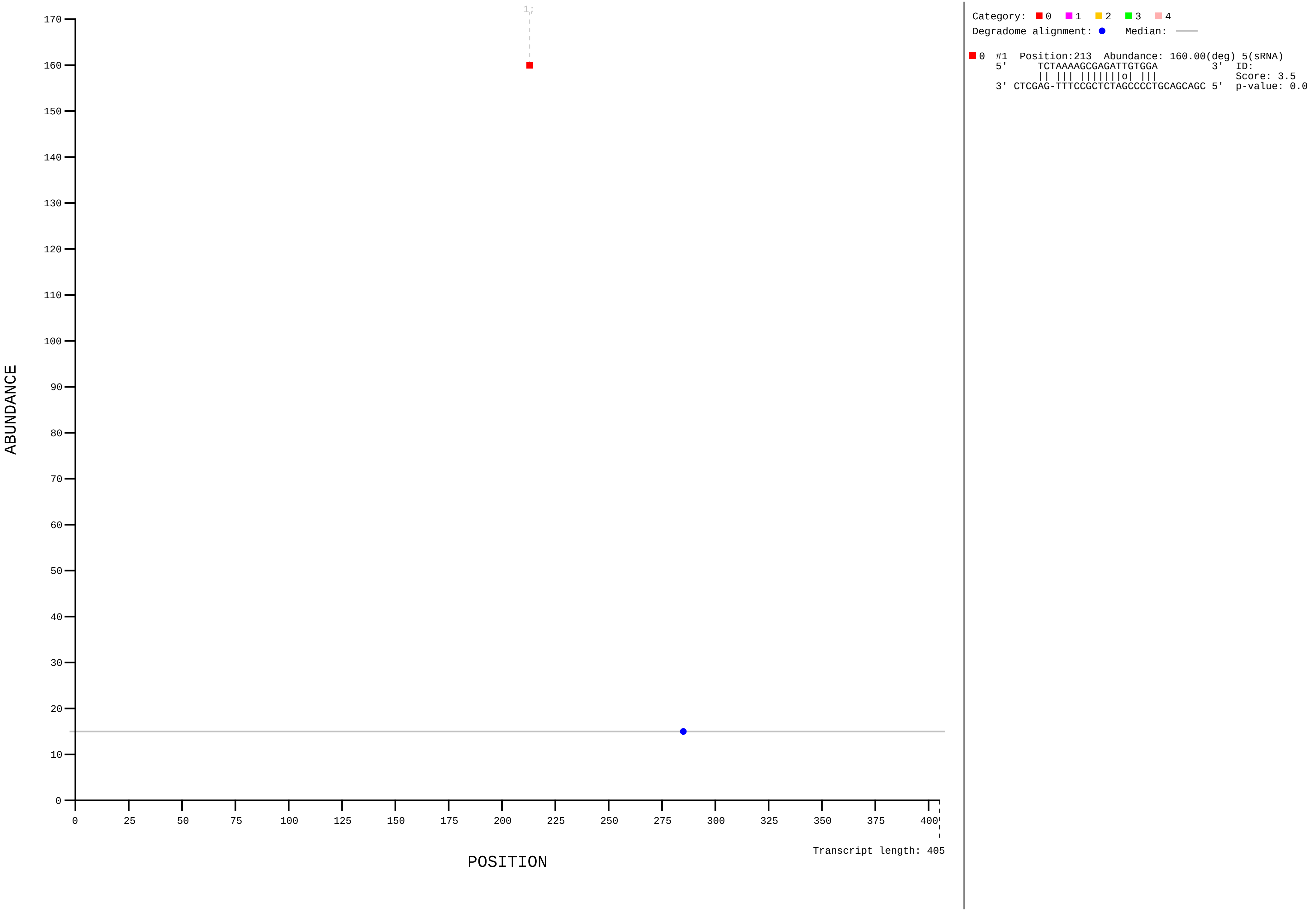

FOXG_08089T0 | Fusarium oxysporum f. sp. lycopersici 4287 hypothetical protein (624 nt)

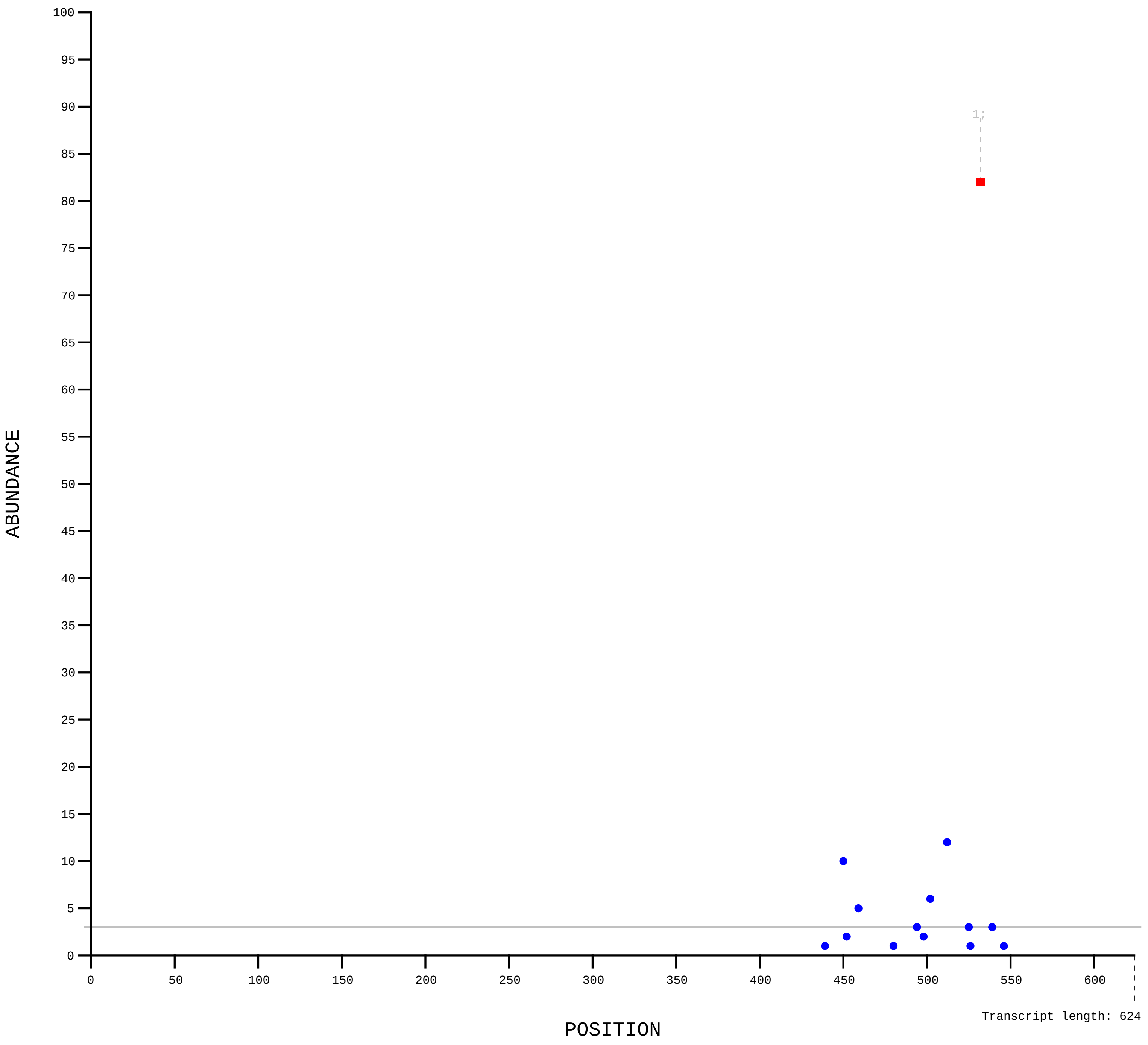

Category: 0 1 2 3 4
Degradome alignment: Median:

0 #1 Position:532 Abundance: 82.00(deg) 5(sRNA)
5' CATAACCCGGAGGTCGCACG 3' ID:
|||| || |||||||| | | Score: 4.0
3' AGGAGTATAGGTCCTCCAGCCTACGGAGCTGG 5' p-value: 0.0

FOXG_05954T0 | Fusarium oxysporum f. sp. lycopersici 4287 hypothetical protein (702 nt)

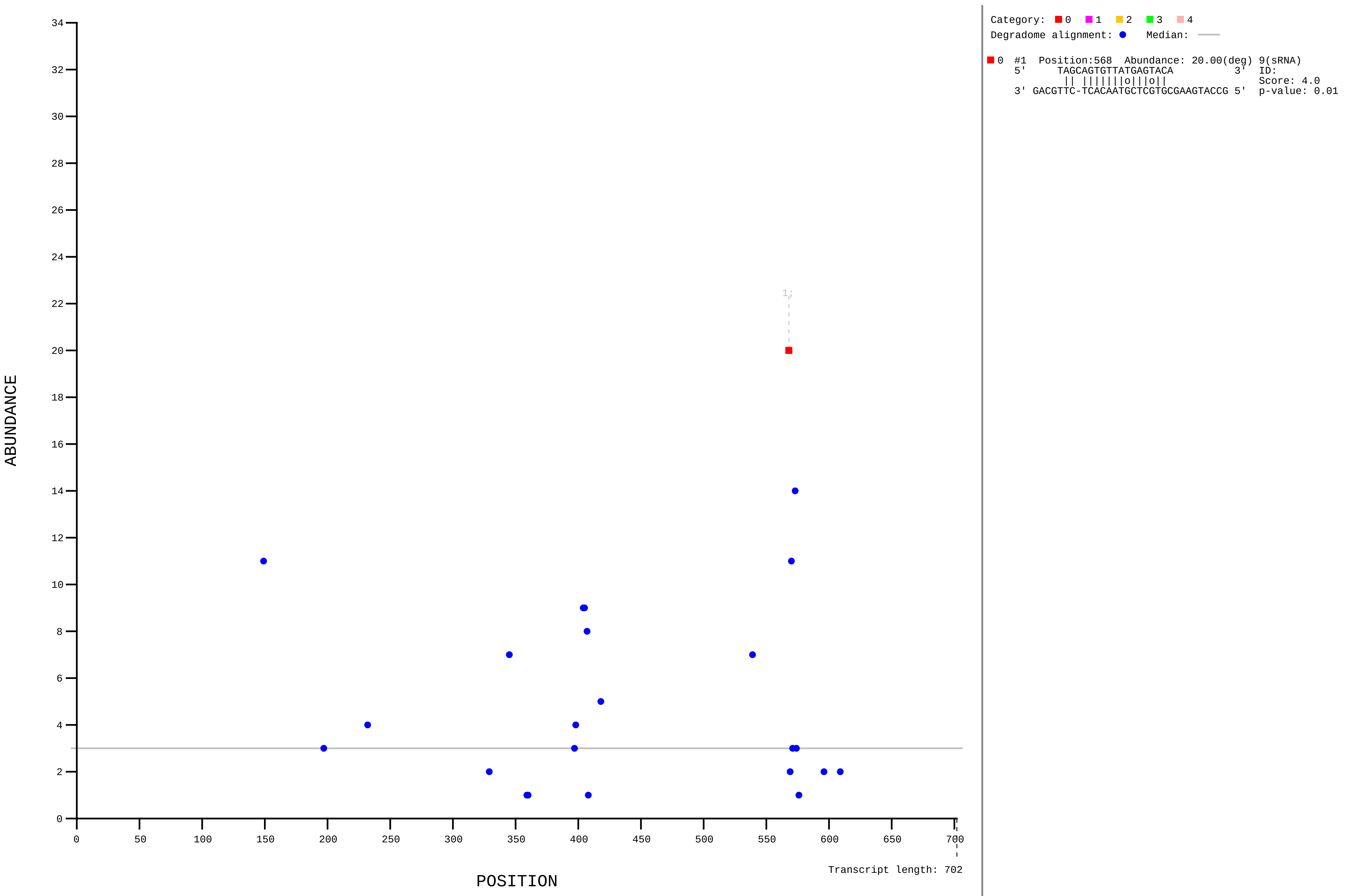

FOXG_10323T0 | Fusarium oxysporum f. sp. lycopersici 4287 hypothetical protein (759 nt)

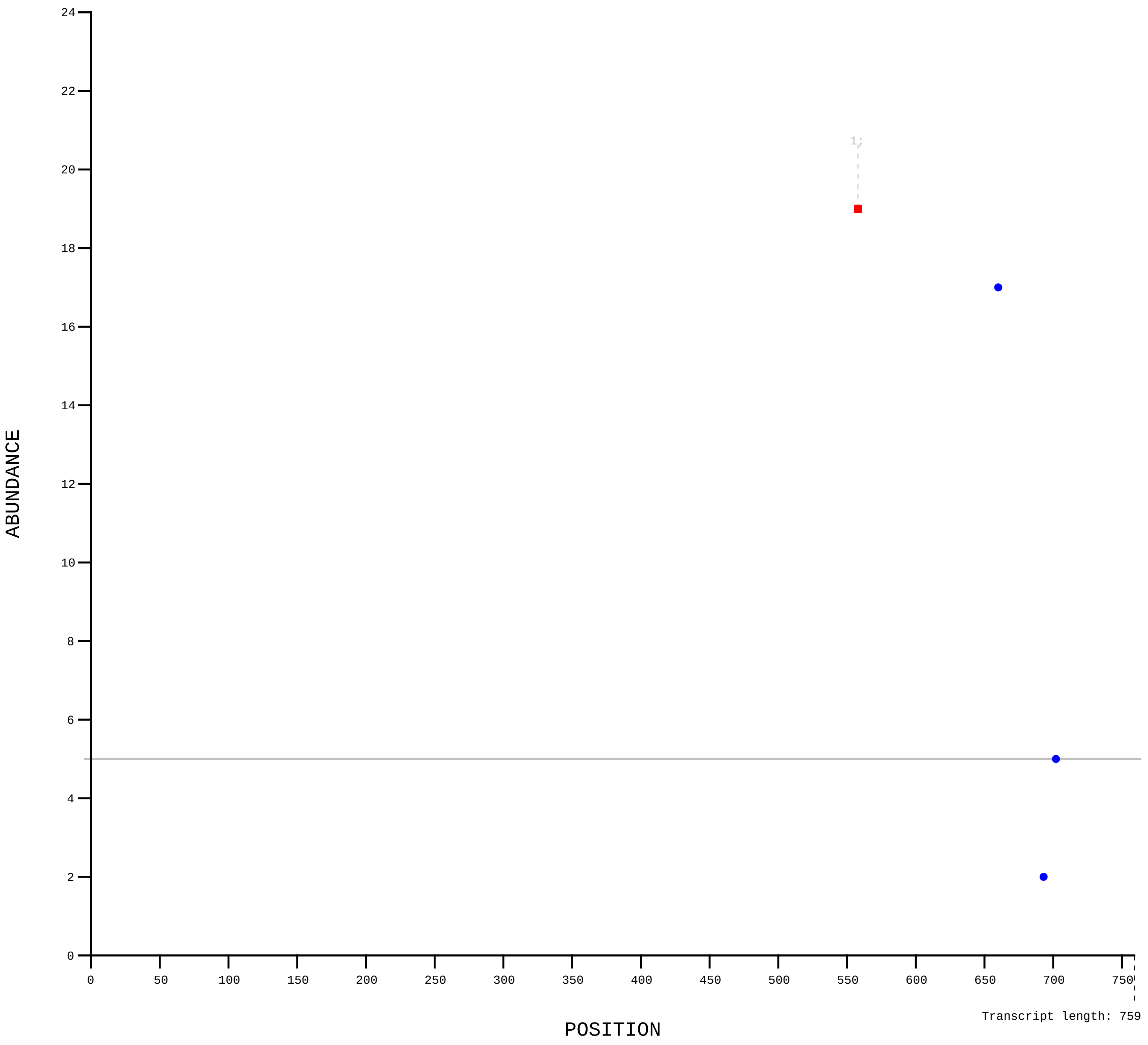

Category: 0 1 2 3 4
Degradome alignment: Median:

0 #1 Position:558 Abundance: 19.00(deg) 12(sRNA)
```
5'      TTCGATTCCCAGATTACGCA         3'  ID:
        | |o|| |||||||o| |||             Score: 4.0
3' GTACATGTTATGGGTCTAGTACGTCAAAAACG 5'  p-value: 0.0
```

FOXG_00784T0 | Fusarium oxysporum f. sp. lycopersici 4287 hypothetical protein (849 nt)

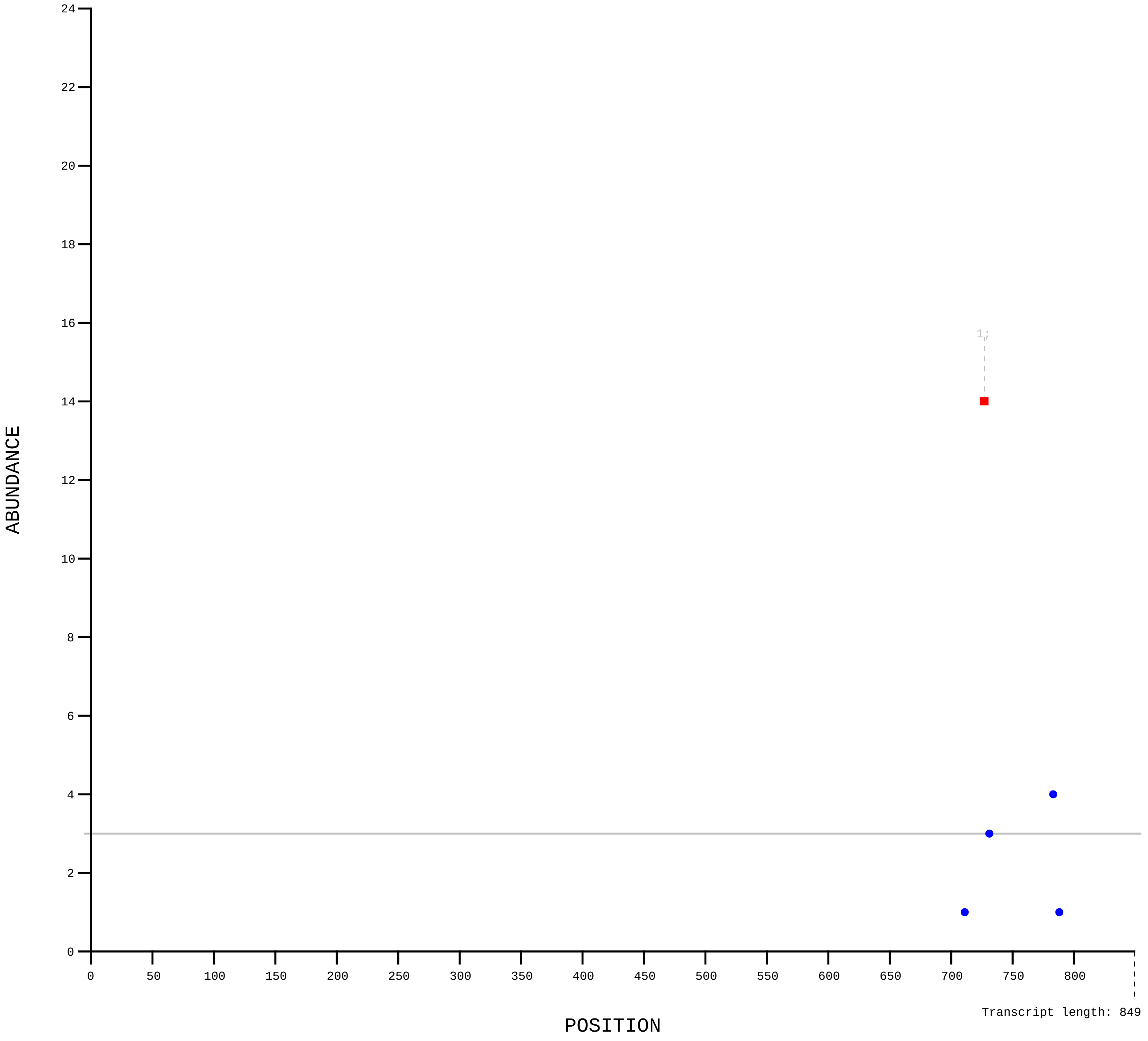

Category: 0 1 2 3 4
Degradome alignment: Median:

```
0  #1  Position:727  Abundance: 14.00(deg) 138(sRNA)
   5'     CGGGCTTGAGAAATACAGC          3'  ID:
          |o||||||||| |o | ||              Score: 4.0
   3' CTAGGTCCGAACTCTCTGAG-CGATAGCTTCG 5'  p-value: 0.0
```

FOXG_06358T0 | Fusarium oxysporum f. sp. lycopersici 4287 hypothetical protein (927 nt)

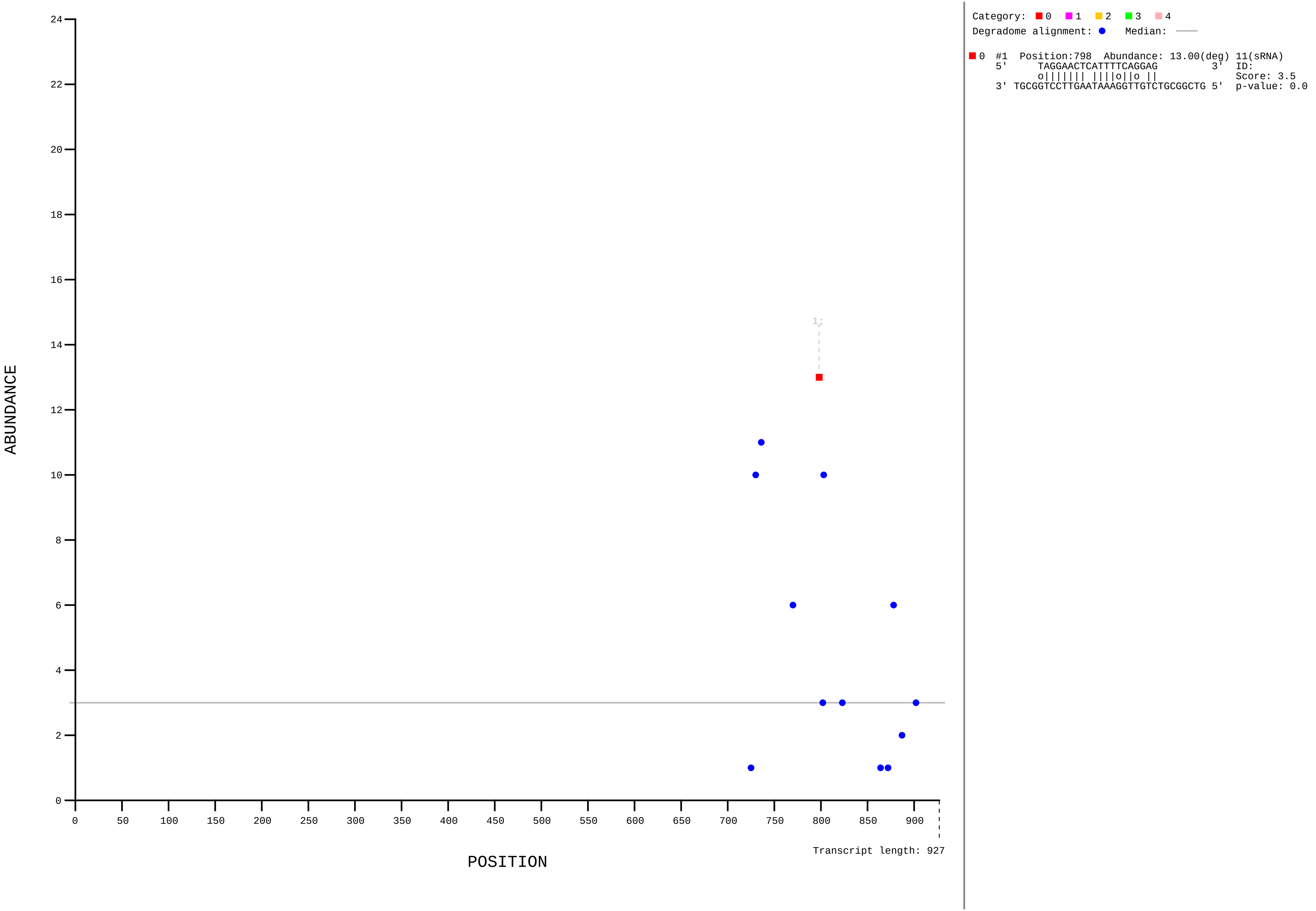

FOXG_02741T0 | Fusarium oxysporum f. sp. lycopersici 4287 hypothetical protein (7659 nt)

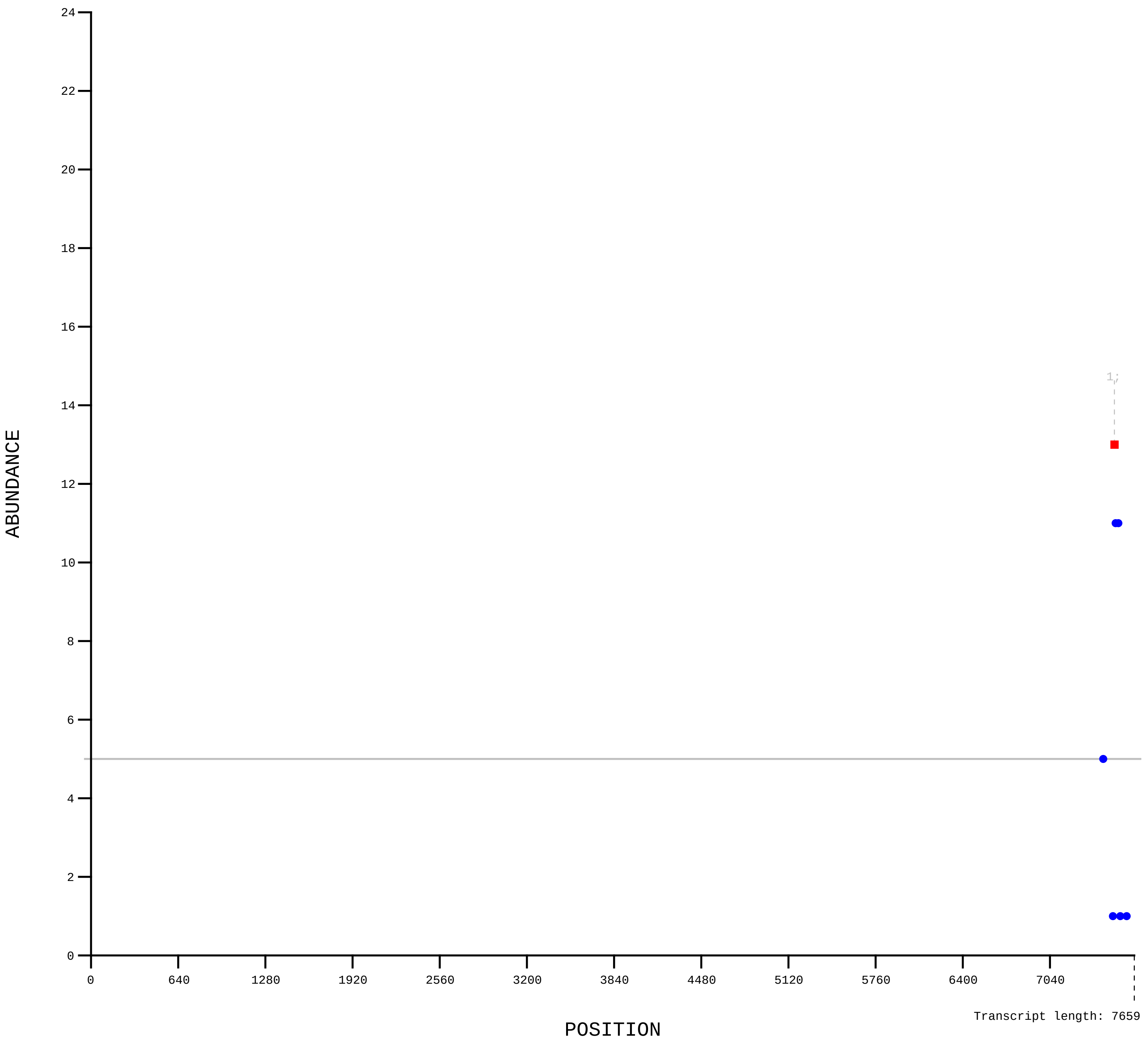

Category: 0 1 2 3 4
Degradome alignment: Median:

0 #1 Position:7513 Abundance: 13.00(deg) 10(sRNA)

```
5'     TAAACTGCCAATTTCTGA-CT        3'  ID:
       ||||| |||||||o|| | ||            Score: 3.5
3' AGAGATTTGTCGGTTAAGGAGTTGAGTTGGCG 5'  p-value: 0.0
```

FOXG_02954T0 | Fusarium oxysporum f. sp. lycopersici 4287 3-ketoacyl-CoA thiolase B (1254 nt)

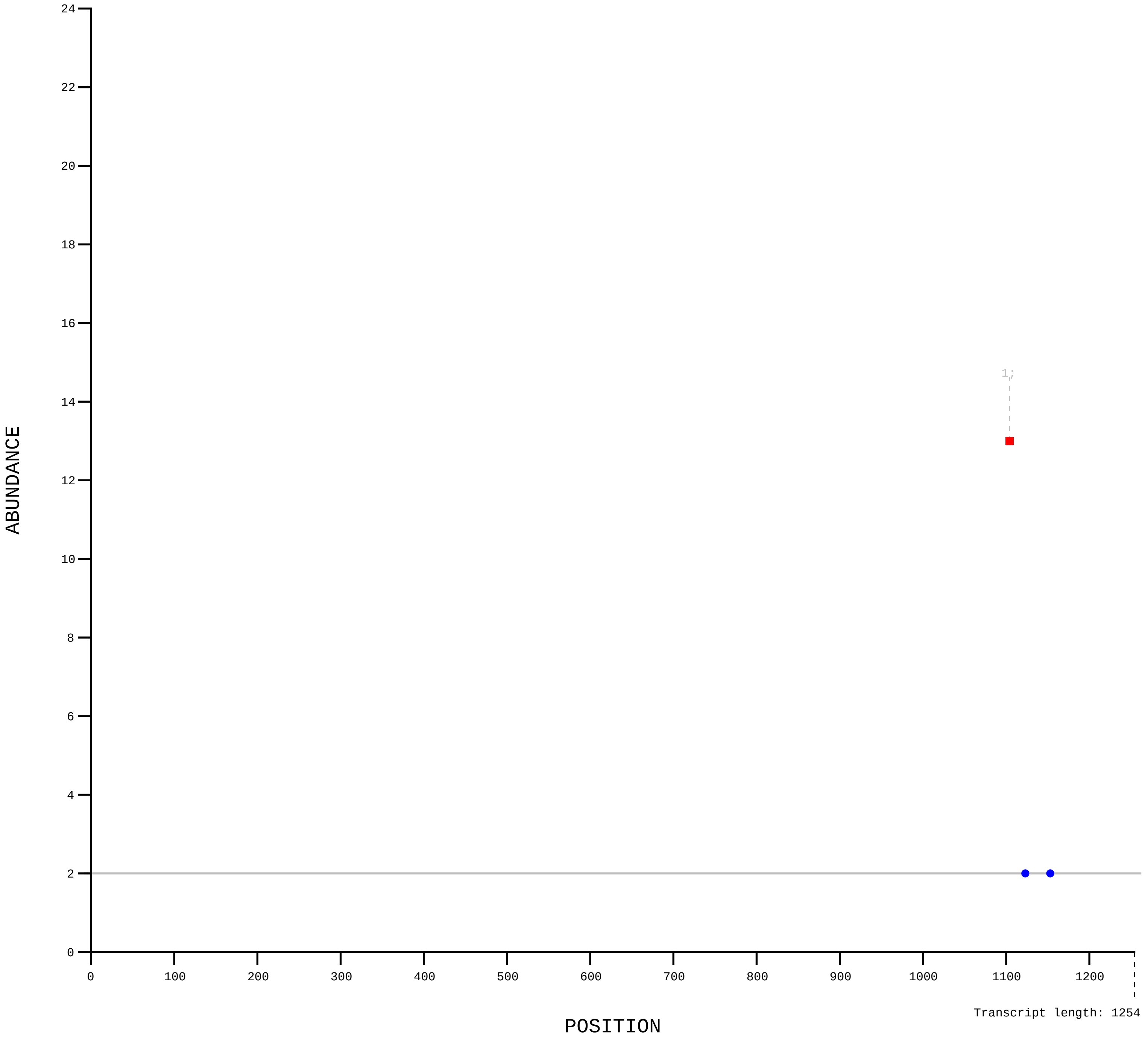


```
Category: 0  1  2  3  4
Degradome alignment:    Median:

0  #1  Position:1104  Abundance: 13.00(deg) 6(sRNA)
   5'     AAGCGCGACAGCGCTTCGG           3'  ID:
           |||||o| |||||o|| |               Score: 4.0
   3' CAGGGTCGCGTTATCGCGGAGGCAGTCCCAAC 5'  p-value: 0.0
```

FOXG_12677T0 | Fusarium oxysporum f. sp. lycopersici 4287 hypothetical protein (1251 nt)

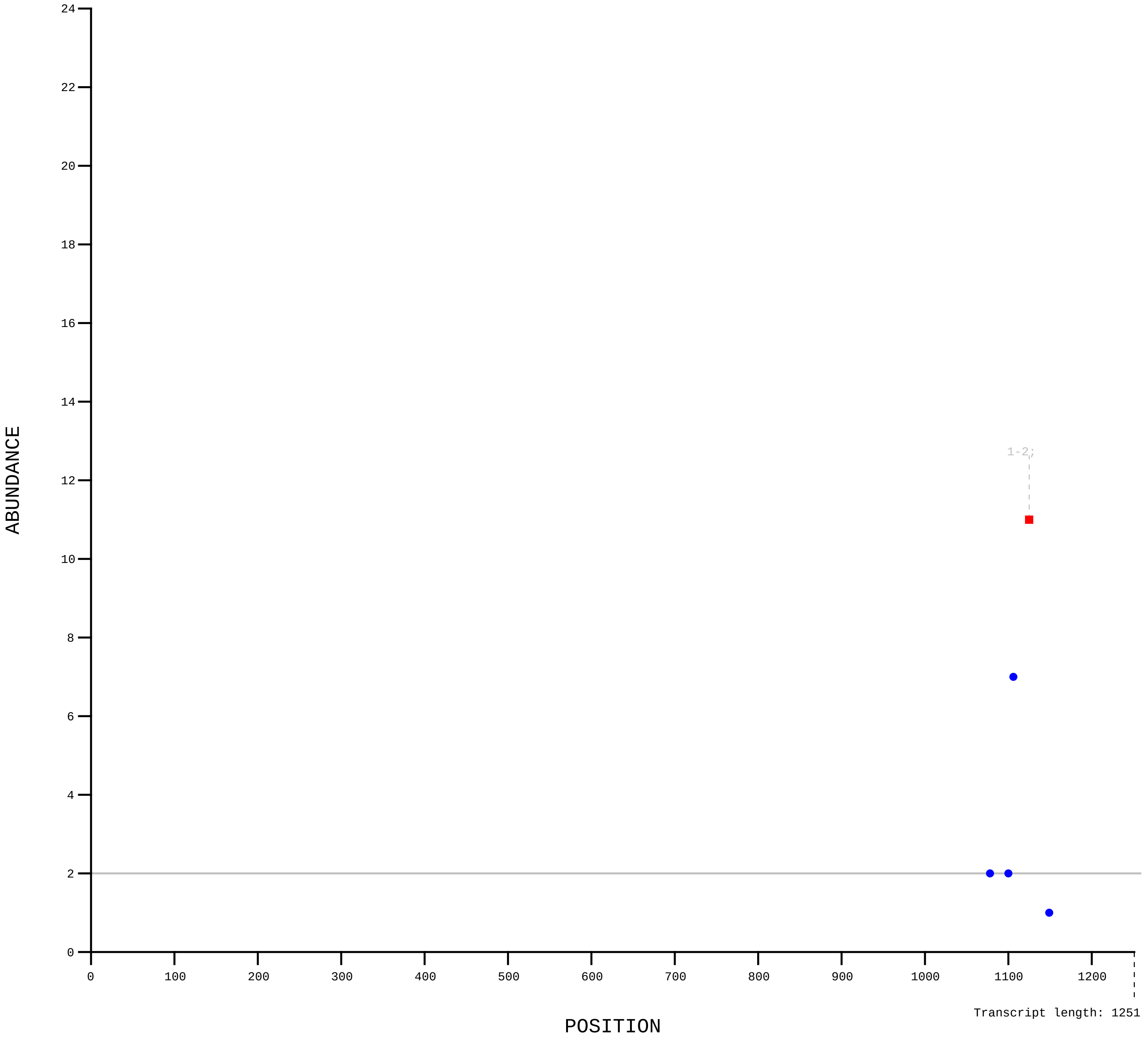

Category: 0 1 2 3 4

Degradome alignment: ● Median: —

```
0  #1  Position:1125  Abundance: 11.00(deg) 9(sRNA)
   5'     TTTTTCTCCTTTGAAGGCA          3'  ID:
          |||||||| |||oo| | ||             Score: 4.0
   3' GACCAAAAAGA-GAAGTTACGGTTATCGTCGC 5'  p-value: 0.05

0  #2  Position:1125  Abundance: 11.00(deg) 6(sRNA)
   5'     TTTTTCTCCTTTGAAGGCAA         3'  ID:
          |||||||| |||oo| | |||            Score: 4.0
   3' GACCAAAAAGA-GAAGTTACGGTTATCGTCGC 5'  p-value: 0.01
```

FOXG_03515T0 | Fusarium oxysporum f. sp. lycopersici 4287 elongation factor 1-alpha (1329 nt)

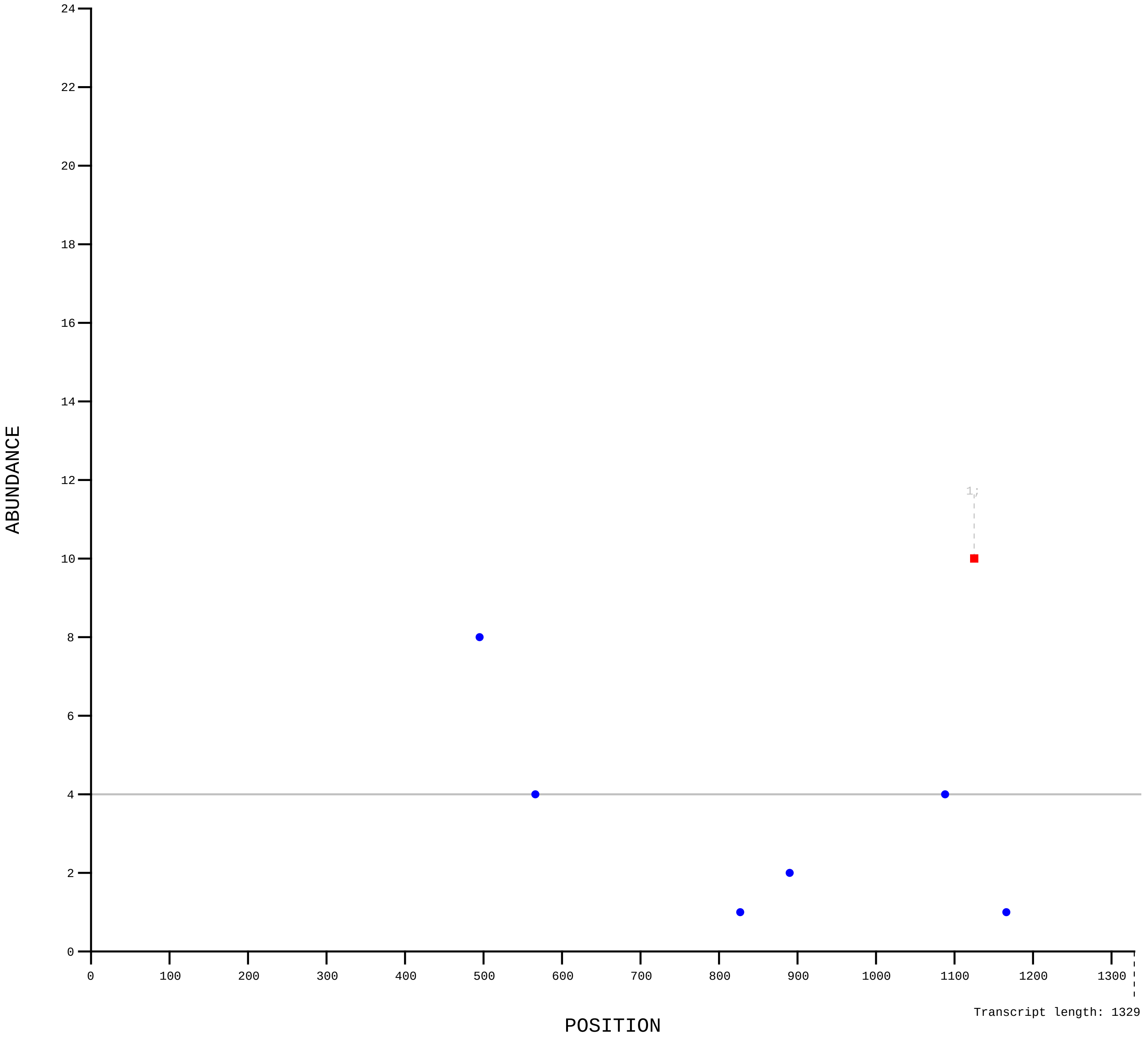

Category: 0 1 2 3 4
Degradome alignment: ● Median:

```
0  #1  Position:1125  Abundance: 10.00(deg) 14(sRNA)
   5'     ACCAGACTCGACGGAACTC          3'  ID:
          |||||||| || | ||||               Score: 4.0
   3' TCAGTGGTCTGAACTAC-TTGAACCCCCGCCG 5'  p-value: 0.0
```

FOXG_01064T0 | Fusarium oxysporum f. sp. lycopersici 4287 N-acetyltransferase 10 (3201 nt)

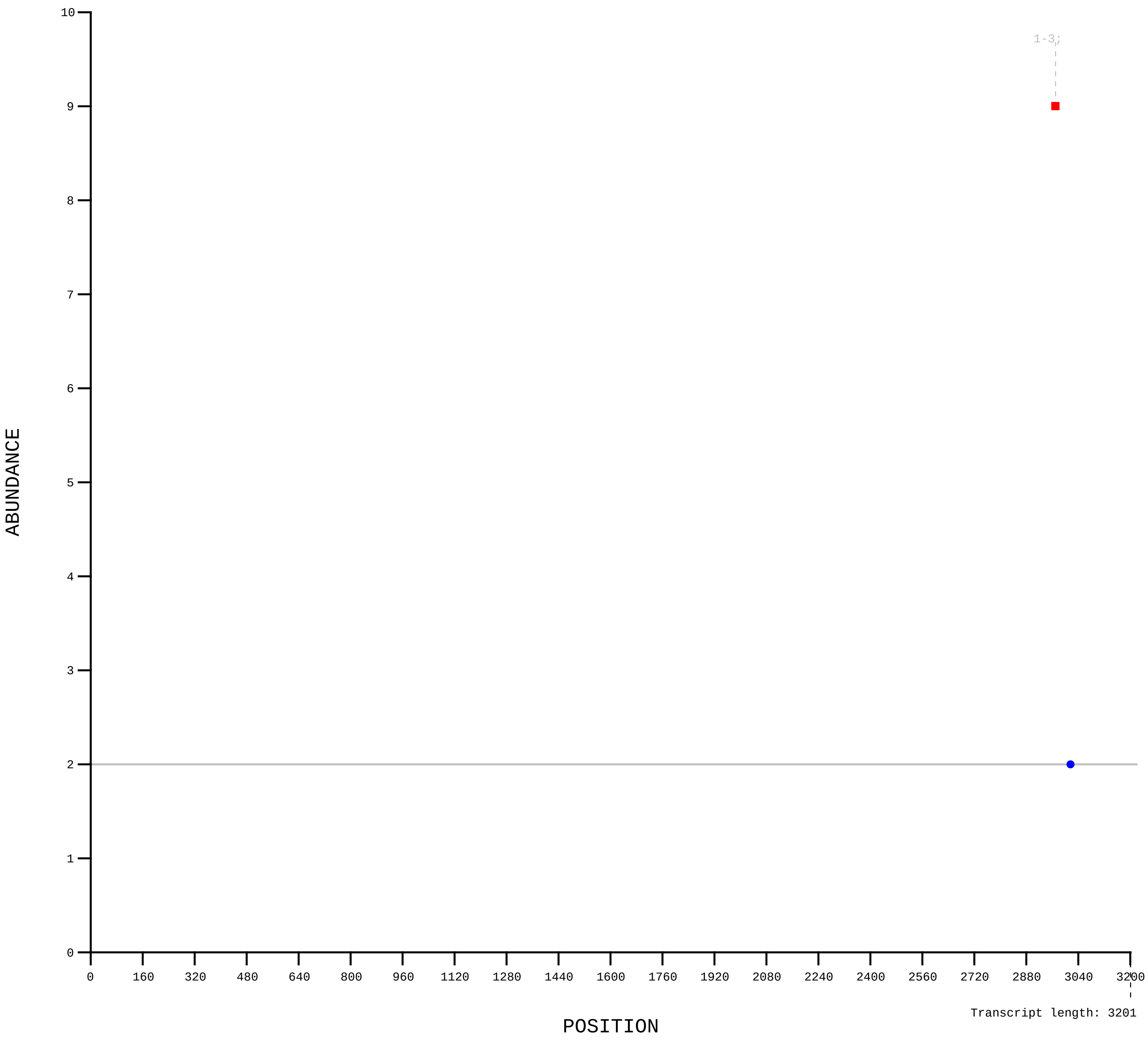


```
Category:  0  1  2  3  4
Degradome alignment:    Median:

0  #1  Position:2970  Abundance: 9.00(deg) 29(sRNA)
   5'    AAGAAT-AATGAGCTCTCCTTA        3'  ID:
         |||| | ||||||||||| ||             Score: 4.0
   3' CGTTTCTCAGTTACTCGAGAGAAACGAAGAAG 5'  p-value: 0.0

0  #2  Position:2970  Abundance: 9.00(deg) 6(sRNA)
   5'    AAGAAT-AATGAGCTCTCCTT         3'  ID:
         |||| | ||||||||||| ||             Score: 3.0
   3' CGTTTCTCAGTTACTCGAGAGAAACGAAGAAG 5'  p-value: 0.0

0  #3  Position:2970  Abundance: 9.00(deg) 5(sRNA)
   5'    AAGAAT-AATGAGCTCTCCT          3'  ID:
         |||| | ||||||||||| |              Score: 3.0
   3' CGTTTCTCAGTTACTCGAGAGAAACGAAGAAG 5'  p-value: 0.0
```

FOXG_12121T0 | Fusarium oxysporum f. sp. lycopersici 4287 hypothetical protein (978 nt)

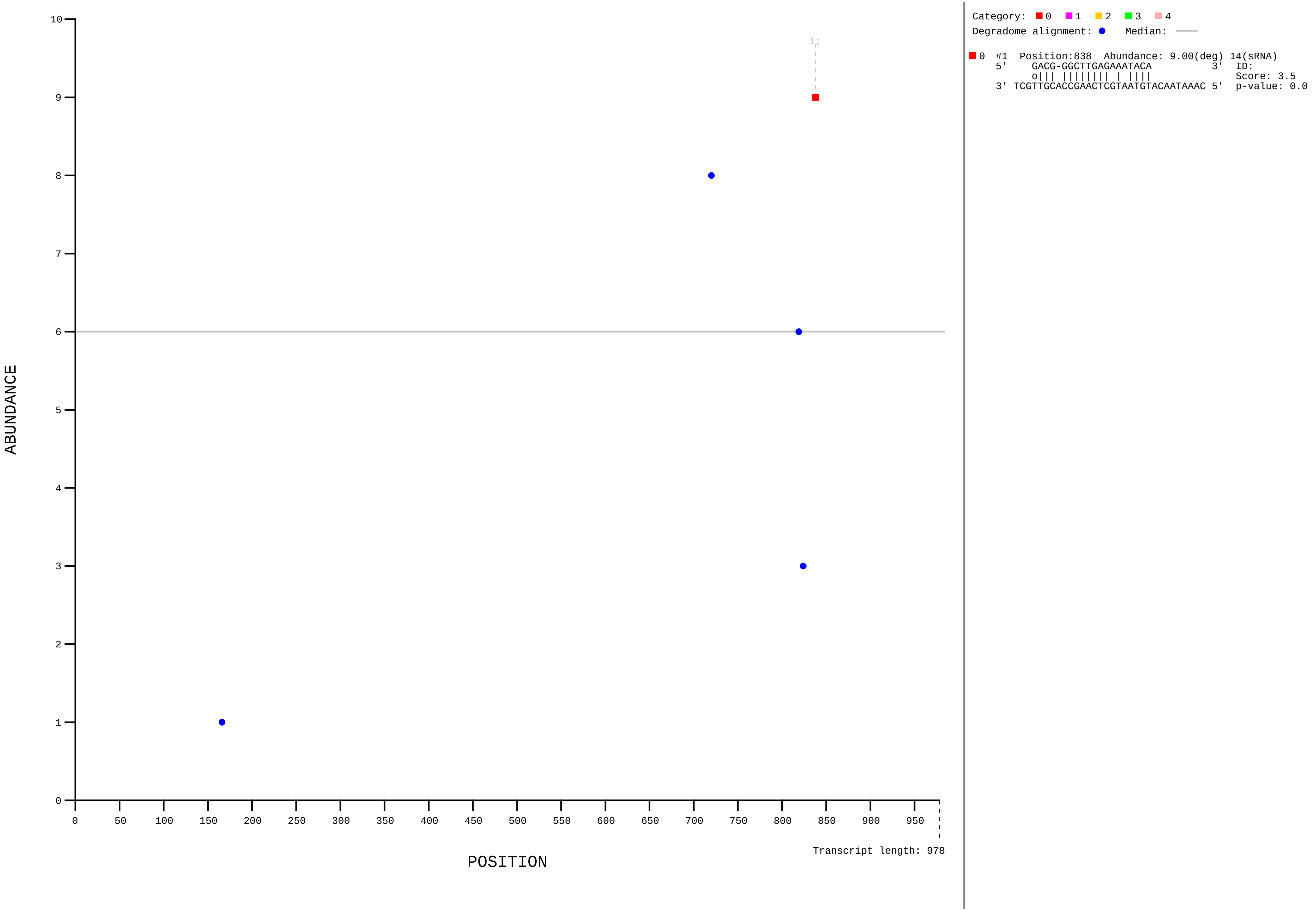

FOXG_13830T0 | Fusarium oxysporum f. sp. lycopersici 4287 small nucleolar ribonucleoprotein com (truncated)

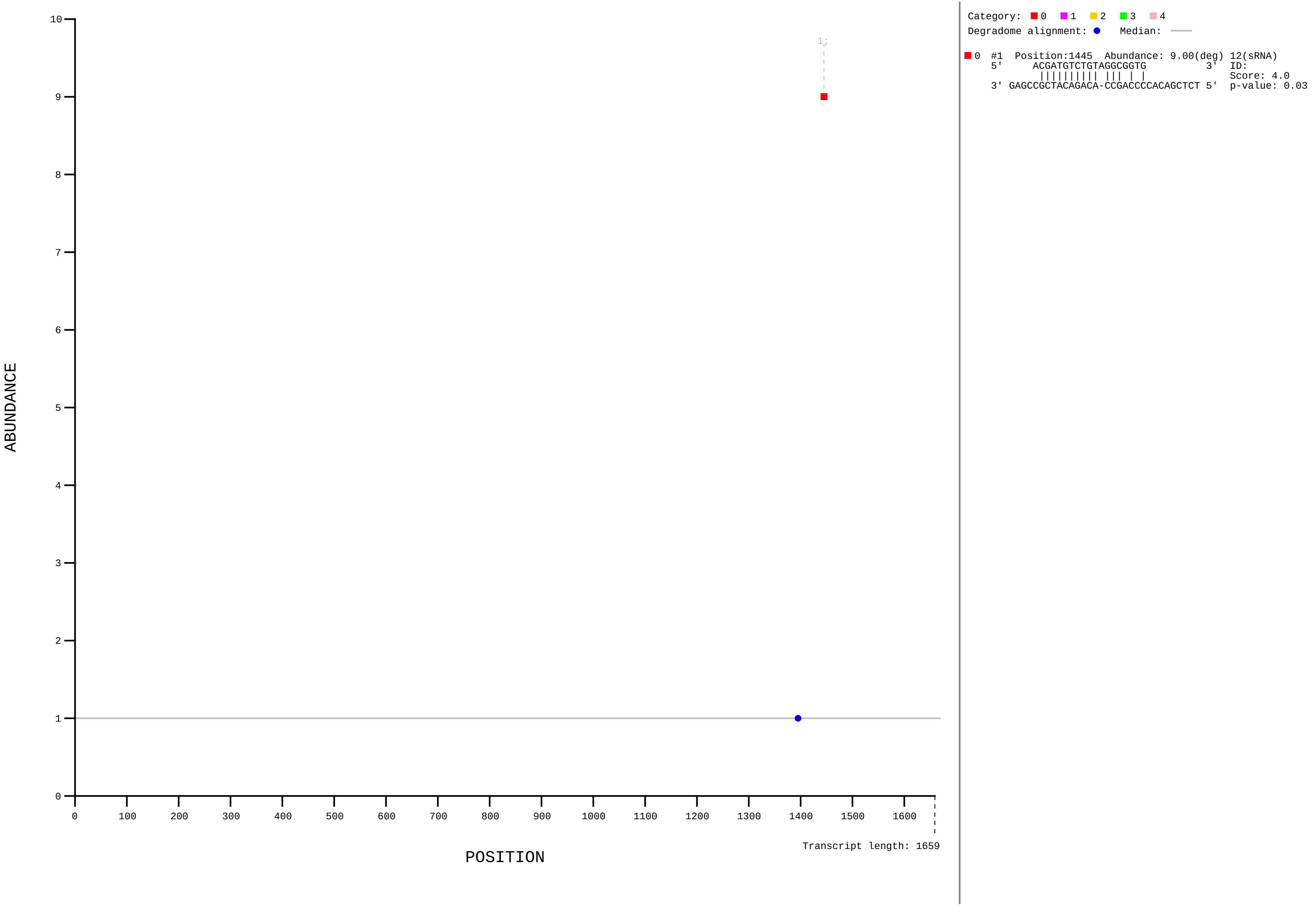

FOXG_08406T0 | Fusarium oxysporum f. sp. lycopersici 4287 hypothetical protein (2445 nt) Note(s): (truncated)

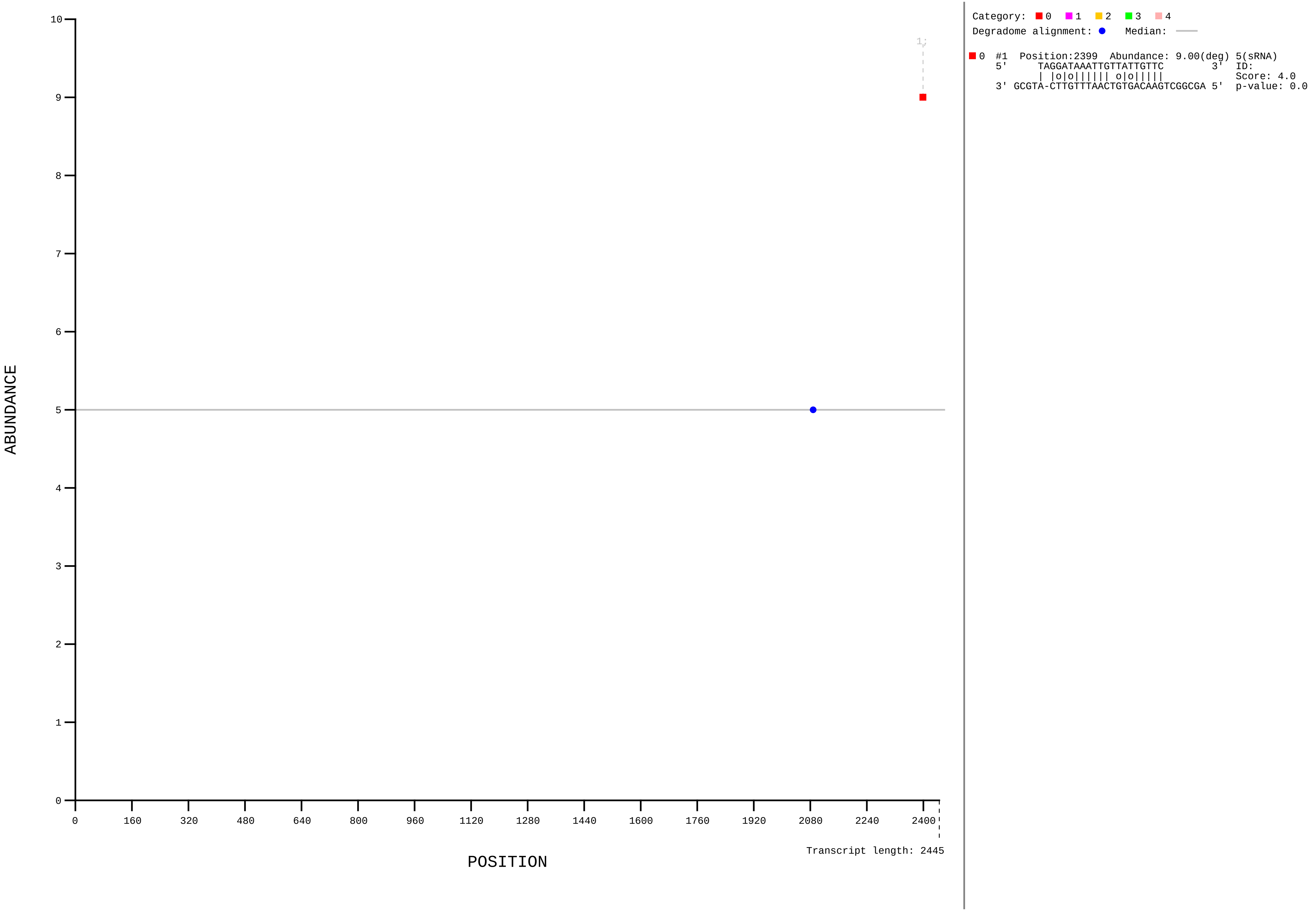

FOXG_13036T0 | Fusarium oxysporum f. sp. lycopersici 4287 hypothetical protein (660 nt)

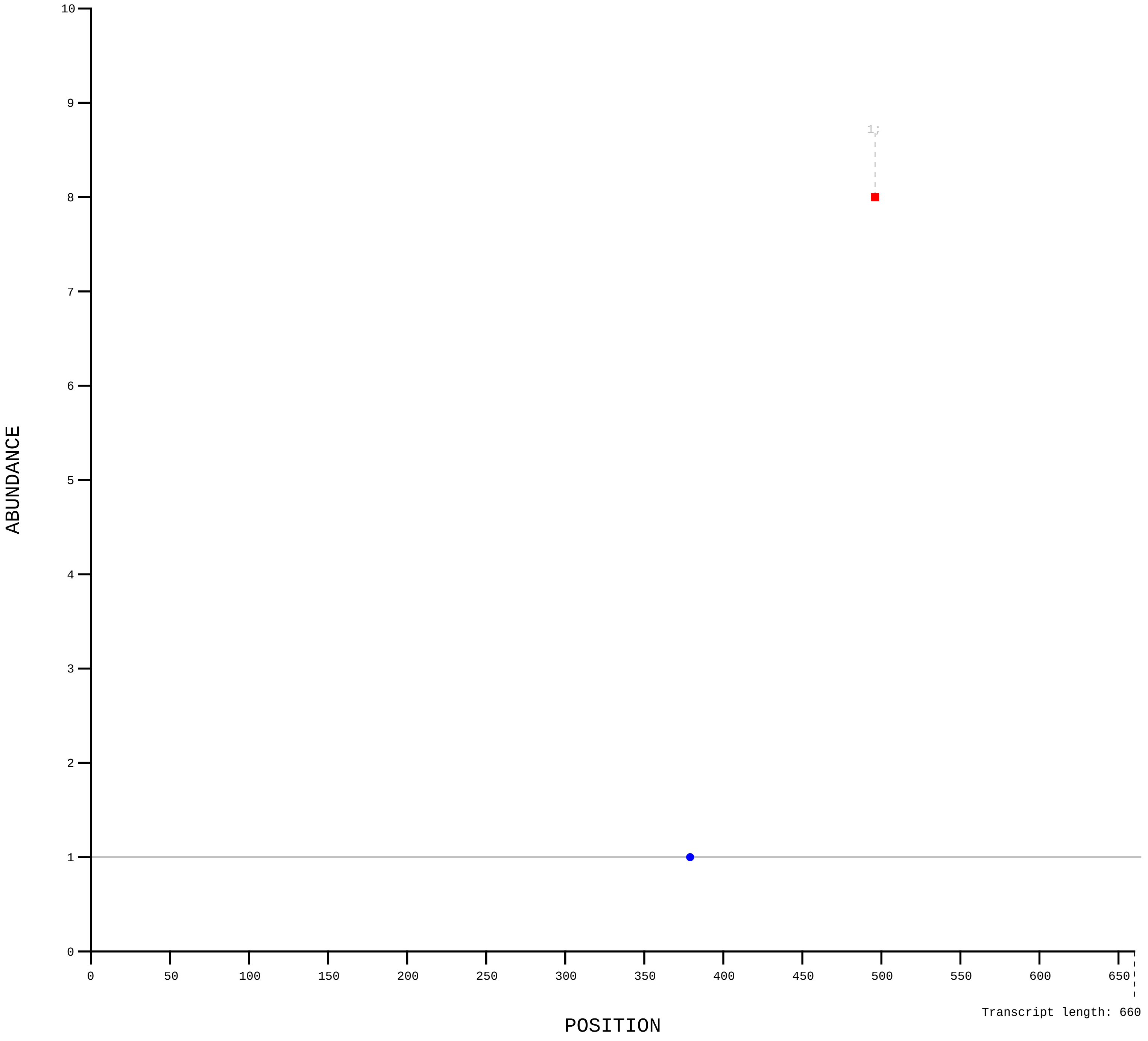

Category: 0 1 2 3 4
Degradome alignment: Median:

0 #1 Position:496 Abundance: 8.00(deg) 45(sRNA)

```
5'     CGATTCCCCTCGGTCGCAA          3'  ID:
       | |||||| ||||| ||||              Score: 3.0
3' AGAAGGTAAGGGAAGCCAACGTTCAGTACGTG 5'  p-value: 0.0
```

FOXG_00061T0 | Fusarium oxysporum f. sp. lycopersici 4287 hypothetical protein (717 nt)

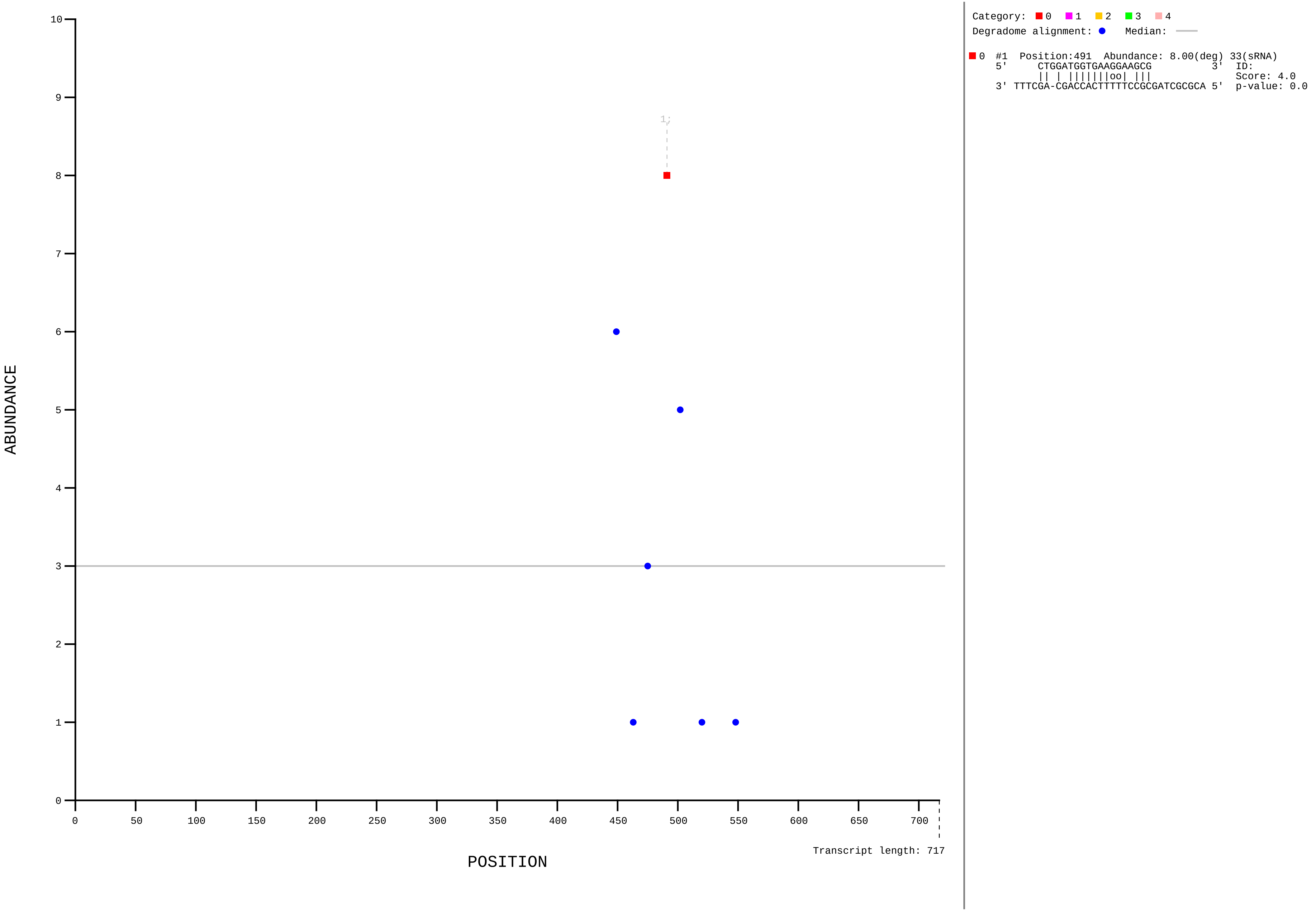

FOXG_05558T0 | Fusarium oxysporum f. sp. lycopersici 4287 oligosaccharyl transferase subunit gamma (990 nt)

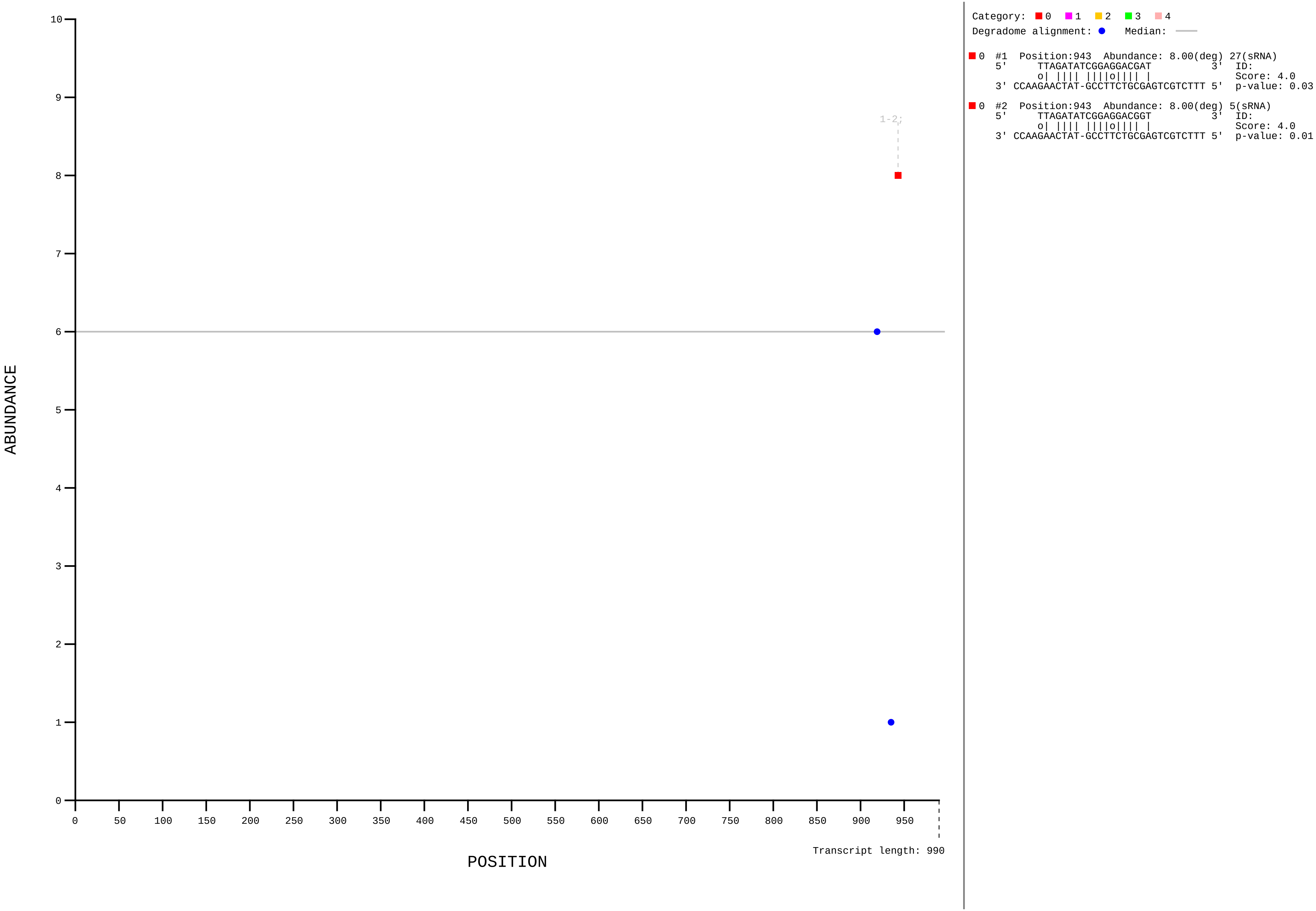

FOXG_07714T0 | Fusarium oxysporum f. sp. lycopersici 4287 hypothetical protein (429 nt)

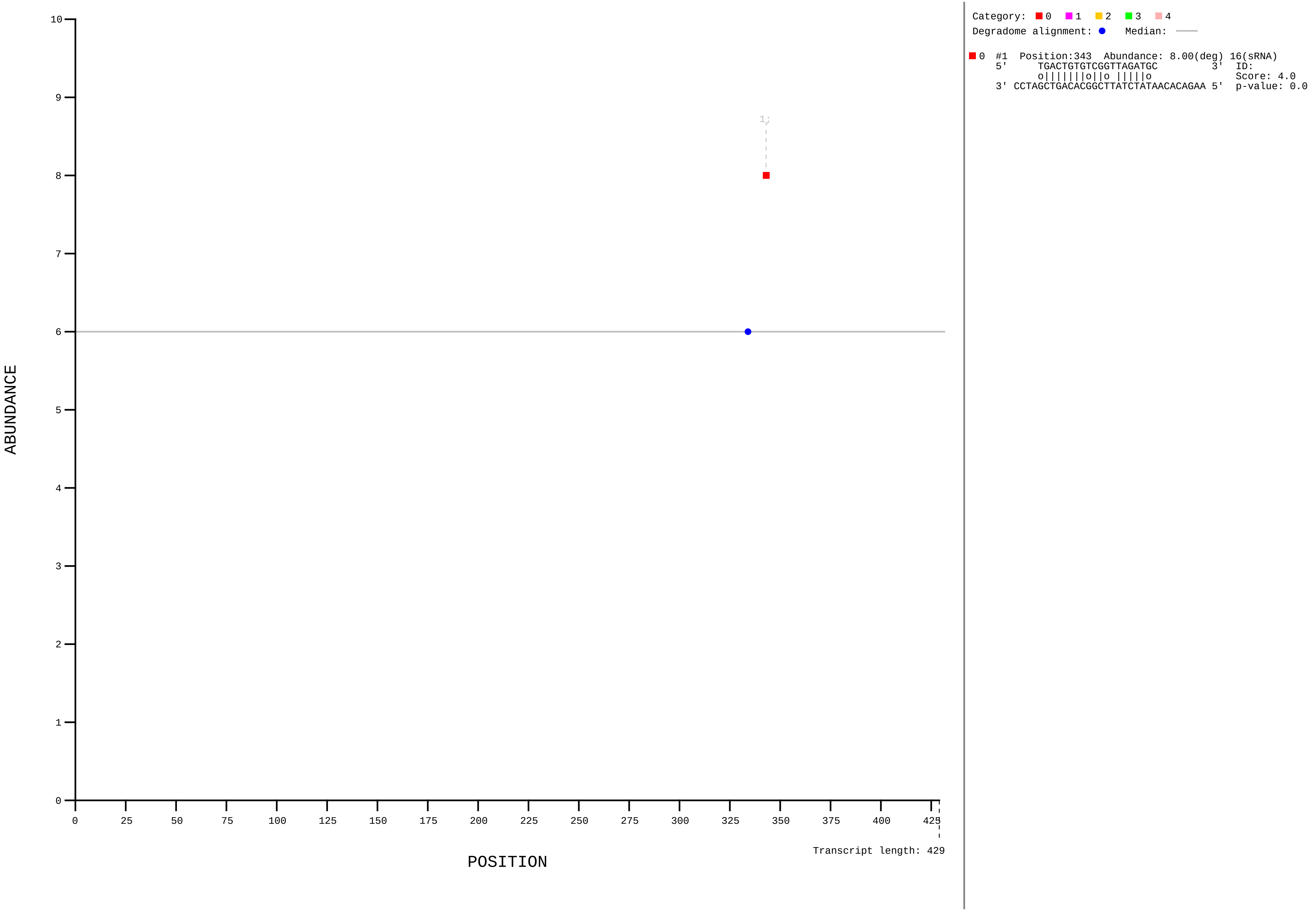

FOXG_05275T0 | Fusarium oxysporum f. sp. lycopersici 4287 hypothetical protein (960 nt)

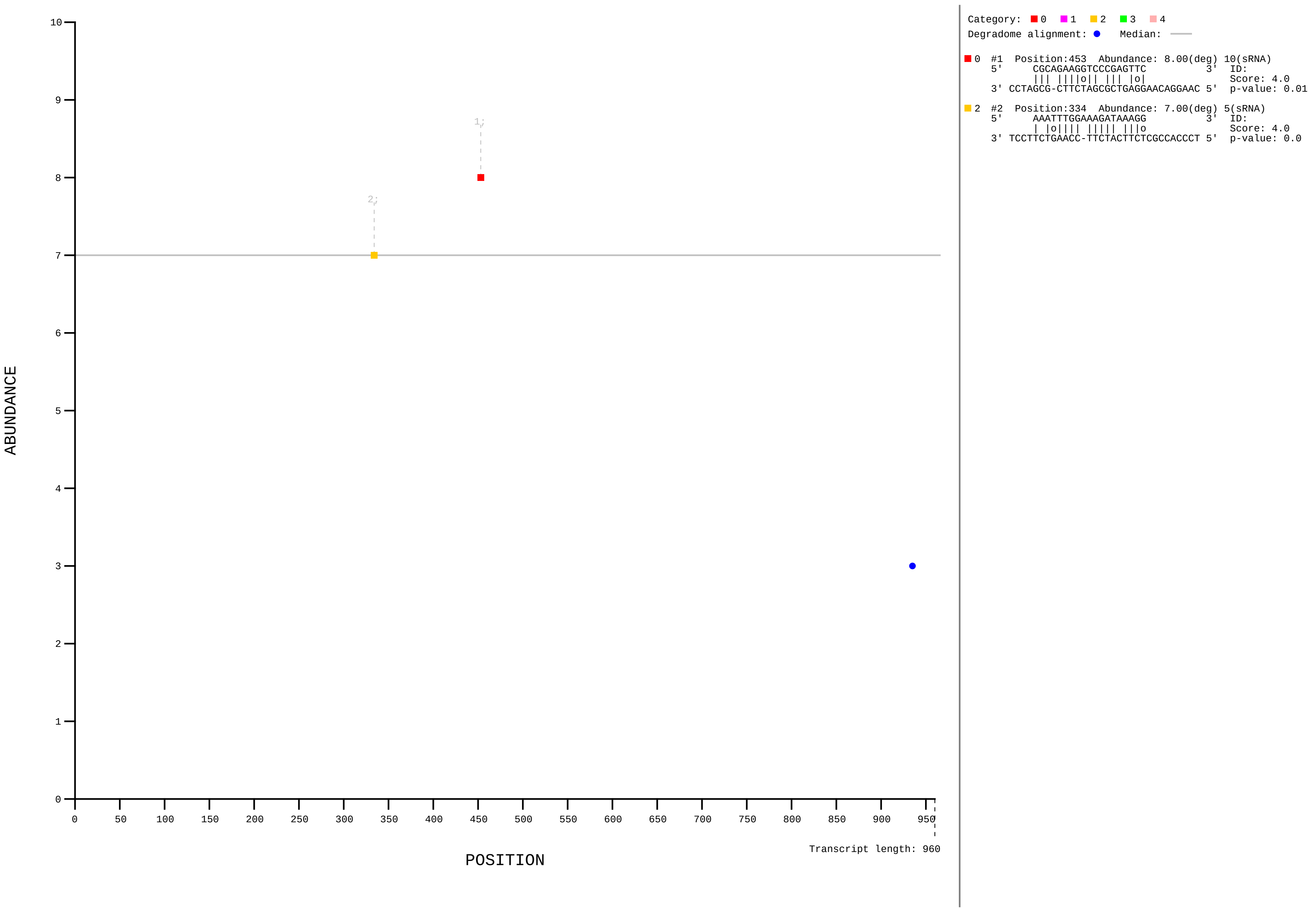

FOXG_00035T0 | Fusarium oxysporum f. sp. lycopersici 4287 enolase (1317 nt)

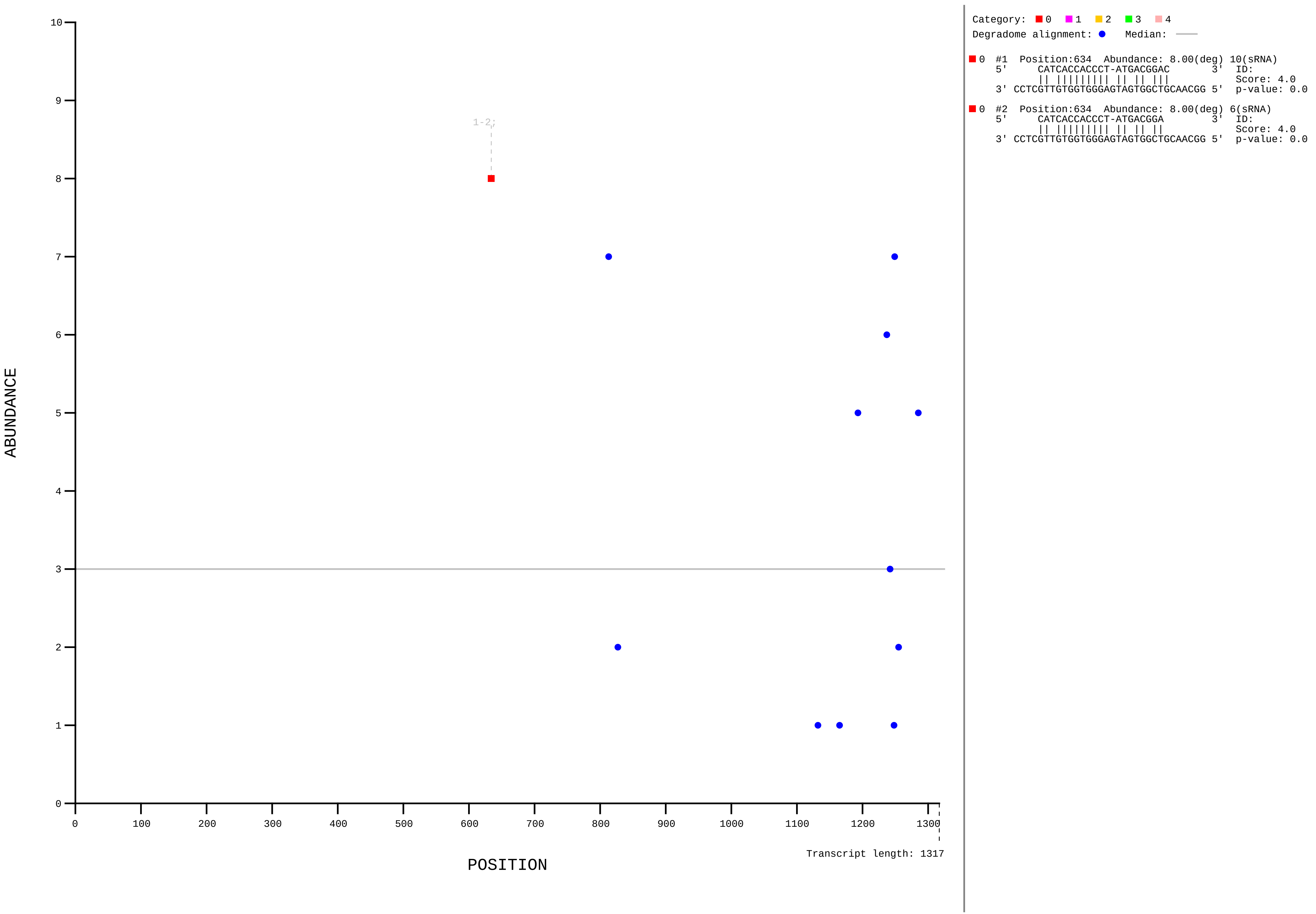

FOXG_11167T0 | Fusarium oxysporum f. sp. lycopersici 4287 hypothetical protein (1251 nt)

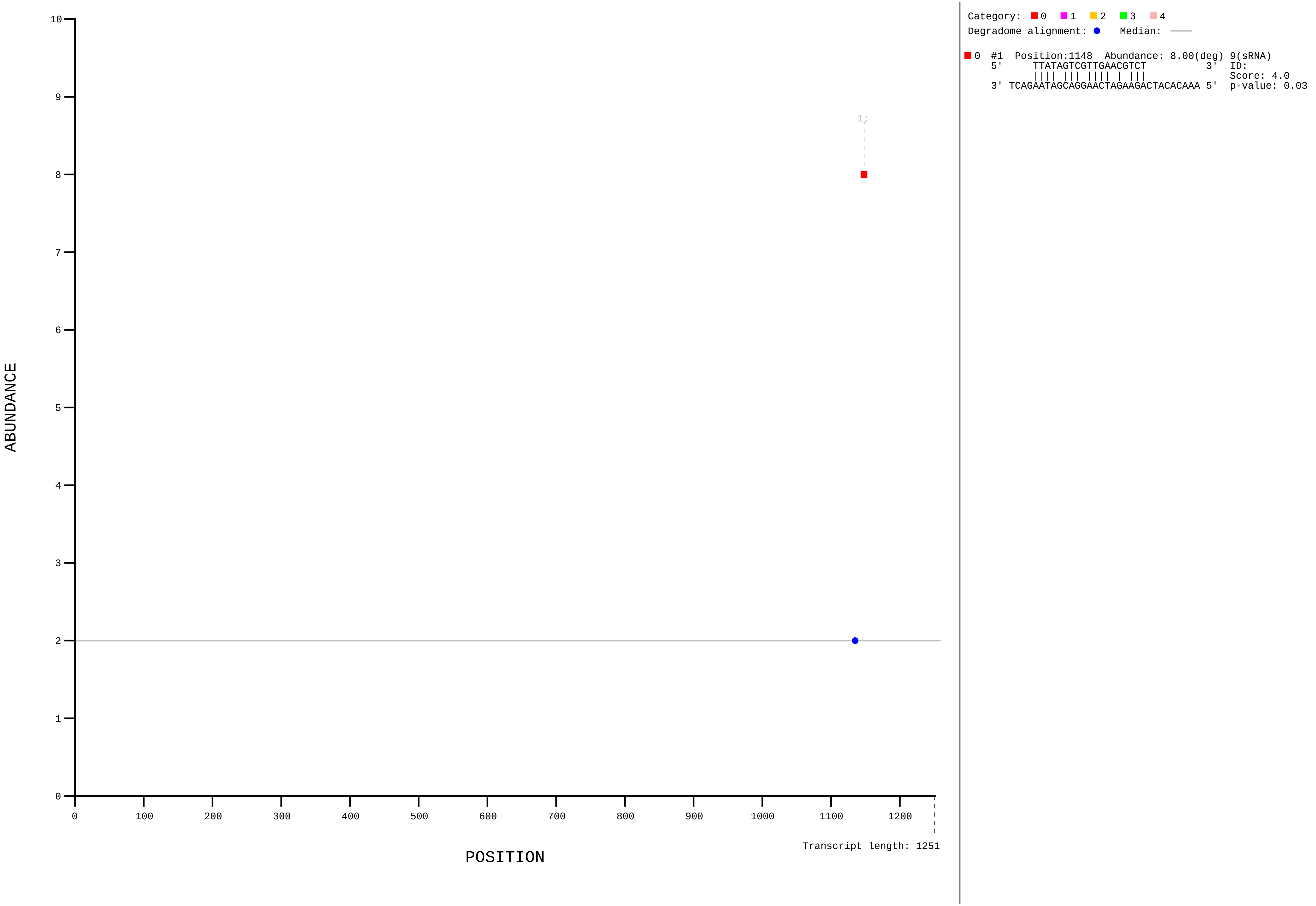

FOXG_00291T0 | Fusarium oxysporum f. sp. lycopersici 4287 bromodomain associated protein (699 nt)

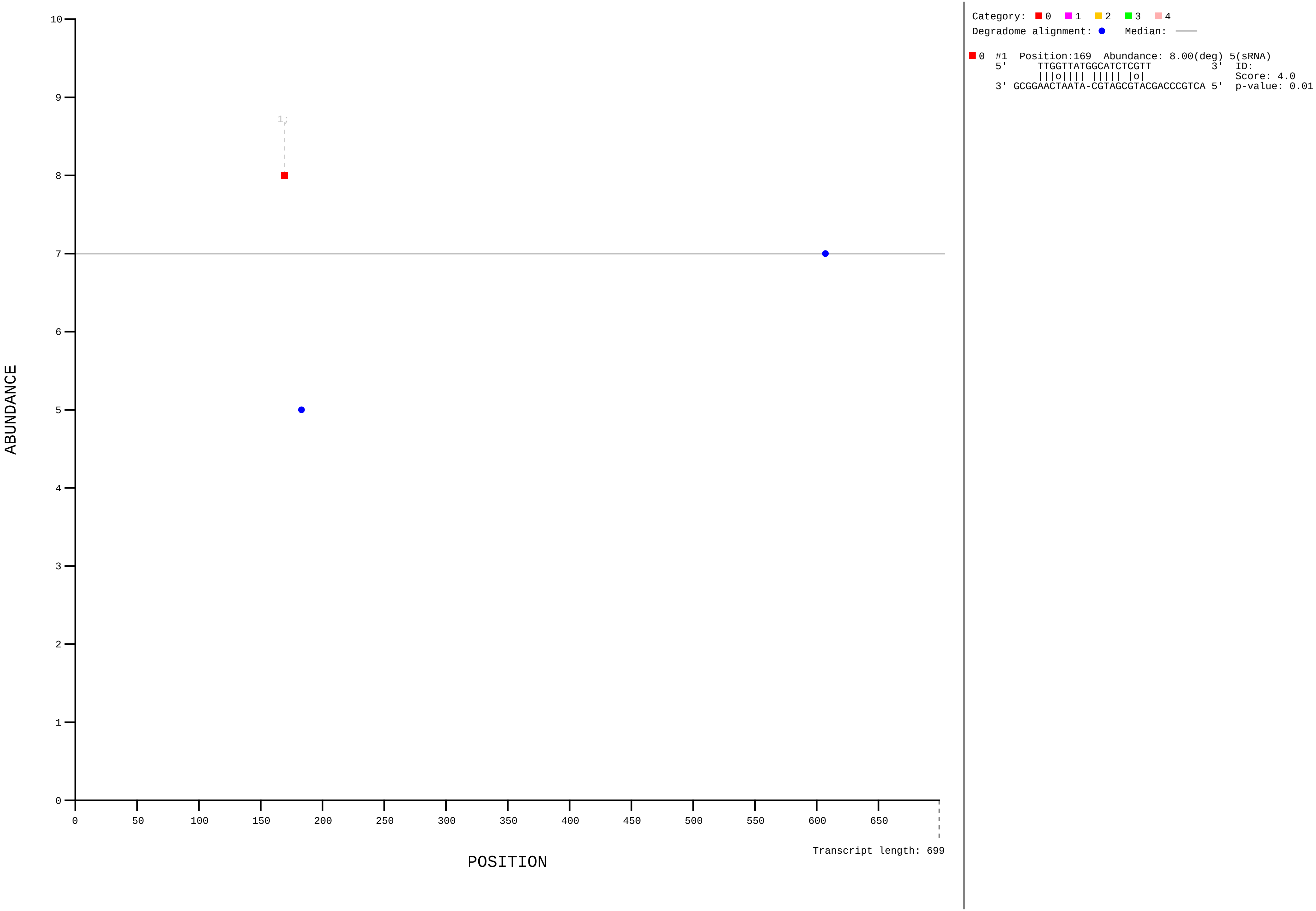

FOXG_07910T0 | Fusarium oxysporum f. sp. lycopersici 4287 inositolphosphorylceramide-B C-26 hydroxylase (1152 nt)

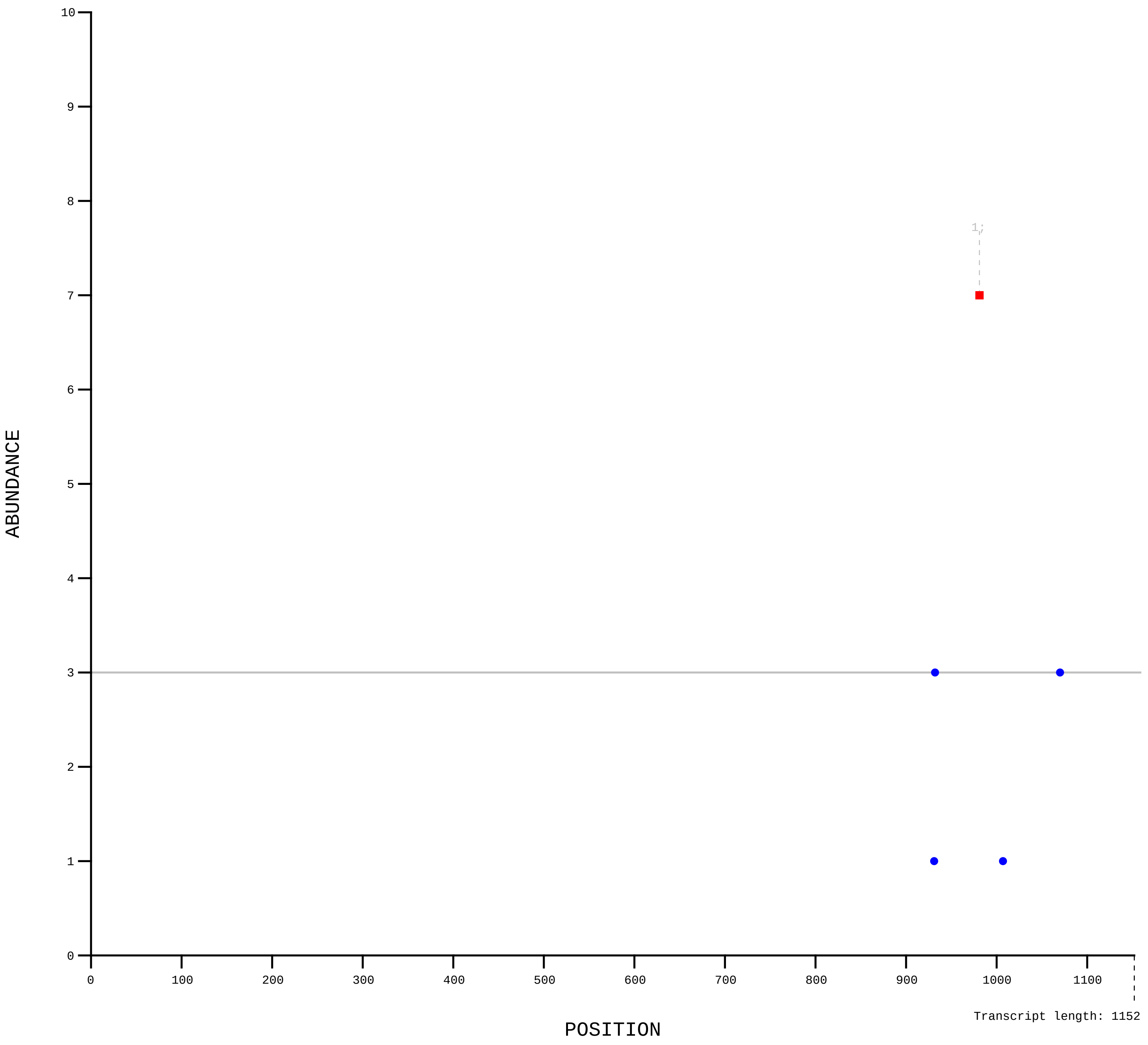


```
Category: ■0 ■1 ■2 ■3 ■4
Degradome alignment: ●   Median: ——

■0  #1  Position:981  Abundance: 7.00(deg) 23(sRNA)
    5'     TAATGTTGTGAAACTGTGG          3'  ID:
           ||||| ||||| | o||o|              Score: 4.0
    3' CTTCATTAC-ACACTCTAGCATCGTCTGCATA 5'  p-value: 0.01
```

FOXG_04260T0 | Fusarium oxysporum f. sp. lycopersici 4287 hypothetical protein (795 nt)

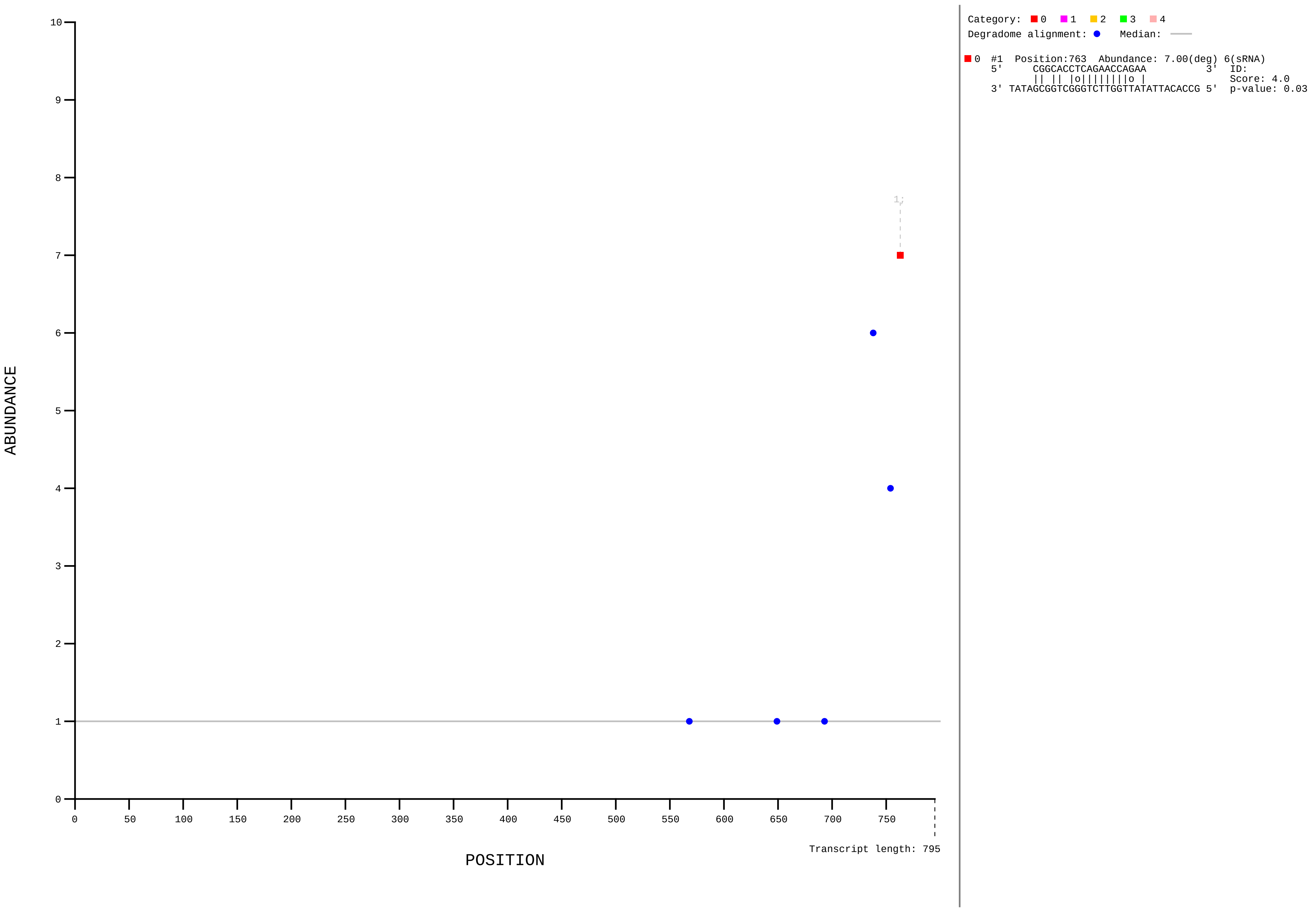

FOXG_05979T0 | Fusarium oxysporum f. sp. lycopersici 4287 hypothetical protein (1092 nt)

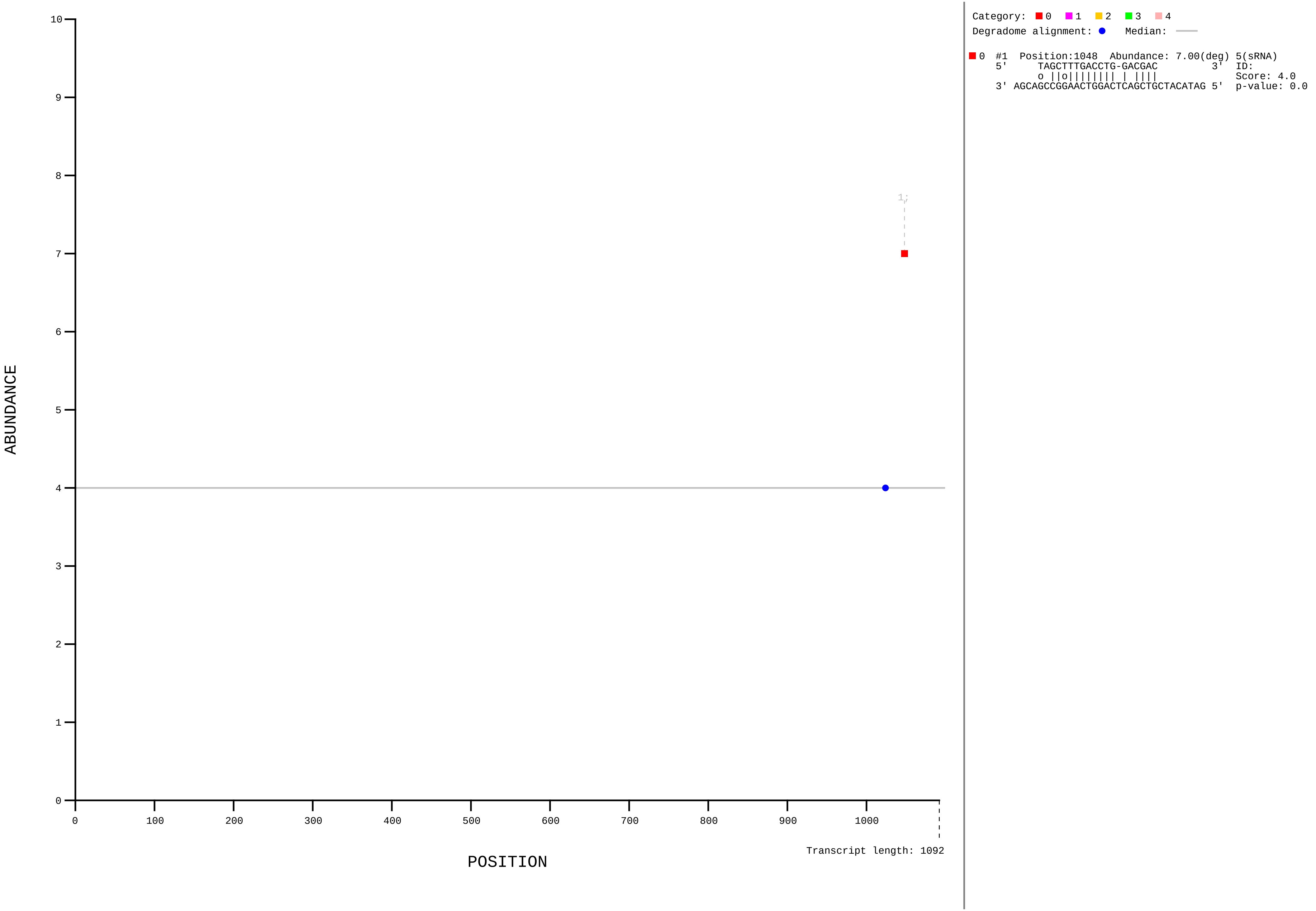

FOXG_01973T0 | Fusarium oxysporum f. sp. lycopersici 4287 hypothetical protein (564 nt)

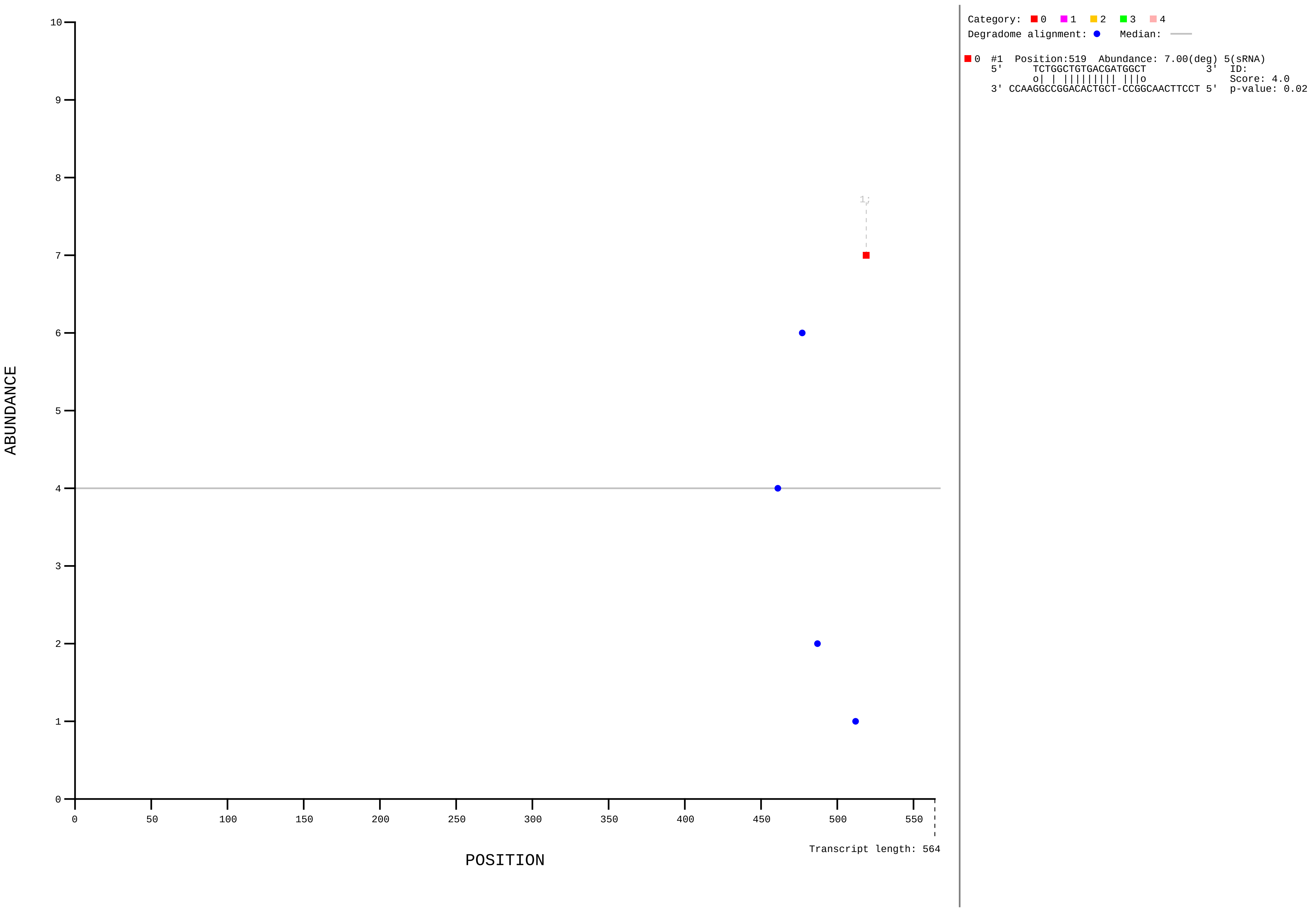

FOXG_09996T0 | Fusarium oxysporum f. sp. lycopersici 4287 hypothetical protein (1512 nt)

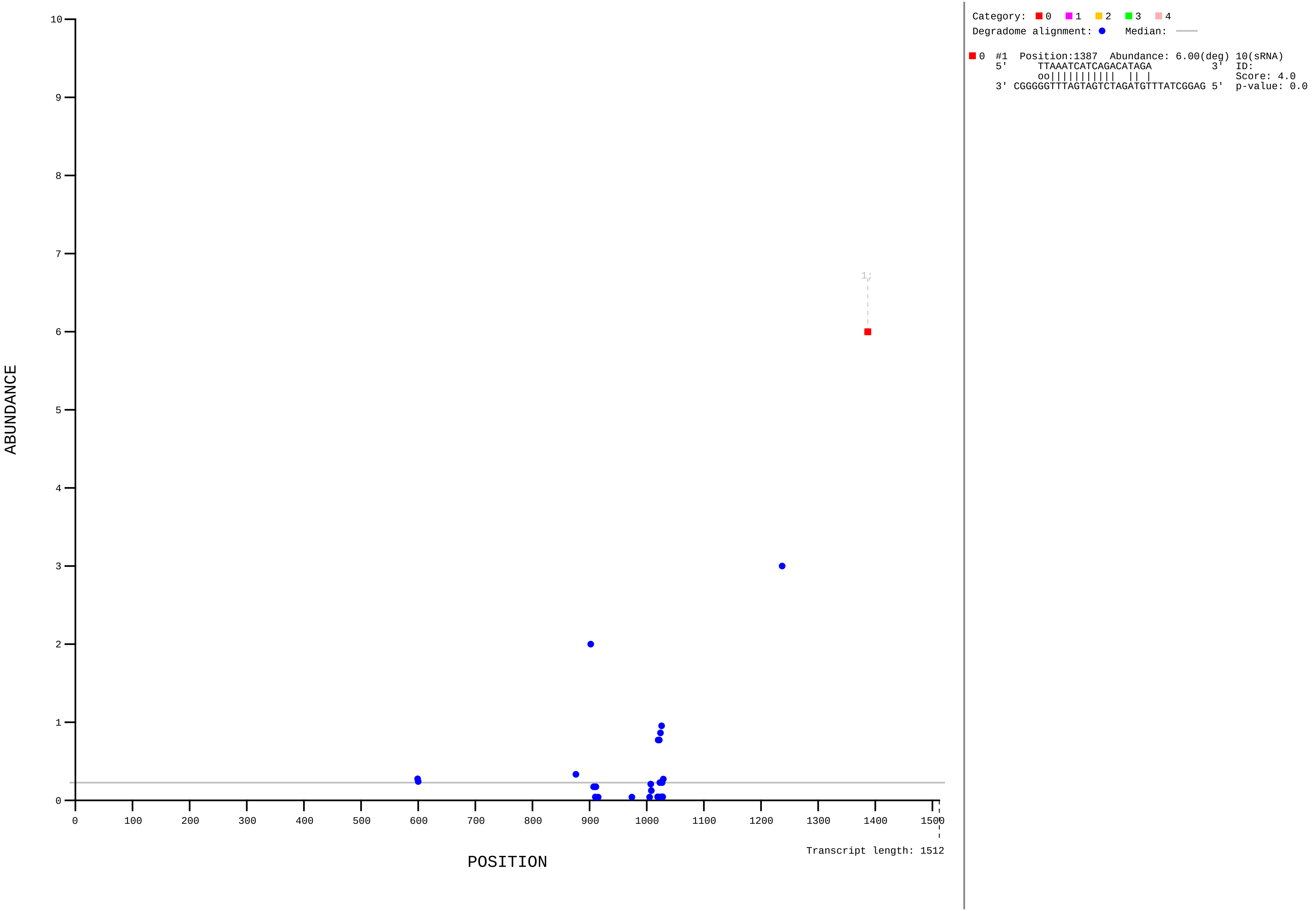

FOXG_07599T0 | Fusarium oxysporum f. sp. lycopersici 4287 hypothetical protein (378 nt)

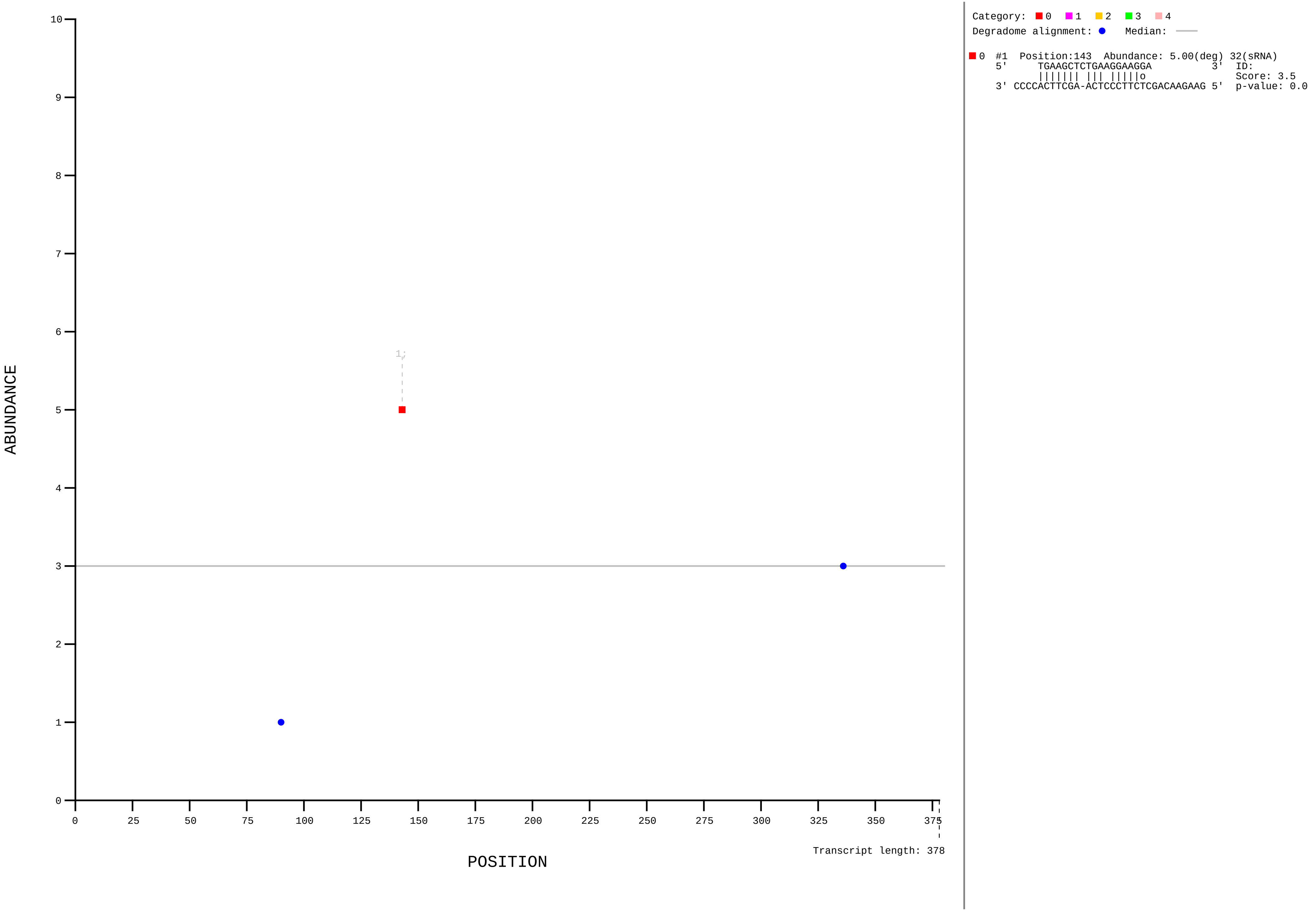

FOXG_17419T0 | Fusarium oxysporum f. sp. lycopersici 4287 hypothetical protein (1098 nt)

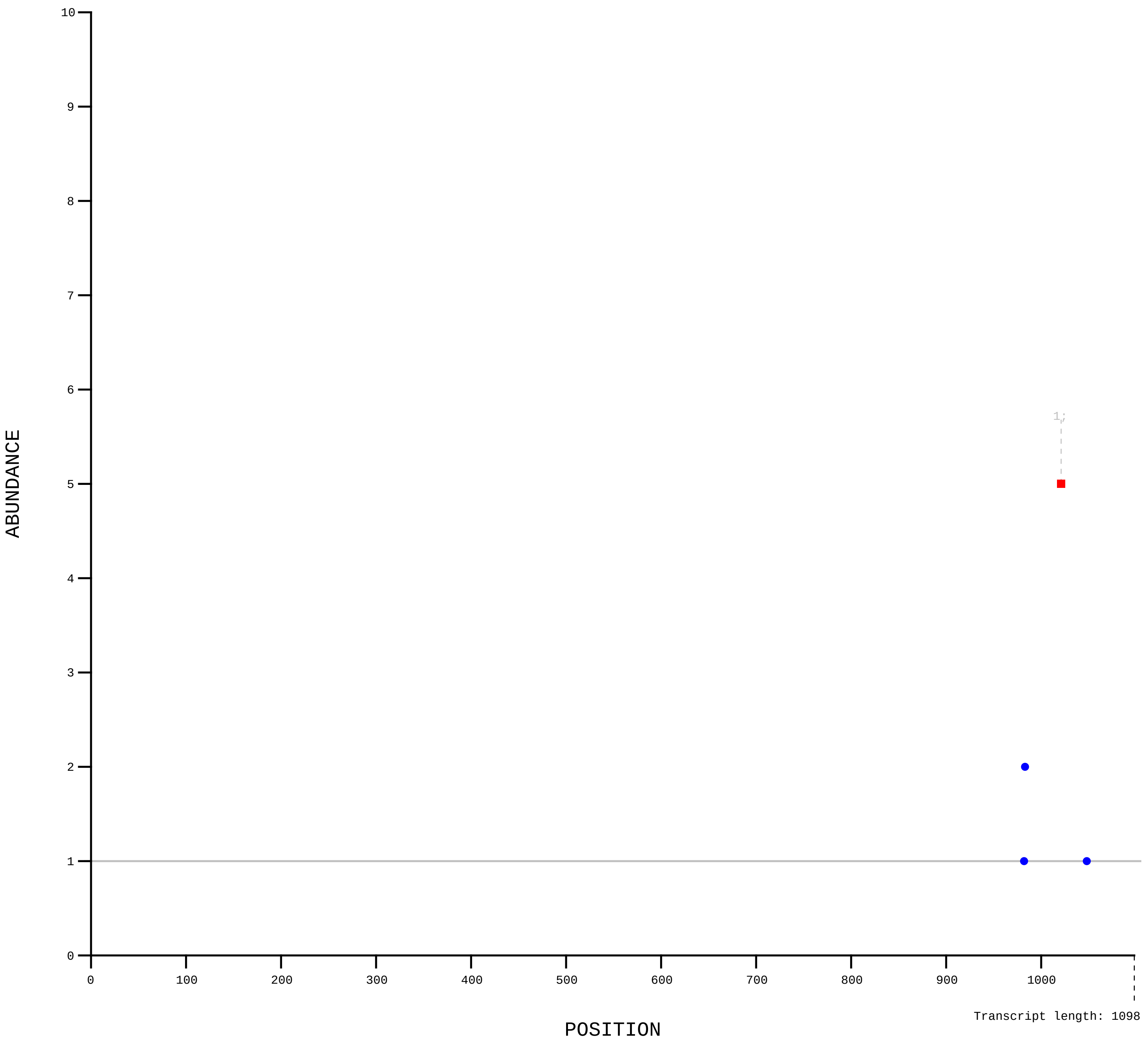

Category: 0 1 2 3 4
Degradome alignment: Median:

```
0  #1  Position:1021  Abundance: 5.00(deg) 10(sRNA)
   5'      TAGGATTAAGATTAGCCGC          3'  ID:
           ||||| ||||| |||  ||              Score: 4.0
   3' CGGTATCCT-ATTCTTATCATCGGTCCTCCTC 5'  p-value: 0.0
```

FOXG_00189T0 | Fusarium oxysporum f. sp. lycopersici 4287 20S proteasome subunit (786 nt)

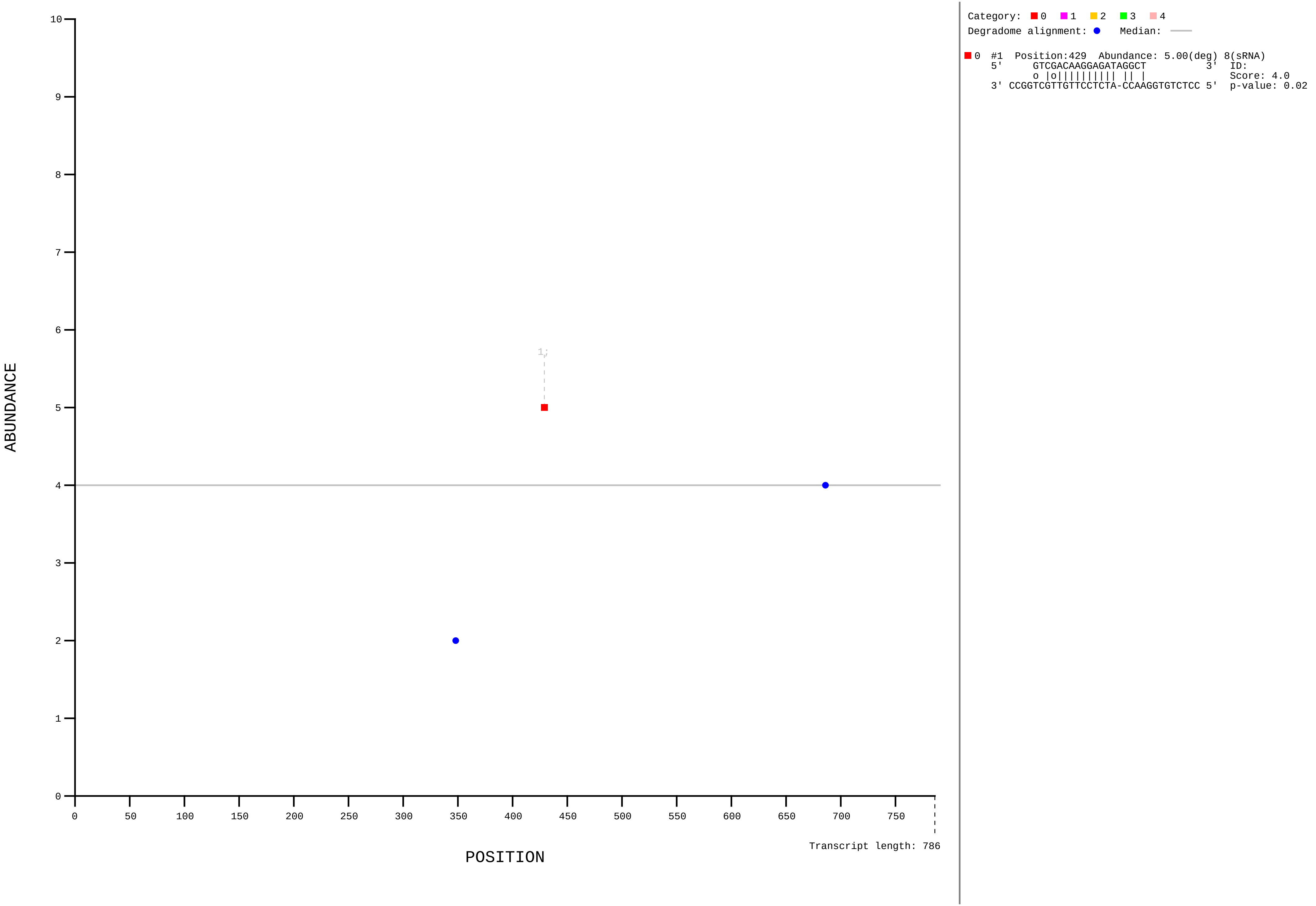

FOXG_07809T0 | Fusarium oxysporum f. sp. lycopersici 4287 hypothetical protein (759 nt)

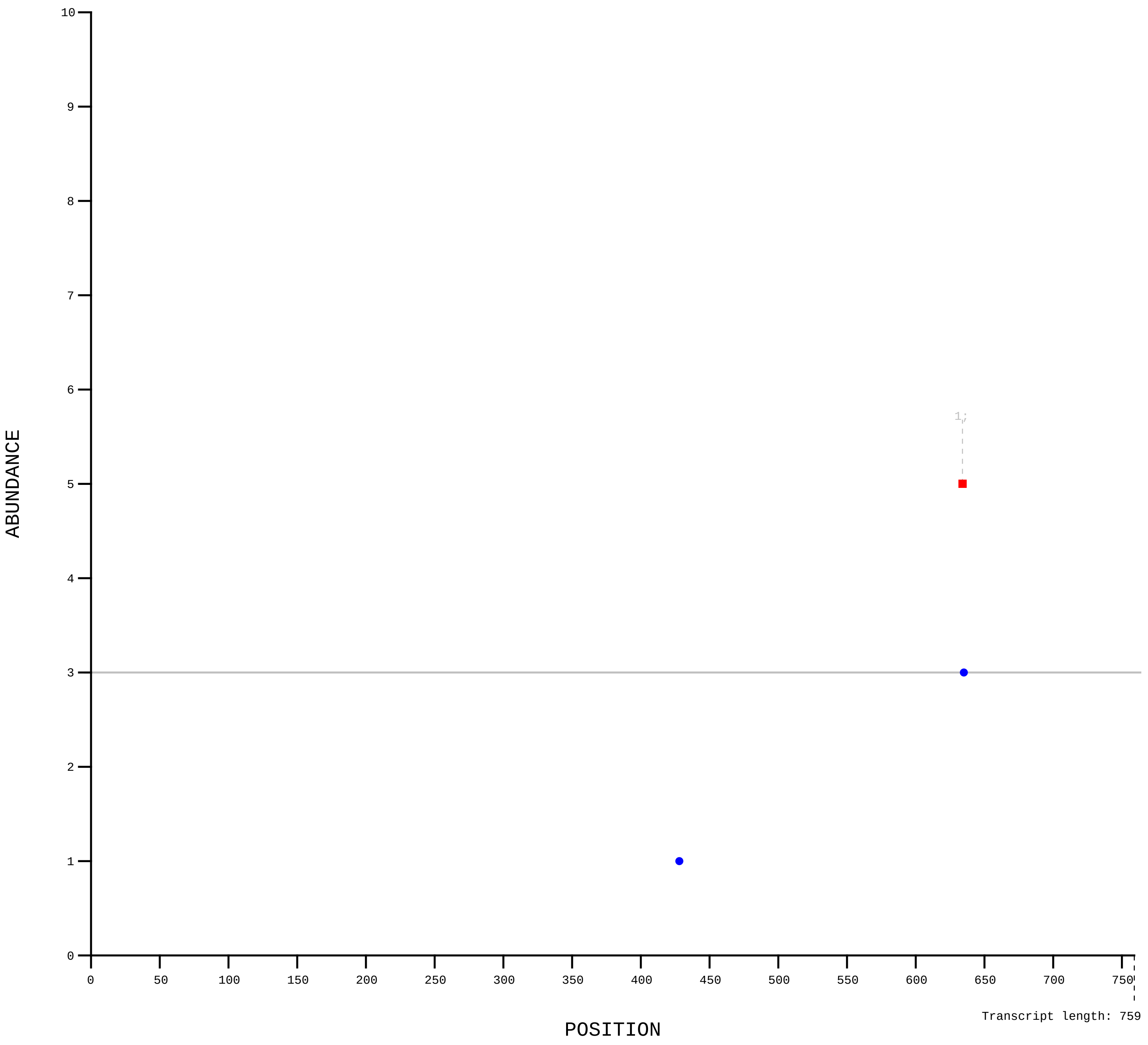

Category: 0 1 2 3 4
Degradome alignment: Median:

0 #1 Position:634 Abundance: 5.00(deg) 8(sRNA)

```
5'      CTTTATGATGA-CGTCGCGG         3'  ID:
        ||o| |||||| | ||o|||             Score: 4.0
3' GGAGGAGAAACTACTCGGAGTGCCACTGCGGA 5'  p-value: 0.02
```

FOXG_03524T0 | Fusarium oxysporum f. sp. lycopersici 4287 hypothetical protein (561 nt)

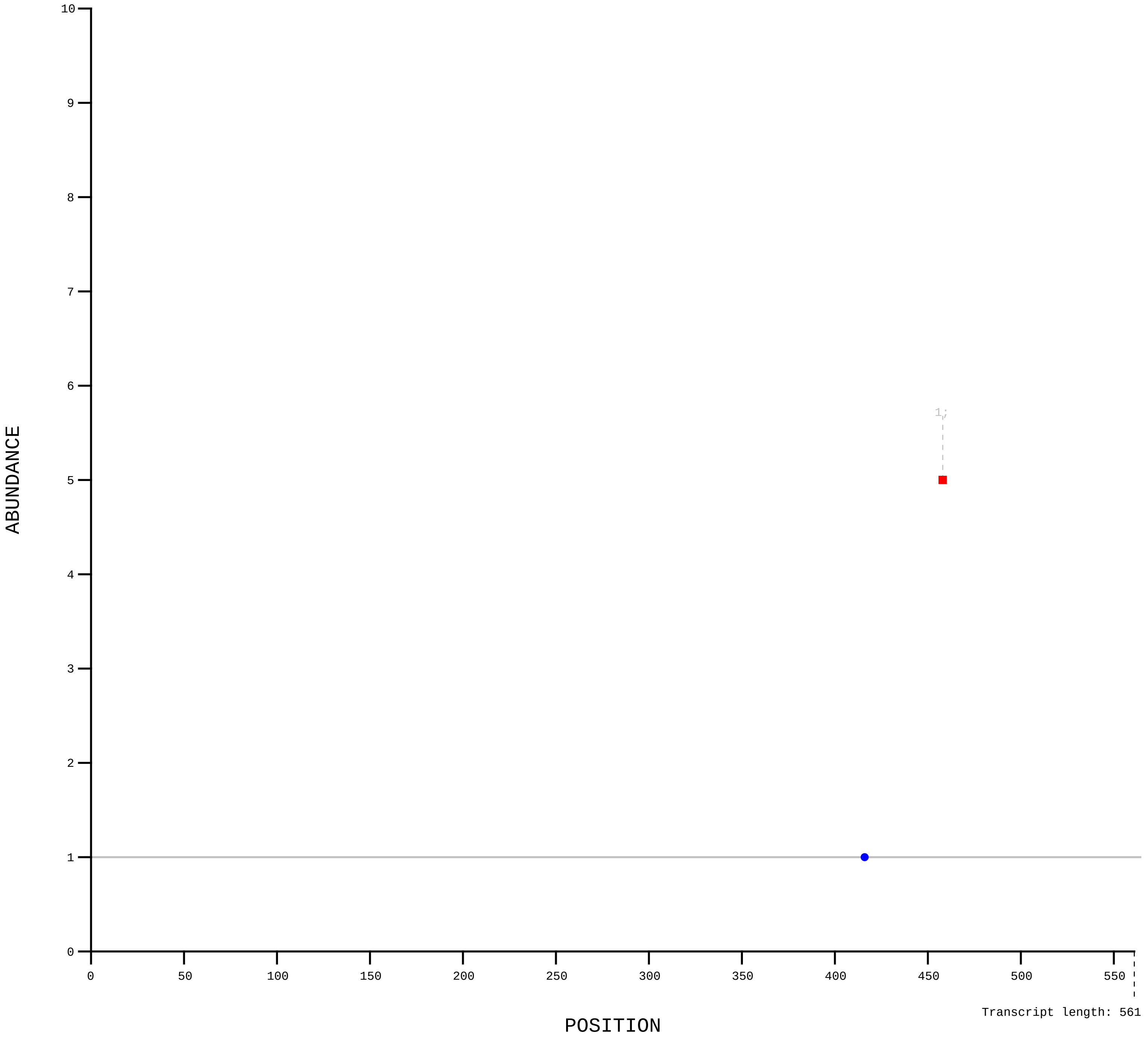

Category: 0 1 2 3 4
Degradome alignment: Median:

0 #1 Position:458 Abundance: 5.00(deg) 5(sRNA)

```
5'     GCTAAAGCTAGGATTAGAA          3'  ID:
       ||o||||| |||||o | |              Score: 4.0
3' TTCGCGGTTTCGCTCCTAGACGTCAAAAGAGG 5'  p-value: 0.0
```

FOXG_06092T0 | Fusarium oxysporum f. sp. lycopersici 4287 hypothetical protein (612 nt)

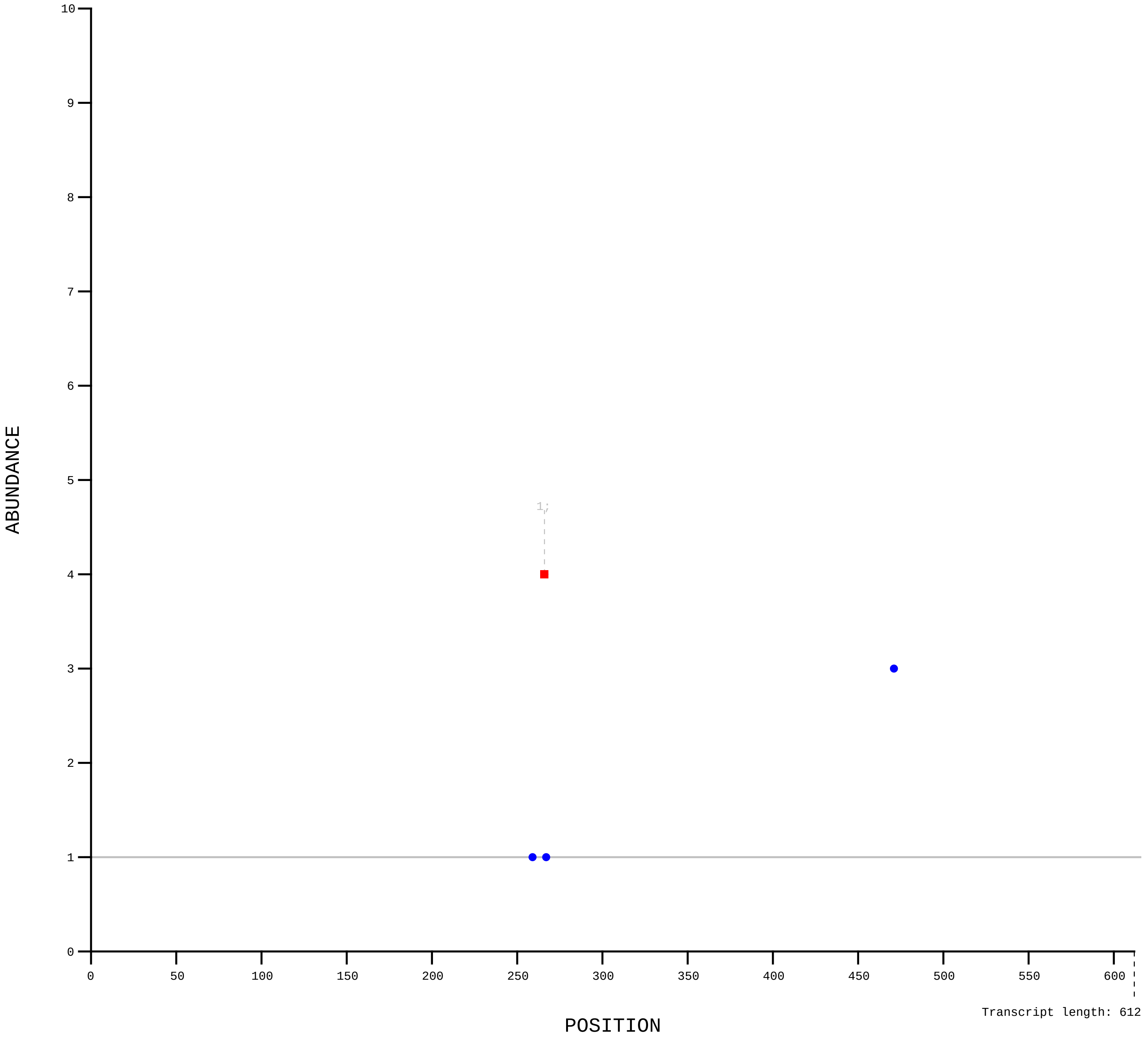

Category: 0 1 2 3 4
Degradome alignment: ● Median: —

```
■0  #1  Position:266  Abundance: 4.00(deg) 8(sRNA)
    5'     TTGAGTGGGAGGGTTAGAGA         3'  ID:
           | ||o||| |||||o|| ||             Score: 4.0
    3' ATGCATCTTACCATCCCAGTCCCTCATAAGTC 5'  p-value: 0.04
```

FOXG_03365T0 | Fusarium oxysporum f. sp. lycopersici 4287 hypothetical protein (594 nt)

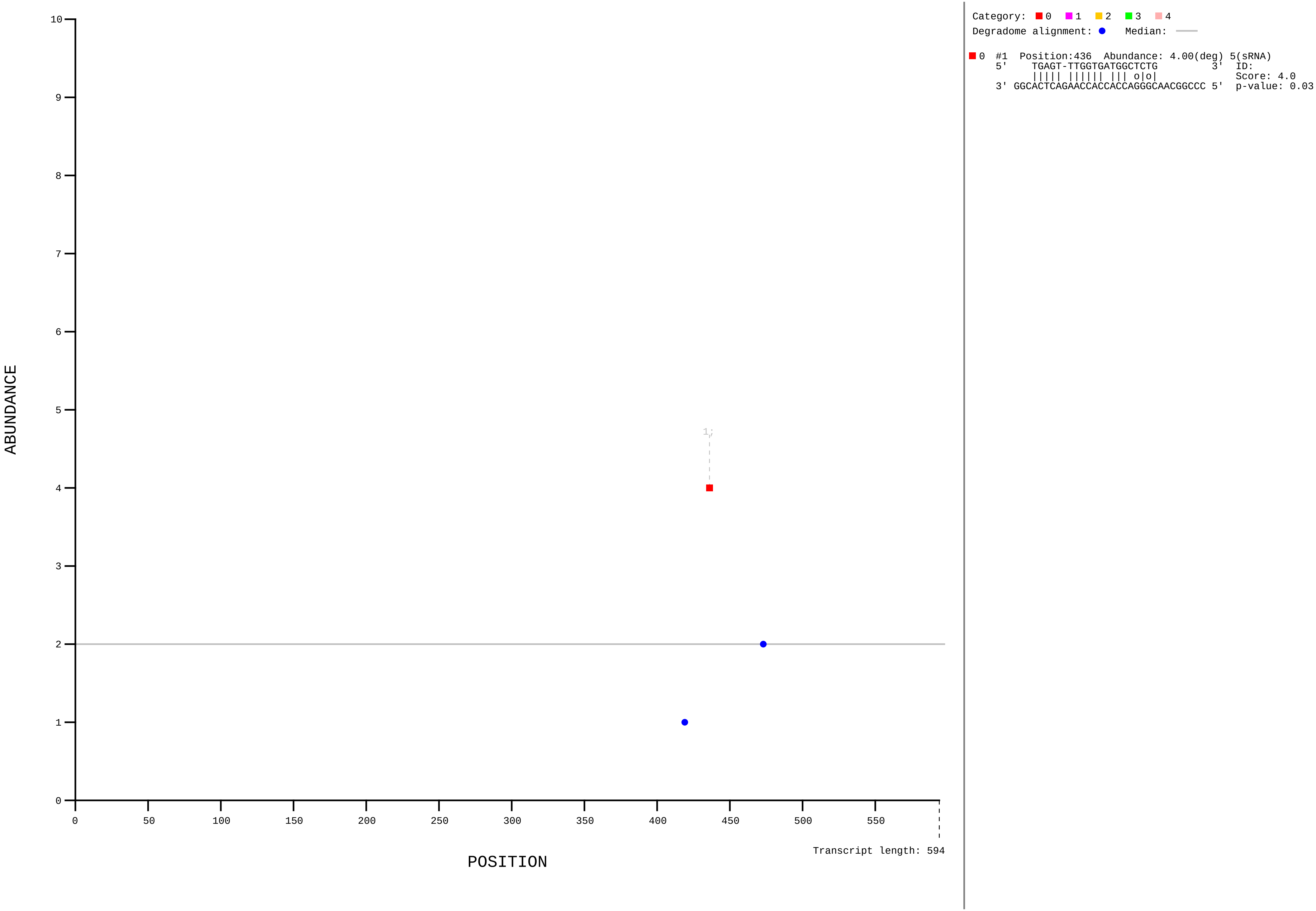

FOXG_15827T0 | Fusarium oxysporum f. sp. lycopersici 4287 hypothetical protein (999 nt)

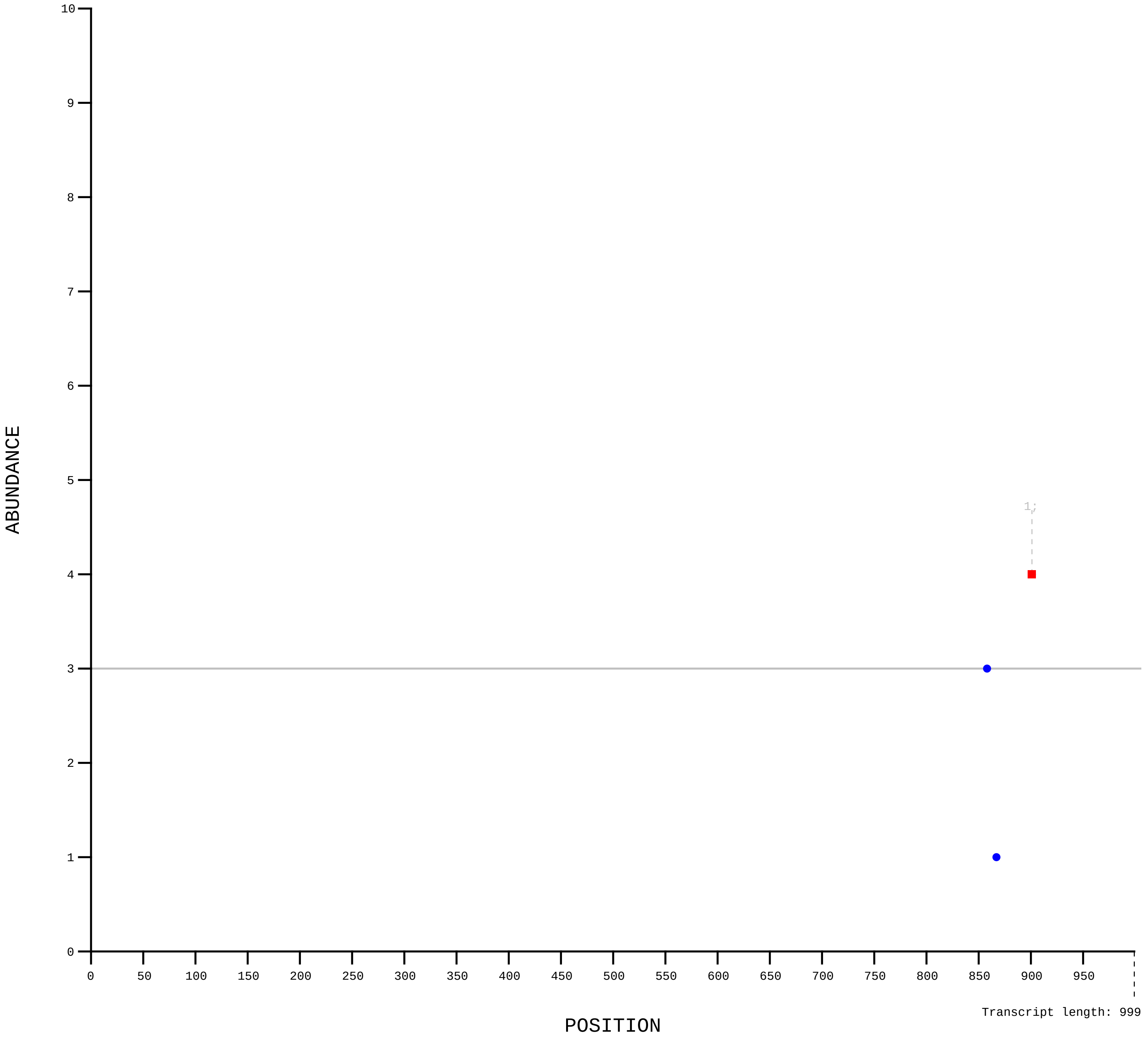

Category: 0 1 2 3 4
Degradome alignment: Median:

```
0  #1  Position:901  Abundance: 4.00(deg) 5(sRNA)
   5'     TGAGAATAGTGAACTTATT          3'  ID:
          |||o||o||||| |o| ||              Score: 3.5
   3' CGCTACTTTTGTCACT-GGAGAAGGAAGCTGG 5'  p-value: 0.01
```

FOXG_04985T0 | Fusarium oxysporum f. sp. lycopersici 4287 golgi integral membrane protein Cln3 (1371 nt) (truncated)

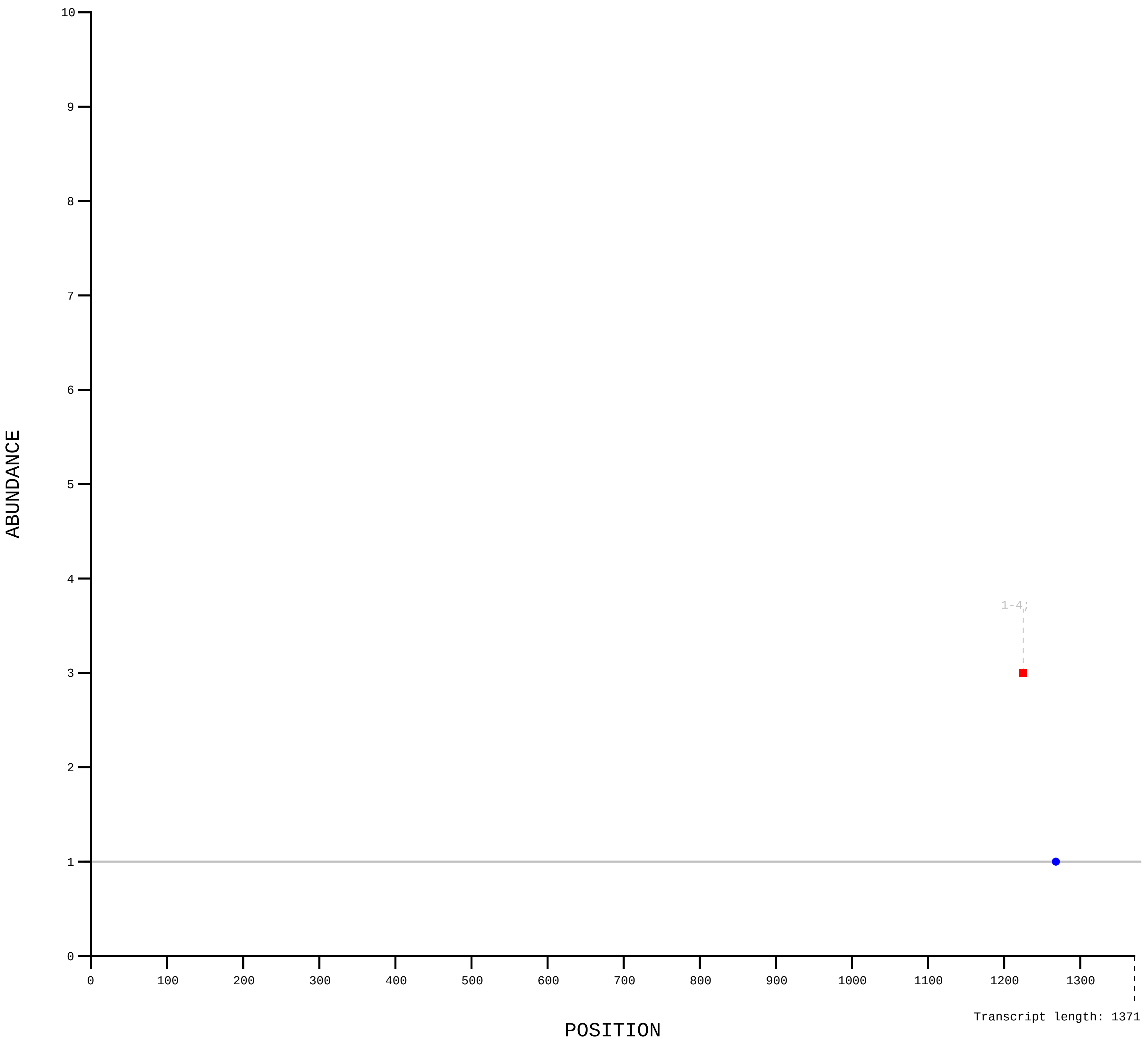


```
Category: 0  1  2  3  4
Degradome alignment:    Median:

0  #1  Position:1225  Abundance: 3.00(deg) 49(sRNA)
   5'     CAGGACTGAATGCTTTATCGT        3'  ID:
          ||o||||o||| ||o|||| |            Score: 3.5
   3' CGAGGTTCTGATTTAAGAGATAGGAGCCCACC 5'  p-value: 0.0

0  #2  Position:1225  Abundance: 3.00(deg) 32(sRNA)
   5'     TGAGATTAAATTCTGTACC          3'  ID:
          oo|||o|||||||| || |              Score: 3.5
   3' CGAGGTTCTGATTTAAGAGATAGGAGCCCACC 5'  p-value: 0.01

0  #3  Position:1225  Abundance: 3.00(deg) 22(sRNA)
   5'     CAGGACTGAATGCTTTATC          3'  ID:
          ||o||||o||| ||o||||              Score: 2.5
   3' CGAGGTTCTGATTTAAGAGATAGGAGCCCACC 5'  p-value: 0.0

0  #4  Position:1225  Abundance: 3.00(deg) 5(sRNA)
   5'     CAGGACTGAATGCTTTATCG         3'  ID:
          ||o||||o||| ||o||||              Score: 3.5
   3' CGAGGTTCTGATTTAAGAGATAGGAGCCCACC 5'  p-value: 0.0
```

FOXG_10289T0 | Fusarium oxysporum f. sp. lycopersici 4287 vacuolar protein sorting-associated protein 35 (2619 nt)

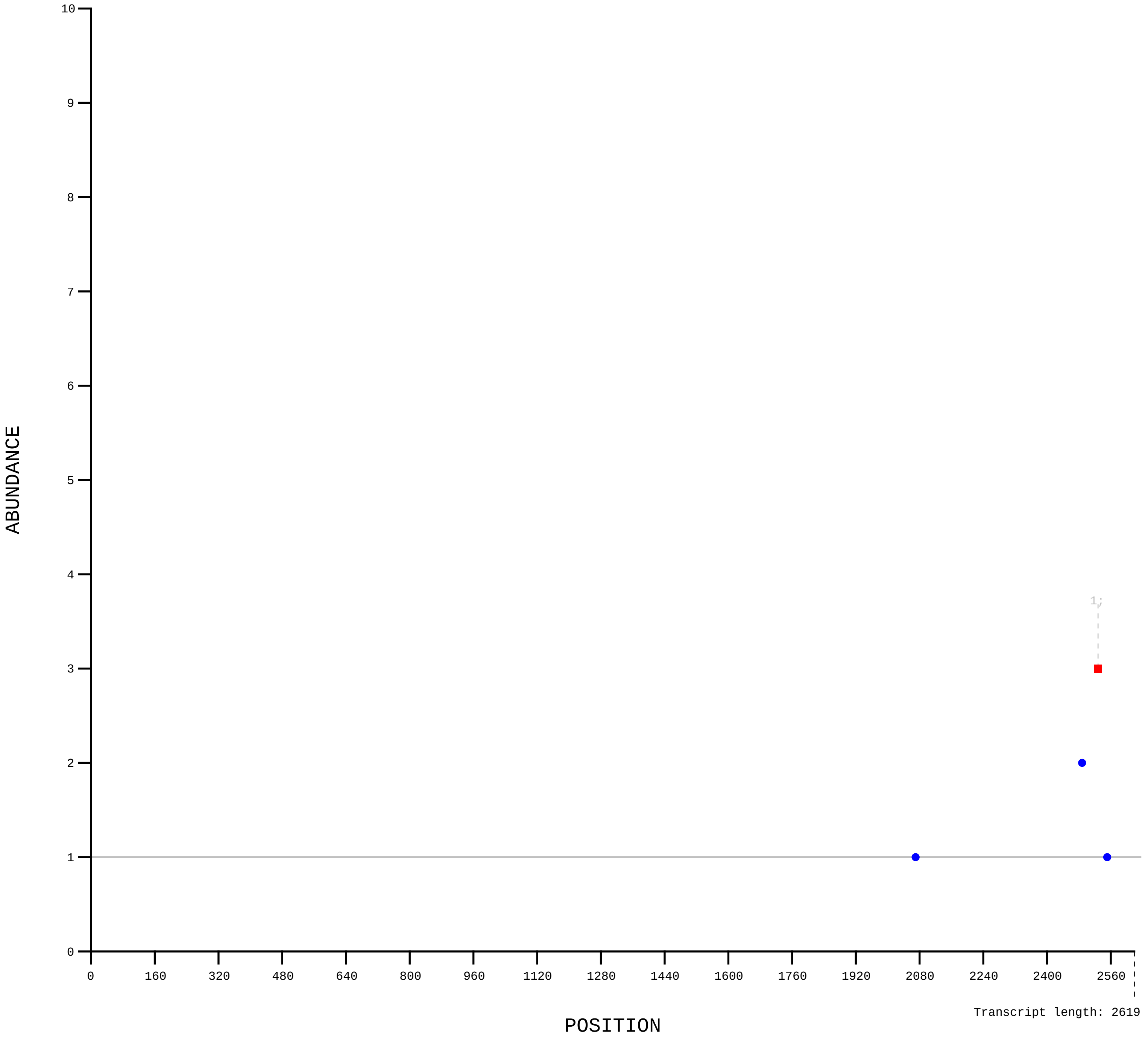

Category: 0 1 2 3 4
Degradome alignment: ● Median:

```
0  #1  Position:2528  Abundance: 3.00(deg) 9(sRNA)
   5'     TTAACCGAAGCAGGATCTAGC        3'  ID:
          |o||| |||||||o|||| |             Score: 4.0
   3' CAAGAGTTGACTTCGTCTTAGAACAACCAACG 5'  p-value: 0.0
```

FOXG_01612T0 | Fusarium oxysporum f. sp. lycopersici 4287 hypothetical protein (4638 nt)

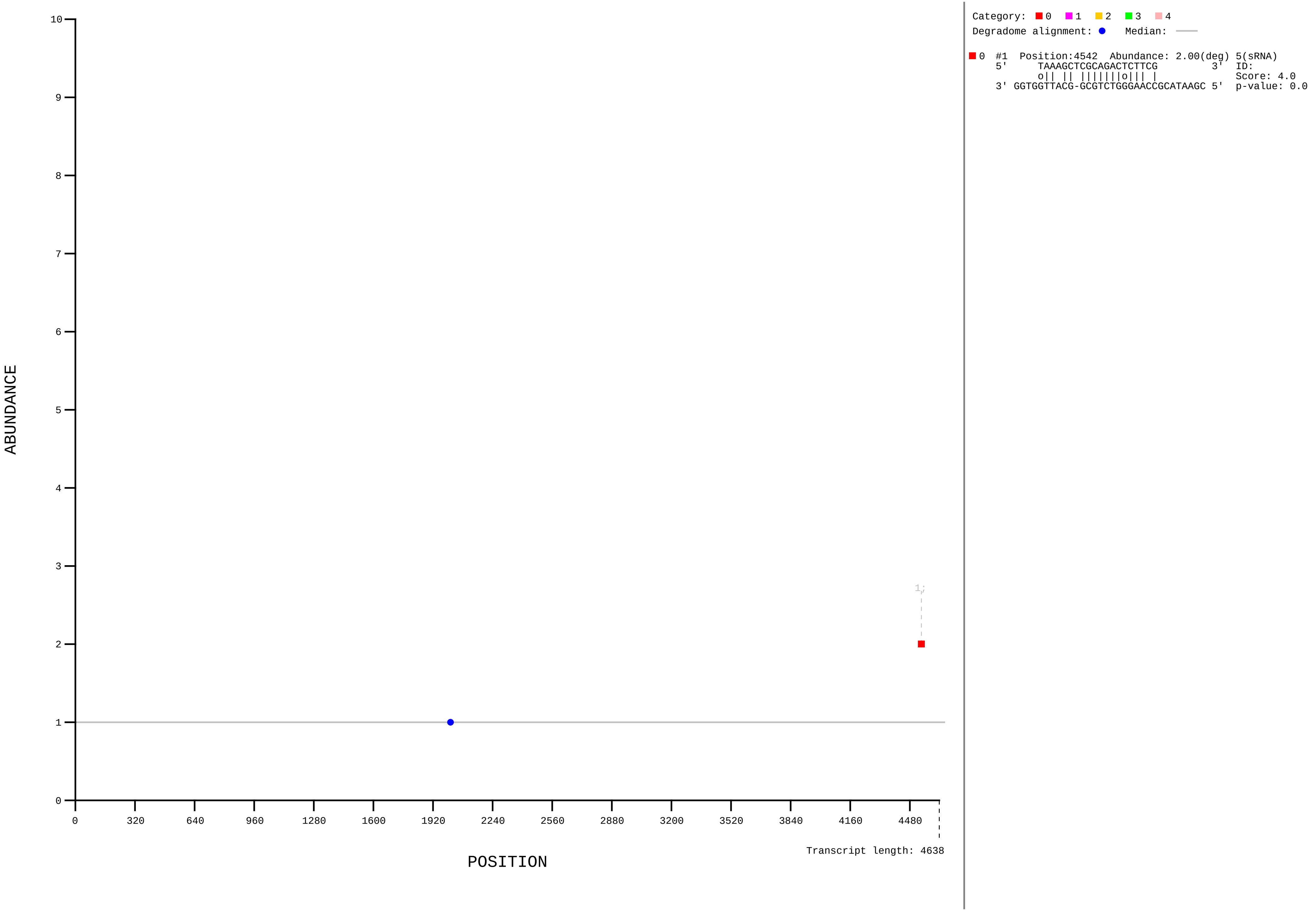

FOXG_09230T0 | Fusarium oxysporum f. sp. lycopersici 4287 hypothetical protein (2679 nt)

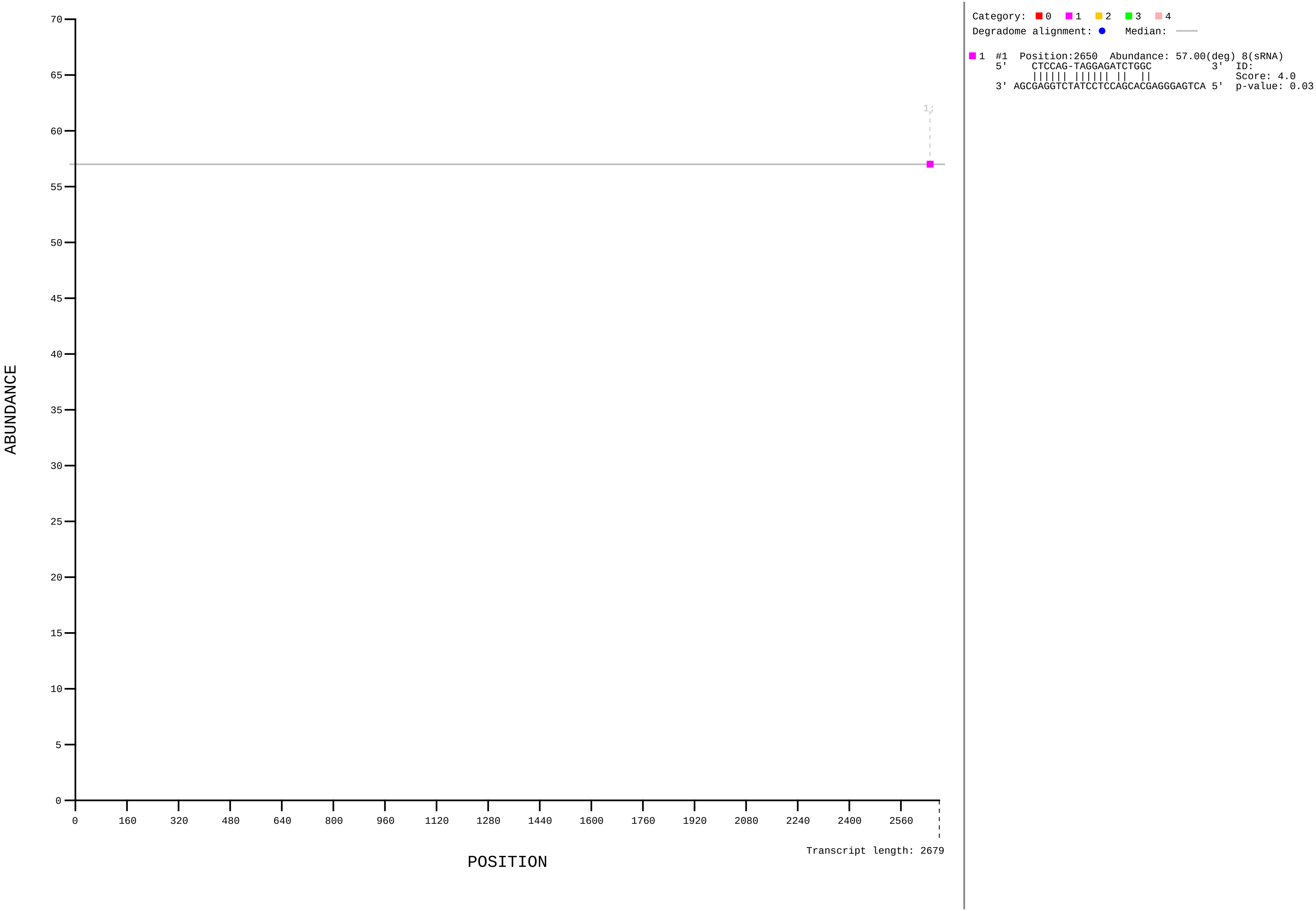

FOXG_05210T0 | Fusarium oxysporum f. sp. lycopersici 4287 tyrosine decarboxylase (1503 nt)

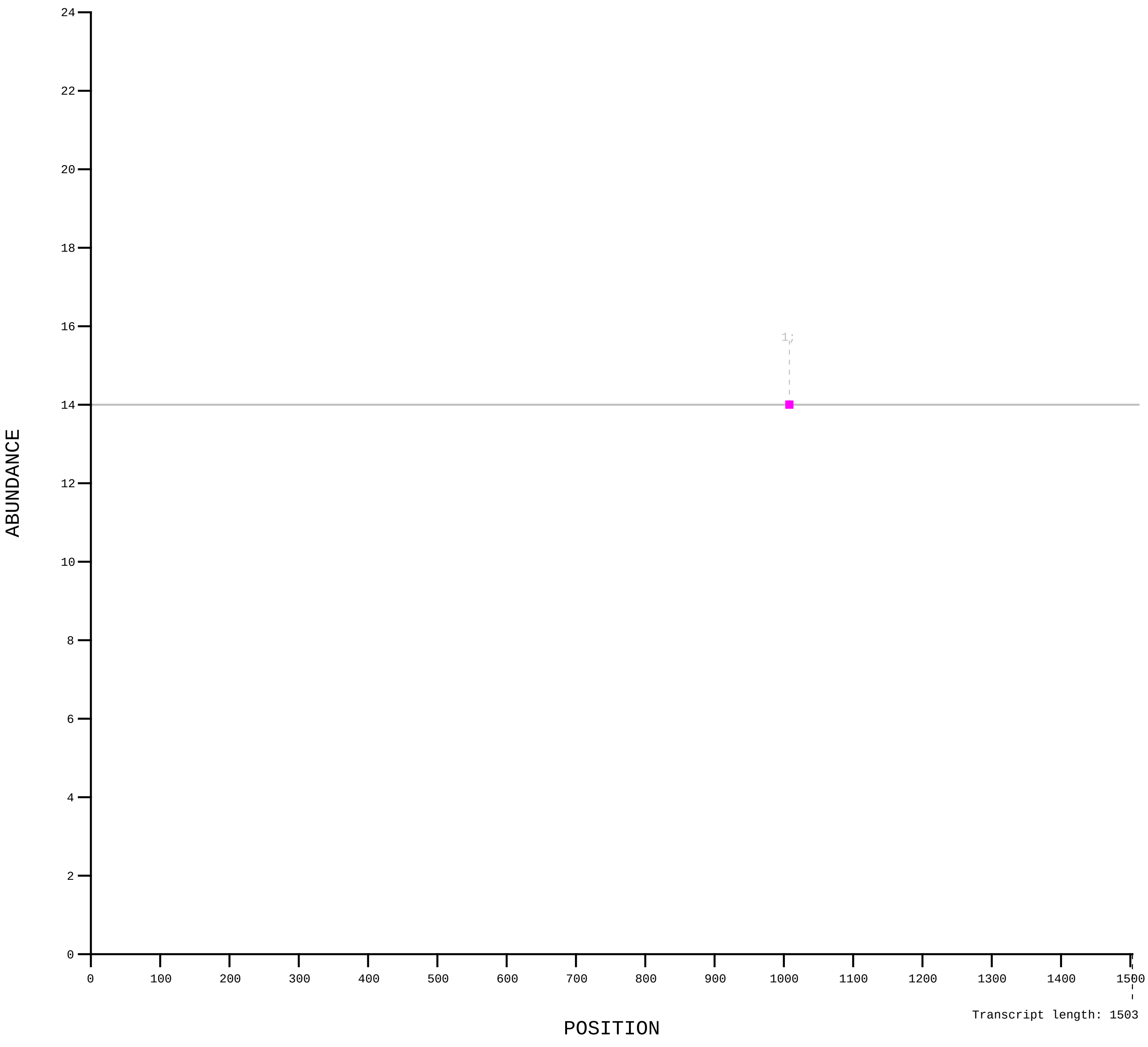

Category: 0 1 2 3 4
Degradome alignment: ● Median:

```
1  #1  Position:1008  Abundance: 14.00(deg) 6(sRNA)
   5'     ACGGATCTGAAGCGTGGTA          3'  ID:
          ||o|| |||||| o|o|o|              Score: 4.0
   3' CTTCTGTCT-GACTTCCTATCGTCCACTAGCA 5'  p-value: 0.0
```

FOXG_09184T0 | Fusarium oxysporum f. sp. lycopersici 4287 Arc1p protein (1170 nt)

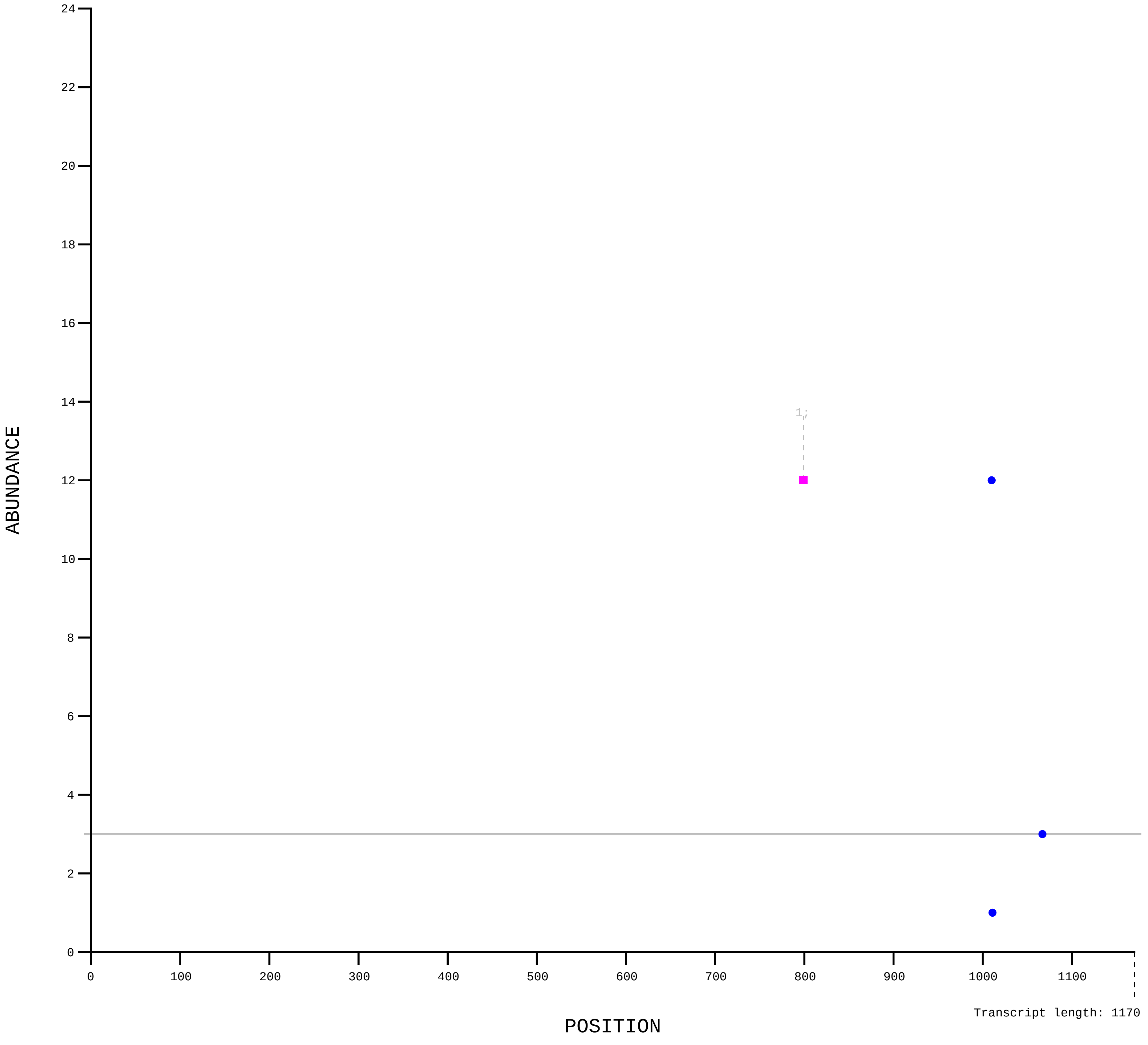

Category: 0 1 2 3 4
Degradome alignment: Median:

```
1  #1  Position:799  Abundance: 12.00(deg) 28(sRNA)
   5'     TGGGGTAGCAGAACGTTGA          3'  ID:
          ||o||| ||||| || o||               Score: 4.0
   3' CCGAACTCCAACGTCT-GCTGCTGCTGAAACG 5'  p-value: 0.0
```

FOXG_11178T0 | Fusarium oxysporum f. sp. lycopersici 4287 hypothetical protein (2301 nt)

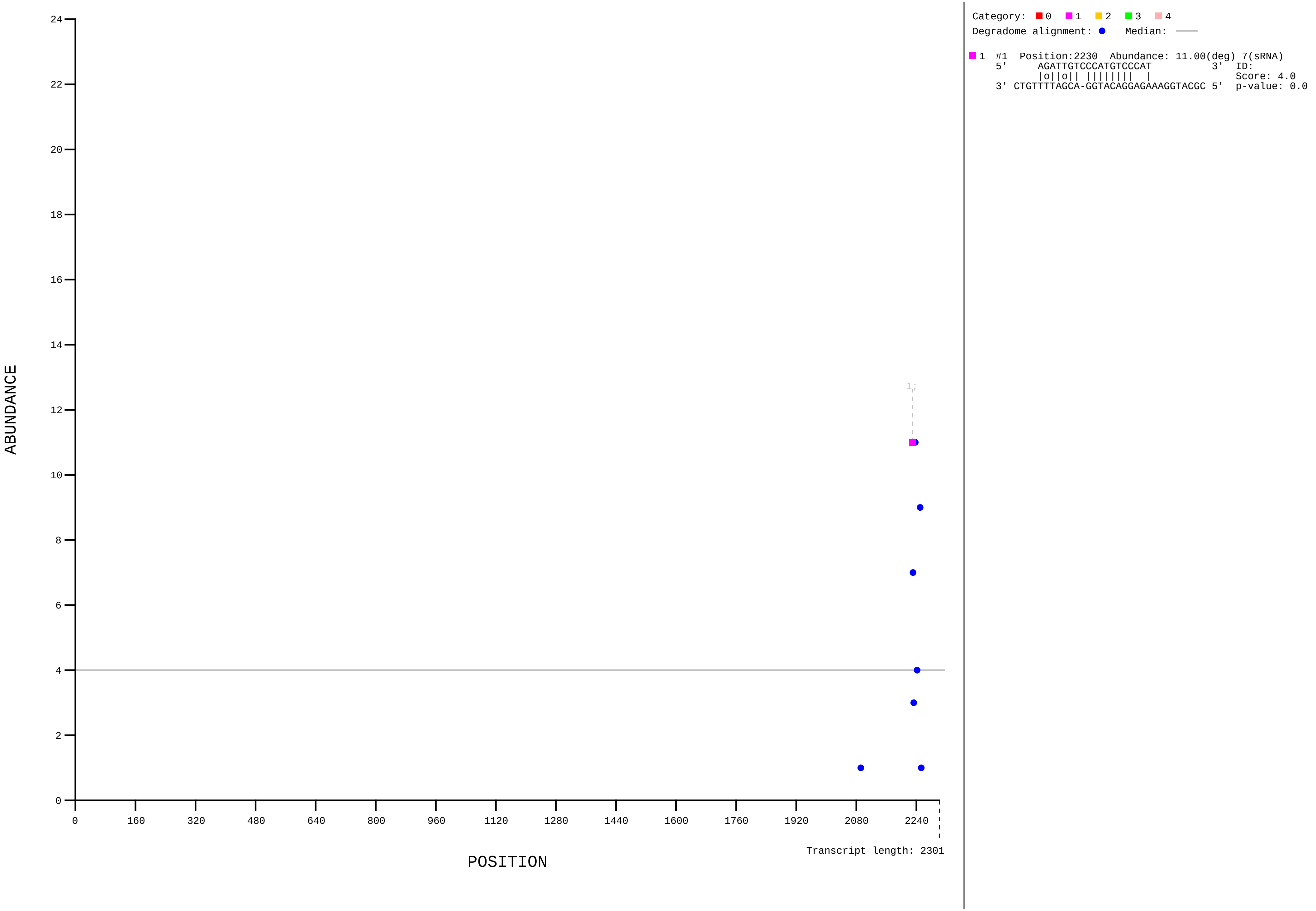

FOXG_00340T0 | Fusarium oxysporum f. sp. lycopersici 4287 hypothetical protein (19692 nt) Note(s): (truncated)

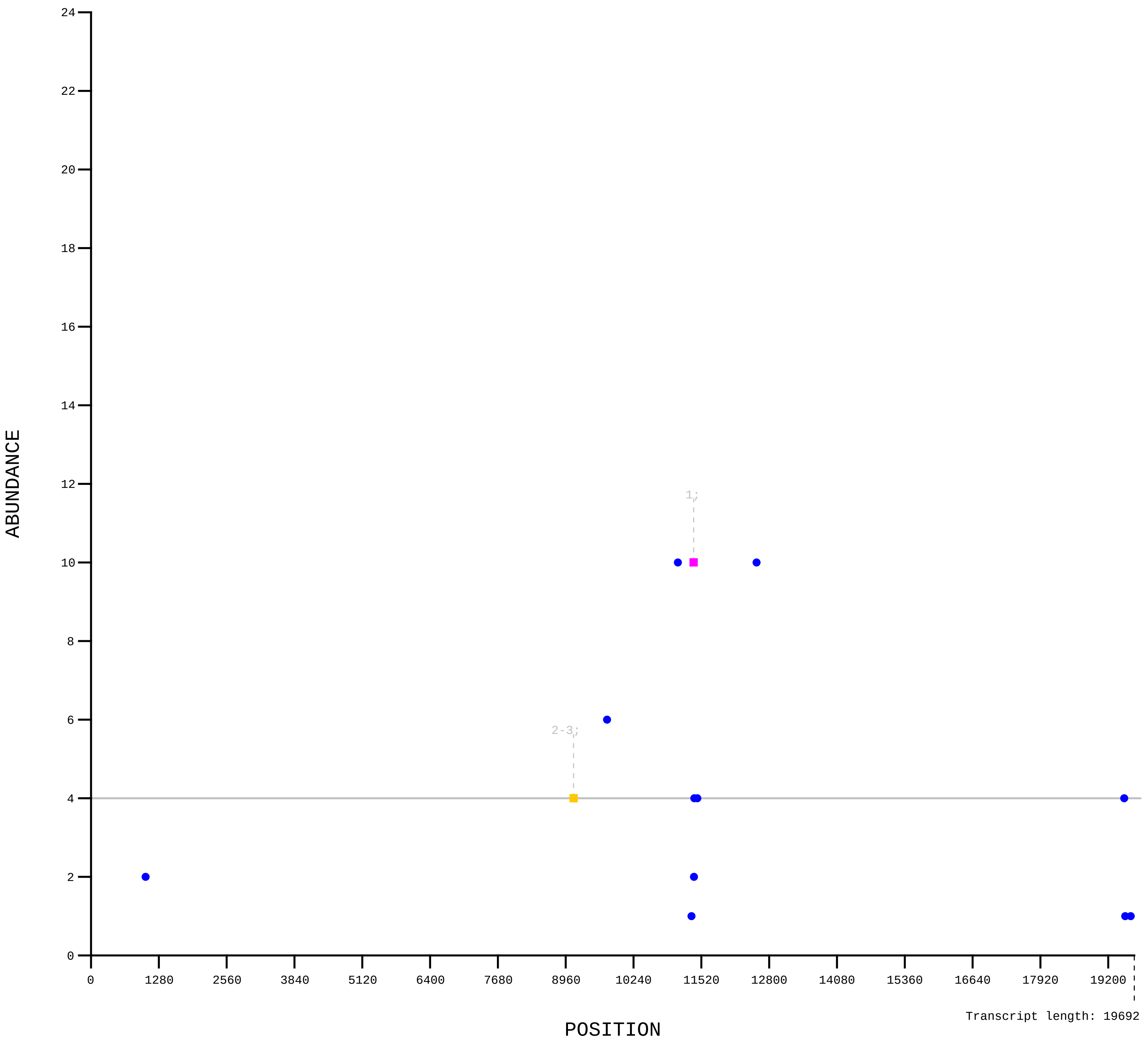


```
Category: ■0 ■1 ■2 ■3 ■4
Degradome alignment: ●   Median: ——

■1  #1  Position:11375  Abundance: 10.00(deg) 9(sRNA)
    5'     TTCTCTTCGGAGGCGTCTG          3'  ID:
            o||||||o||||| ||o|              Score: 3.5
    3' ACCATGGAGAAGTCTCCG-AGGCCAAAGGAAC 5'  p-value: 0.0

■2  #2  Position:9108  Abundance: 4.00(deg) 8(sRNA)
    5'     CTCCAGTAGGAGATCTGGC          3'  ID:
           |o||| ||||||||| | |              Score: 3.5
    3' GCCCGGGGTAATCCTCTAGCCAGACGCTCGCA 5'  p-value: 0.01

■2  #3  Position:9108  Abundance: 4.00(deg) 6(sRNA)
    5'     CTCCAGTAGGAGATTTGGC          3'  ID:
           |o||| ||||||||o | |              Score: 4.0
    3' GCCCGGGGTAATCCTCTAGCCAGACGCTCGCA 5'  p-value: 0.01
```

FOXG_07584T0 | Fusarium oxysporum f. sp. lycopersici 4287 hypothetical protein (441 nt)

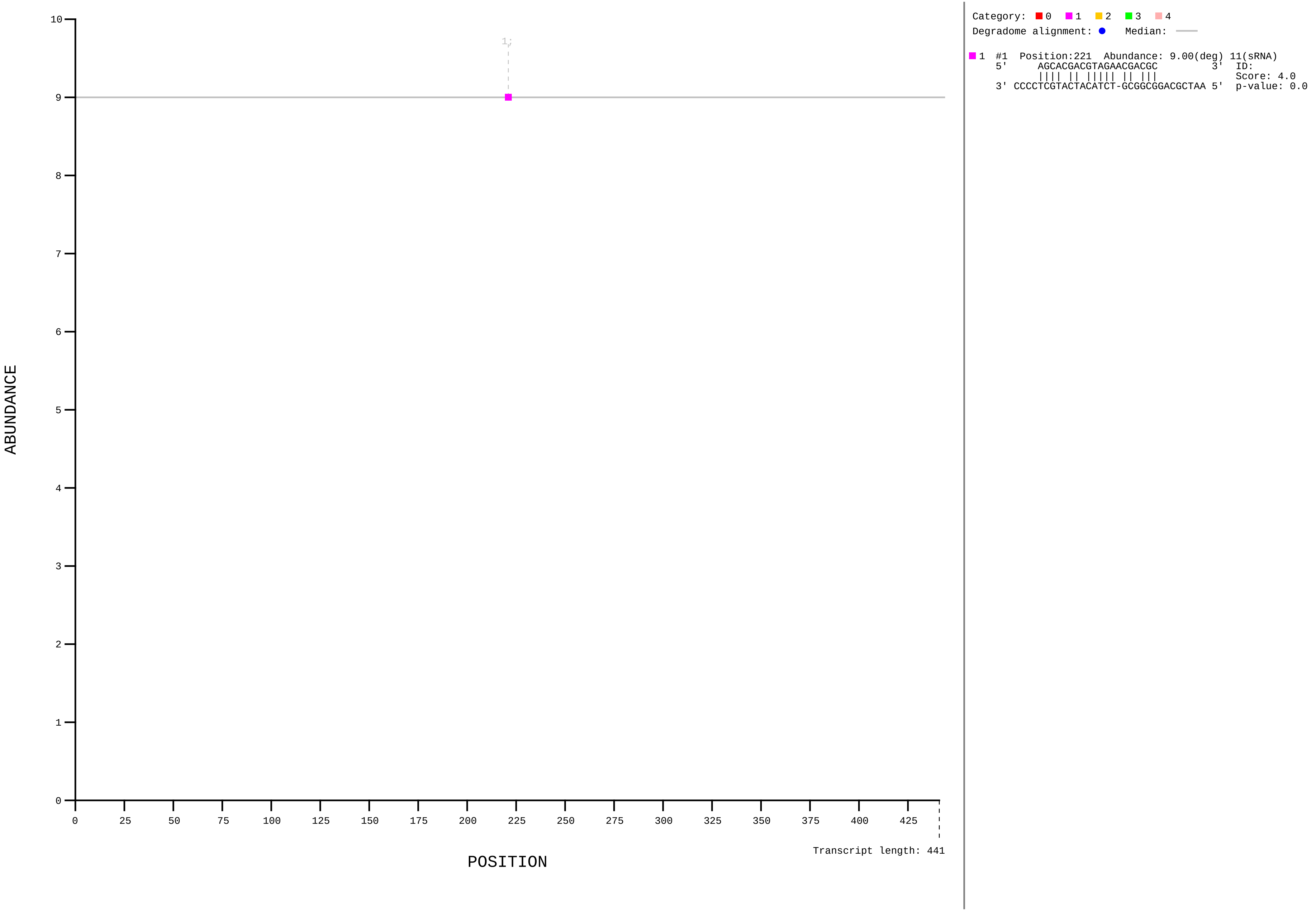

FOXG_11413T0 | Fusarium oxysporum f. sp. lycopersici 4287 hypothetical protein (837 nt)

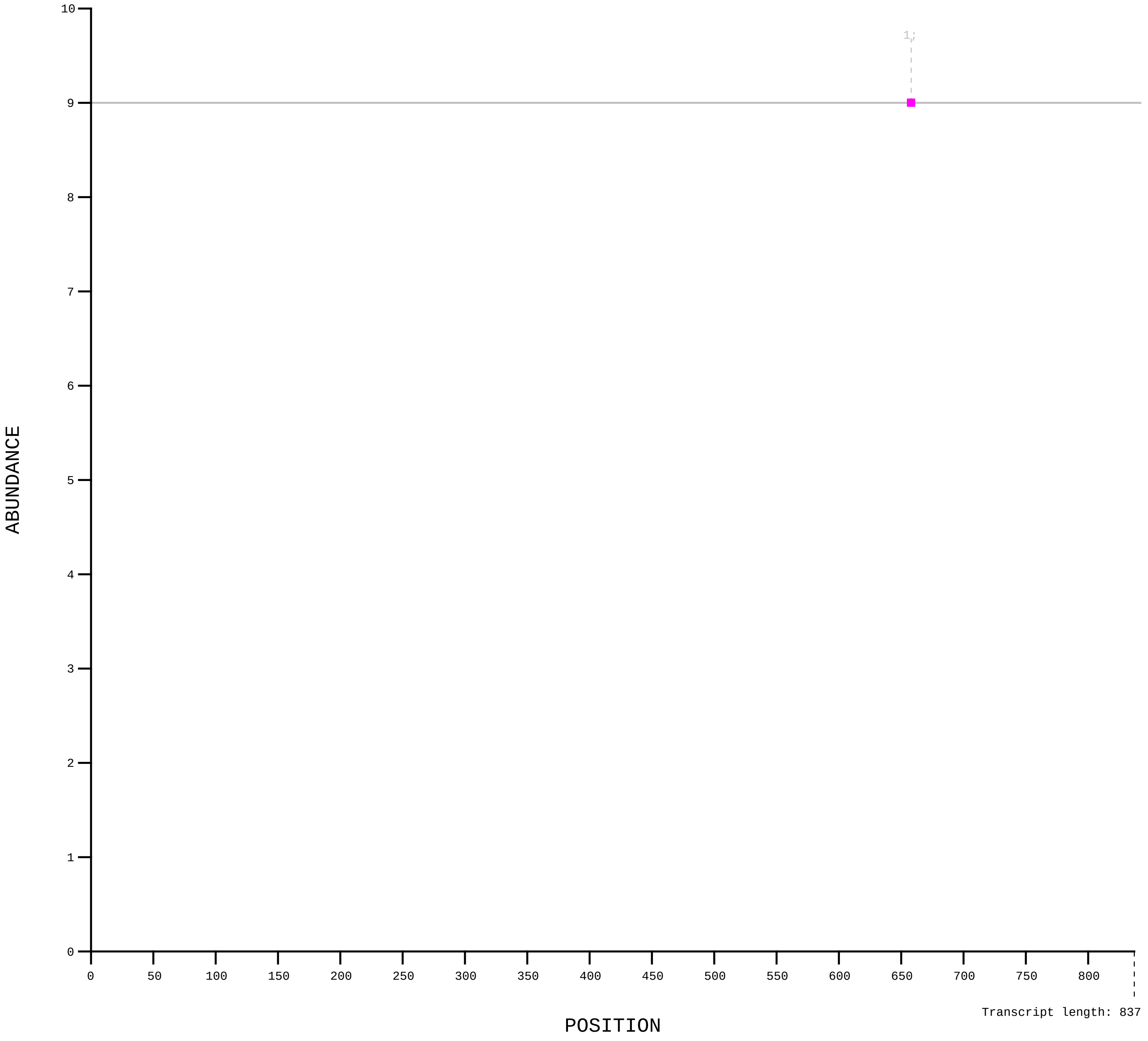

Category: 0 1 2 3 4
Degradome alignment: Median:

1 #1 Position:658 Abundance: 9.00(deg) 5(sRNA)

```
5'     CTGATACTCATACTTTTCTTG       3'  ID:
       ||||| |o||| |||| ||||           Score: 3.5
3' TACCGACTAGGGGTACGAAA-GAACAGGAGGC 5'  p-value: 0.0
```

FOXG_13120T0 | Fusarium oxysporum f. sp. lycopersici 4287 hypothetical protein (543 nt)

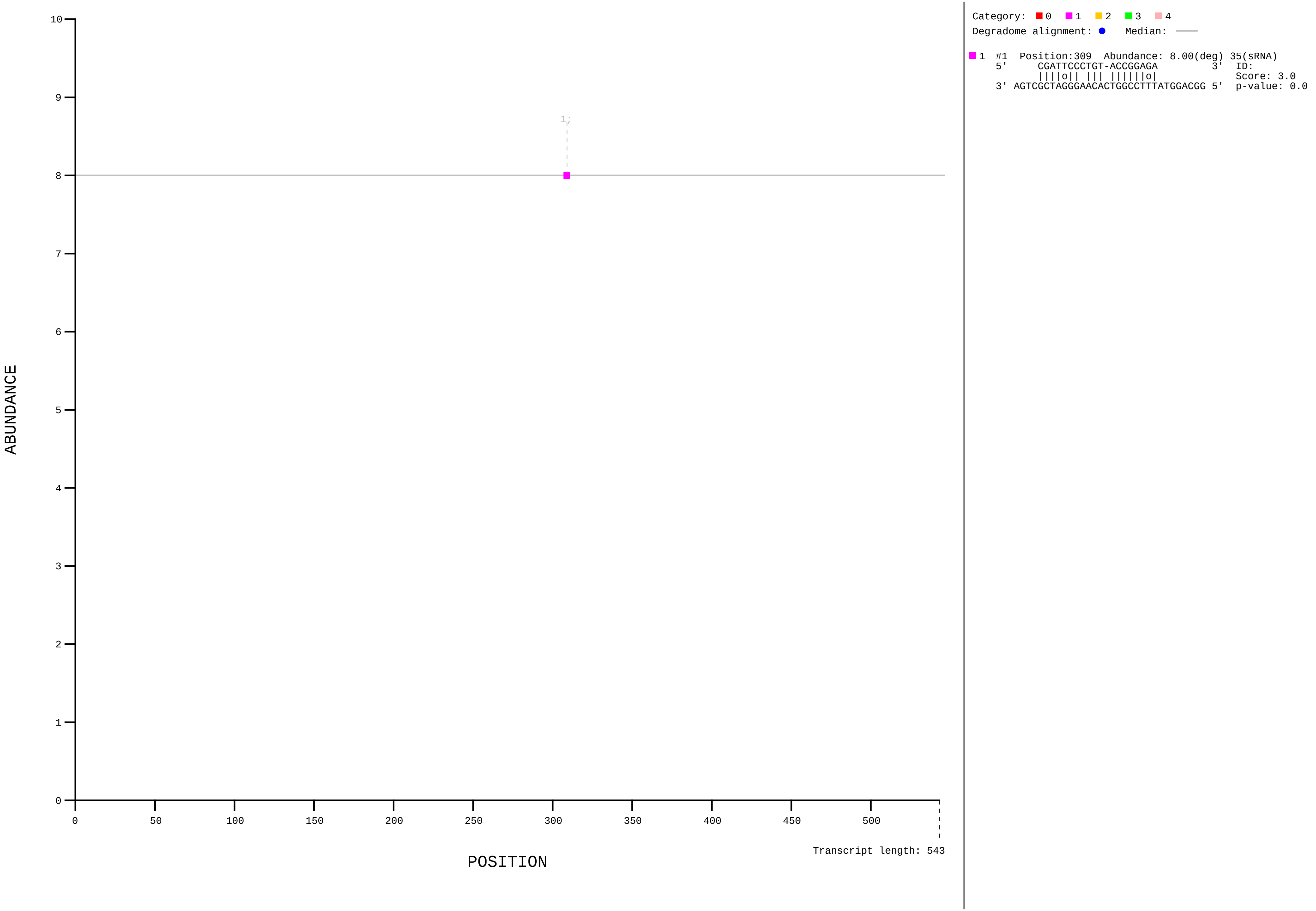

FOXG_11704T0 | Fusarium oxysporum f. sp. lycopersici 4287 hypothetical protein (1191 nt)

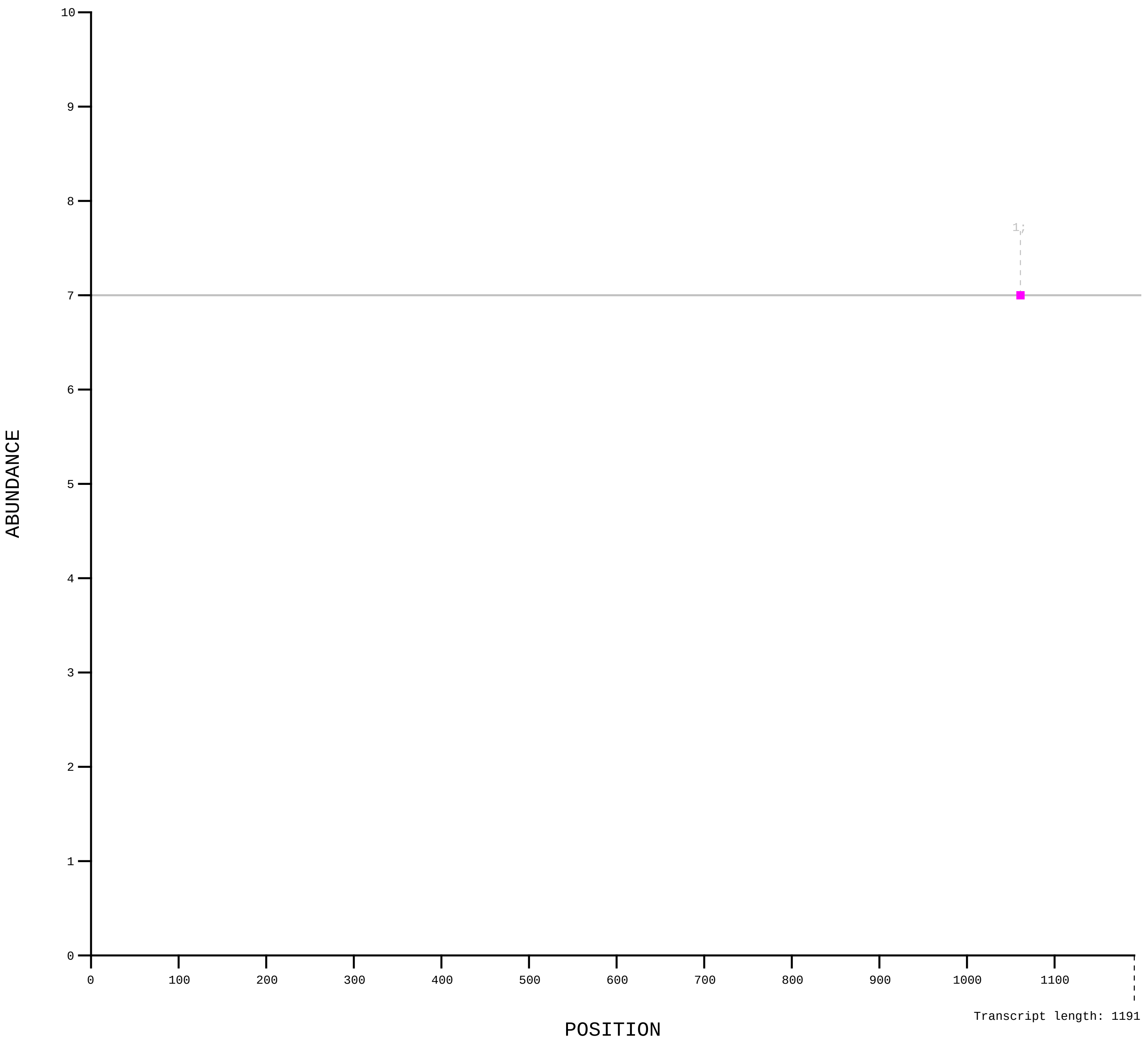

Category: 0 1 2 3 4

Degradome alignment: Median:

```
1  #1  Position:1061  Abundance: 7.00(deg) 14(sRNA)
   5'      TCCACGATTTTCTTATGAA          3'  ID:
           || |||||o||||o|  ||              Score: 4.0
   3' TAGAAGTTGCTAGAAGAGTTGTTGTATGAGGG 5'  p-value: 0.02
```

FOXG_02353T0 | Fusarium oxysporum f. sp. lycopersici 4287 hypothetical protein (1494 nt)

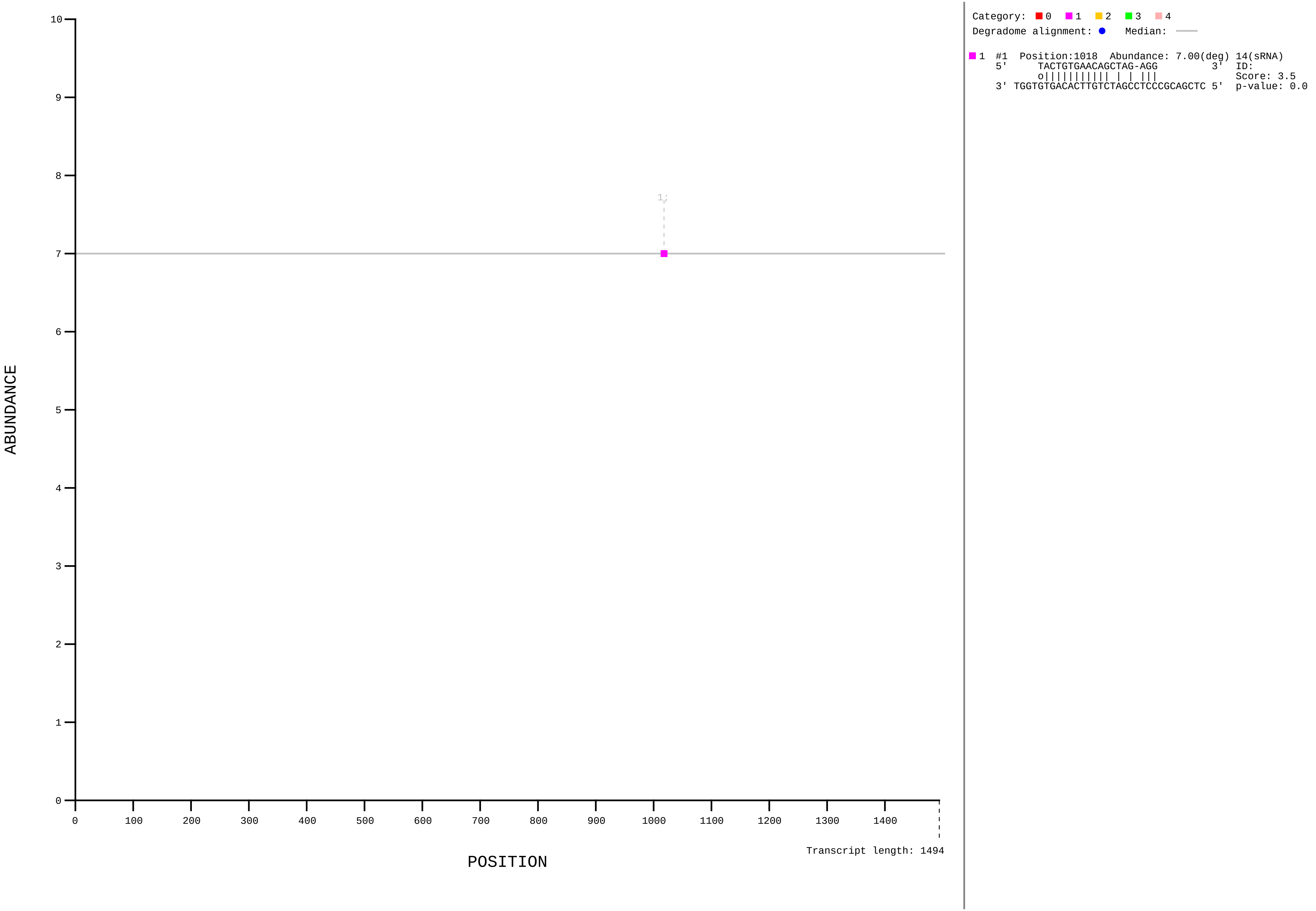

FOXG_05497T0 | Fusarium oxysporum f. sp. lycopersici 4287 mitochondrial precursor proteins import receptor (1863 nt)

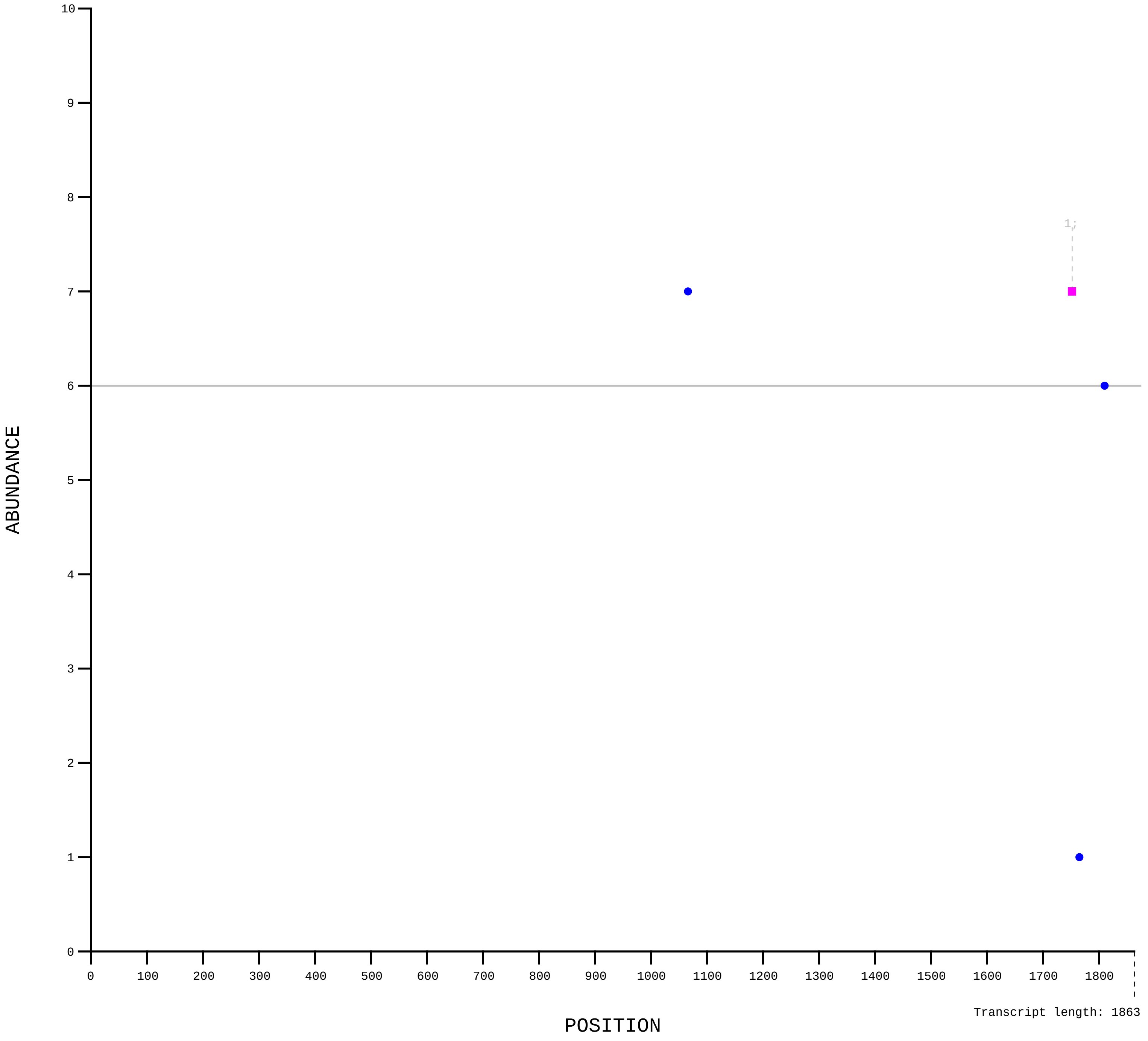

Category: 0 1 2 3 4
Degradome alignment: ● Median:

```
1  #1  Position:1752  Abundance: 7.00(deg) 5(sRNA)
   5'      TTCAGCATGTGACGAGTGG           3'  ID:
           o|||||||o||||o||  |               Score: 3.5
   3' AACGGAGTCGTATACTGTTCCGCAACTGTTAG 5'  p-value: 0.0
```

FOXG_01130T0 | Fusarium oxysporum f. sp. lycopersici 4287 hypothetical protein (666 nt)

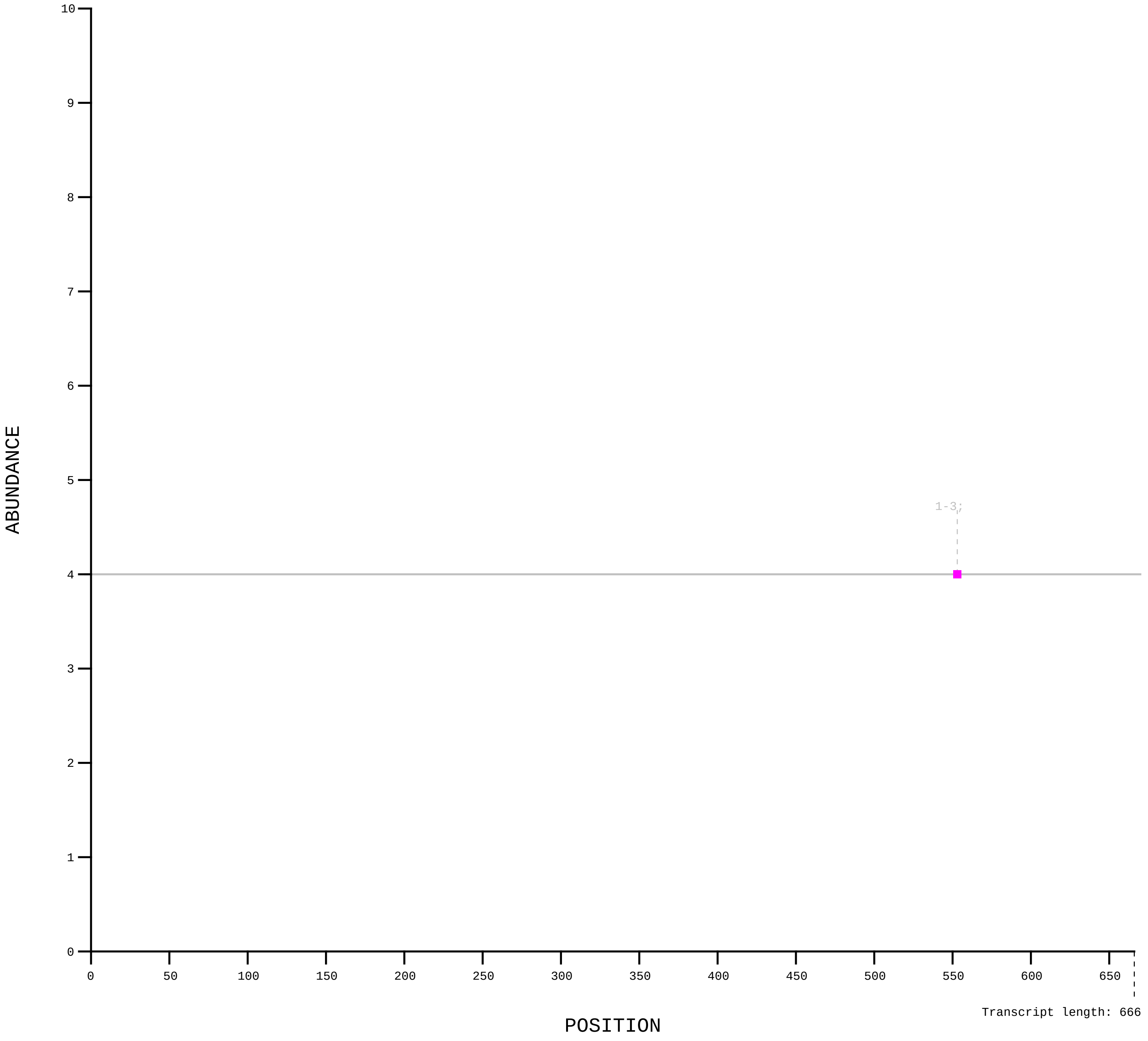

Category: 0 1 2 3 4
Degradome alignment: ● Median:

```
1  #1  Position:553  Abundance: 4.00(deg) 322(sRNA)
   5'     TAGAACATGTAGAATTAGAAGG       3'  ID:
          o|||| ||||||| || ||||o           Score: 4.0
   3' AGAGGTCTTCTACATCTCAACCTTCTCGATAG 5'  p-value: 0.0

1  #2  Position:553  Abundance: 4.00(deg) 27(sRNA)
   5'     TAGAACATGTAGAATTAGAAG        3'  ID:
          o|||| ||||||| || ||||            Score: 3.5
   3' AGAGGTCTTCTACATCTCAACCTTCTCGATAG 5'  p-value: 0.0

1  #3  Position:553  Abundance: 4.00(deg) 13(sRNA)
   5'     TAGAACATGTAGAATTAGA          3'  ID:
          o|||| ||||||| || ||              Score: 3.5
   3' AGAGGTCTTCTACATCTCAACCTTCTCGATAG 5'  p-value: 0.0
```

FOXG_07923T0 | Fusarium oxysporum f. sp. lycopersici 4287 hypothetical protein (1401 nt)

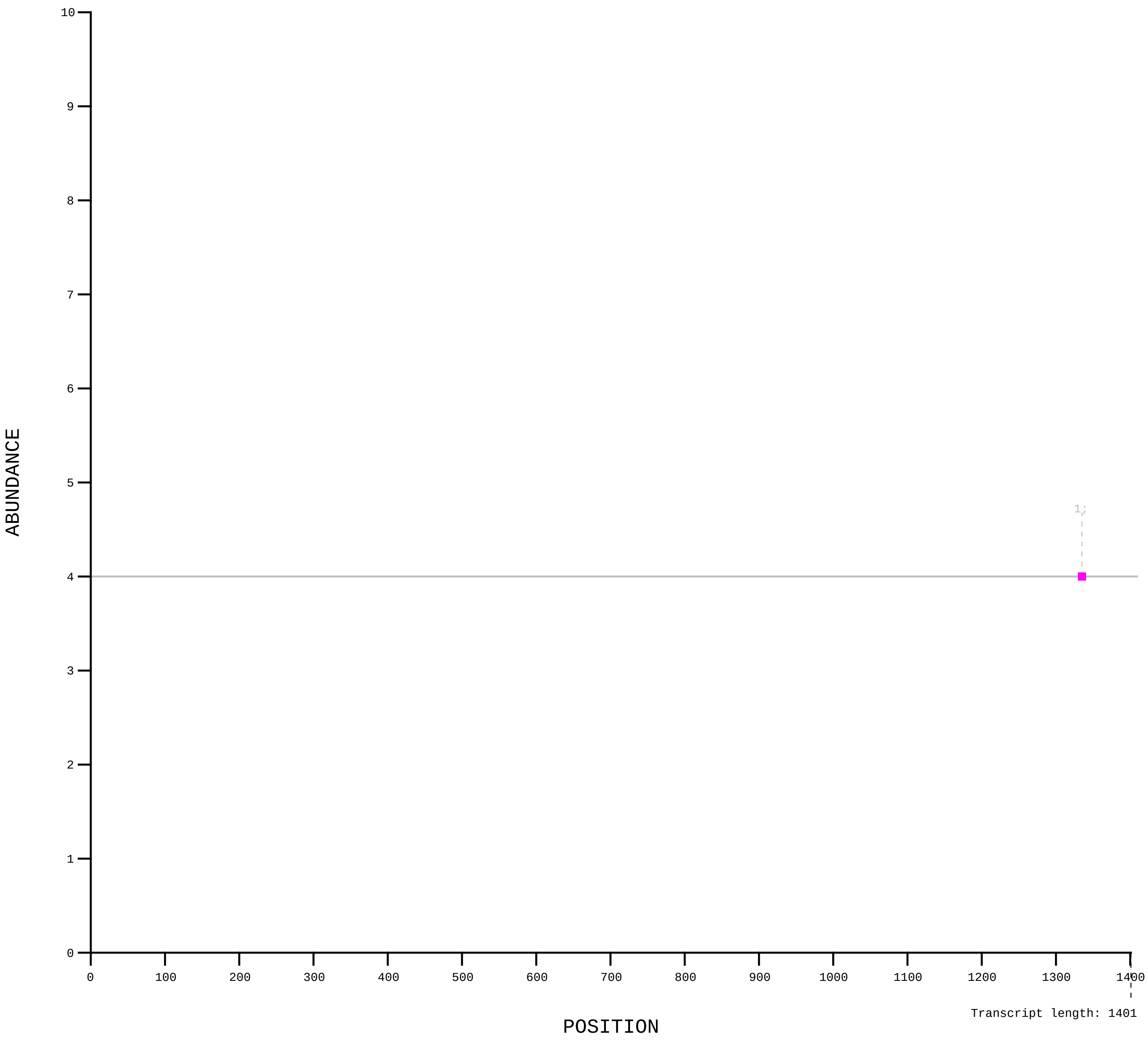


```
Category: 0 1 2 3 4
Degradome alignment:    Median:

1  #1  Position:1335  Abundance: 4.00(deg) 9(sRNA)
   5'     TGGTATCTATCGGATATGG          3'  ID:
          |||| ||o|||| || ||o              Score: 4.0
   3' ATCAACCACAGGTAGC-TAGACTTCTCTACCA 5'  p-value: 0.0
```

FOXG_01731T0 | Fusarium oxysporum f. sp. lycopersici 4287 NF-X1 finger transcription factor (2892 nt)

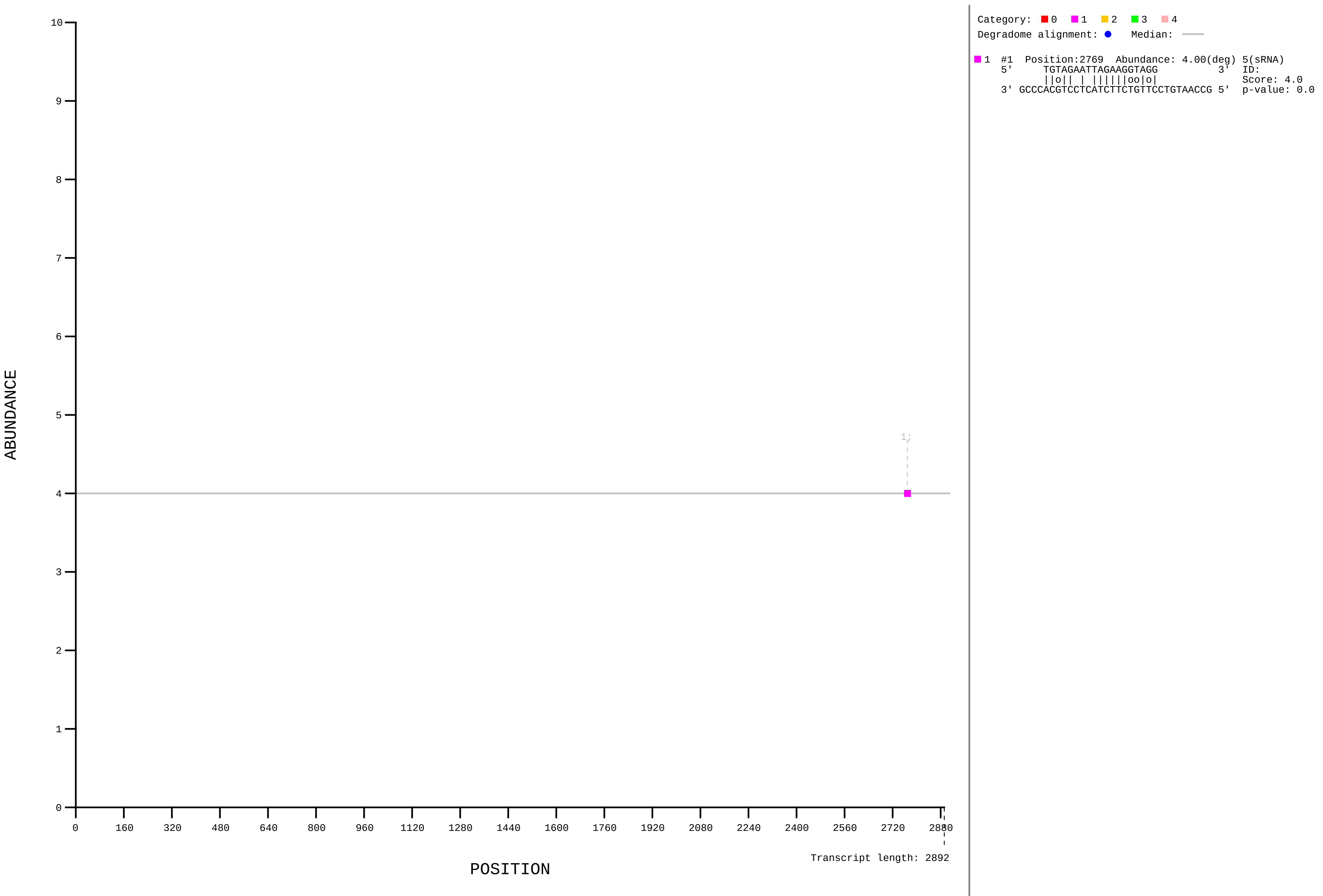

FOXG_01784T0 | Fusarium oxysporum f. sp. lycopersici 4287 glutamic acid decarboxylase isoform 67 (1521 nt)

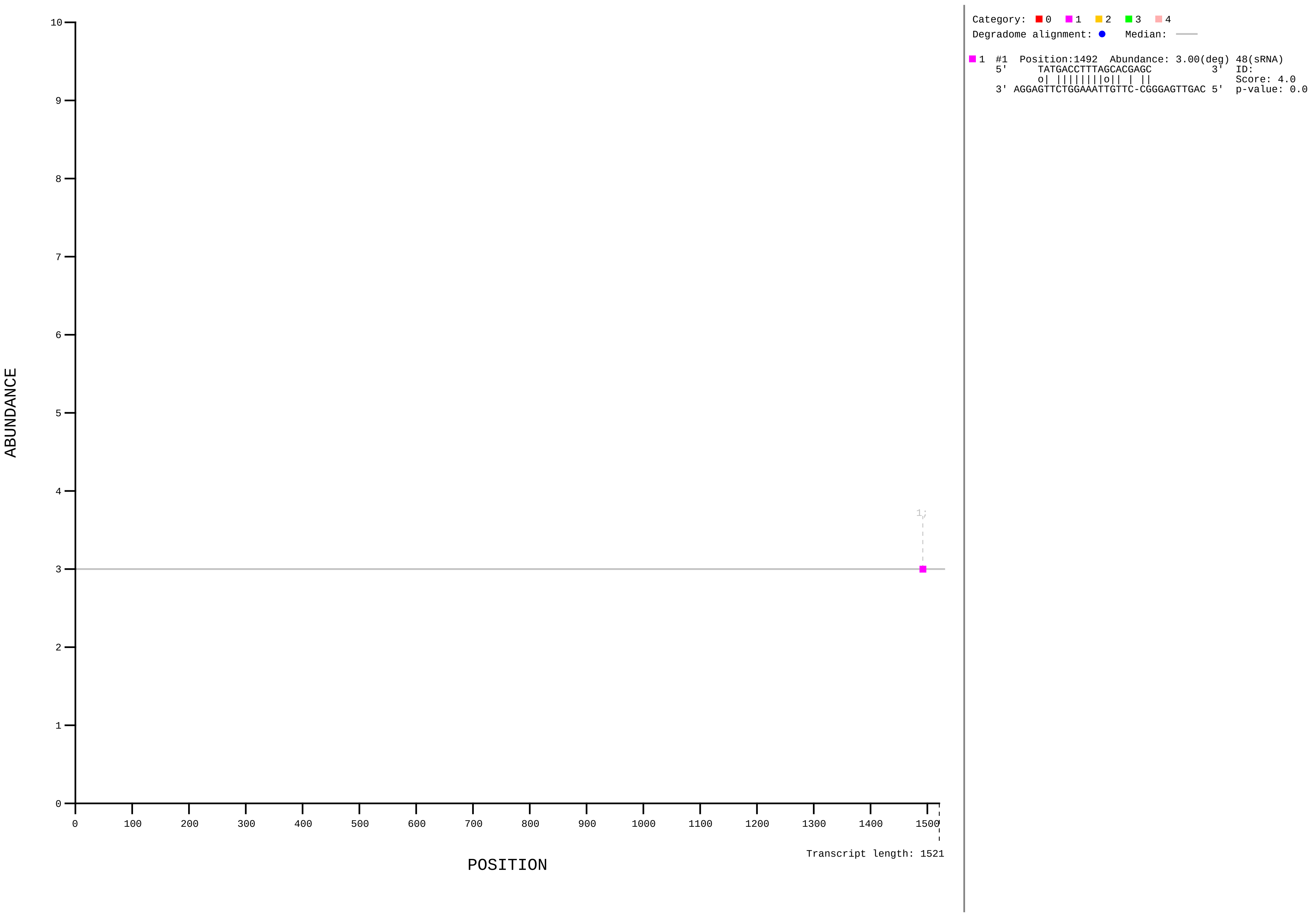

FOXG_03705T0 | Fusarium oxysporum f. sp. lycopersici 4287 hypothetical protein (1551 nt) Note(s): (truncated)

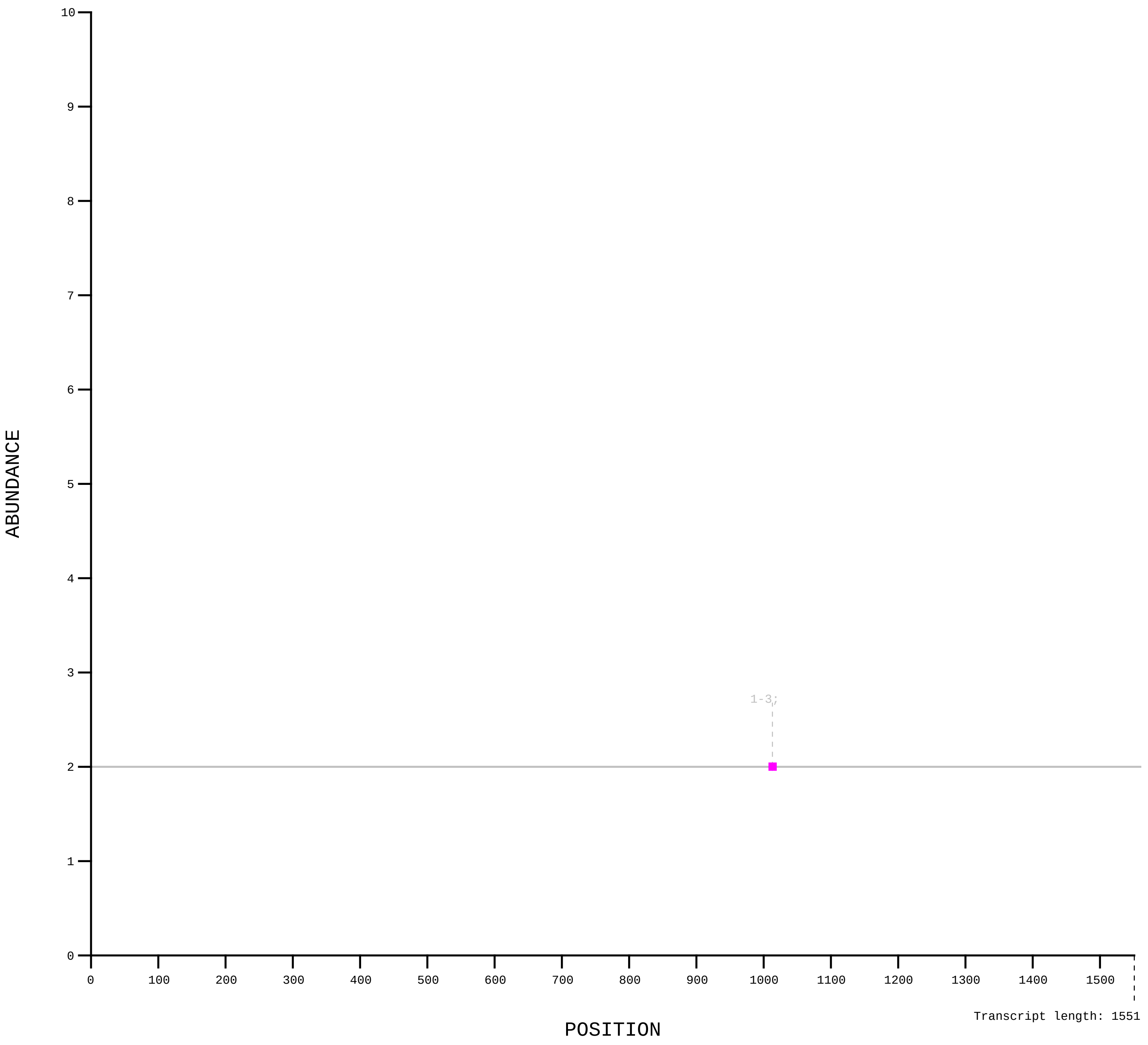


```
Category:  0  1  2  3  4
Degradome alignment:     Median:

1  #1  Position:1013  Abundance: 2.00(deg) 2473(sRNA)
   5'     TATAAAACGAAGAACAAGAGG        3'  ID:
          o||| || ||||| |||||o|            Score: 4.0
   3' CGCGGTATCTT-CTTCTAGTTCTTCGAGAGCT 5'  p-value: 0.0

1  #2  Position:1013  Abundance: 2.00(deg) 161(sRNA)
   5'     TATAAAACGAAGAACAAGAG         3'  ID:
          o||| || ||||| |||||o             Score: 4.0
   3' CGCGGTATCTT-CTTCTAGTTCTTCGAGAGCT 5'  p-value: 0.0

1  #3  Position:1013  Abundance: 2.00(deg) 71(sRNA)
   5'     TATAAAACGAAGAACAAGA          3'  ID:
          o||| || ||||| |||||              Score: 3.5
   3' CGCGGTATCTT-CTTCTAGTTCTTCGAGAGCT 5'  p-value: 0.0
```

FOXG_07896T0 | Fusarium oxysporum f. sp. lycopersici 4287 FMI1 protein (897 nt)

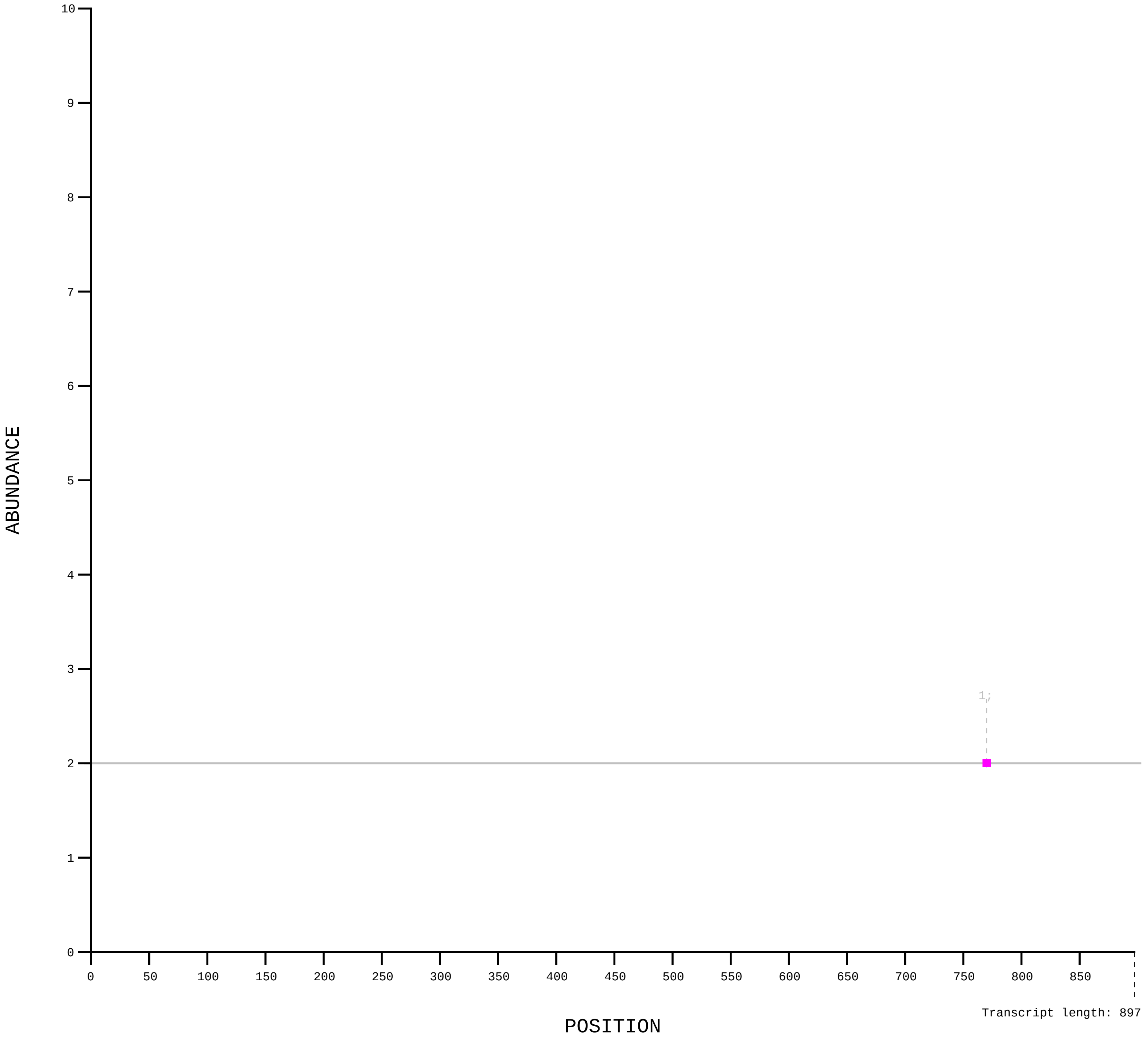

Category: 0 1 2 3 4
Degradome alignment: Median:

```
1  #1  Position:770  Abundance: 2.00(deg) 10(sRNA)
   5'     CGATAGCAGATTCTGCA-CCAGC      3'  ID:
          | ||| ||o||||o||| |||||          Score: 4.0
   3' AGAAGGTATGGTTTAAGGCGTAGGTCGGTTTC 5'  p-value: 0.0
```

FOXG_13343T0 | Fusarium oxysporum f. sp. lycopersici 4287 hypothetical protein (867 nt)

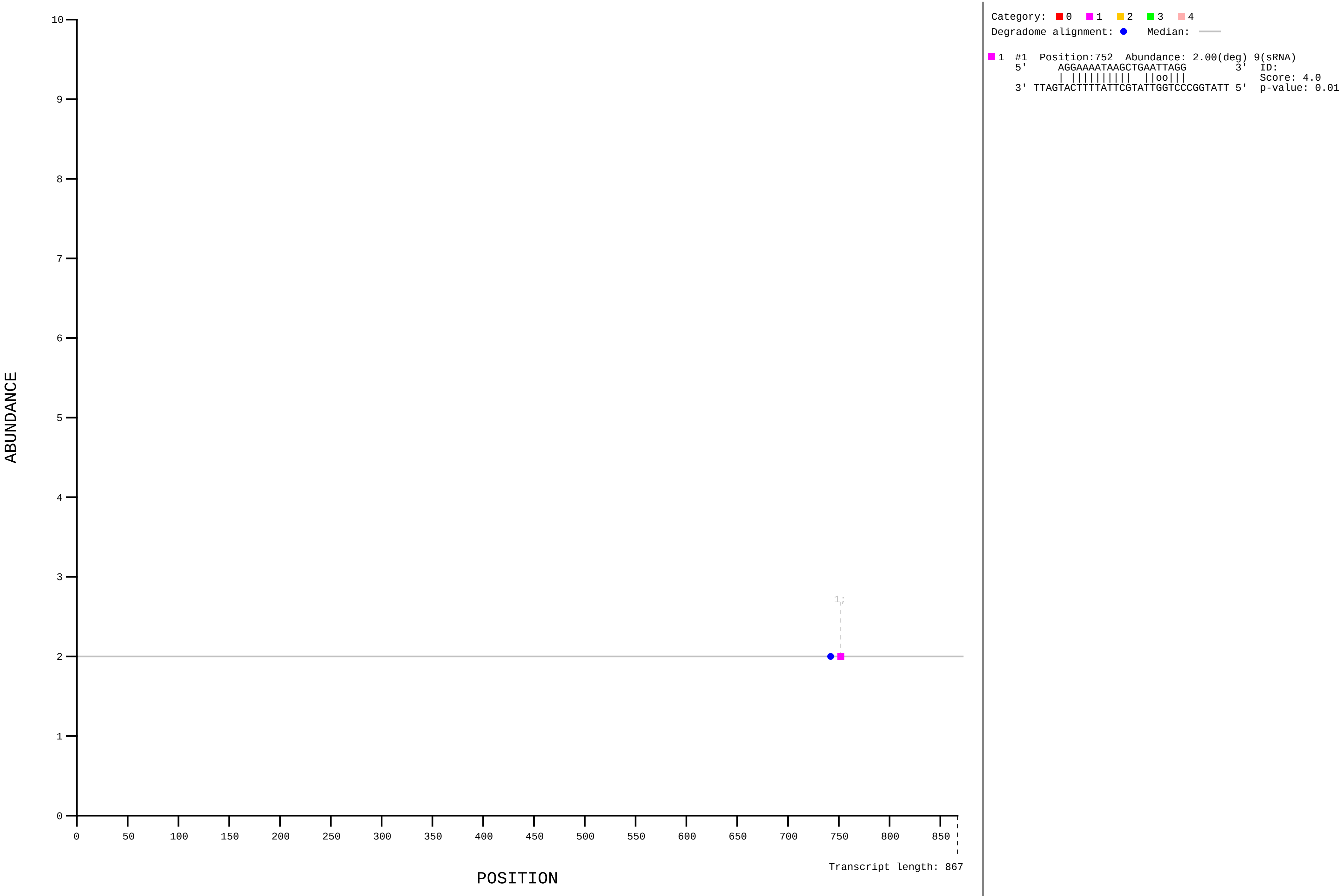

FOXG_14860T0 | Fusarium oxysporum f. sp. lycopersici 4287 hypothetical protein (777 nt) Note(s): (truncated)

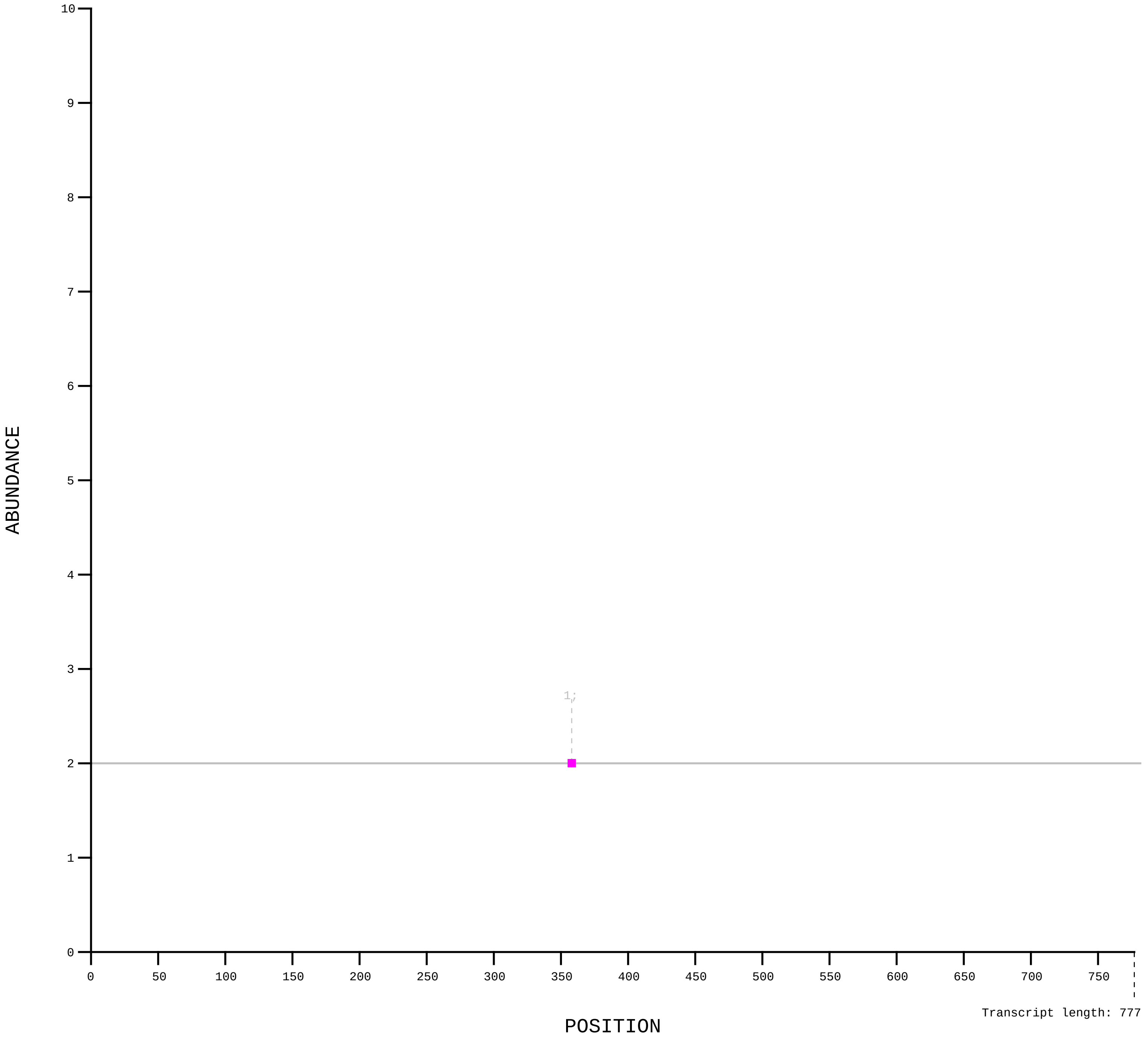

Category: 0 1 2 3 4
Degradome alignment: Median:

```
1  #1  Position:358  Abundance: 2.00(deg) 5(sRNA)
   5'     TTGAGAATCCTGGCGGACGG         3'  ID:
          ||| |||o|||||||||| ||            Score: 2.5
   3' GAGAAAC-CTTGGGACCGCCTACCTGGTATTG 5'  p-value: 0.0
```

FOXG_01961T0 | Fusarium oxysporum f. sp. lycopersici 4287 hypothetical protein (2367 nt)

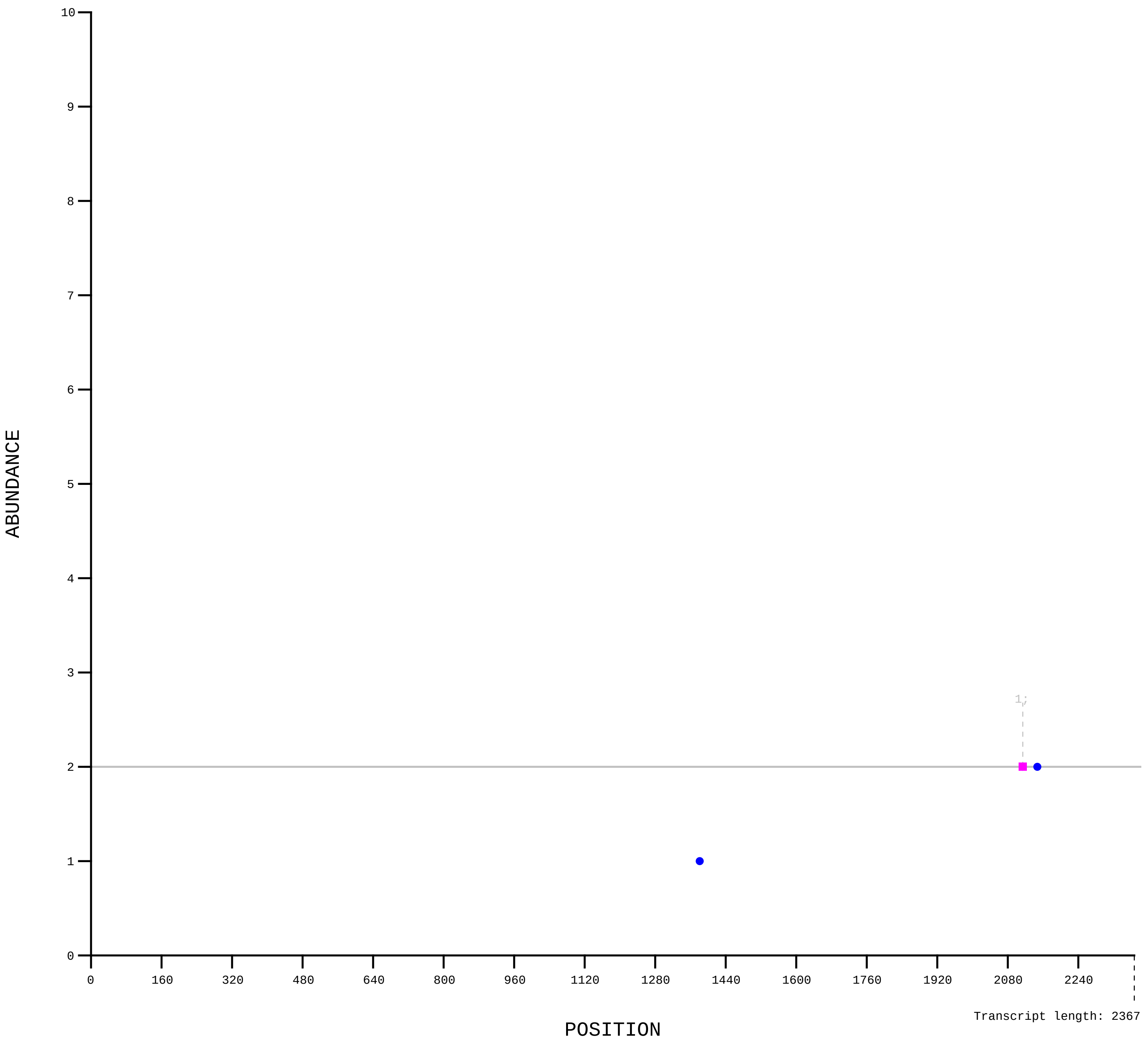

Category: 0 1 2 3 4
Degradome alignment: ● Median:

```
1  #1  Position:2114  Abundance: 2.00(deg) 5(sRNA)
   5'     AACTATATGTAGGTTCTTCTATA      3'  ID:
          ||||||| |||||| ||||o ||          Score: 3.5
   3' TCTATTGATAT-CATCCATGAAGGCATTGATA 5'  p-value: 0.0
```

FOXG_07841T0 | Fusarium oxysporum f. sp. lycopersici 4287 formate dehydrogenase (1098 nt)

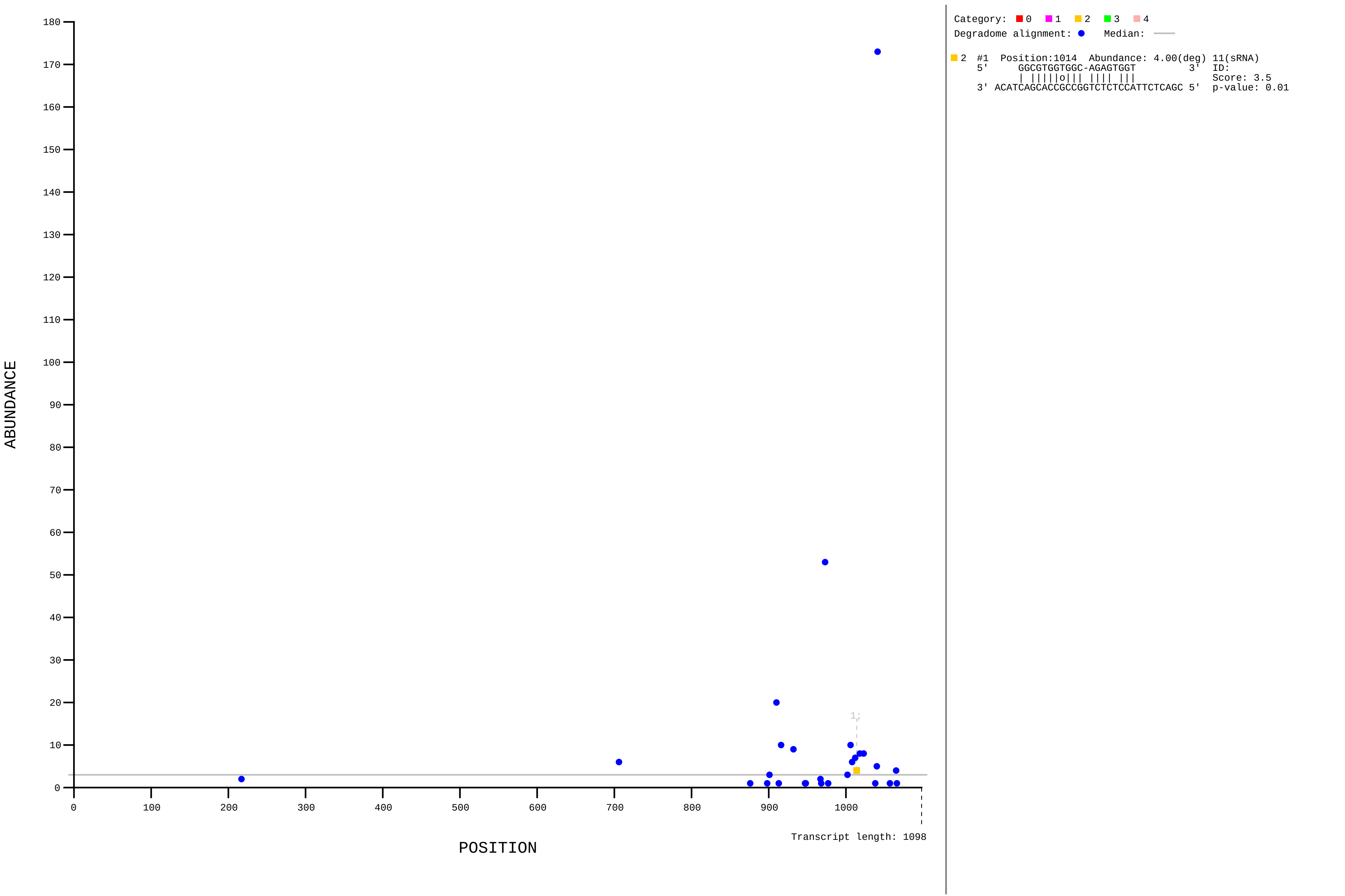

FOXG_16470T0 | Fusarium oxysporum f. sp. lycopersici 4287 hypothetical protein (762 nt)

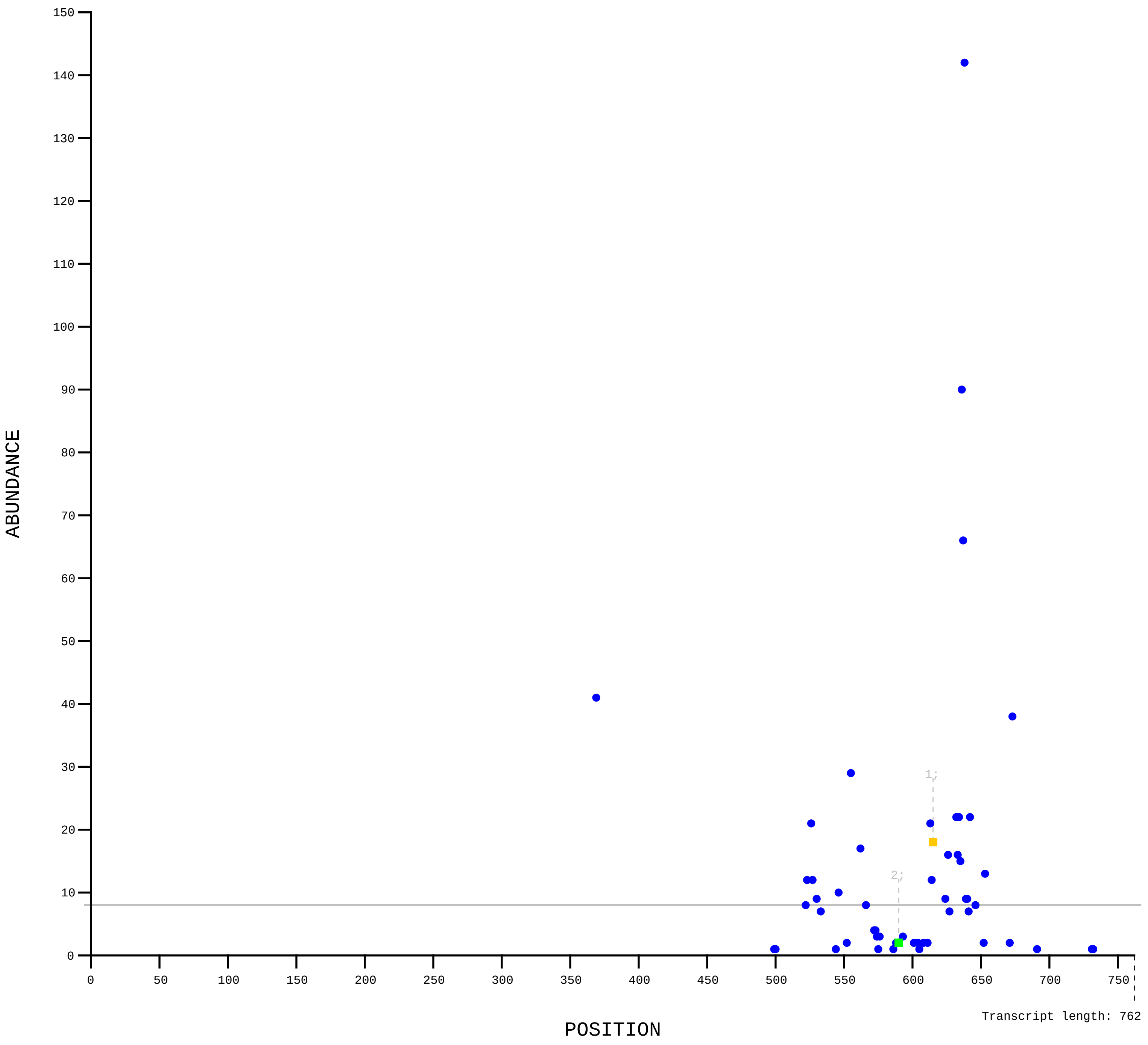

Category: 0 1 2 3 4
Degradome alignment: Median:

2 #1 Position:615 Abundance: 18.00(deg) 12(sRNA)

```
5'      AGGATTAAGCTCATGTTTT          3'  ID:
        |||| |||||| ||  |||              Score: 4.0
3' GTTGTCCT-ATTCGACTAGTAAAAGCGTTTAC 5'  p-value: 0.01
```

3 #2 Position:590 Abundance: 2.00(deg) 14(sRNA)

```
5'      TGATGTGTCGTCTCCGGAC          3'  ID:
        ||||o|o| |||| ||||               Score: 4.0
3' GTTTACTATATA-CAGAAGCCTTATCACTATA 5'  p-value: 0.0
```

FOXG_15708T0 | Fusarium oxysporum f. sp. lycopersici 4287 hypothetical protein (8859 nt) Note(s): (truncated)

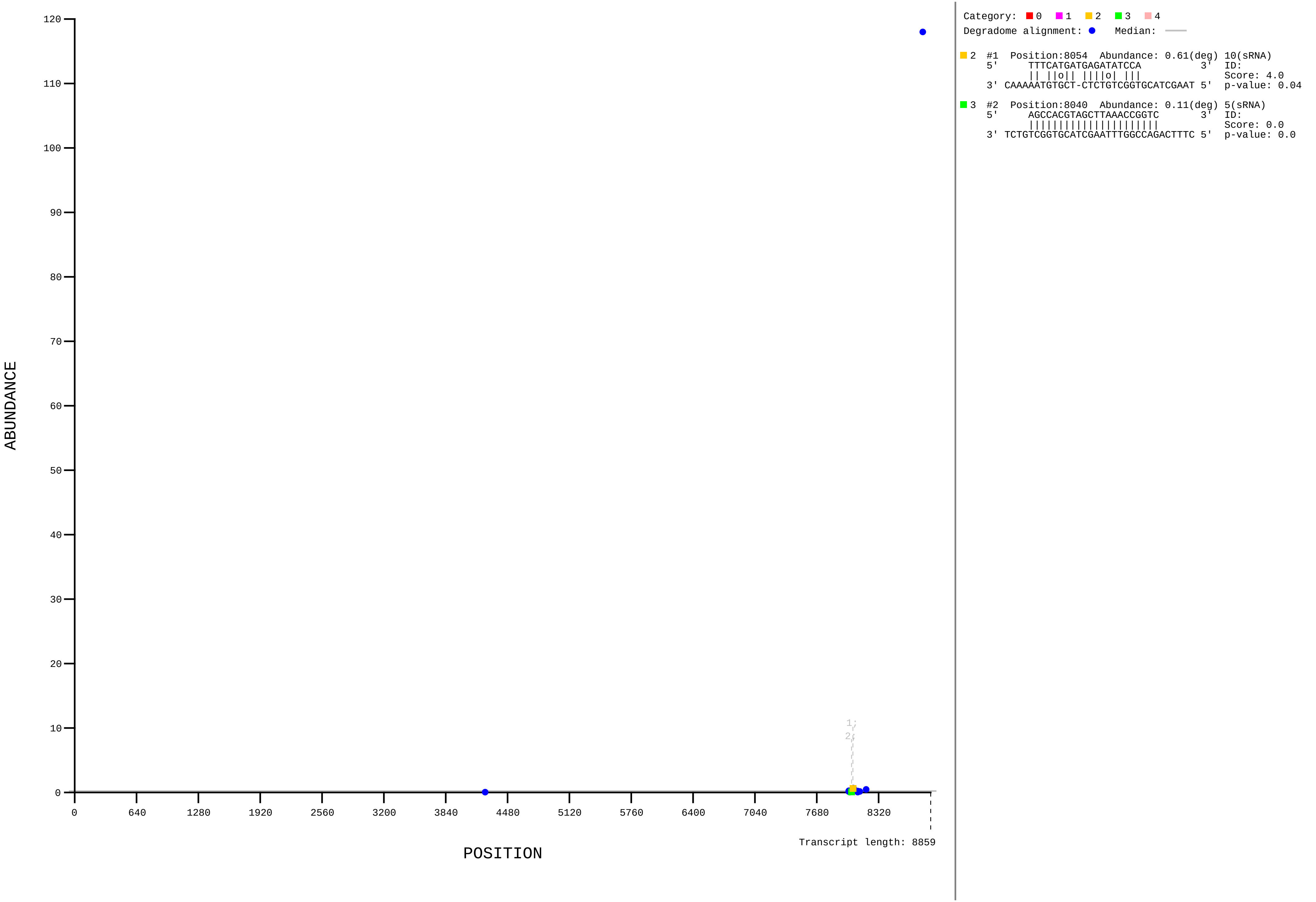

FOXG_17106T0 | Fusarium oxysporum f. sp. lycopersici 4287 peroxidase/catalase HPI (1557 nt) Note(s): (truncated)

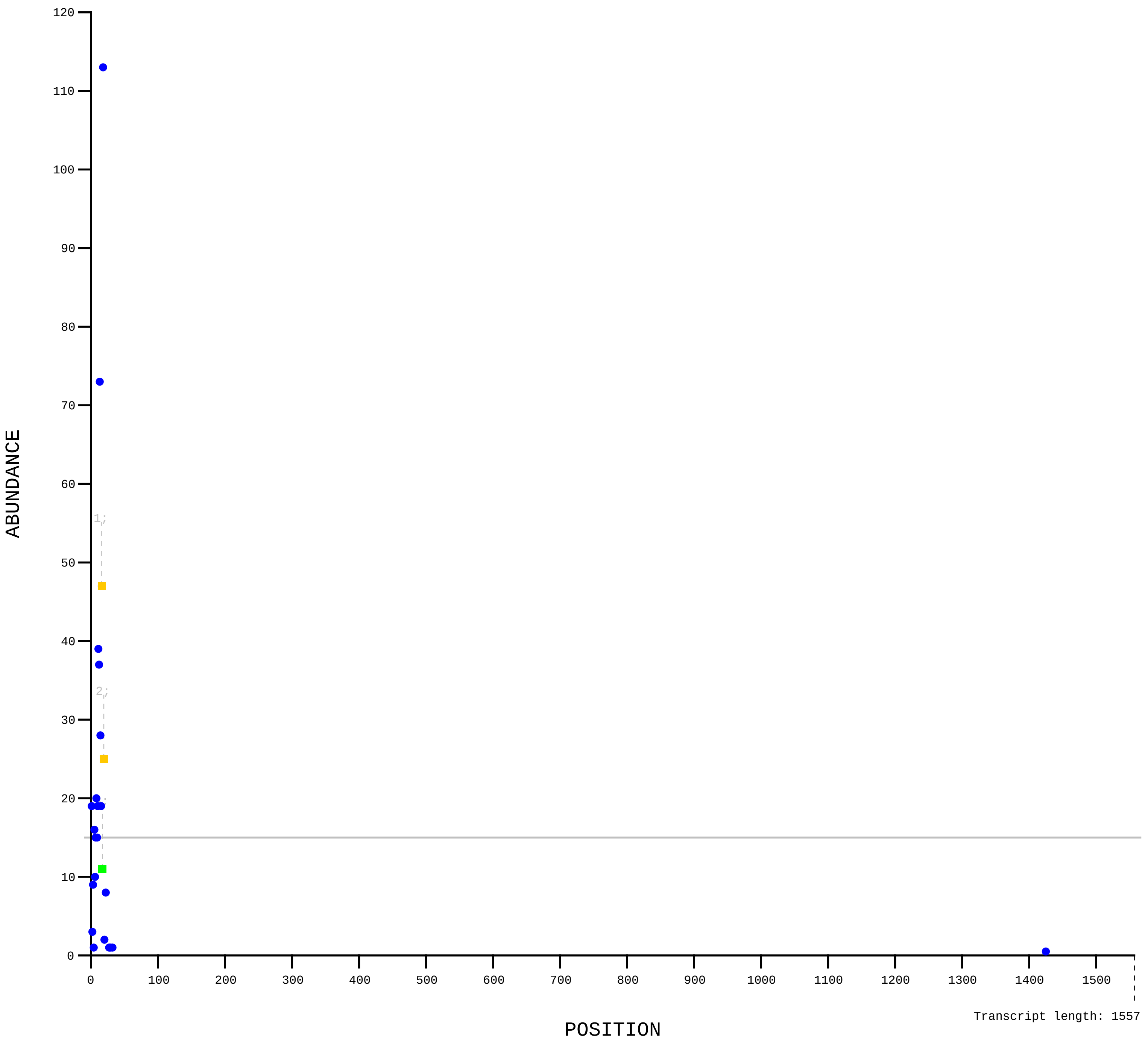

Category: 0 1 2 3 4
Degradome alignment: Median:

```
2  #1  Position:16  Abundance: 47.00(deg) 6(sRNA)
   5'      CGTTCTGGTTCTGAGGTGC          3'  ID:
           |||||||||||||||||||              Score: 0.0
   3' TTCCGCAAGACCAAGACTCCACGGCGGTA    5'  p-value: 0.0

2  #2  Position:19  Abundance: 25.00(deg) 5(sRNA)
   5'      AGGCGTTCTGGTTCTGAGGTGC       3'  ID:
           ||||||||||||||||||||||           Score: 0.0
   3' AGATTCCGCAAGACCAAGACTCCACGGCGGTA 5'  p-value: 0.0

3  #3  Position:17  Abundance: 11.00(deg) 5(sRNA)
   5'      GCGTTCTGGTTCTGAGGTGC         3'  ID:
           ||||||||||||||||||||             Score: 0.0
   3' ATTCCGCAAGACCAAGACTCCACGGCGGTA   5'  p-value: 0.0
```

FOXG_03334T0 | Fusarium oxysporum f. sp. lycopersici 4287 hypothetical protein (384 nt)

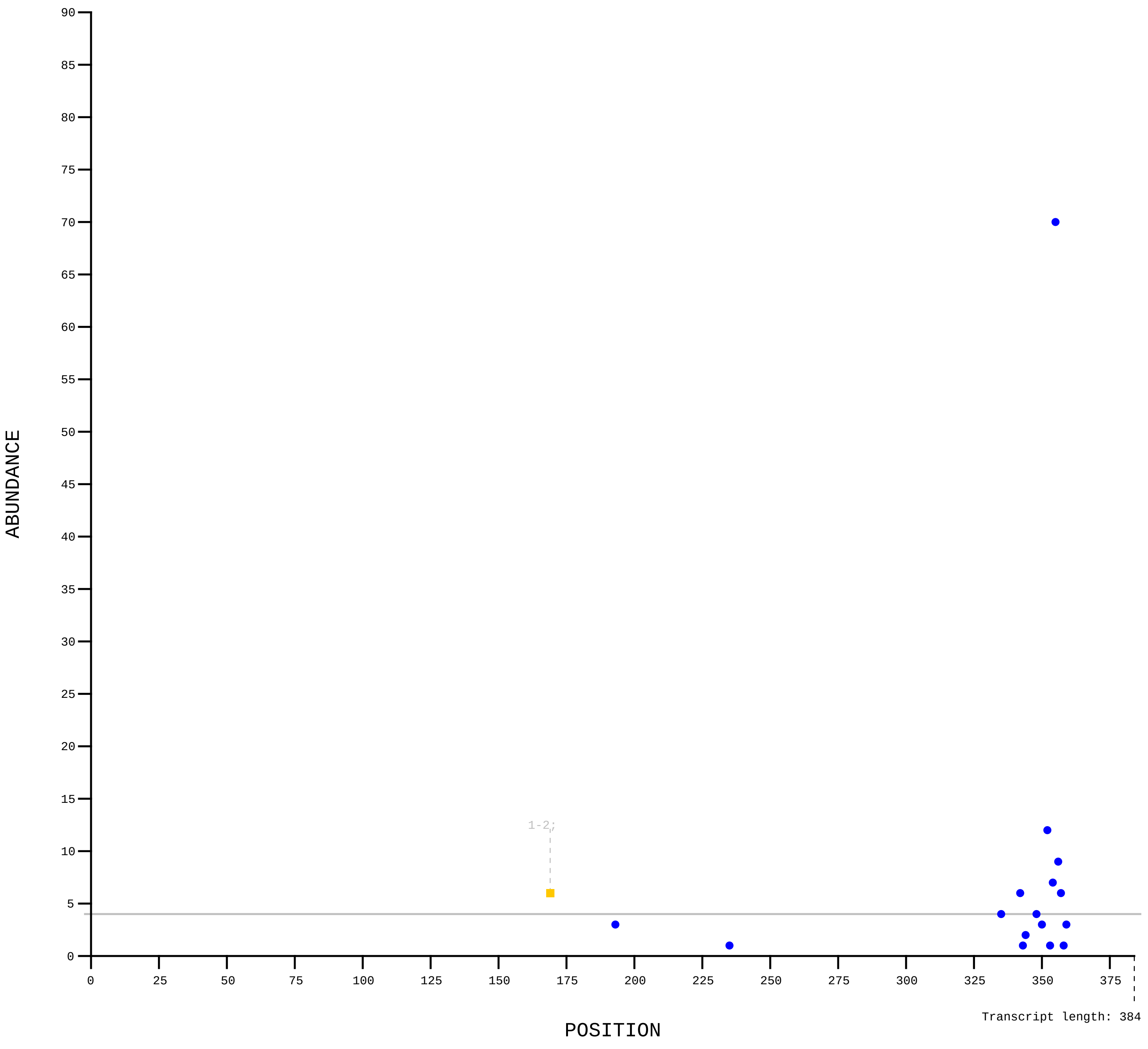

Category: 0 1 2 3 4

Degradome alignment: ● Median:

```
2  #1  Position:169  Abundance: 6.00(deg) 8(sRNA)
   5'     CTCCAGTAGGAGATCTGGC          3'  ID:
          | ||||||||||||| |||              Score: 2.0
   3' CATCGCGGTCATCCTCTAG-CCGTTTCCATCA 5'  p-value: 0.0

2  #2  Position:169  Abundance: 6.00(deg) 6(sRNA)
   5'     CTCCAGTAGGAGATTTGGC          3'  ID:
          | ||||||||||||o | |              Score: 3.5
   3' CATCGCGGTCATCCTCTAGCCGGTTCCATCAT 5'  p-value: 0.03
```

FOXG_03138T0 | Fusarium oxysporum f. sp. lycopersici 4287 ATP-dependent RNA helicase SUB2 (1305 nt)

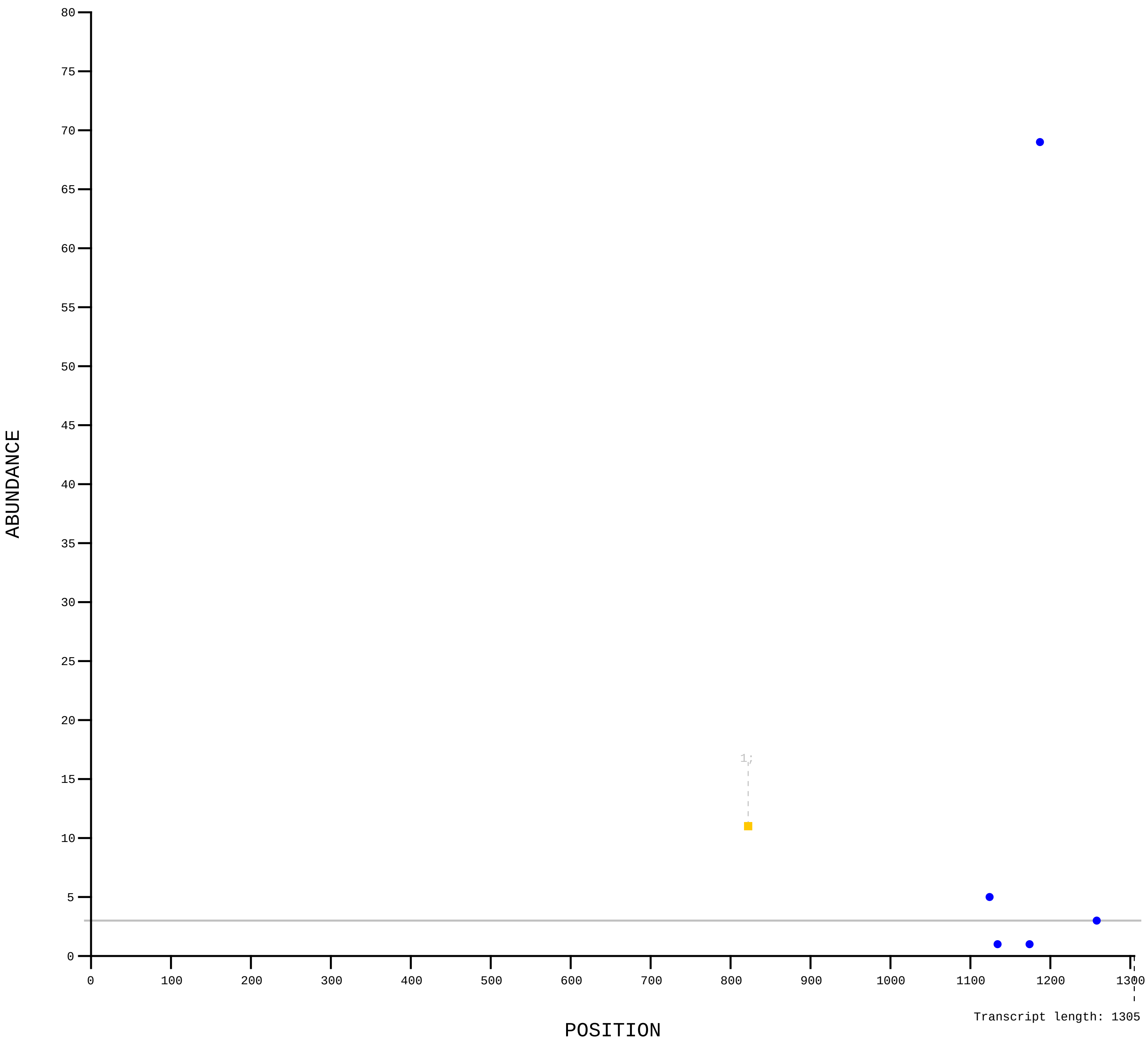

Category: 0 1 2 3 4
Degradome alignment: Median:

```
2  #1  Position:822  Abundance: 11.00(deg) 6(sRNA)
   5'      TTGCAGCTAGATATAGGAAC          3'  ID:
           || ||o|| ||||||| |||              Score: 3.5
   3' AAAGAAGGTTGAACTATATC-TTGACGACTTC 5'  p-value: 0.0
```

FOXG_04188T0 | Fusarium oxysporum f. sp. lycopersici 4287 hypothetical protein (621 nt) Note(s): (truncated)

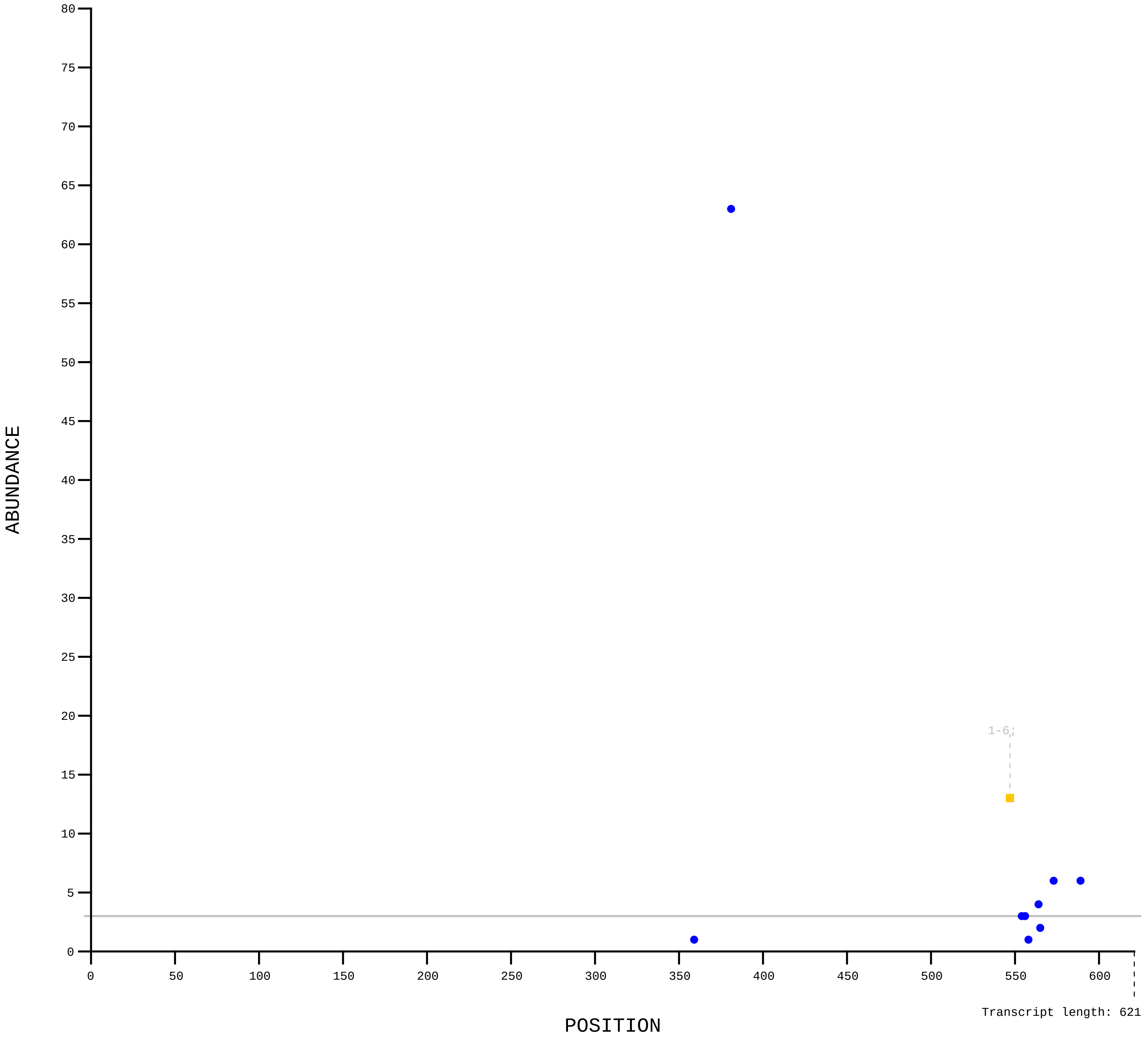


```
Category: 0 1 2 3 4
Degradome alignment:    Median:

2  #1  Position:547  Abundance: 13.00(deg) 190(sRNA)
    5'     TGGAGATTGTGGACTGCGTC         3'  ID:
           | |||  |o||||||||  |||           Score: 3.5
    3' GACGAGCTCGAGCACCTGAC-CAGCCGTCAGT 5'  p-value: 0.02

2  #2  Position:547  Abundance: 13.00(deg) 110(sRNA)
    5'     TGGAGATTGTGGACTGCGT          3'  ID:
           | |||  |o||||||||  ||            Score: 3.5
    3' GACGAGCTCGAGCACCTGAC-CAGCCGTCAGT 5'  p-value: 0.0

2  #3  Position:547  Abundance: 13.00(deg) 63(sRNA)
    5'     TGGAGATTGTGGACTGCGTCG        3'  ID:
           | |||  |o||||||||  ||||          Score: 3.5
    3' GACGAGCTCGAGCACCTGAC-CAGCCGTCAGT 5'  p-value: 0.01

2  #4  Position:547  Abundance: 13.00(deg) 37(sRNA)
    5'     TGGAGATTGTGGACTGCGTCGGC      3'  ID:
           | |||  |o||||||||  ||||||        Score: 3.5
    3' GACGAGCTCGAGCACCTGAC-CAGCCGTCAGT 5'  p-value: 0.0

2  #5  Position:547  Abundance: 13.00(deg) 13(sRNA)
    5'     TGGAGATTGTGGACTGCGTCGG       3'  ID:
           | |||  |o||||||||  |||||         Score: 3.5
    3' GACGAGCTCGAGCACCTGAC-CAGCCGTCAGT 5'  p-value: 0.0

2  #6  Position:547  Abundance: 13.00(deg) 11(sRNA)
    5'     TGGAGATTGTGGACTGCGTCGGCA     3'  ID:
           | |||  |o||||||||  |||||||       Score: 3.5
    3' GACGAGCTCGAGCACCTGAC-CAGCCGTCAGT 5'  p-value: 0.0
```

FOXG_12657T0 | Fusarium oxysporum f. sp. lycopersici 4287 40S ribosomal protein S12 (438 nt)

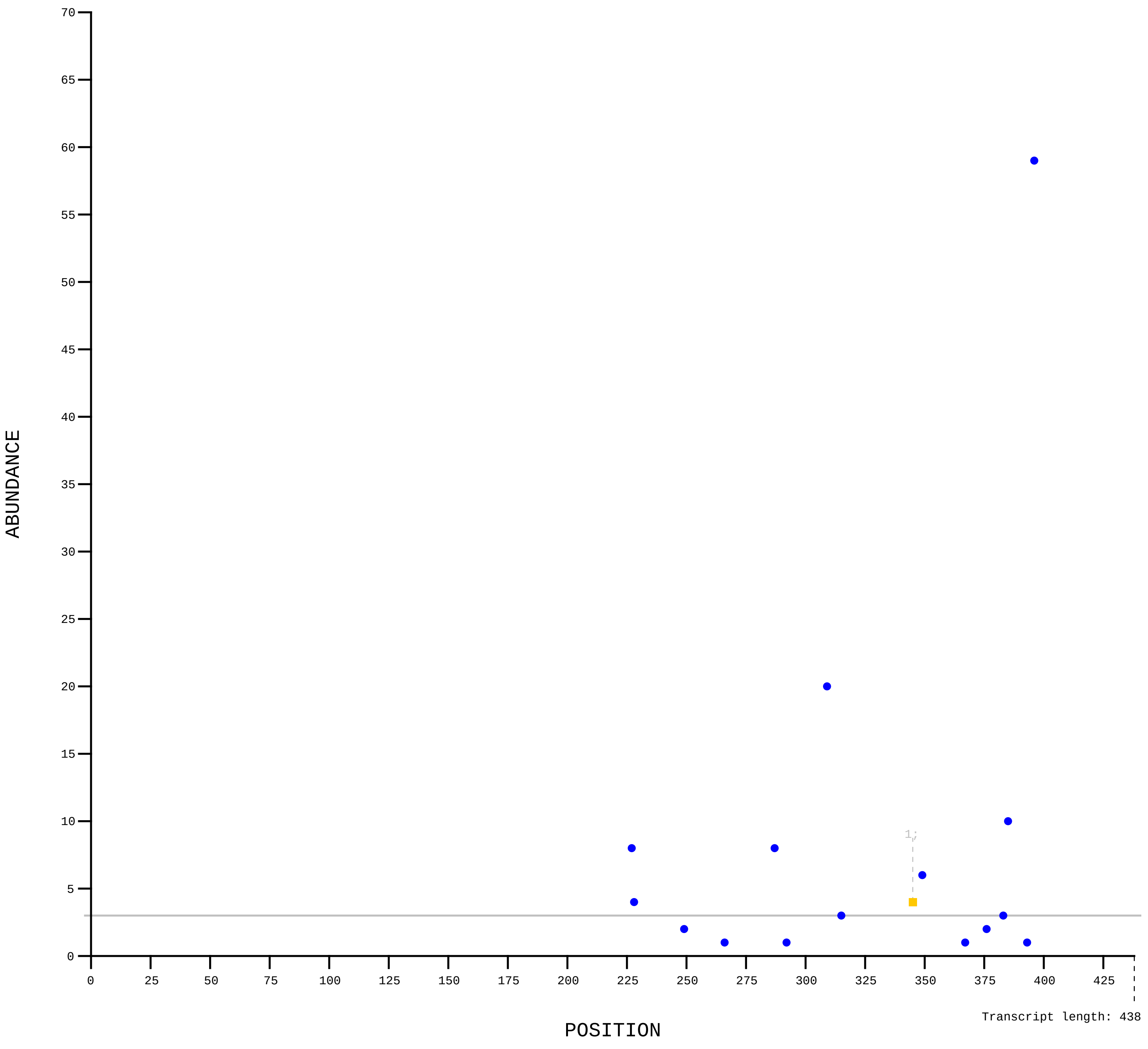

Category: 0 1 2 3 4
Degradome alignment: Median:

```
2  #1  Position:345  Abundance: 4.00(deg) 7(sRNA)
   5'     TGAATGTCGACCTTTGGCA          3'  ID:
            ||||o|| |||o|||||               Score: 4.0
   3' TGGCCCTTATAG-TGGGAACCGGAACGGGAAC 5'  p-value: 0.03
```

FOXG_00815T0 | Fusarium oxysporum f. sp. lycopersici 4287 hypothetical protein (864 nt)

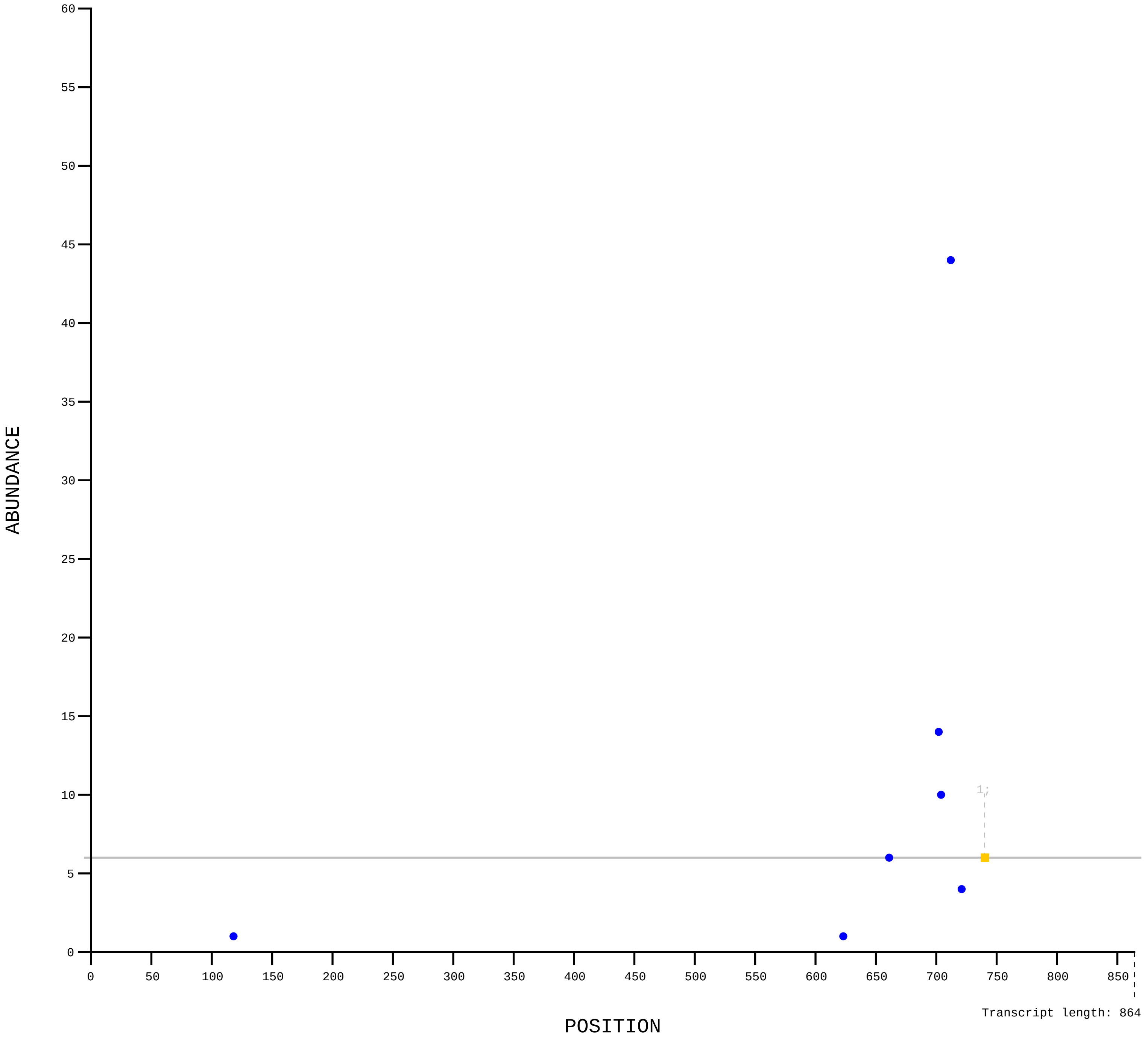

Category: 0 1 2 3 4
Degradome alignment: Median:

```
2  #1  Position:740  Abundance: 6.00(deg) 7(sRNA)
   5'     TGTATTTCTCAAGCCCGTC          3'  ID:
          ||o||o||o|||o| | ||              Score: 4.0
   3' CAACACGTAGAGGGTTTGTGGAGGGAAGCCGA 5'  p-value: 0.04
```

FOXG_11080T0 | Fusarium oxysporum f. sp. lycopersici 4287 mitochondrial ribosomal protein S19 (276 nt)

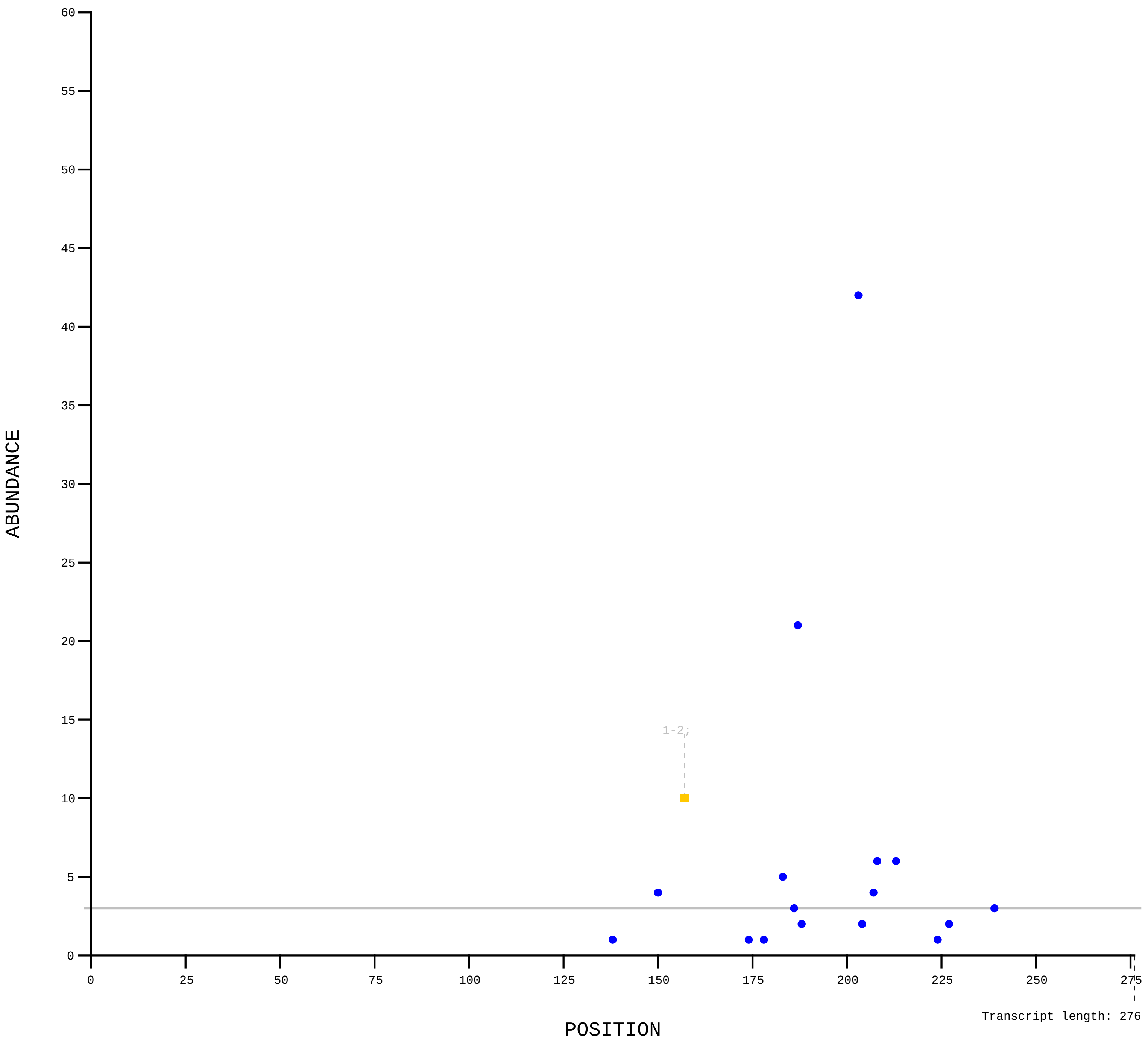


```
Category: ■0 ■1 ■2 ■3 ■4
Degradome alignment: ●   Median: ——

■2  #1  Position:157  Abundance: 10.00(deg) 18(sRNA)
    5'     ACTTGAACTTGAGACGGGA          3'  ID:
             ||o||||||||||| | |             Score: 3.5
    3' ACGTGGAGCTTGAACTCTGGC-TGTTTCAACC 5'  p-value: 0.01

■2  #2  Position:157  Abundance: 10.00(deg) 13(sRNA)
    5'     ACTTGAACTTGAGACGGGAA         3'  ID:
             ||o||||||||||| |o |            Score: 4.0
    3' ACGTGGAGCTTGAACTCTGGCTGTTTCAACCC 5'  p-value: 0.01
```

FOXG_09062T0 | Fusarium oxysporum f. sp. lycopersici 4287 hypothetical protein (1293 nt) Note(s): (truncated)

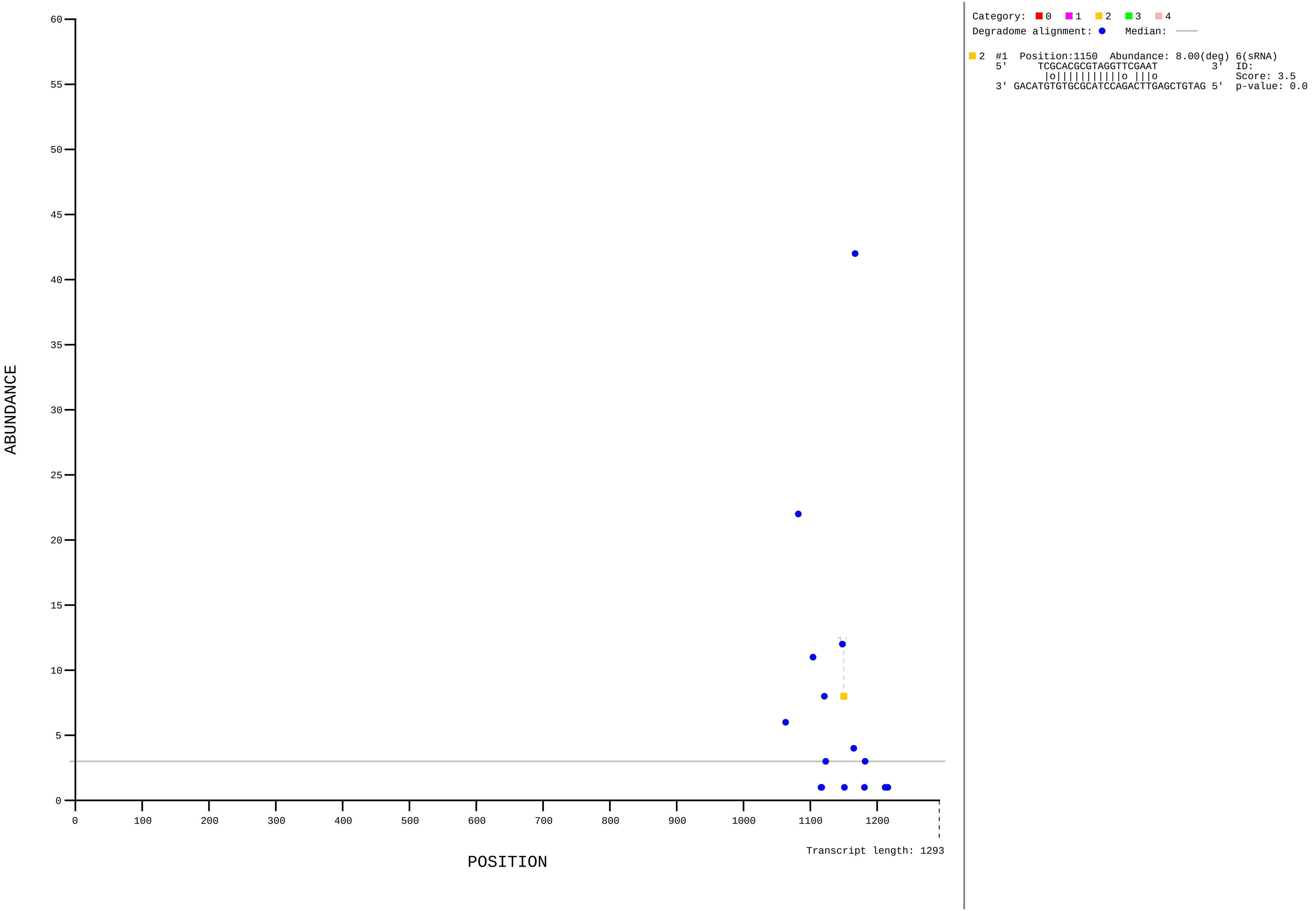

FOXG_12884T0 | Fusarium oxysporum f. sp. lycopersici 4287 ubiquitin fusion protein (465 nt)

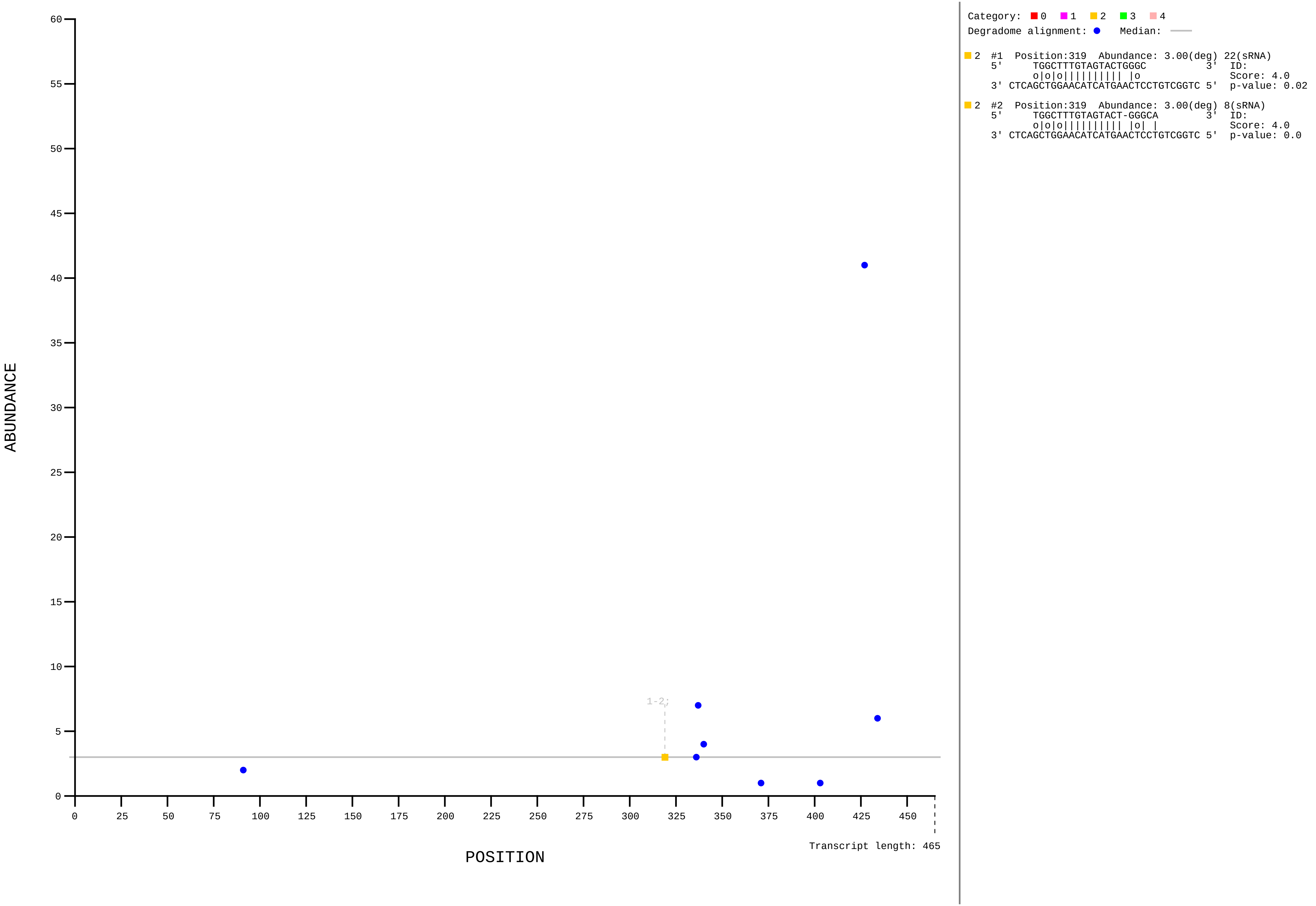

FOXG_02084T0 | Fusarium oxysporum f. sp. lycopersici 4287 hypothetical protein (1386 nt)

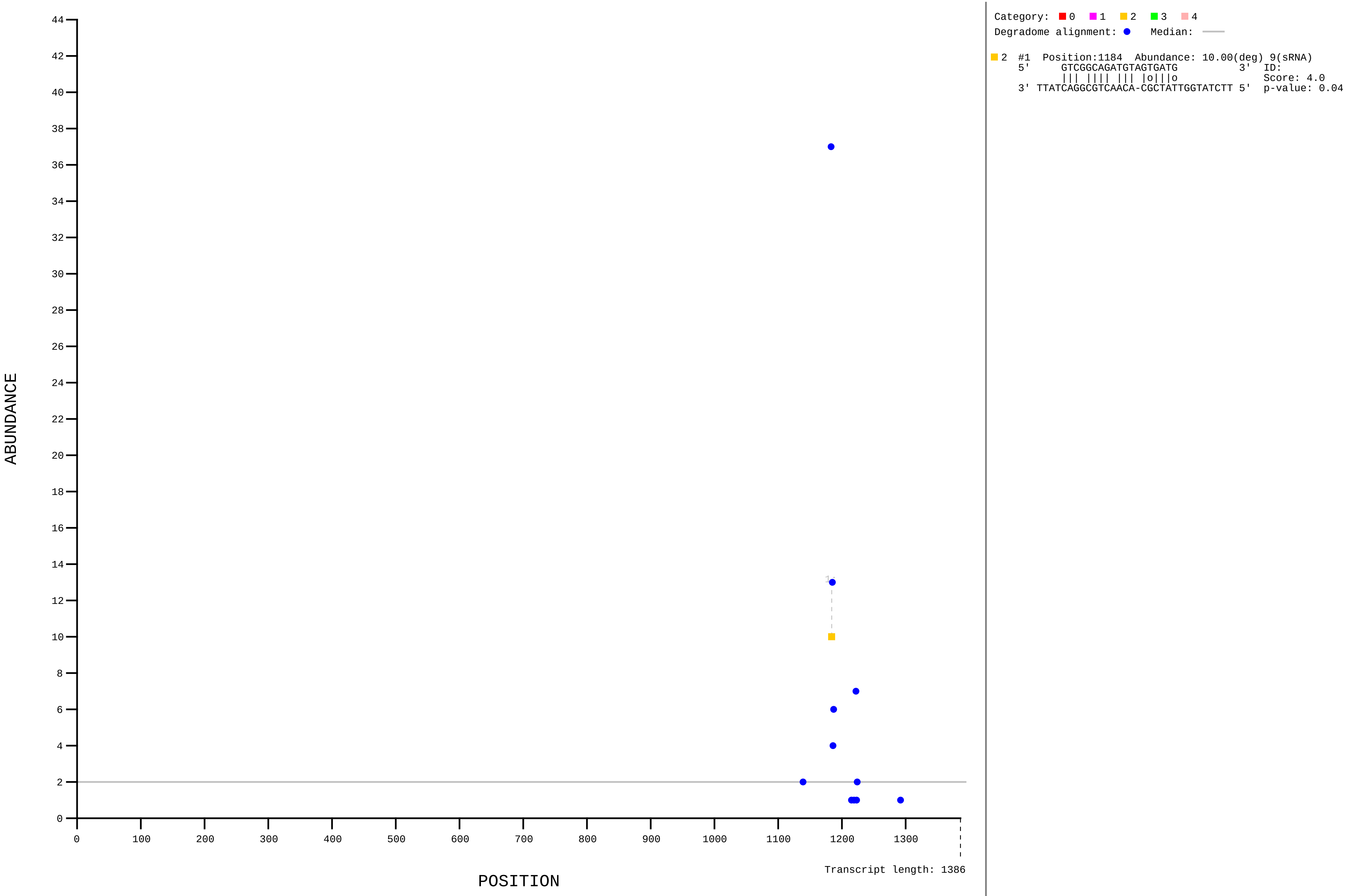

FOXG_09487T0 | Fusarium oxysporum f. sp. lycopersici 4287 hypothetical protein (1170 nt)

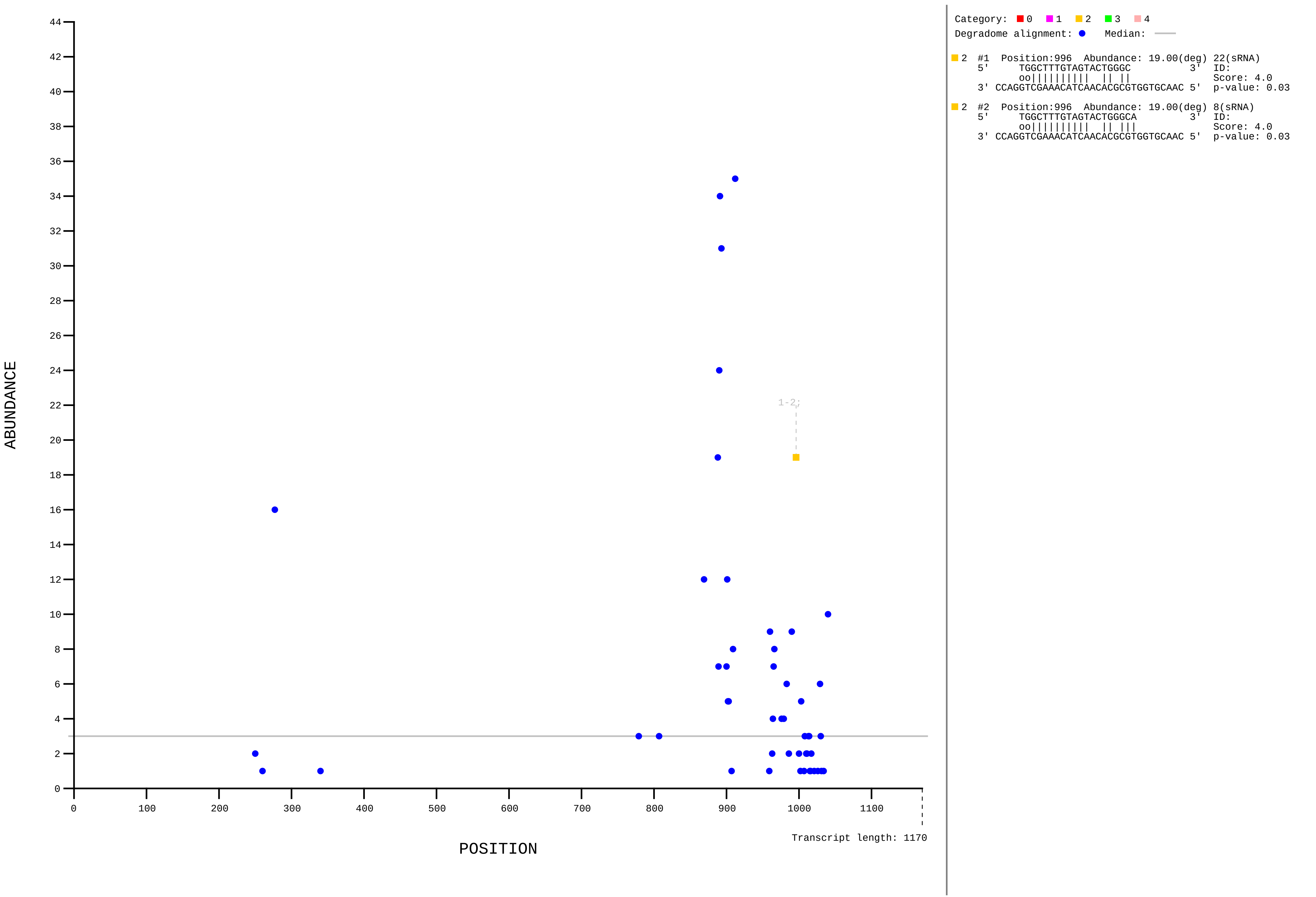

FOXG_05777T0 | Fusarium oxysporum f. sp. lycopersici 4287 hypothetical protein (201 nt)

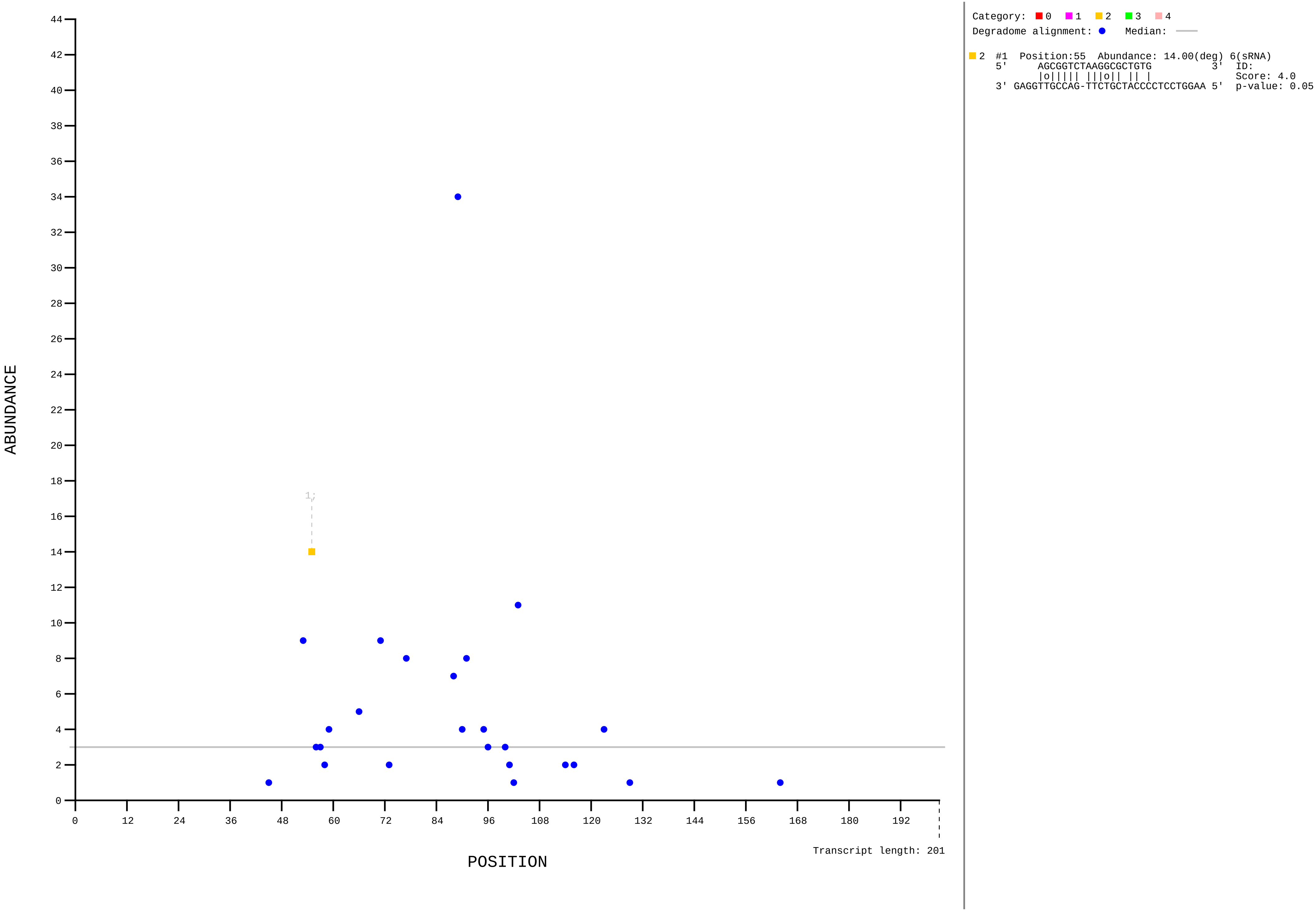

FOXG_02104T0 | Fusarium oxysporum f. sp. lycopersici 4287 hypothetical protein (897 nt)

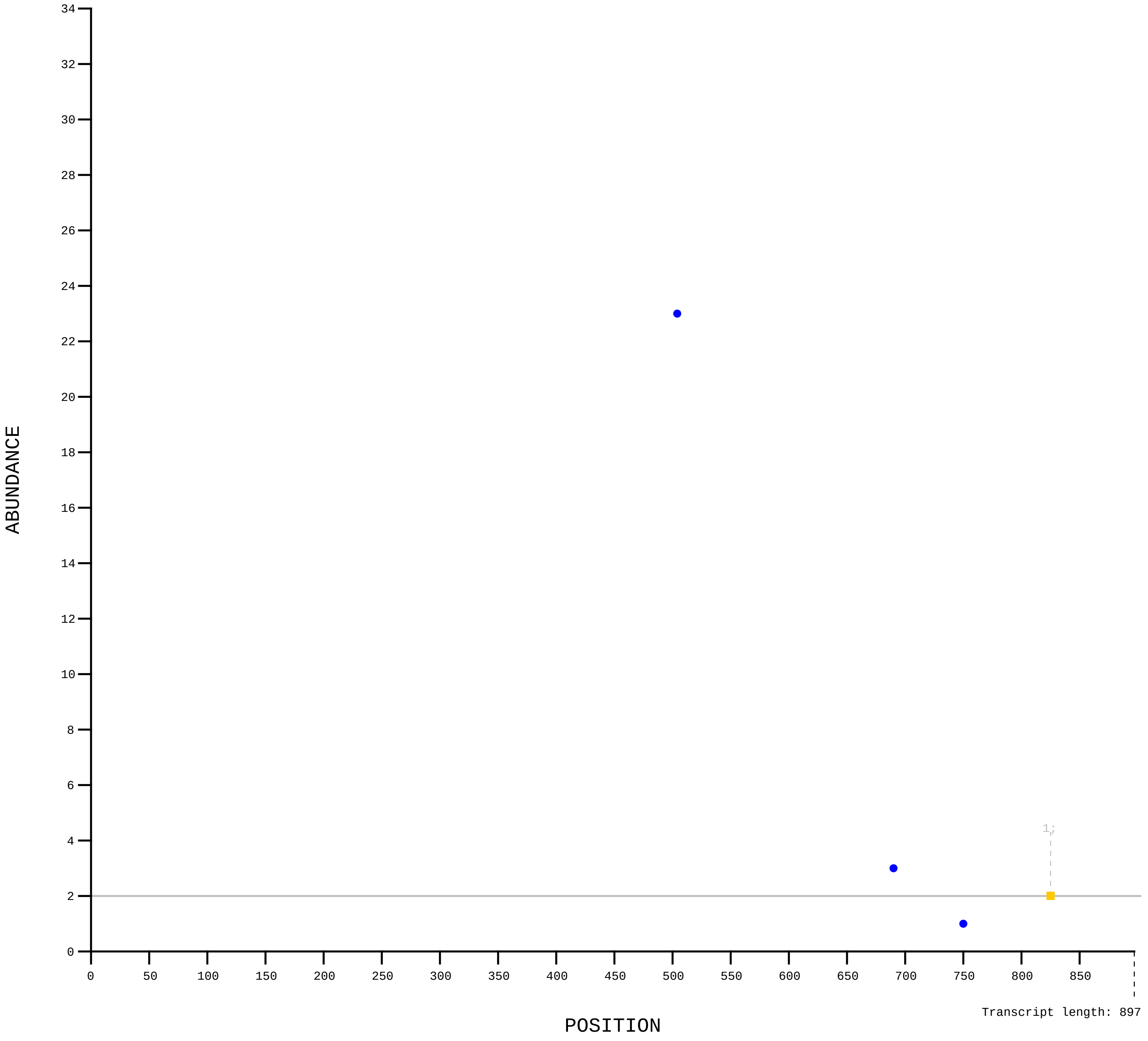

Category: 0 1 2 3 4
Degradome alignment: ● Median:

```
2  #1  Position:825  Abundance: 2.00(deg) 10(sRNA)
   5'      GATCAAGTGTAGTATCTGTTC         3'  ID:
           | |||||o||||| ||||oo|             Score: 3.5
   3' ACAGCGAGTTCGCATCA-AGACGGGGGAACTC 5'  p-value: 0.0
```

FOXG_13714T0 | Fusarium oxysporum f. sp. lycopersici 4287 hypothetical protein (1023 nt)

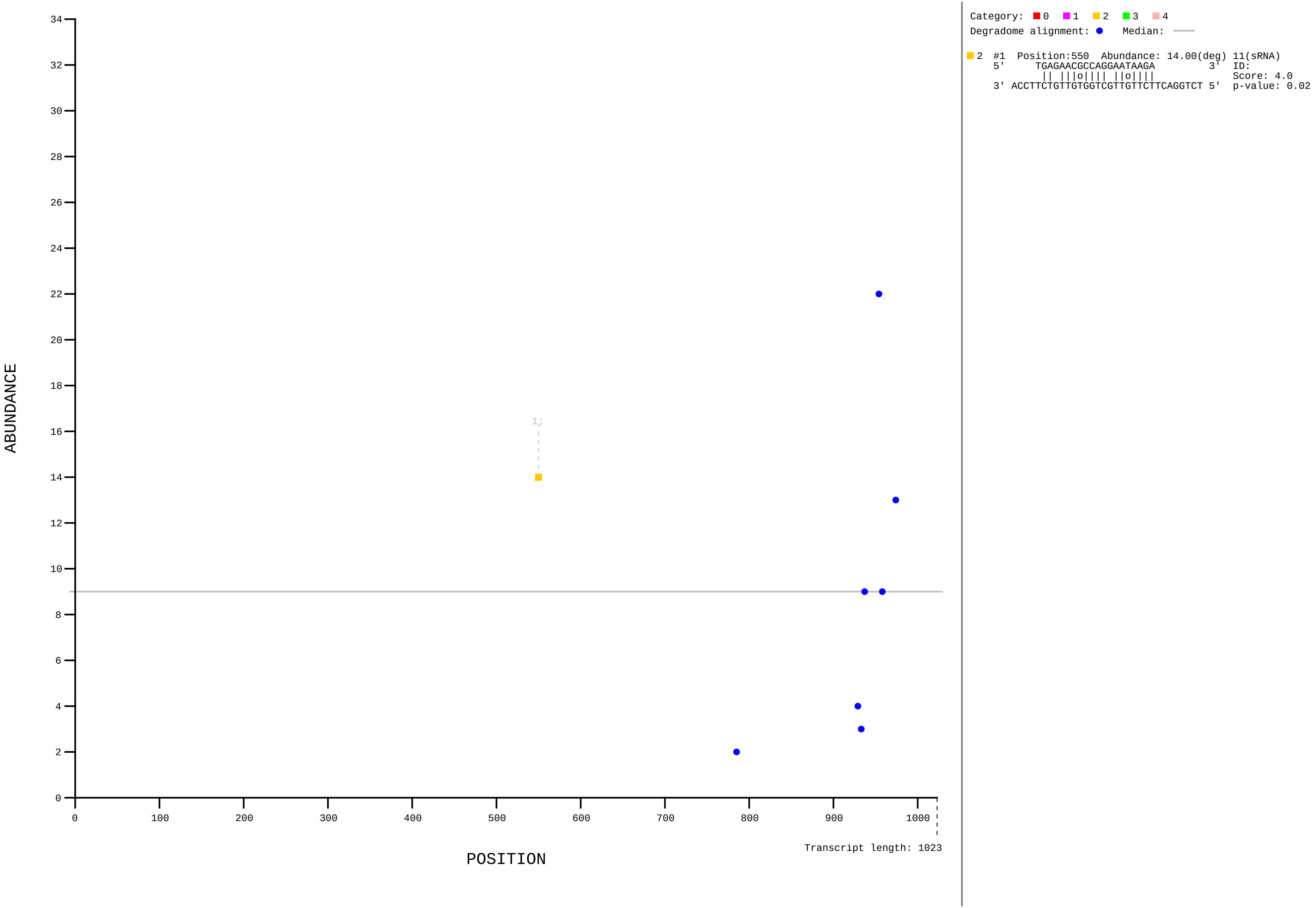

FOXG_00241T0 | Fusarium oxysporum f. sp. lycopersici 4287 endopolyphosphatase (2133 nt)

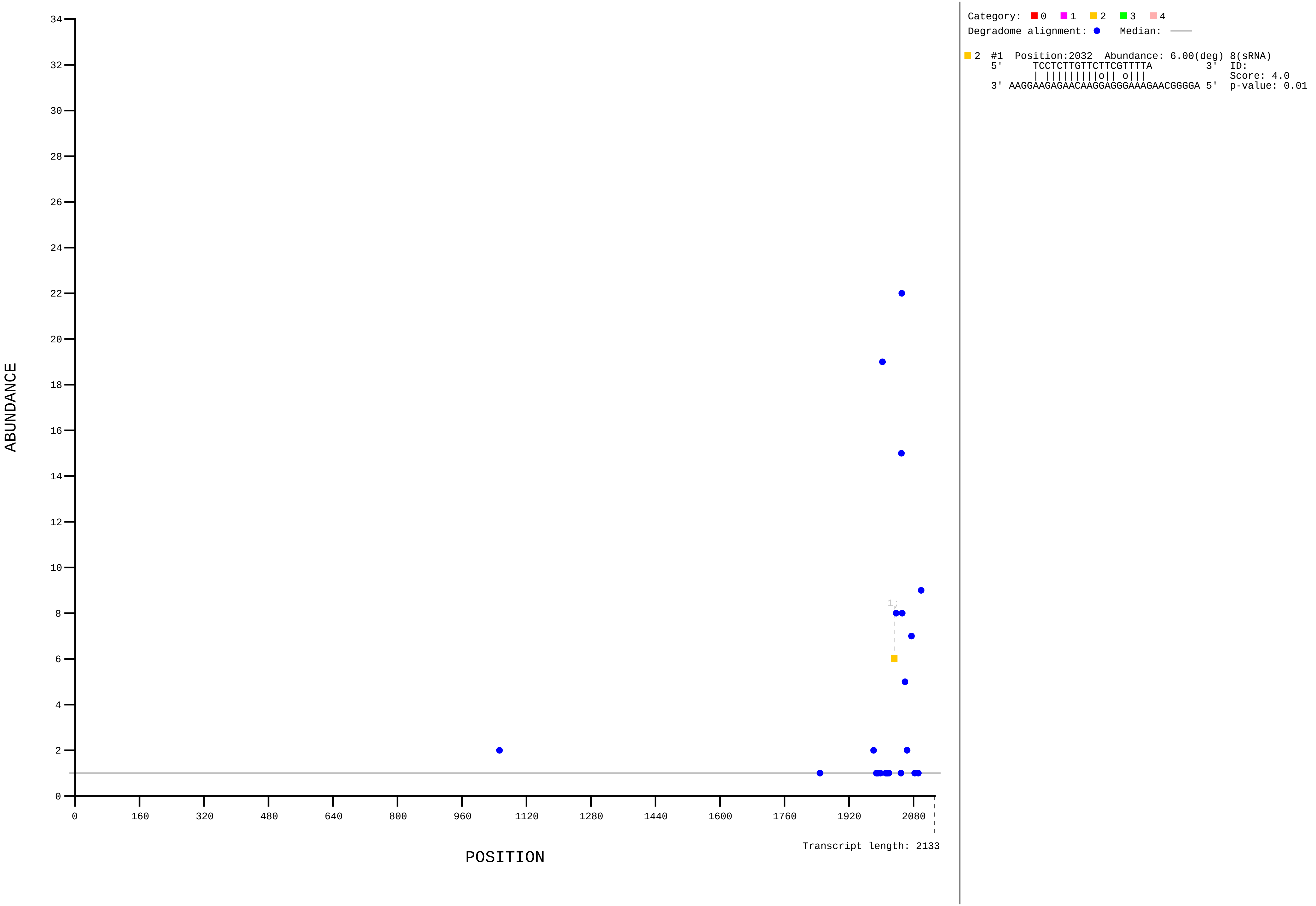

FOXG_08087T0 | Fusarium oxysporum f. sp. lycopersici 4287 hypothetical protein (1173 nt)

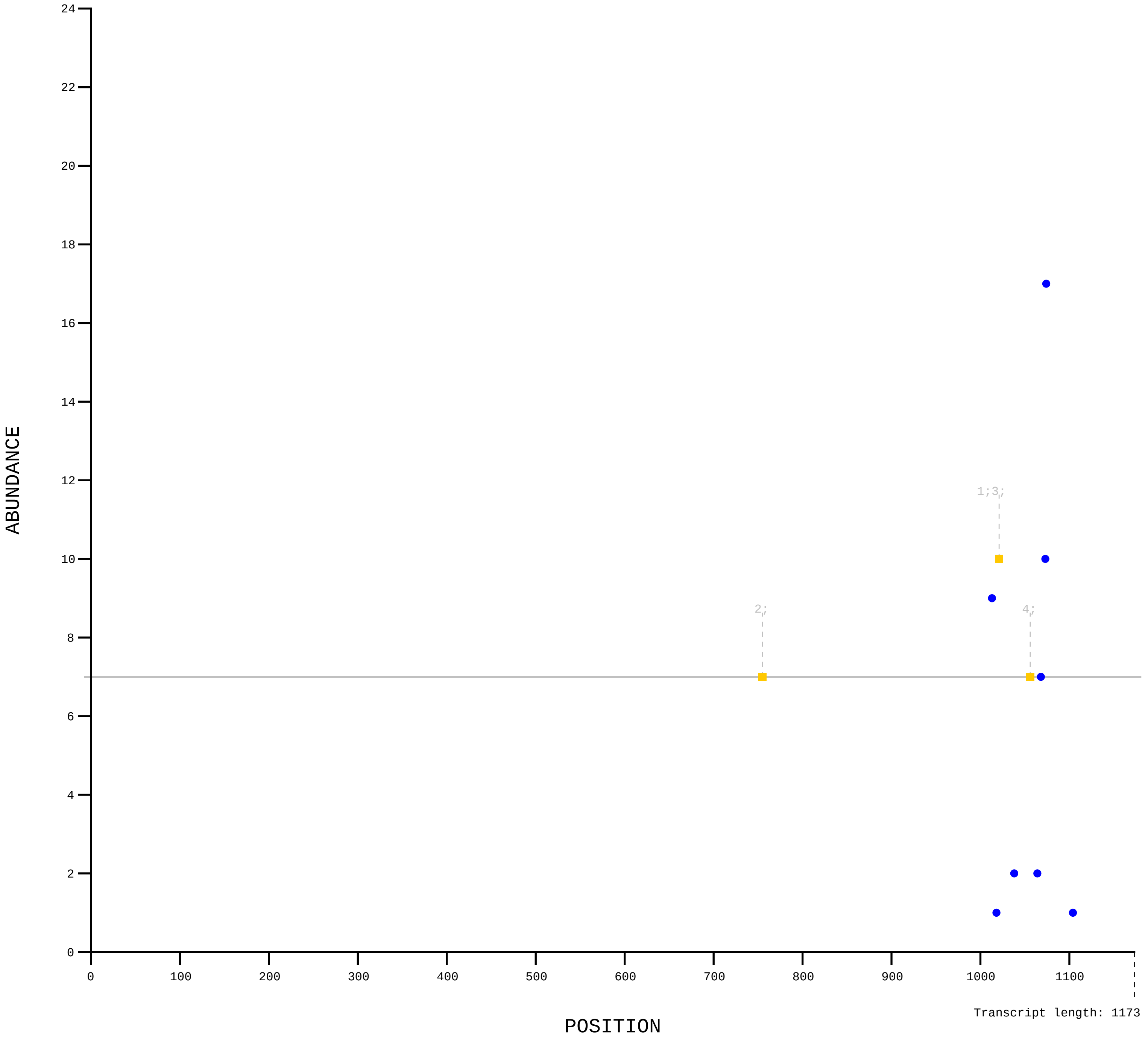


```
Category: ■0 ■1 ■2 ■3 ■4
Degradome alignment: ●   Median: ——

■2  #1  Position:1021  Abundance: 10.00(deg) 30(sRNA)
    5'     TATCGTCAGTCAACTATAGGC        3'  ID:
           o|o||||||||| || | |||            Score: 4.0
    3' AGCCGTGGCAGTCAGTCGA-ACCCGAATAAAA 5'  p-value: 0.0

■2  #2  Position:755  Abundance: 7.00(deg) 25(sRNA)
    5'     TCAGGCTCTGAAACTCGGC          3'  ID:
           ||||||||||| | o|o |              Score: 4.0
    3' GGTAAGTCCGAGACTCTAGGTAGAGCTATCGC 5'  p-value: 0.01

■2  #3  Position:1021  Abundance: 10.00(deg) 14(sRNA)
    5'     TATCGTCAGTCAACTATAGG         3'  ID:
           o|o||||||||| || | ||             Score: 4.0
    3' AGCCGTGGCAGTCAGTCGA-ACCCGAATAAAA 5'  p-value: 0.0

■2  #4  Position:1056  Abundance: 7.00(deg) 13(sRNA)
    5'     TGCAAAAGCCTTCACAGAGG         3'  ID:
           | ||| |||||| |||| ||             Score: 4.0
    3' ACCGA-GTTGTCGGAACTGTCCCCGTCCGAAC 5'  p-value: 0.01
```

FOXG_04890T0 | Fusarium oxysporum f. sp. lycopersici 4287 hypothetical protein (2211 nt)

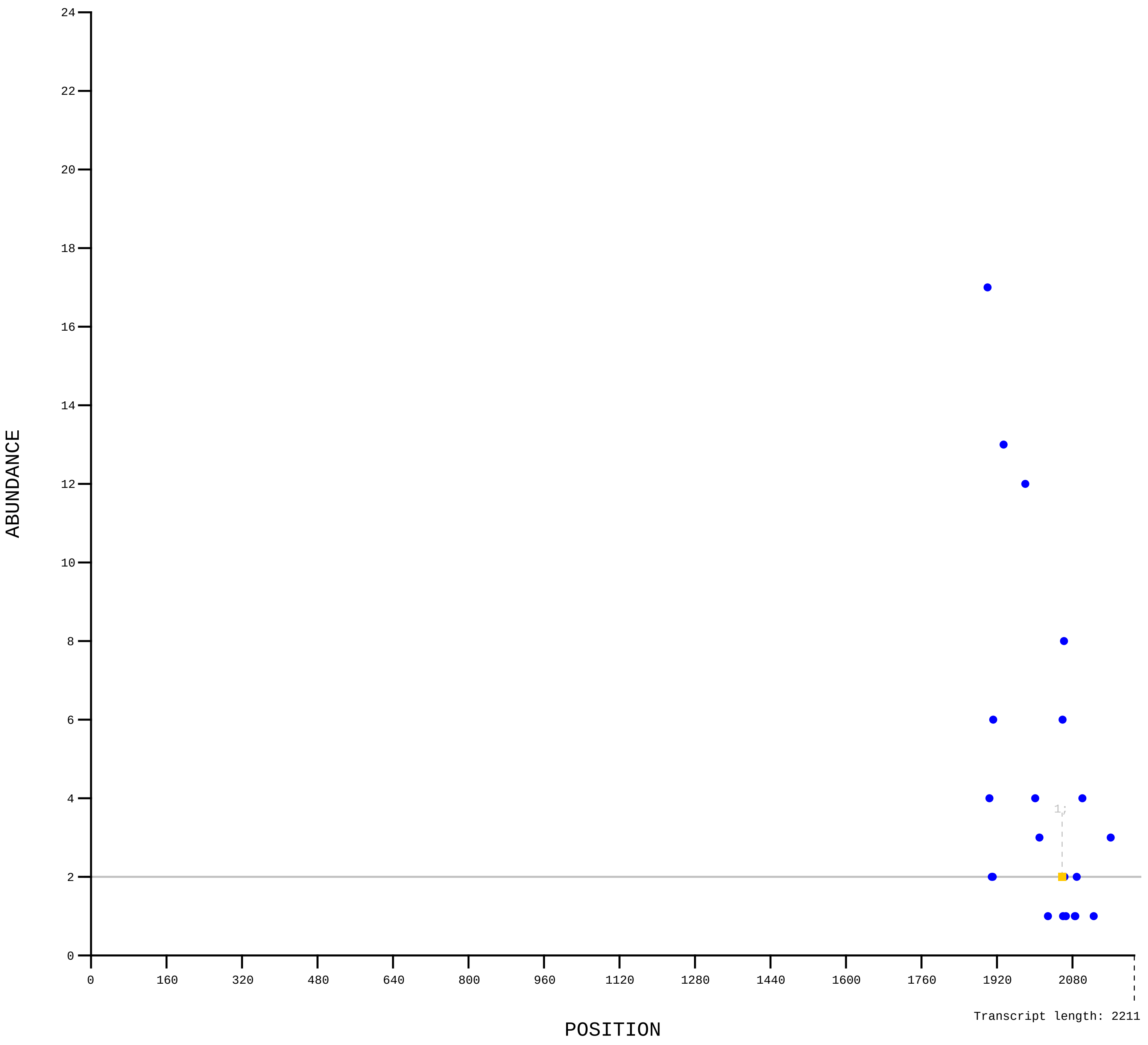

Category: 0 1 2 3 4
Degradome alignment: ● Median: —

```
2  #1  Position:2058  Abundance: 2.00(deg) 13(sRNA)
   5'     CTTTATAGGATC-TGTGGCAG        3'  ID:
          |||o|||| ||| ||o| |||            Score: 4.0
   3' ATACGAAGTATCGTAGGACGCAGTCGAGGACA 5'  p-value: 0.01
```

FOXG_05725T0 | Fusarium oxysporum f. sp. lycopersici 4287 hypothetical protein (1464 nt)

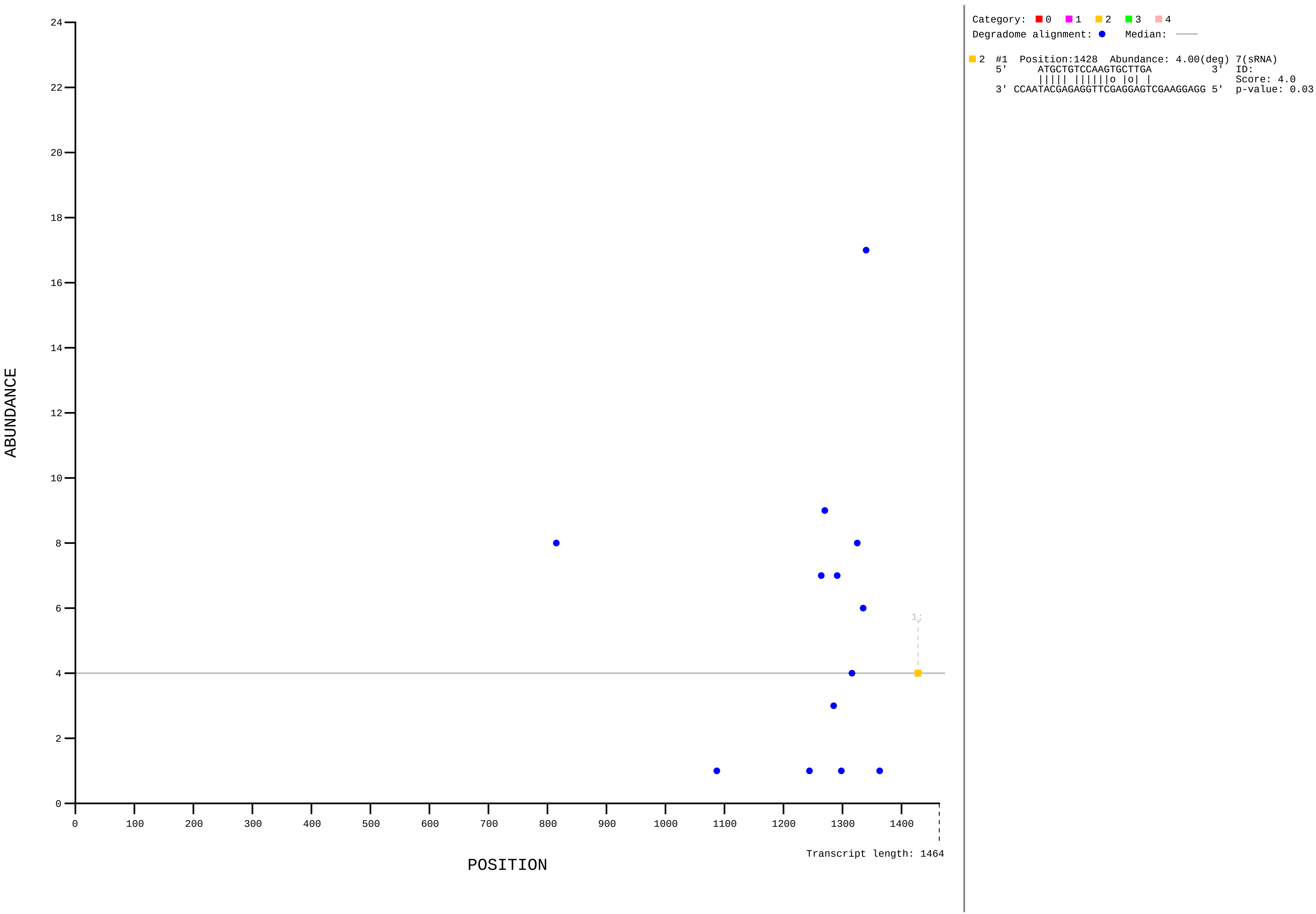

FOXG_08407T0 | Fusarium oxysporum f. sp. lycopersici 4287 glycolipid 2-alpha-mannosyltransferase (truncated)

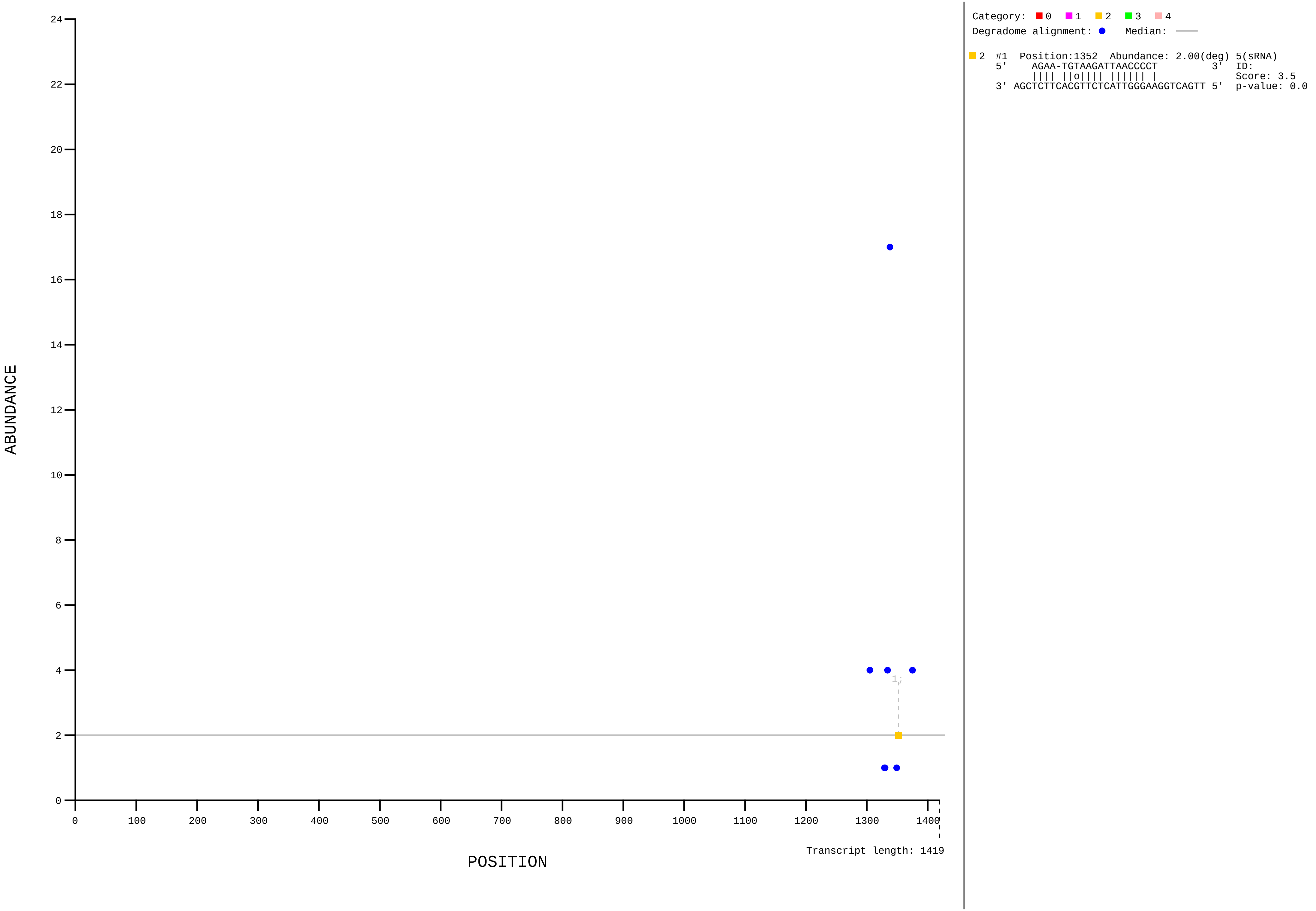

FOXG_16469T0 | Fusarium oxysporum f. sp. lycopersici 4287 hypothetical protein (741 nt)

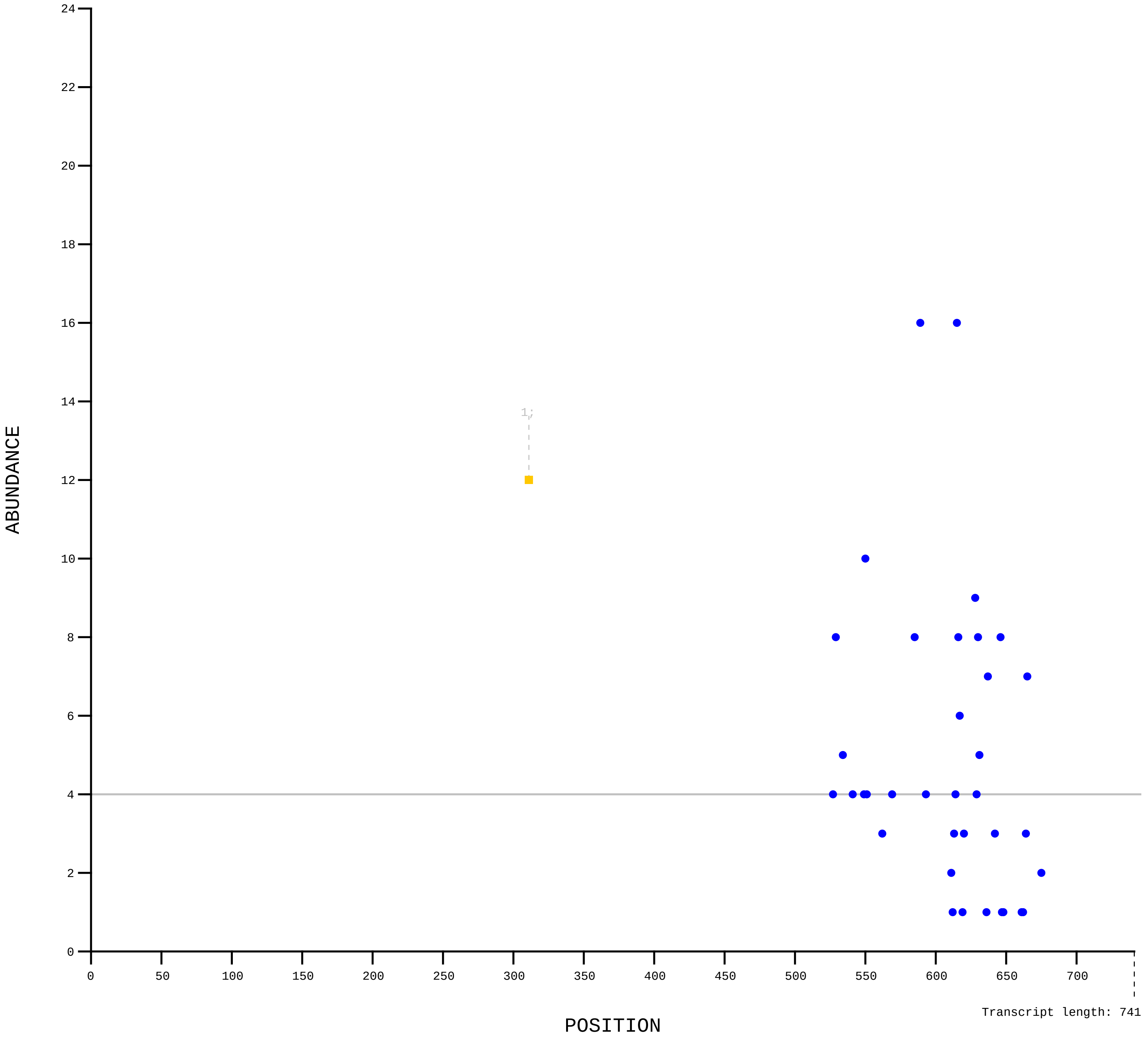


```
Category: 0  1  2  3  4
Degradome alignment:    Median:

2  #1  Position:311  Abundance: 12.00(deg) 11(sRNA)
   5'    AACAGTG-ACTTTAACGGTA          3'  ID:
         ||||o|| ||||o||| | |              Score: 4.0
   3' CCGTTGTTACATGAAGTTGGCTTACGTTGACT 5'  p-value: 0.0
```

FOXG_11955T0 | Fusarium oxysporum f. sp. lycopersici 4287 hypothetical protein (1326 nt)

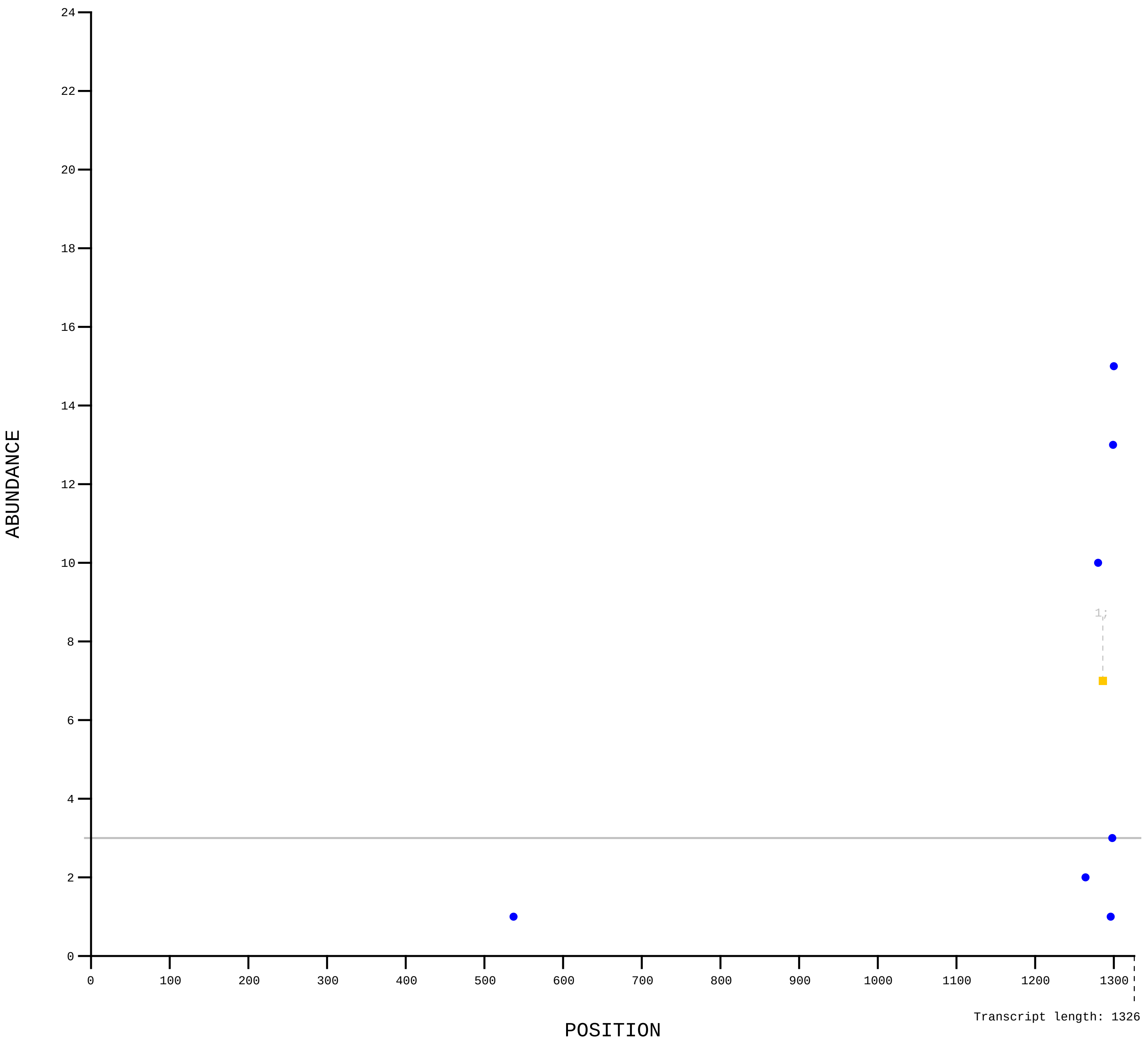


```
Category: 0 1 2 3 4
Degradome alignment:    Median:

2  #1  Position:1286  Abundance: 7.00(deg) 121(sRNA)
   5'     TCGGACACTGATGCTGAGA          3'  ID:
          || ||||||||o||o||||              Score: 2.0
   3' CTTCAG-CTGTGACTGCGGCTCTGACTTCGAC 5'  p-value: 0.0
```

FOXG_01040T0 | Fusarium oxysporum f. sp. lycopersici 4287 hypothetical protein (1470 nt)

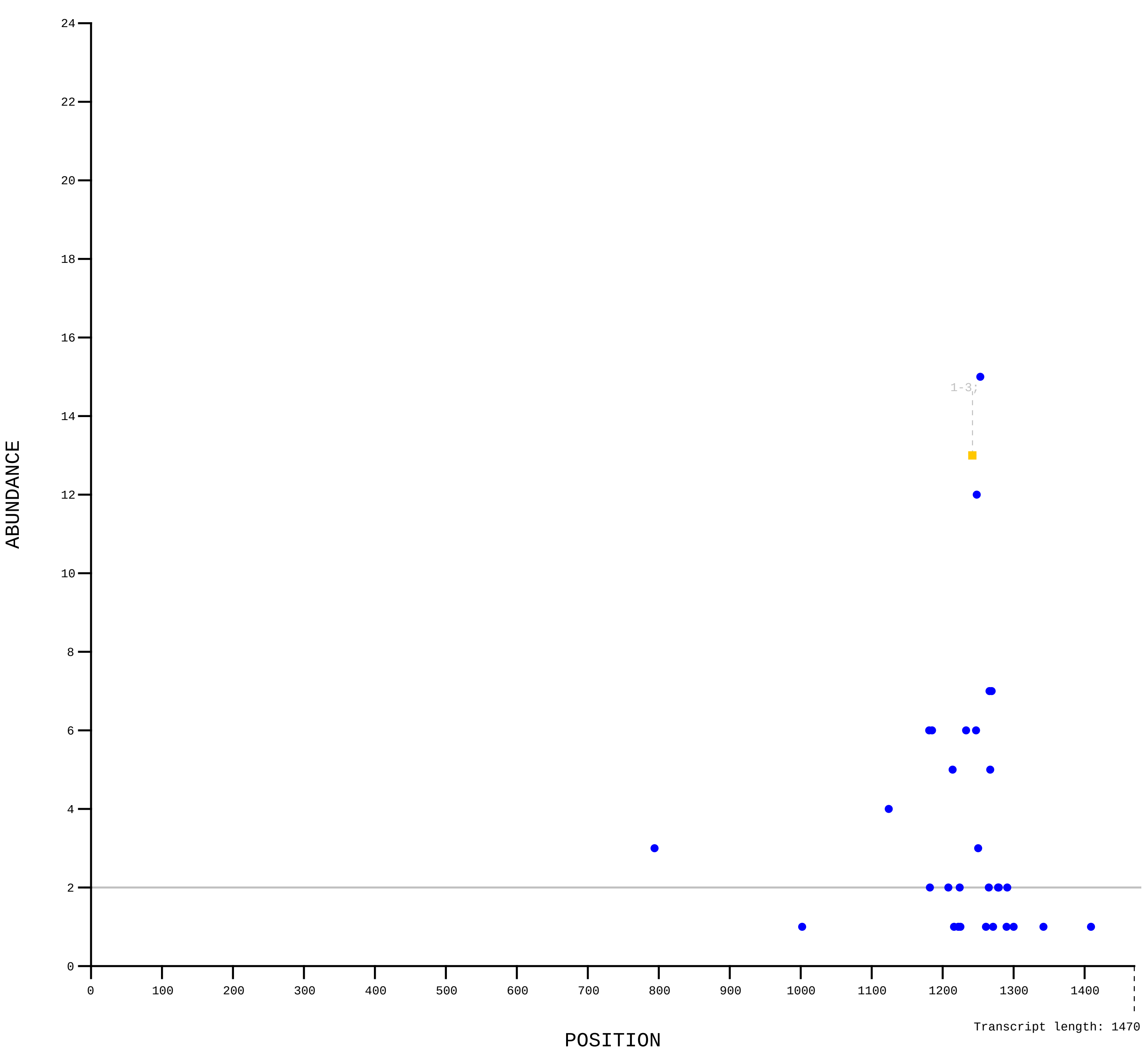


```
Category: 0 1 2 3 4
Degradome alignment:    Median:

2  #1  Position:1242  Abundance: 13.00(deg) 25(sRNA)
   5'     GCGGGTTTAGCTCAGTTGGG         3'  ID:
          || ||||| |||||||| |              Score: 4.0
   3' CCAGCG-CCAAAACGAGTCAAGCATGTTTGCC 5'  p-value: 0.02

2  #2  Position:1242  Abundance: 13.00(deg) 21(sRNA)
   5'     GCGGGTTTAGCTCAGTTGGGA        3'  ID:
          || ||||| |||||||| | |            Score: 4.0
   3' CCAGCG-CCAAAACGAGTCAAGCATGTTTGCC 5'  p-value: 0.0

2  #3  Position:1242  Abundance: 13.00(deg) 16(sRNA)
   5'     GCGGGTTTAGCTCAGTTGG          3'  ID:
          || ||||| |||||||| |              Score: 3.0
   3' CCAGCG-CCAAAACGAGTCAAGCATGTTTGCC 5'  p-value: 0.0
```

FOXG_09855T0 | Fusarium oxysporum f. sp. lycopersici 4287 catechol dioxygenase (957 nt)

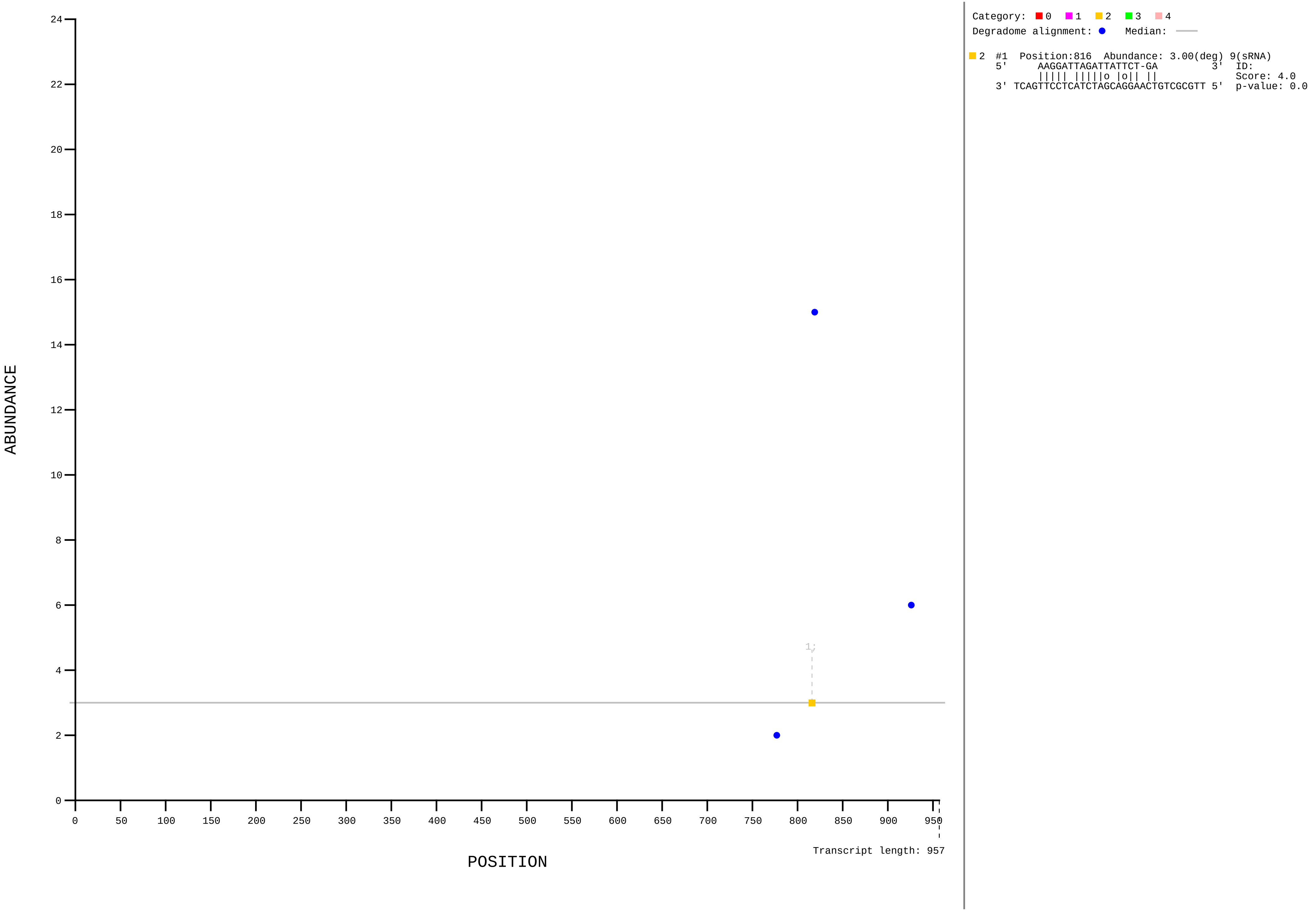

FOXG_01111T0 | Fusarium oxysporum f. sp. lycopersici 4287 peptidyl-prolyl cis-trans isomerase, (truncated)

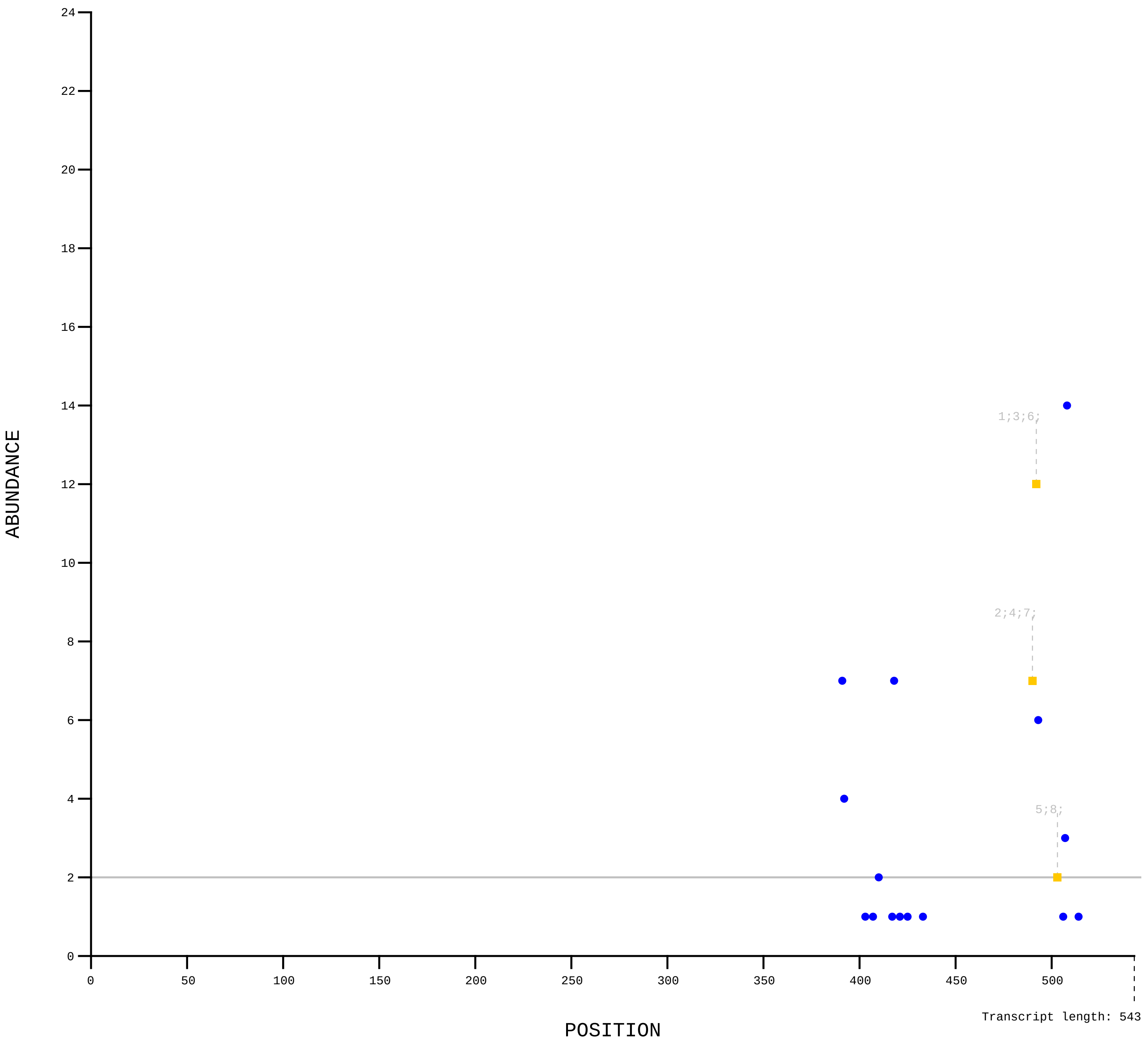


```
Category: 0 1 2 3 4
Degradome alignment: ●   Median: ——

2  #1  Position:492  Abundance: 12.00(deg) 79(sRNA)
   5'      TCTGACAACACCTTTGTCGGA        3'  ID:
           | ||||| |||||o||o||||            Score: 3.0
   3' CATGA-ACTGTCGTGGAGACGGCCTCGGCCAT 5'  p-value: 0.0

2  #2  Position:490  Abundance: 7.00(deg) 48(sRNA)
   5'      TGACAACACCTTTGTCGGATC        3'  ID:
           ||||| |||||o||o|||| |            Score: 3.0
   3' ATGAACTGTCGTGGAGACGGCCTCGGCCATCG 5'  p-value: 0.0

2  #3  Position:492  Abundance: 12.00(deg) 47(sRNA)
   5'      TCTGACAACACCTTTGTCGG         3'  ID:
           | ||||| |||||o||o|||             Score: 3.0
   3' CATGA-ACTGTCGTGGAGACGGCCTCGGCCAT 5'  p-value: 0.0

2  #4  Position:490  Abundance: 7.00(deg) 27(sRNA)
   5'      TGACAACACCTTTGTCGGA          3'  ID:
           ||||| |||||o||o||||              Score: 2.0
   3' ATGAACTGTCGTGGAGACGGCCTCGGCCATCG 5'  p-value: 0.0

2  #5  Position:503  Abundance: 2.00(deg) 16(sRNA)
   5'      GGCTTCTGCTACTTGGC-GC         3'  ID:
            ||||||| ||||||o| ||             Score: 3.5
   3' CCGTGCGAAGACCATGAACTGTCGTGGAGACG 5'  p-value: 0.0

2  #6  Position:492  Abundance: 12.00(deg) 12(sRNA)
   5'      TCTGACAACACCTTTGTCG          3'  ID:
           | ||||| |||||o||o||              Score: 3.0
   3' CATGA-ACTGTCGTGGAGACGGCCTCGGCCAT 5'  p-value: 0.01

2  #7  Position:490  Abundance: 7.00(deg) 5(sRNA)
   5'      TGACAACACCTTTGTCGGAT         3'  ID:
           ||||| |||||o||o||||              Score: 3.0
   3' ATGAACTGTCGTGGAGACGGCCTCGGCCATCG 5'  p-value: 0.0

2  #8  Position:503  Abundance: 2.00(deg) 5(sRNA)
   5'      GGCTTCTGCTACTTGGC-GCA        3'  ID:
            ||||||| ||||||o| |||            Score: 3.5
   3' CCGTGCGAAGACCATGAACTGTCGTGGAGACG 5'  p-value: 0.02
```

FOXG_08367T0 | Fusarium oxysporum f. sp. lycopersici 4287 hypothetical protein (1770 nt)

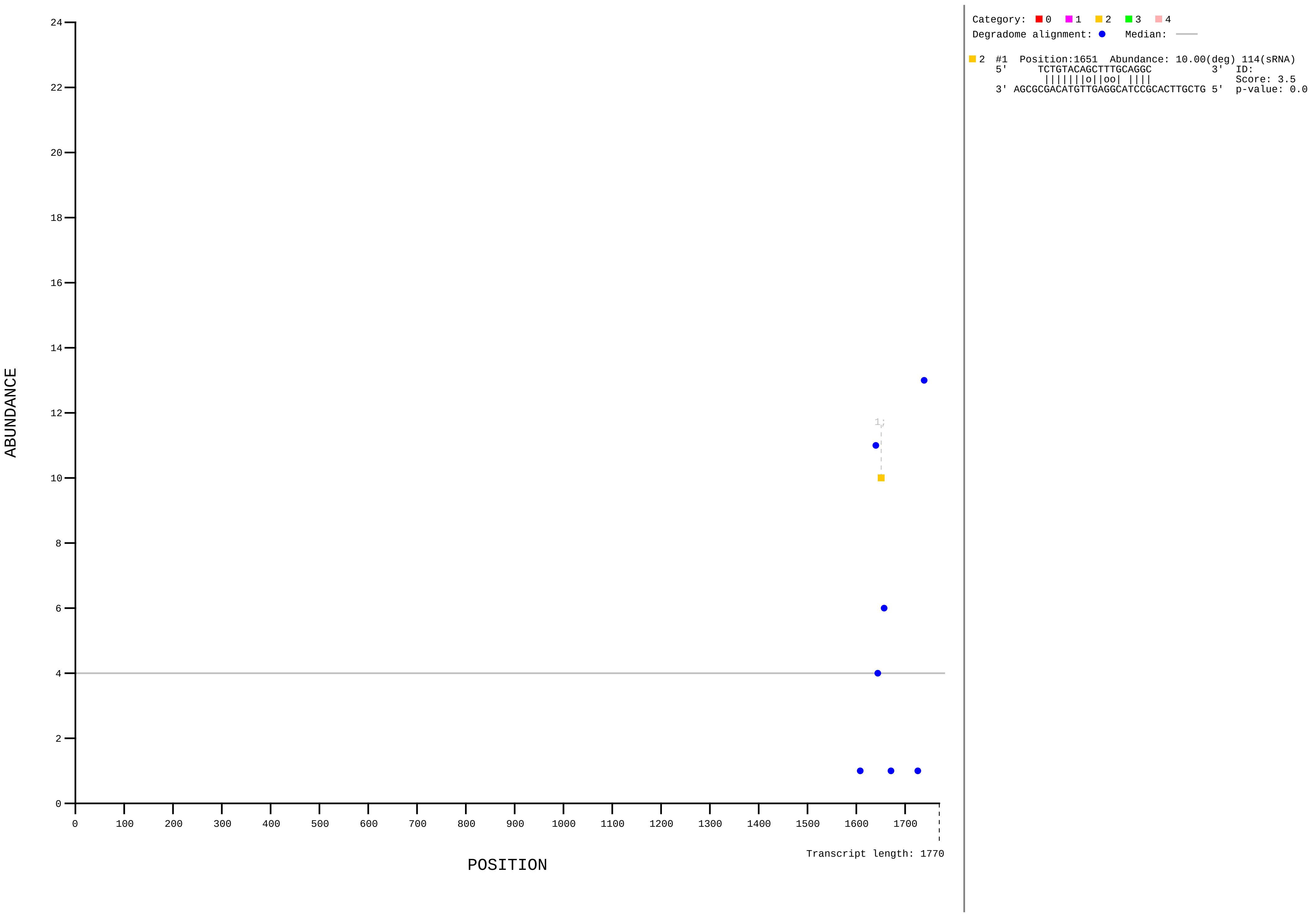

FOXG_00795T0 | Fusarium oxysporum f. sp. lycopersici 4287 heat shock 70 kDa protein (1959 nt)

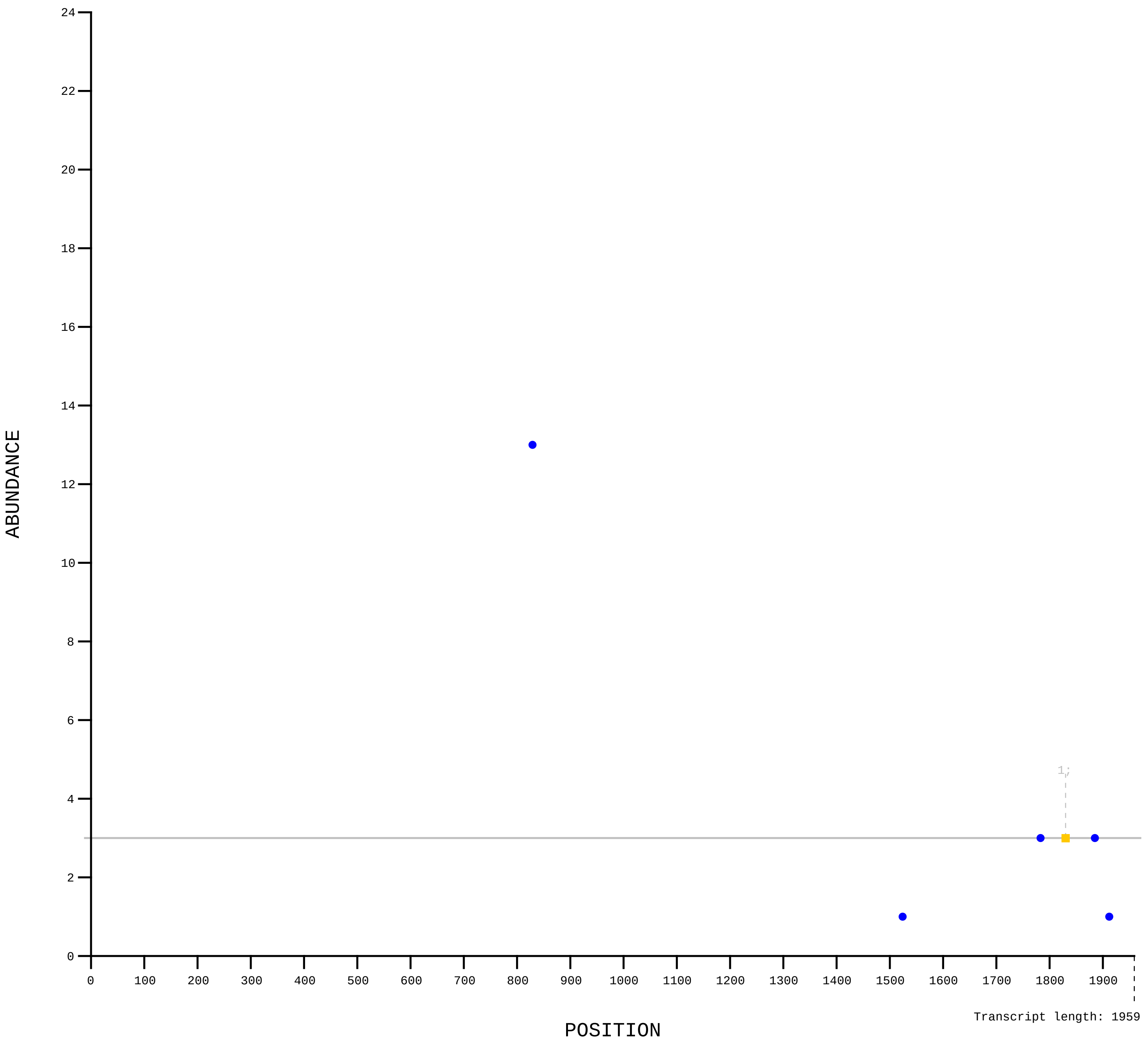

Category: 0 1 2 3 4

Degradome alignment: ● Median:

```
2  #1  Position:1830  Abundance: 3.00(deg) 16(sRNA)
   5'     ATCAGTTCGGTAGAACTTCACCG      3'  ID:
          |o|||o|| ||||||||||| |o          Score: 3.5
   3' GTGGTGGTCGAGGCATCTTGAAGTAGTACTAC 5'  p-value: 0.0
```

FOXG_01511T0 | Fusarium oxysporum f. sp. lycopersici 4287 ABC1 protein (2181 nt)

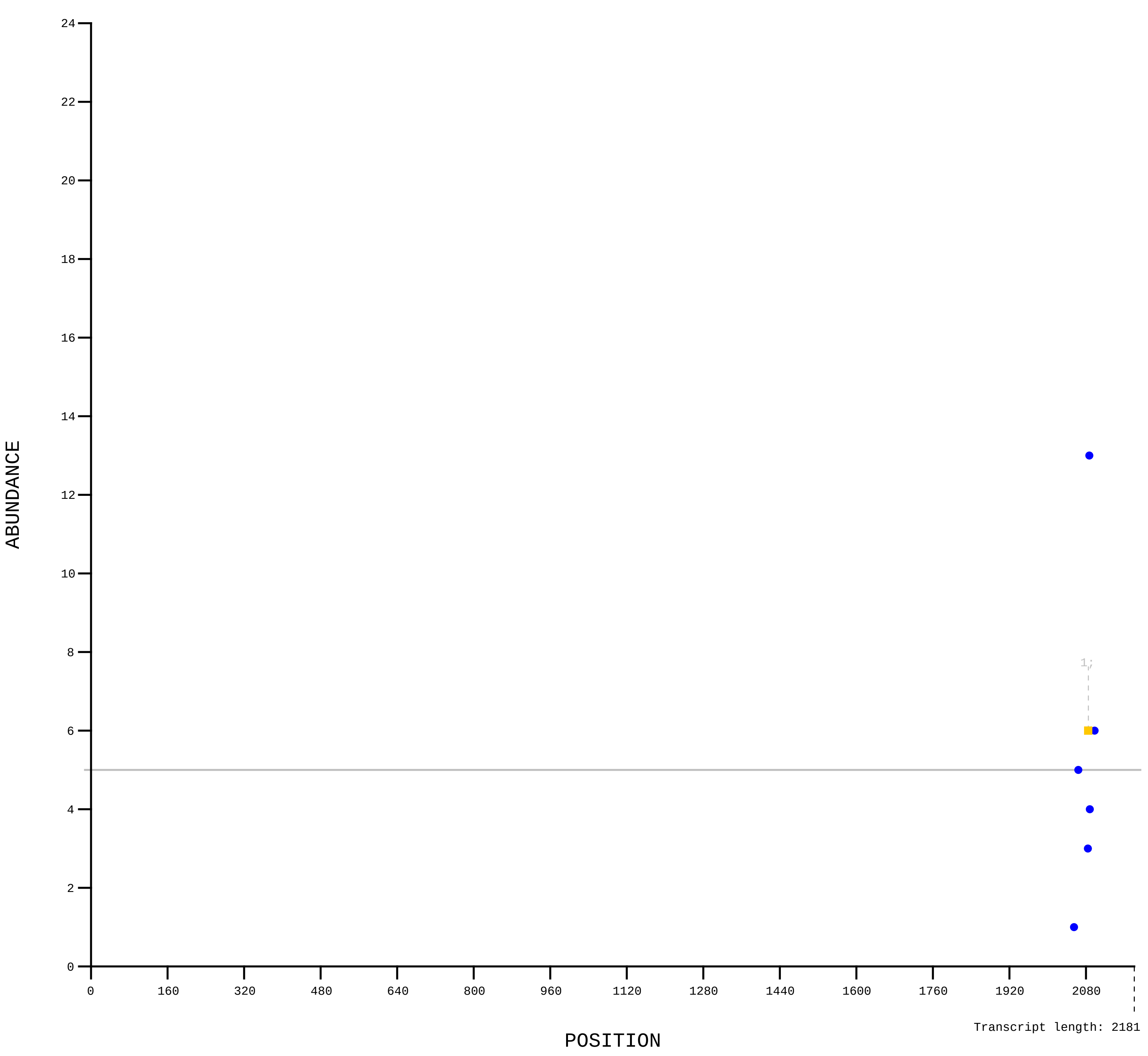

Category: 0 1 2 3 4
Degradome alignment: Median:

```
2  #1  Position:2085  Abundance: 6.00(deg) 7(sRNA)
   5'    ACG-ATCGCTAAATTGTCGG          3'  ID:
         ||| |o|||||| o| ||||              Score: 4.0
   3' CCTTGCGTGGCGATTCGAAAGCCACGTCTGAC 5'  p-value: 0.02
```

FOXG_10059T0 | Fusarium oxysporum f. sp. lycopersici 4287 phosphate transporter (1545 nt) Note(s): At (truncated)

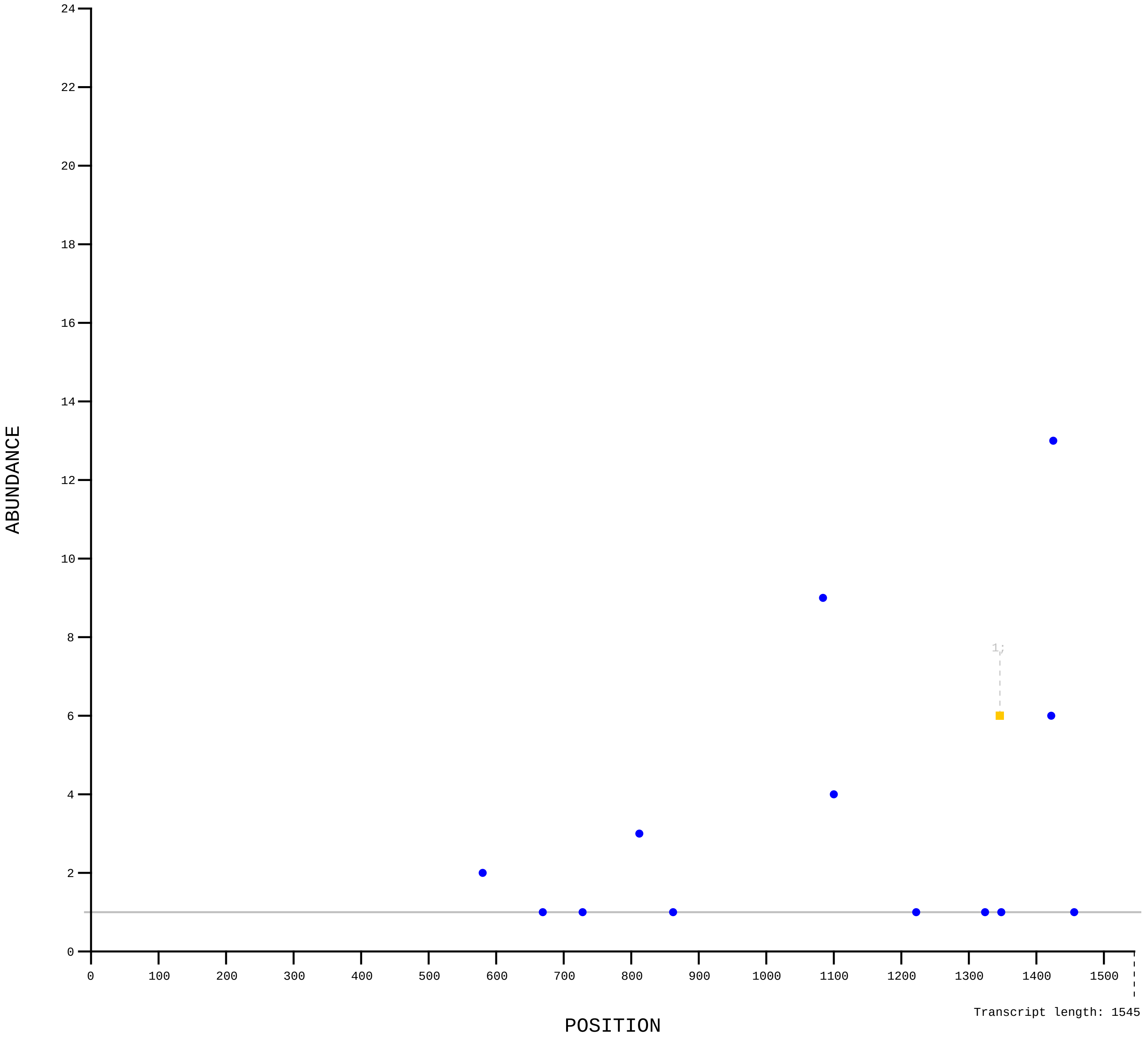

Category: 0 1 2 3 4
Degradome alignment: Median:

2 #1 Position:1346 Abundance: 6.00(deg) 7(sRNA)
5' TGTAACTTAGAGGAGACTAGG 3' ID:
||| ||| ||||o|||| ||| Score: 3.5
3' TCGAACA-TGACTCTCTTCTGCTCCTGTCACT 5' p-value: 0.0

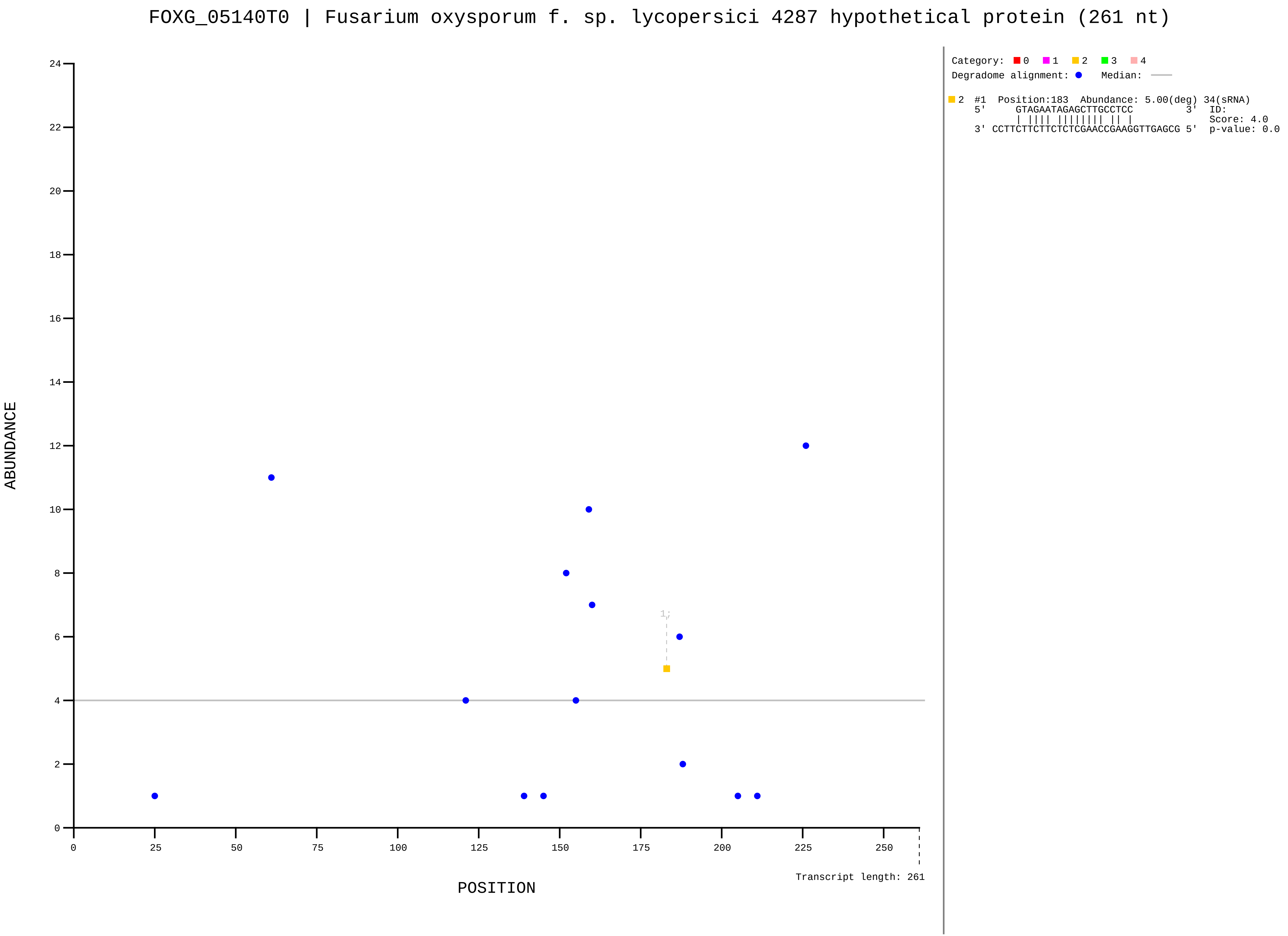

FOXG_05140T0 | Fusarium oxysporum f. sp. lycopersici 4287 hypothetical protein (261 nt)
ABUNDANCE
POSITION
Transcript length: 261
Category: 0 1 2 3 4
Degradome alignment:
Median:
2 #1 Position:183 Abundance: 5.00(deg) 34(sRNA)
5' GTAGAATAGAGCTTGCCTCC 3' ID:
Score: 4.0
3' CCTTCTTCTTCTCTCGAACCGAAGGTTGAGCG 5' p-value: 0.0

FOXG_13196T0 | Fusarium oxysporum f. sp. lycopersici 4287 hypothetical protein (939 nt)

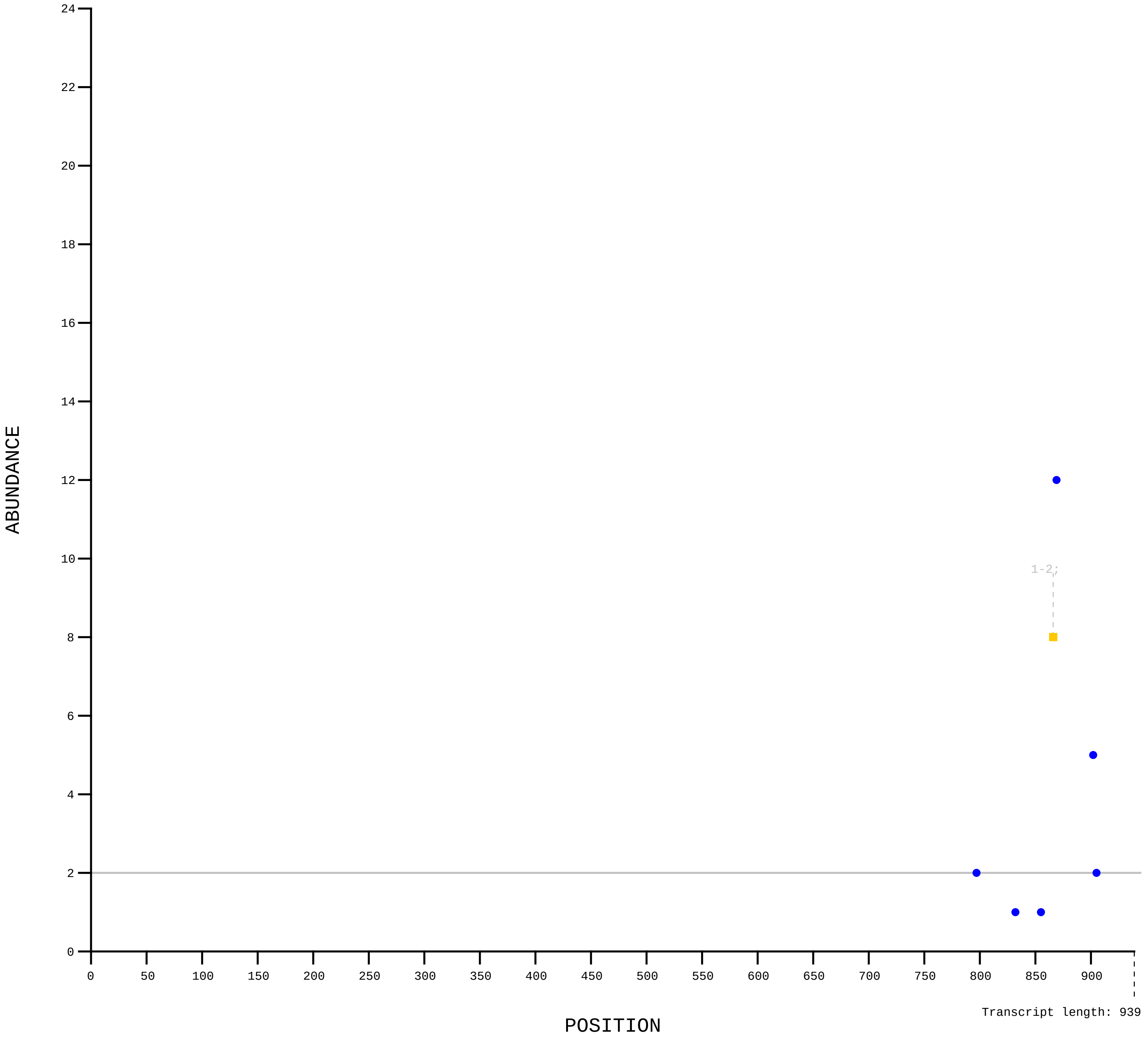

Category: 0 1 2 3 4
Degradome alignment: ● Median:

2 #1 Position:866 Abundance: 8.00(deg) 13(sRNA)

```
5'    CCGAGGTTT-TTCCCTGCCGC        3'  ID:
      |||| |o|| ||| |o|||||            Score: 4.0
3' TCTGGCTACGAATAAGTGGCGGCGGTTTAGTT 5'  p-value: 0.01
```

2 #2 Position:866 Abundance: 8.00(deg) 10(sRNA)

```
5'    CCGAGGTTT-TTCCCTGCCGCC       3'  ID:
      |||| |o|| ||| |o||||||           Score: 4.0
3' TCTGGCTACGAATAAGTGGCGGCGGTTTAGTT 5'  p-value: 0.01
```

FOXG_17426T0 | Fusarium oxysporum f. sp. lycopersici 4287 hypothetical protein (1659 nt)

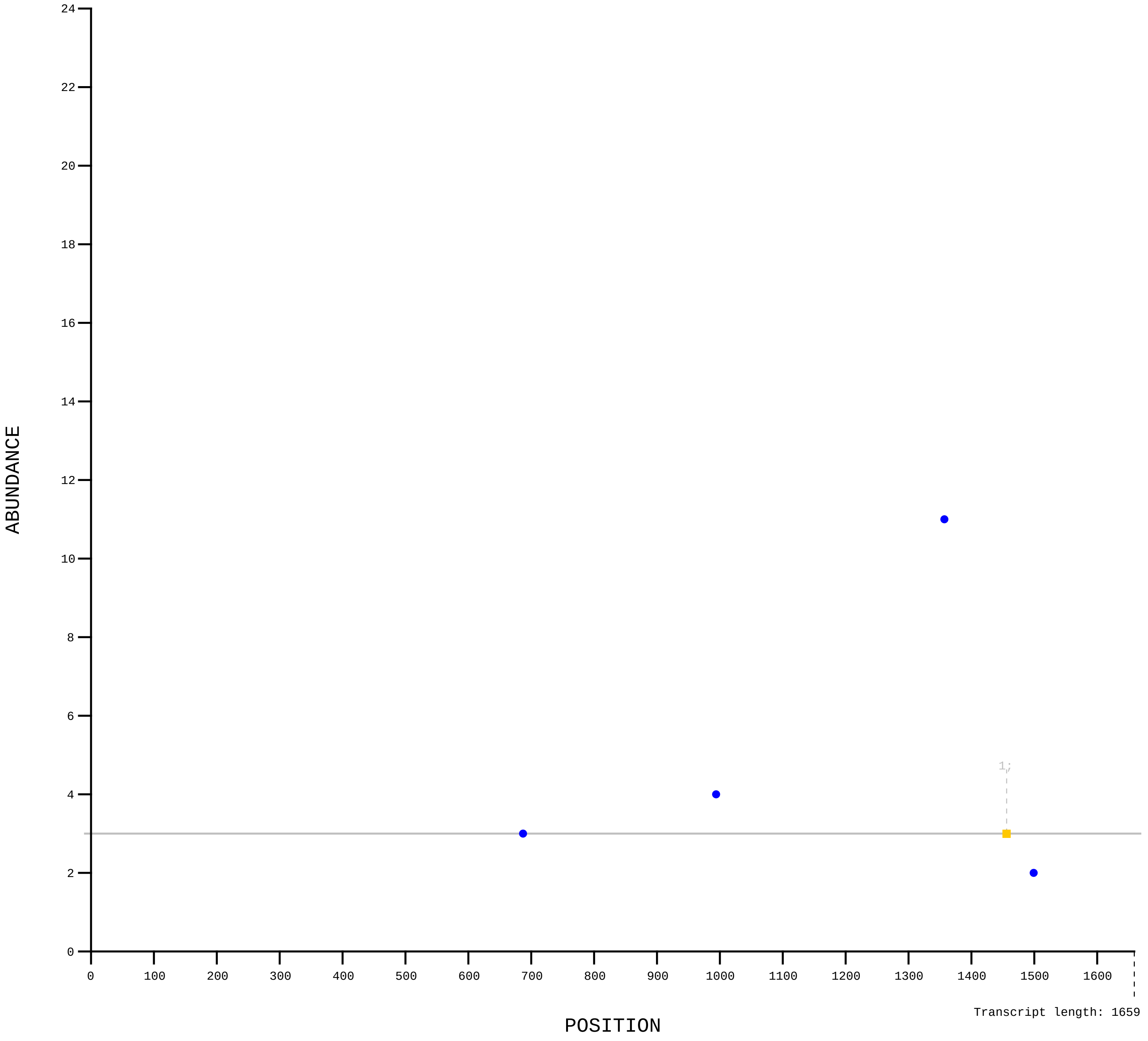

Category: 0 1 2 3 4
Degradome alignment: ● Median:

```
2  #1  Position:1456  Abundance: 3.00(deg) 5(sRNA)
   5'     TGAGACTGTAGATCCT-TCA         3'  ID:
          |||| ||||||||  | |||             Score: 4.0
   3' AAGCACTCAGACATCTACCATAGTAGTGTACG 5'  p-value: 0.02
```

FOXG_05271T0 | Fusarium oxysporum f. sp. lycopersici 4287 hypothetical protein (3051 nt)

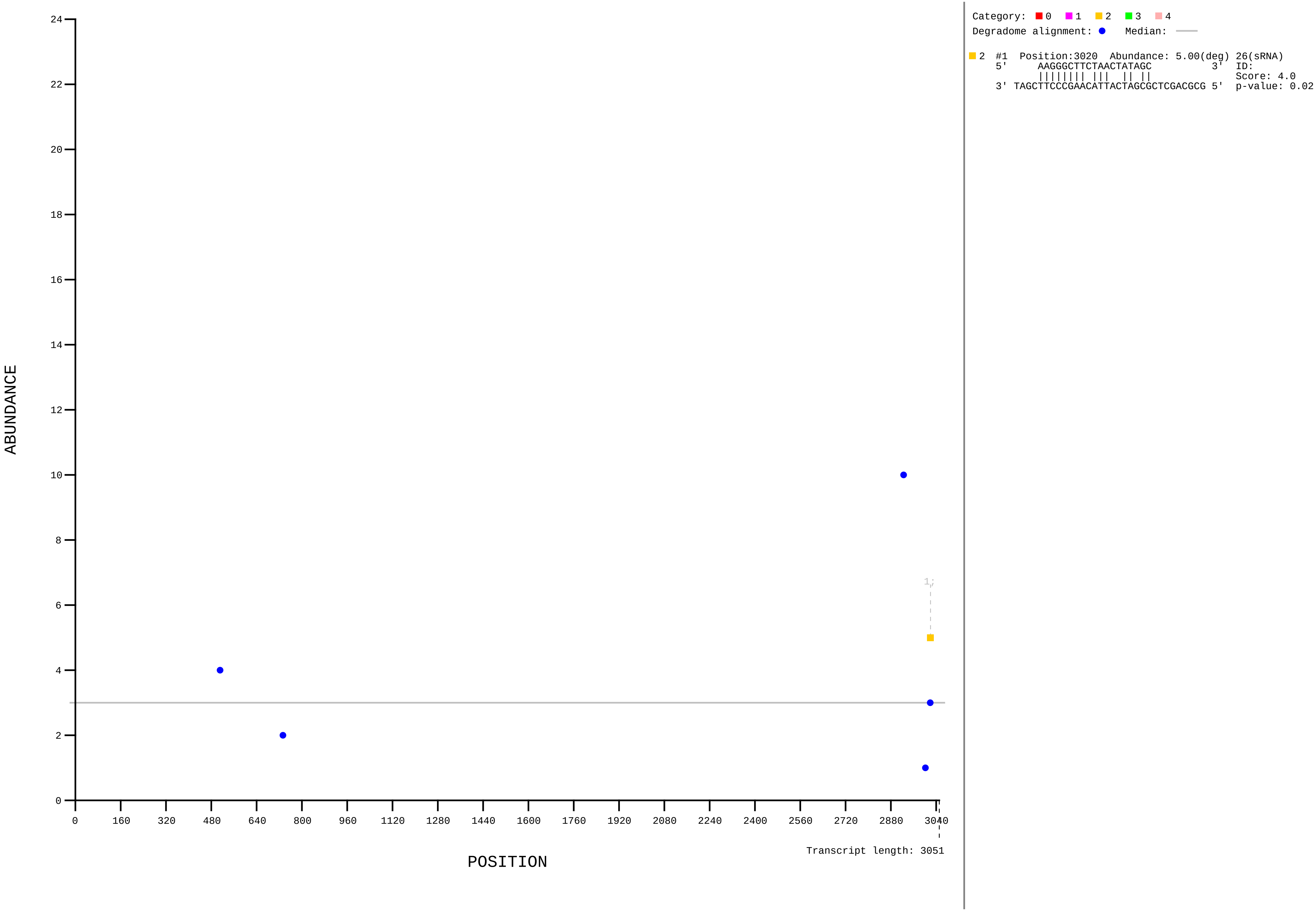

FOXG_10253T0 | Fusarium oxysporum f. sp. lycopersici 4287 hypothetical protein (1002 nt)

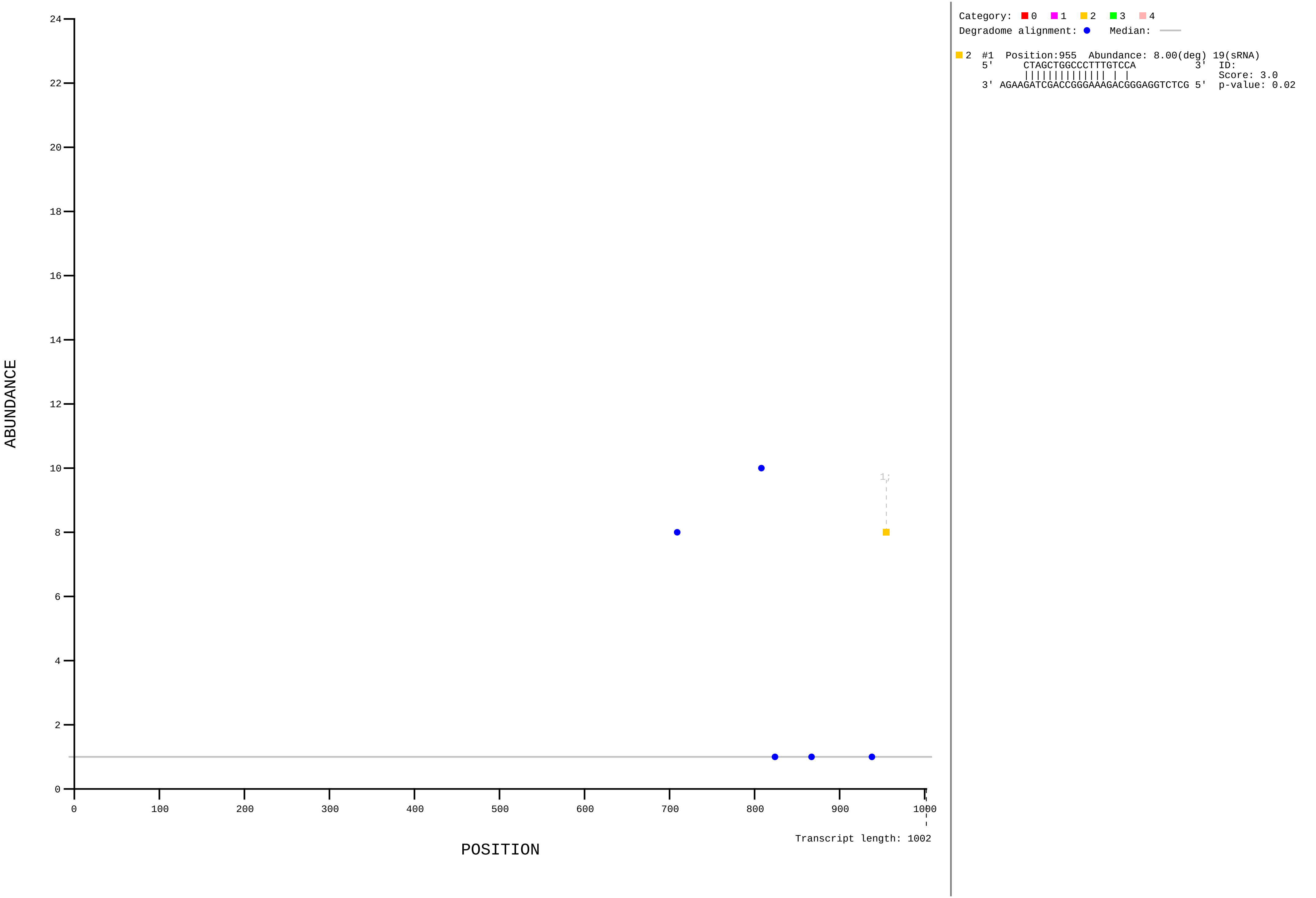

FOXG_07665T0 | Fusarium oxysporum f. sp. lycopersici 4287 cysteinyl-tRNA synthetase, mitochondri (truncated)

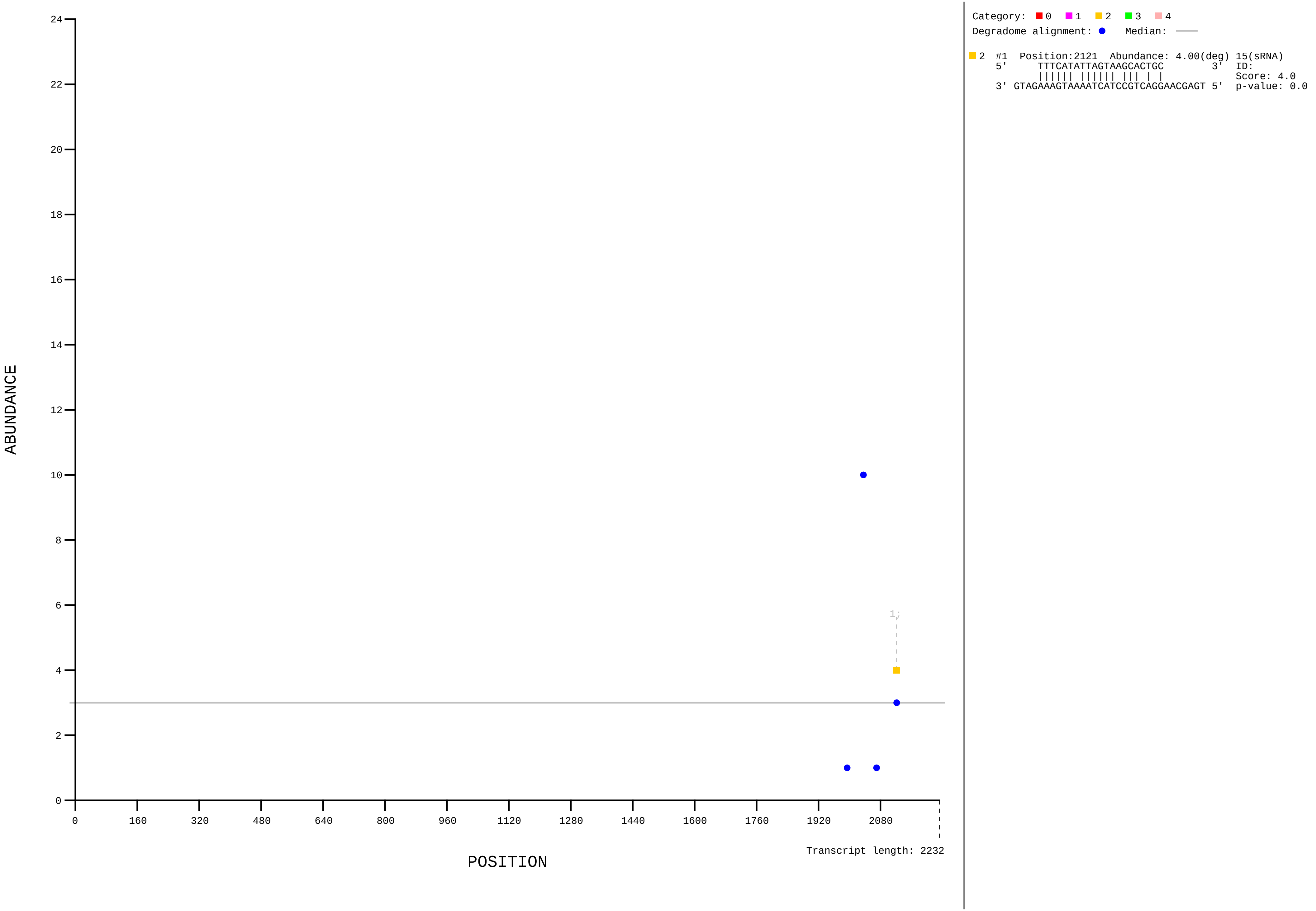

FOXG_01979T0 | Fusarium oxysporum f. sp. lycopersici 4287 hypothetical protein (210 nt)

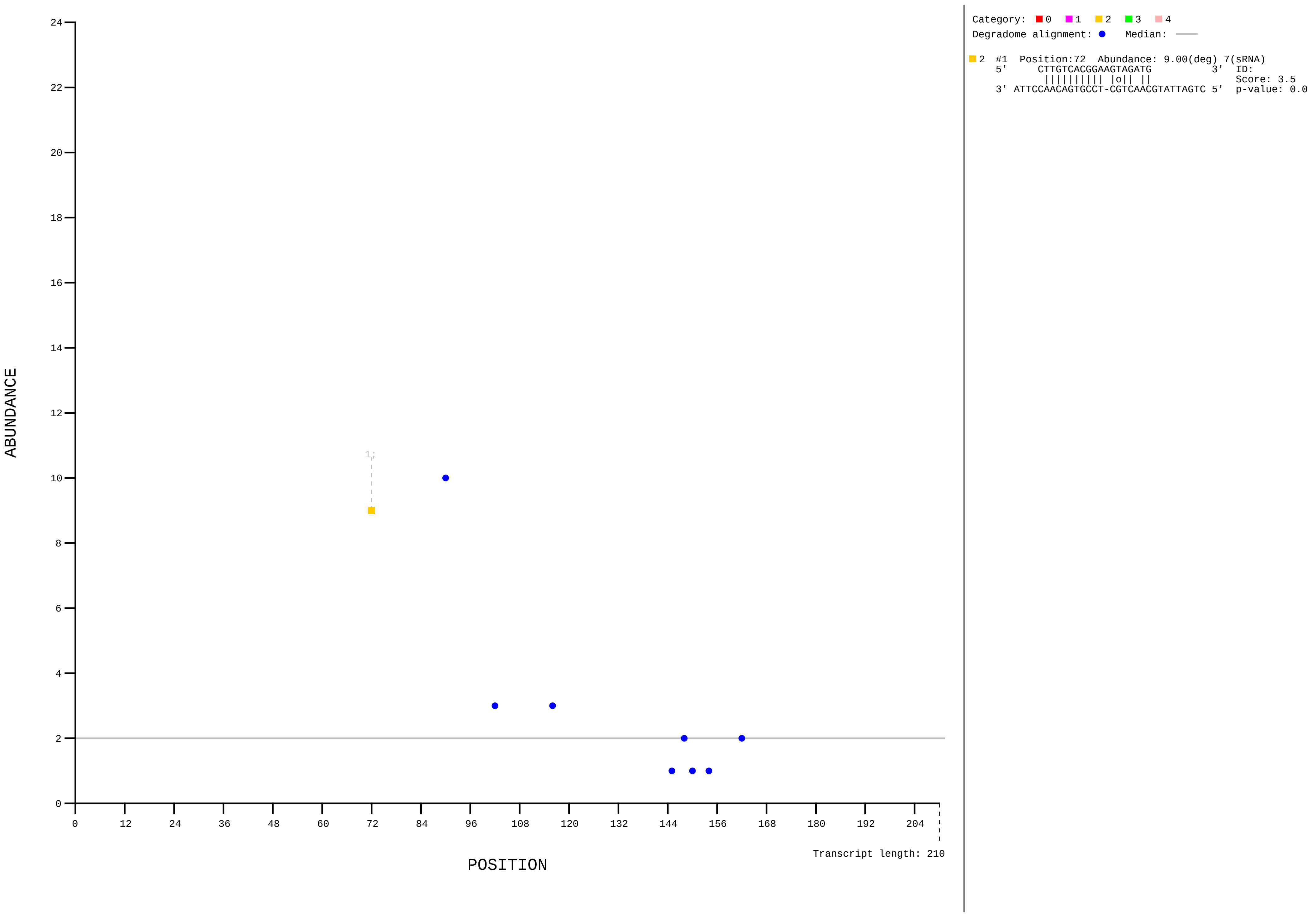

FOXG_08094T0 | Fusarium oxysporum f. sp. lycopersici 4287 succinyl-CoA:3-ketoacid-coenzyme A transferase (1560 nt)

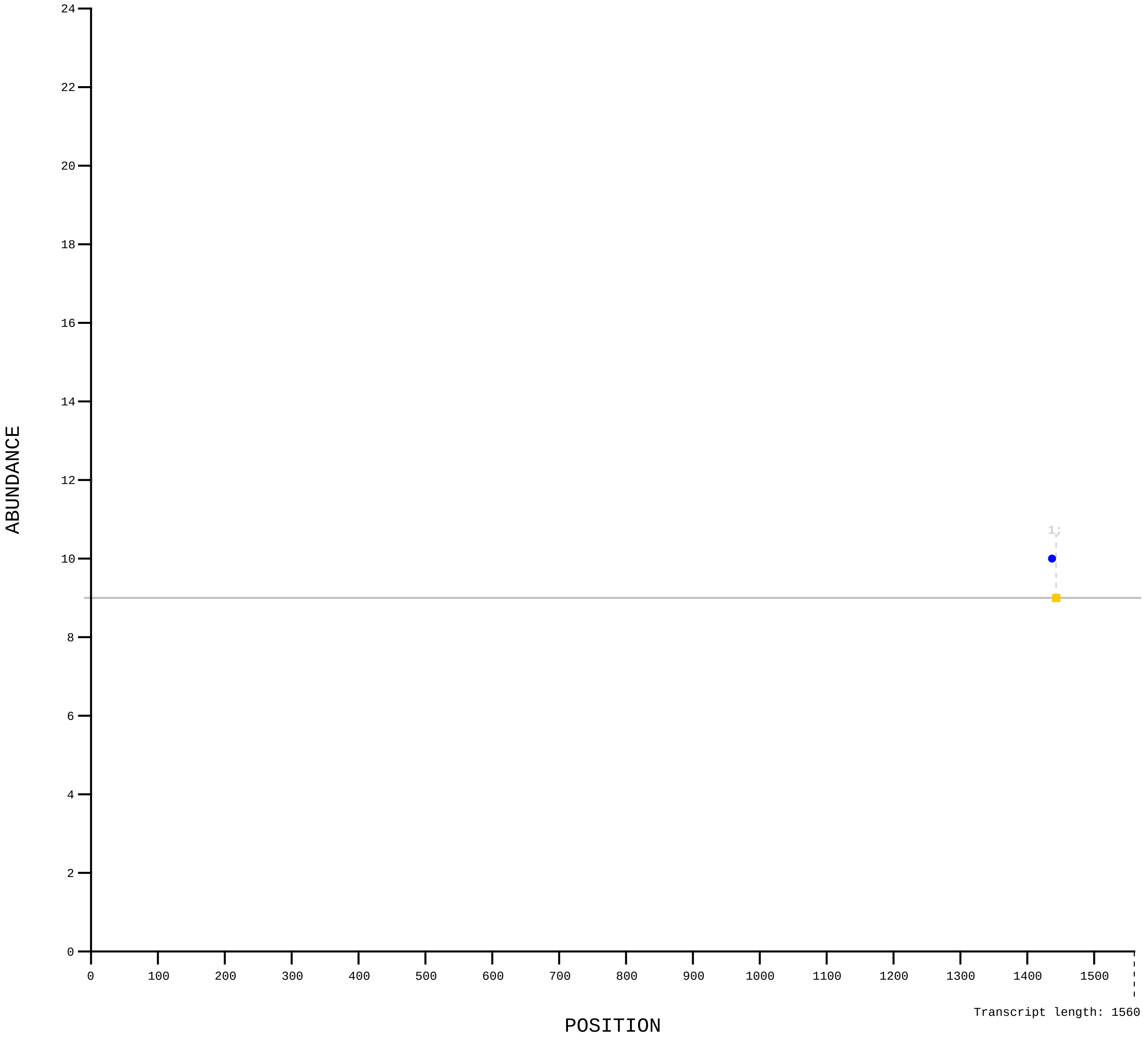

Category: 0 1 2 3 4
Degradome alignment: ● Median: —

```
2  #1  Position:1443  Abundance: 9.00(deg) 5(sRNA)
   5'     GCGACAGTCGACTACGAGC          3'  ID:
          |||| |||||||  |||o|              Score: 3.5
   3' CAACCGCT-TCAGCTGTAGCTTGTGTGGGTCG 5'  p-value: 0.01
```

FOXG_09346T0 | Fusarium oxysporum f. sp. lycopersici 4287 ornithine aminotransferase (1314 nt)

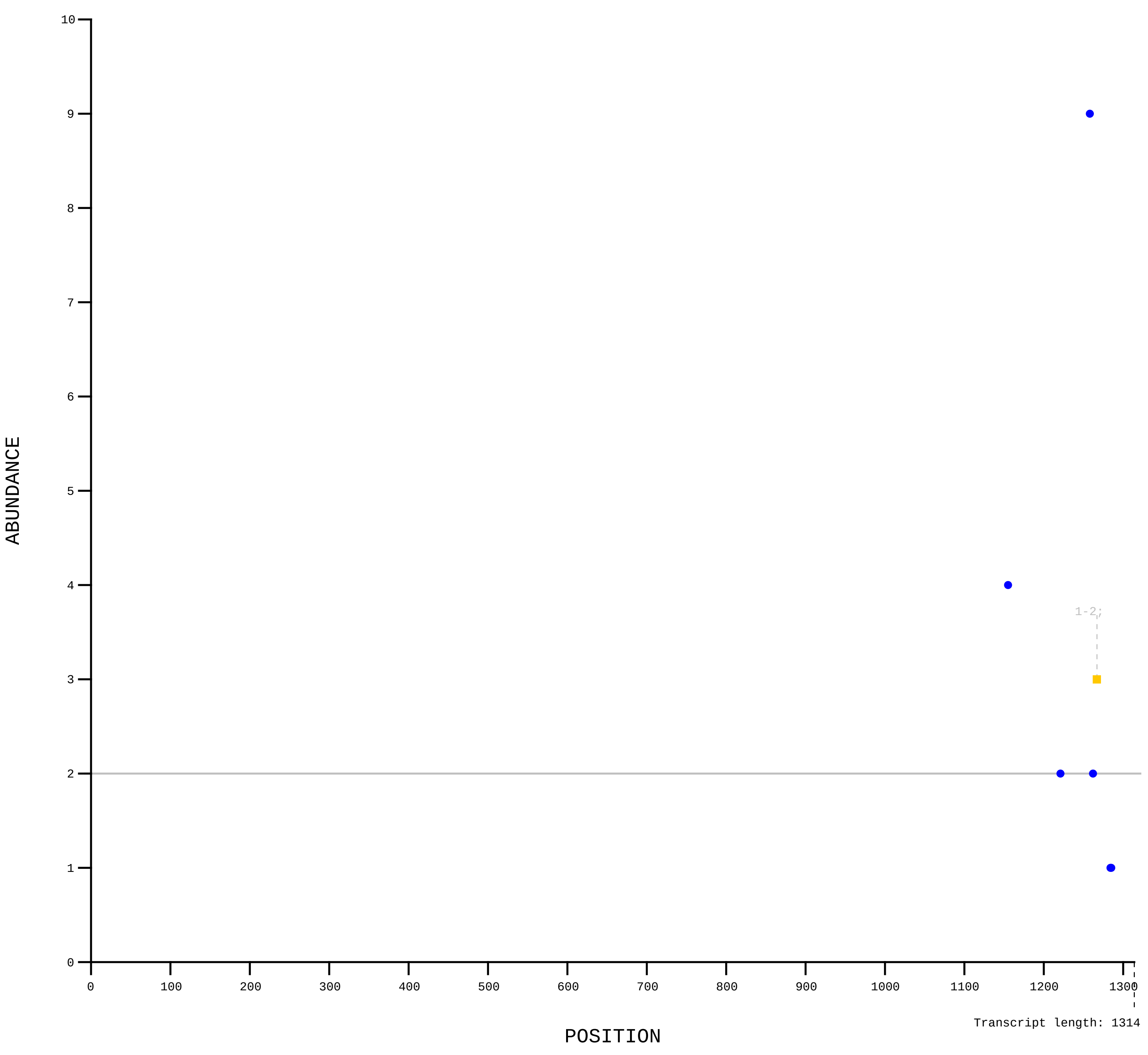

Category: 0 1 2 3 4
Degradome alignment: ● Median: —

```
2  #1  Position:1267  Abundance: 3.00(deg) 25(sRNA)
   5'     TGGTAGACTCACAAATGCAT         3'  ID:
          ||o||| |||| ||||| ||             Score: 3.5
   3' AGGAACTATCGGAGTCTTTAC-TAGAGCTCTG 5'  p-value: 0.0

2  #2  Position:1267  Abundance: 3.00(deg) 15(sRNA)
   5'     TGGTAGACTCACAAATGCA          3'  ID:
          ||o||| |||| ||||| |              Score: 3.5
   3' AGGAACTATCGGAGTCTTTAC-TAGAGCTCTG 5'  p-value: 0.01
```

FOXG_15410T0 | Fusarium oxysporum f. sp. lycopersici 4287 hypothetical protein (2949 nt)

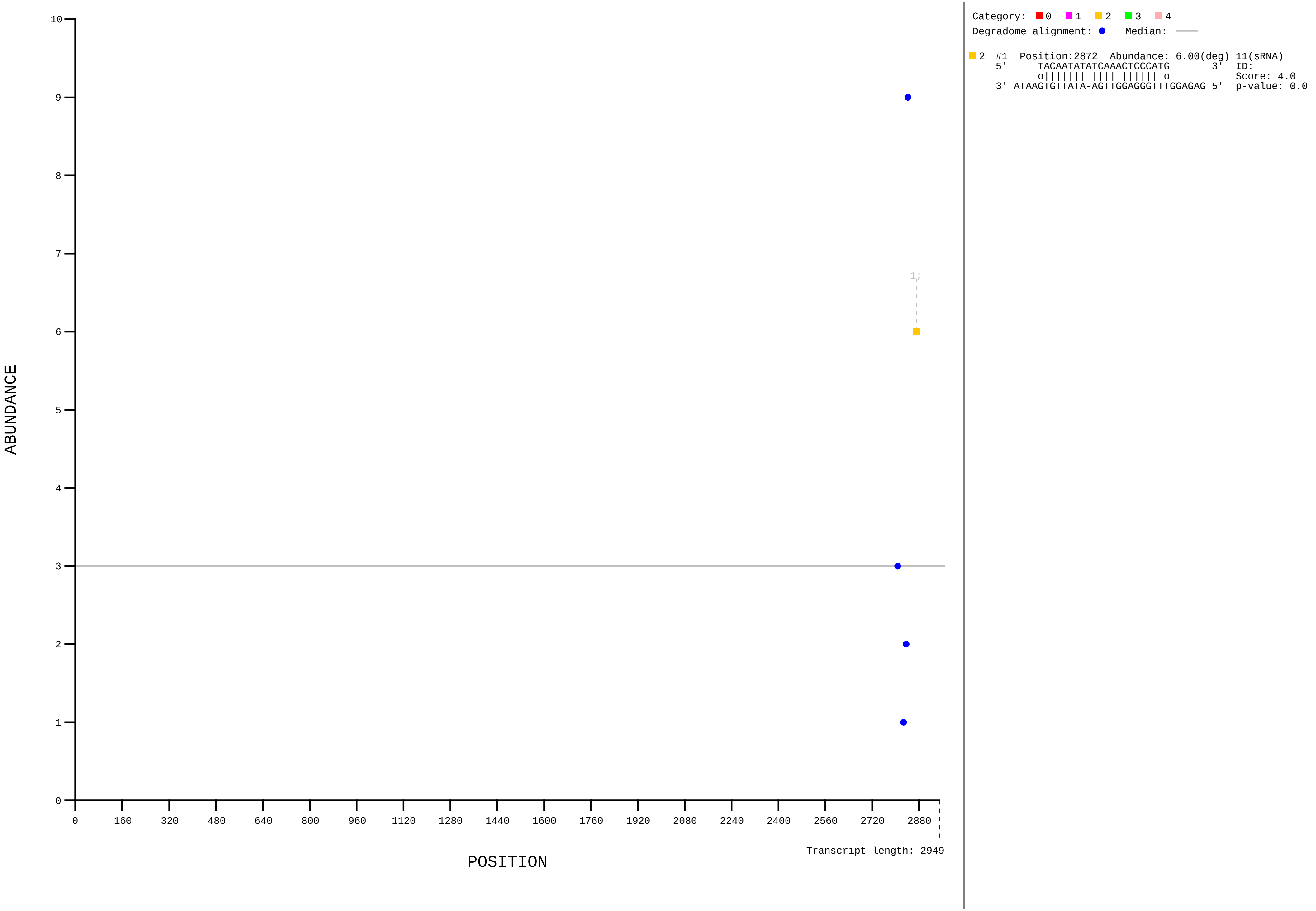

FOXG_08379T0 | Fusarium oxysporum f. sp. lycopersici 4287 FK506-binding protein 1 (447 nt)

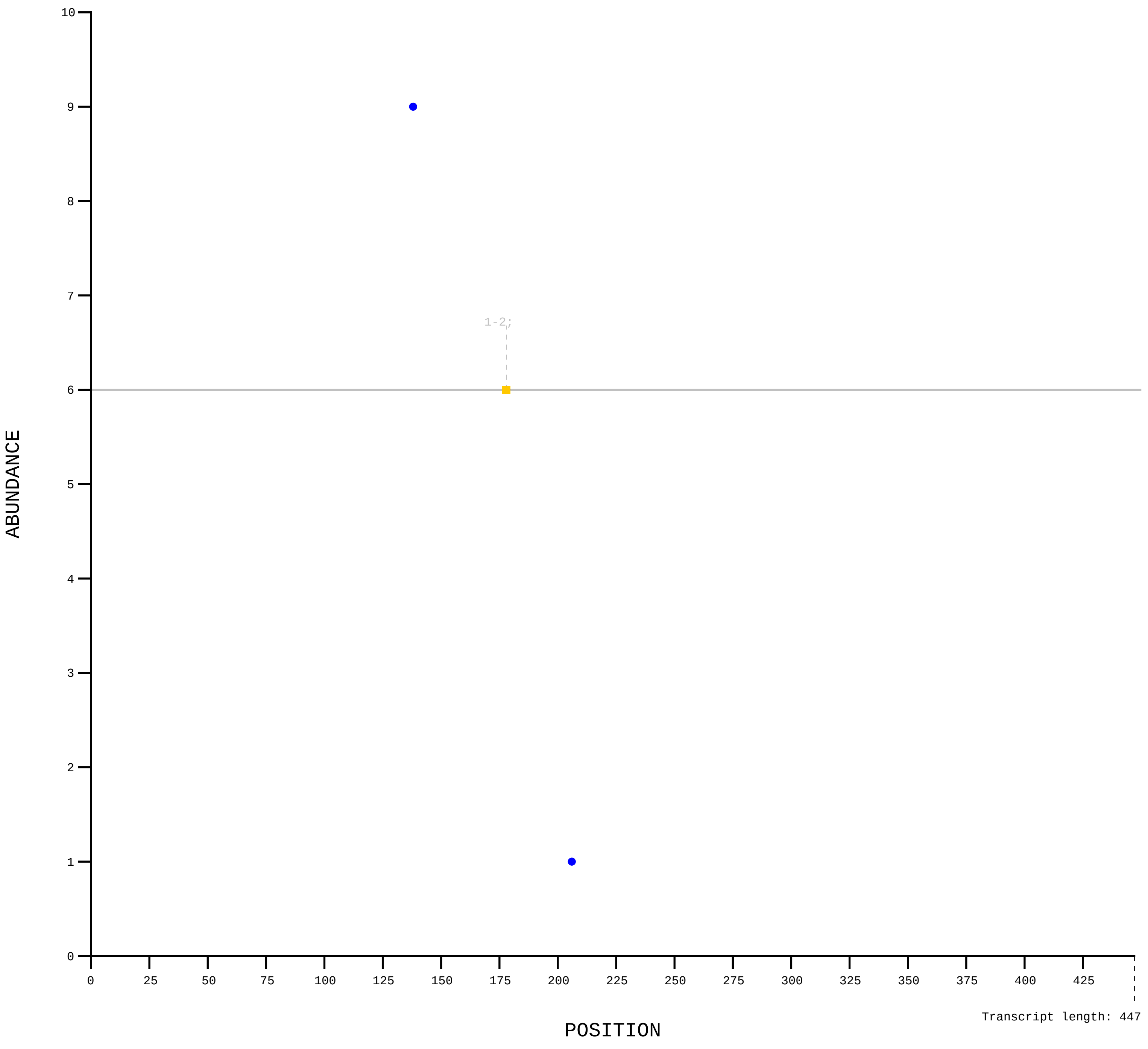


```
Category: 0 1 2 3 4
Degradome alignment:    Median:

2  #1  Position:178  Abundance: 6.00(deg) 9(sRNA)
   5'     TGGTATGTTGGATGGCGGCC         3'  ID:
          o||| |o|||||||| |o||             Score: 3.5
   3' GTCGGCCACATAACCTACCACTGGAAAACCGG 5'  p-value: 0.0

2  #2  Position:178  Abundance: 6.00(deg) 8(sRNA)
   5'     TGGTATGTTGGATGGCGGC          3'  ID:
          o||| |o|||||||| |o|              Score: 3.5
   3' GTCGGCCACATAACCTACCACTGGAAAACCGG 5'  p-value: 0.0
```

FOXG_03438T0 | Fusarium oxysporum f. sp. lycopersici 4287 hypothetical protein (1845 nt)

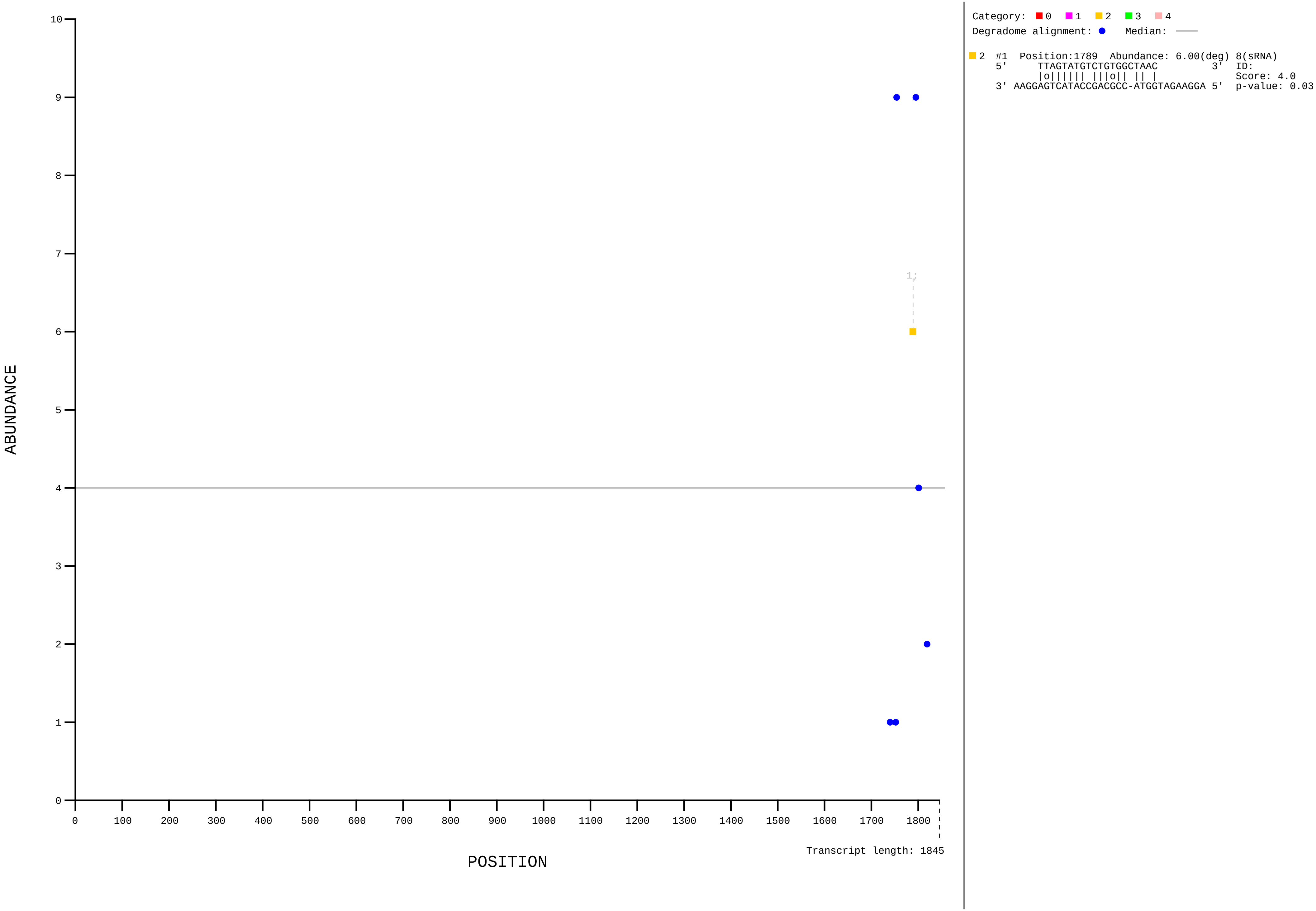

FOXG_03462T0 | Fusarium oxysporum f. sp. lycopersici 4287 hypothetical protein (534 nt)

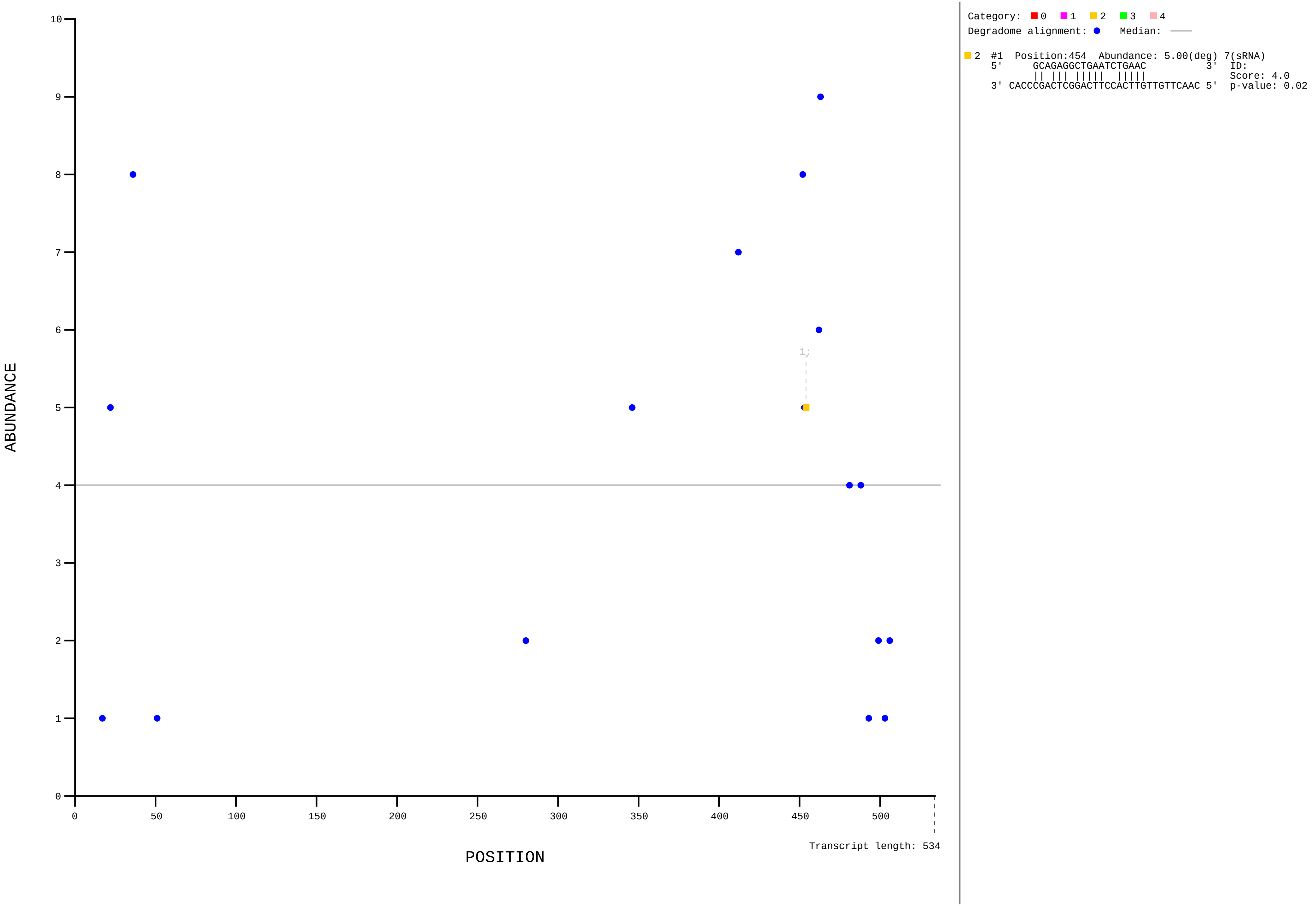

FOXG_06305T0 | Fusarium oxysporum f. sp. lycopersici 4287 vacuolar protein sorting-associated p (truncated)

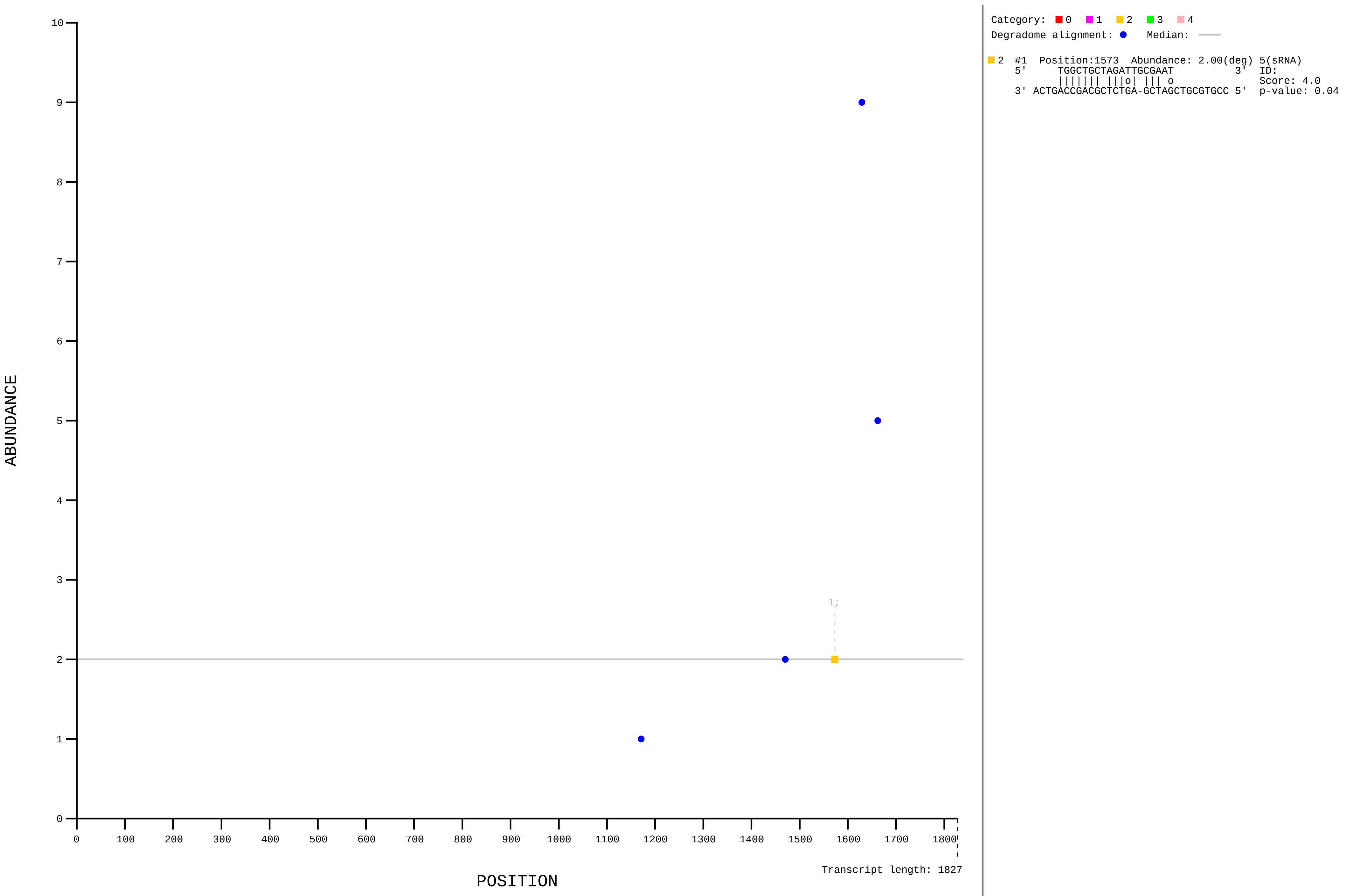

FOXG_15398T0 | Fusarium oxysporum f. sp. lycopersici 4287 multidrug resistant protein (1827 nt)

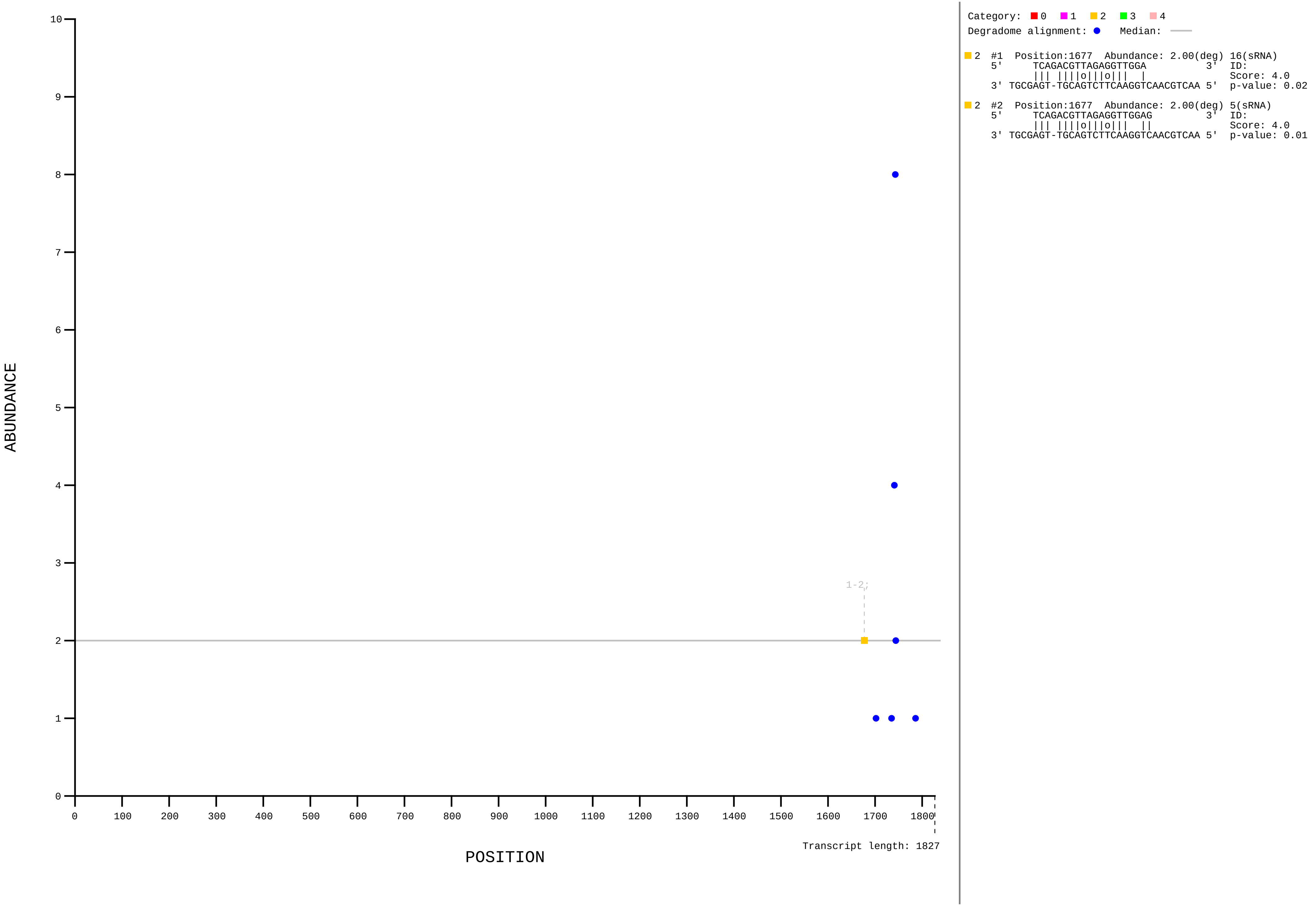

FOXG_04362T0 | Fusarium oxysporum f. sp. lycopersici 4287 hypothetical protein (990 nt)

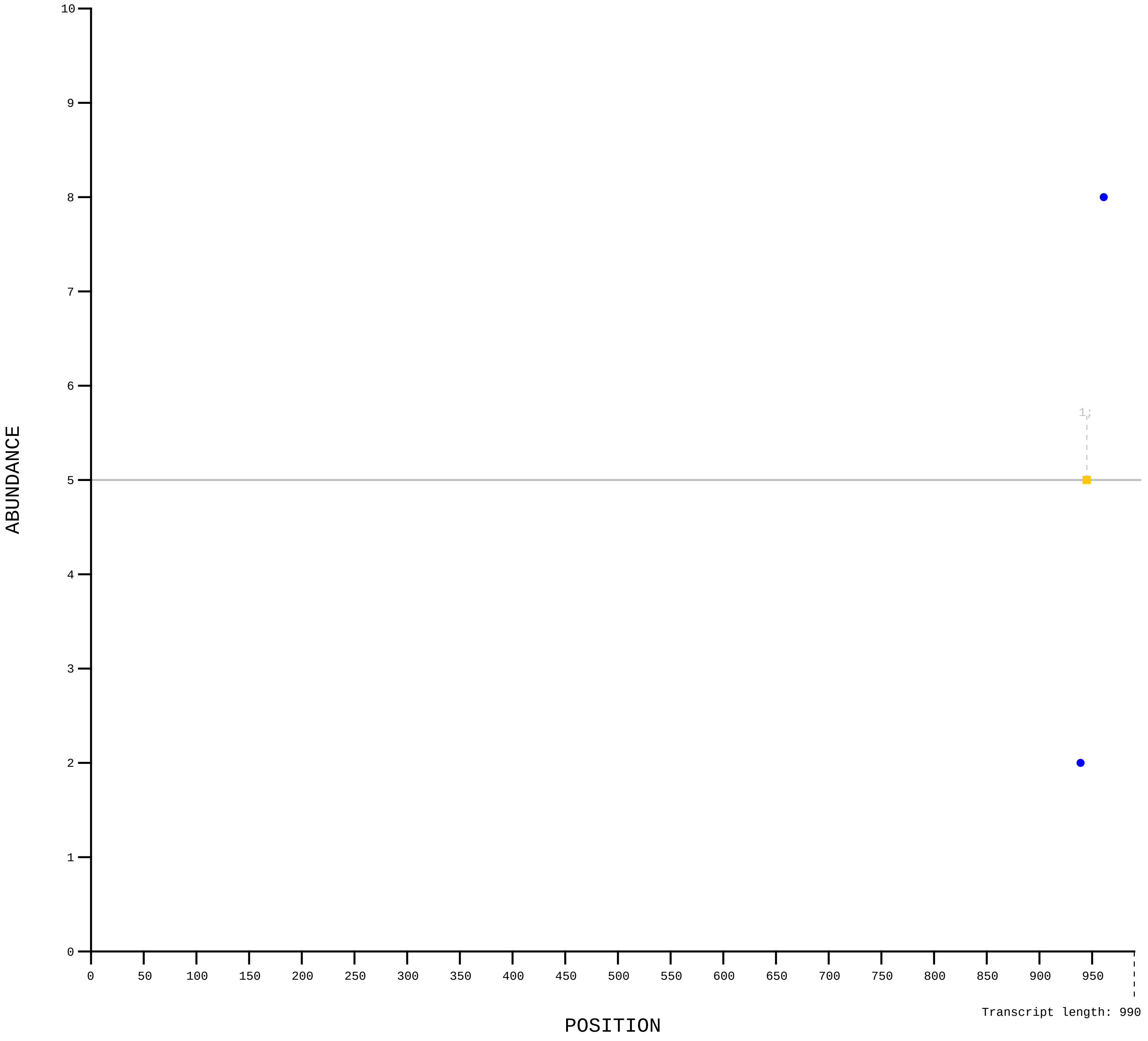

Category: 0 1 2 3 4
Degradome alignment: ● Median:

```
2  #1  Position:945  Abundance: 5.00(deg) 6(sRNA)
   5'     AGCTTTGTCCAGAACTCGCGG         3'  ID:
          |||o|||o||||| |o ||||             Score: 3.5
   3' TCTCTCGGAACGGGTCT-GGTCGCCCTCTCGC 5'  p-value: 0.0
```

FOXG_00706T0 | Fusarium oxysporum f. sp. lycopersici 4287 AhpC/TSA family protein (555 nt)

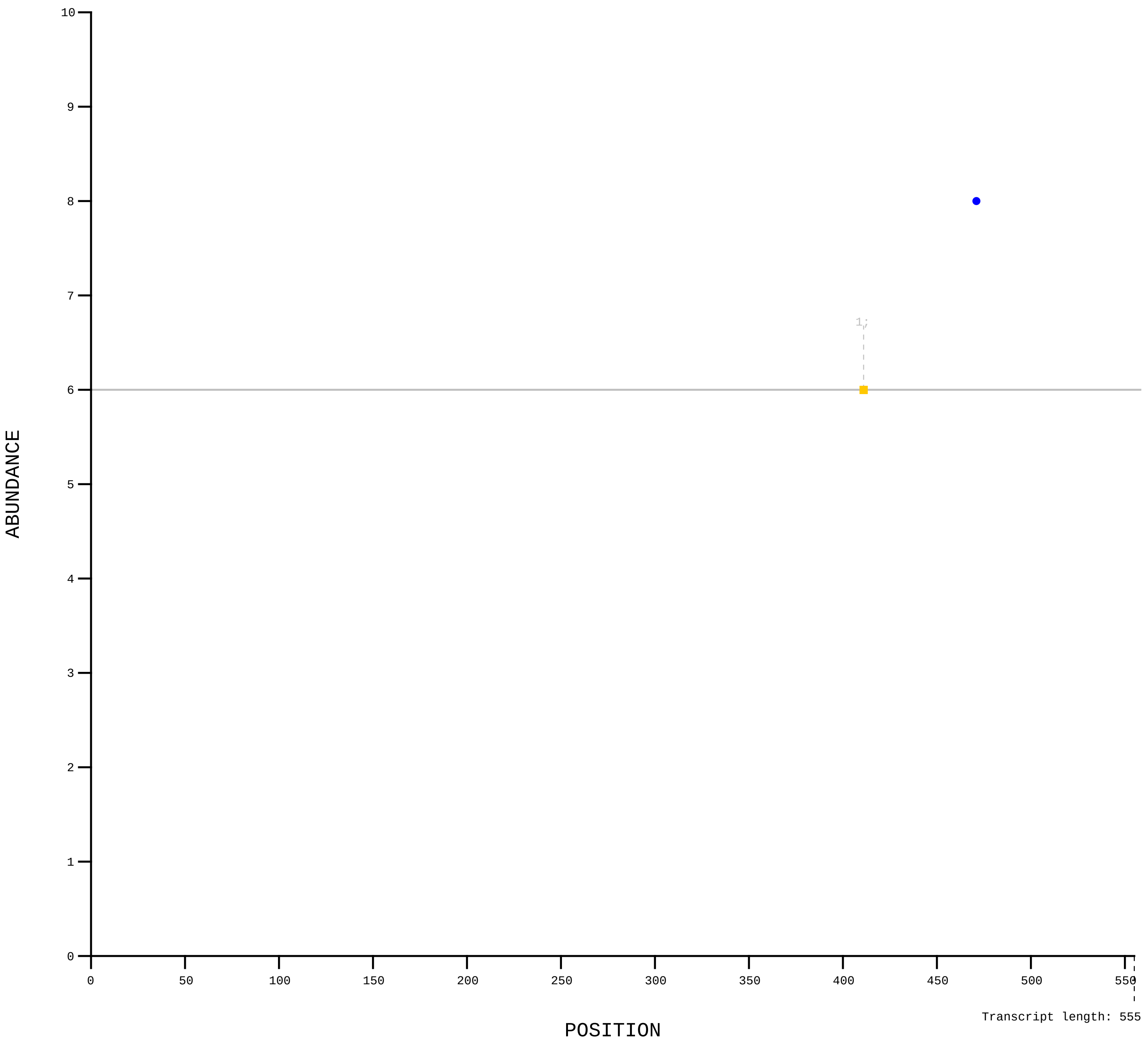

Category: 0 1 2 3 4
Degradome alignment: ● Median:

```
2  #1  Position:411  Abundance: 6.00(deg) 5(sRNA)
   5'     TCGAAGACGAAGGACCACGGC        3'  ID:
          |||||| | |||| ||| |||            Score: 4.0
   3' GCGGAGCTTC-GGTTCCAGGTCCCGGAATCAC 5'  p-value: 0.0
```

FOXG_10533T0 | Fusarium oxysporum f. sp. lycopersici 4287 malate dehydrogenase (1011 nt)

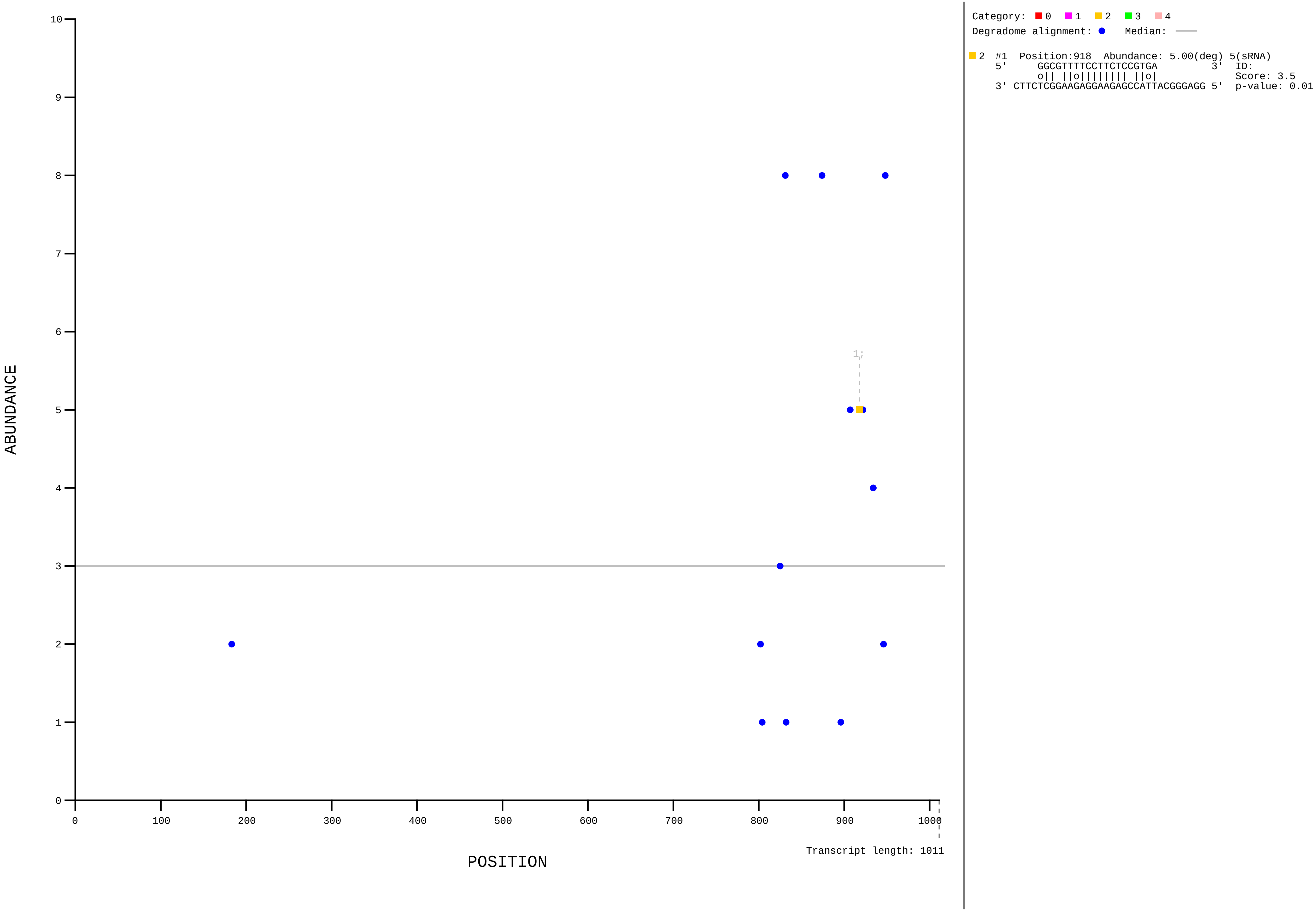

FOXG_05109T0 | Fusarium oxysporum f. sp. lycopersici 4287 NADH-ubiquinone oxidoreductase 21.3 kDa subunit (573 nt)

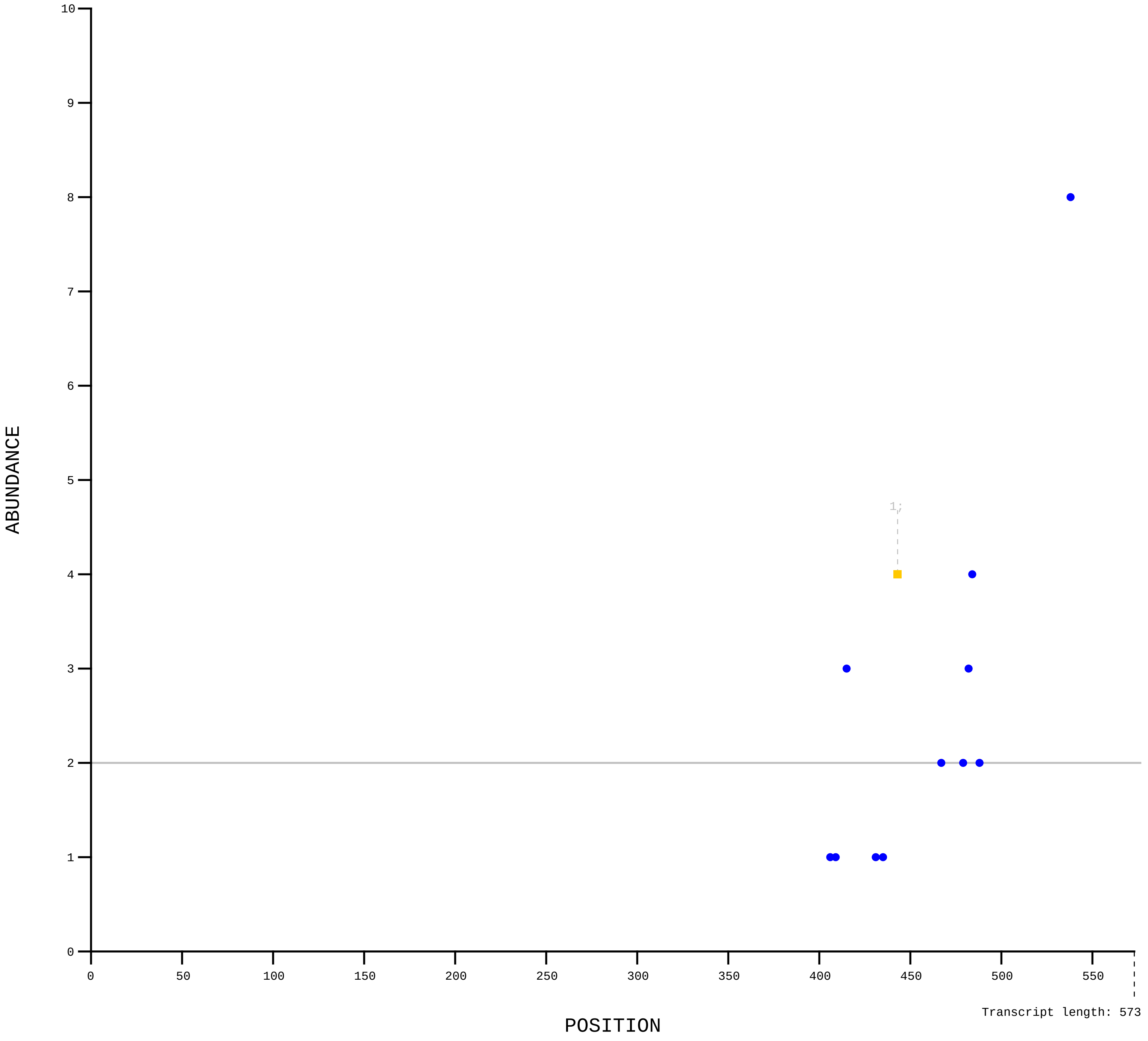

Category: 0 1 2 3 4
Degradome alignment: ● Median: —

```
2  #1  Position:443  Abundance: 4.00(deg) 5(sRNA)
   5'     ACGGACACATCTTT-GTAGT         3'  ID:
          |||o|||||||o|| || |o             Score: 3.5
   3' TAATTGCTTGTGTAGGAATCAGCGTAGCCAGC 5'  p-value: 0.0
```

FOXG_14085T0 | Fusarium oxysporum f. sp. lycopersici 4287 hypothetical protein (1317 nt)

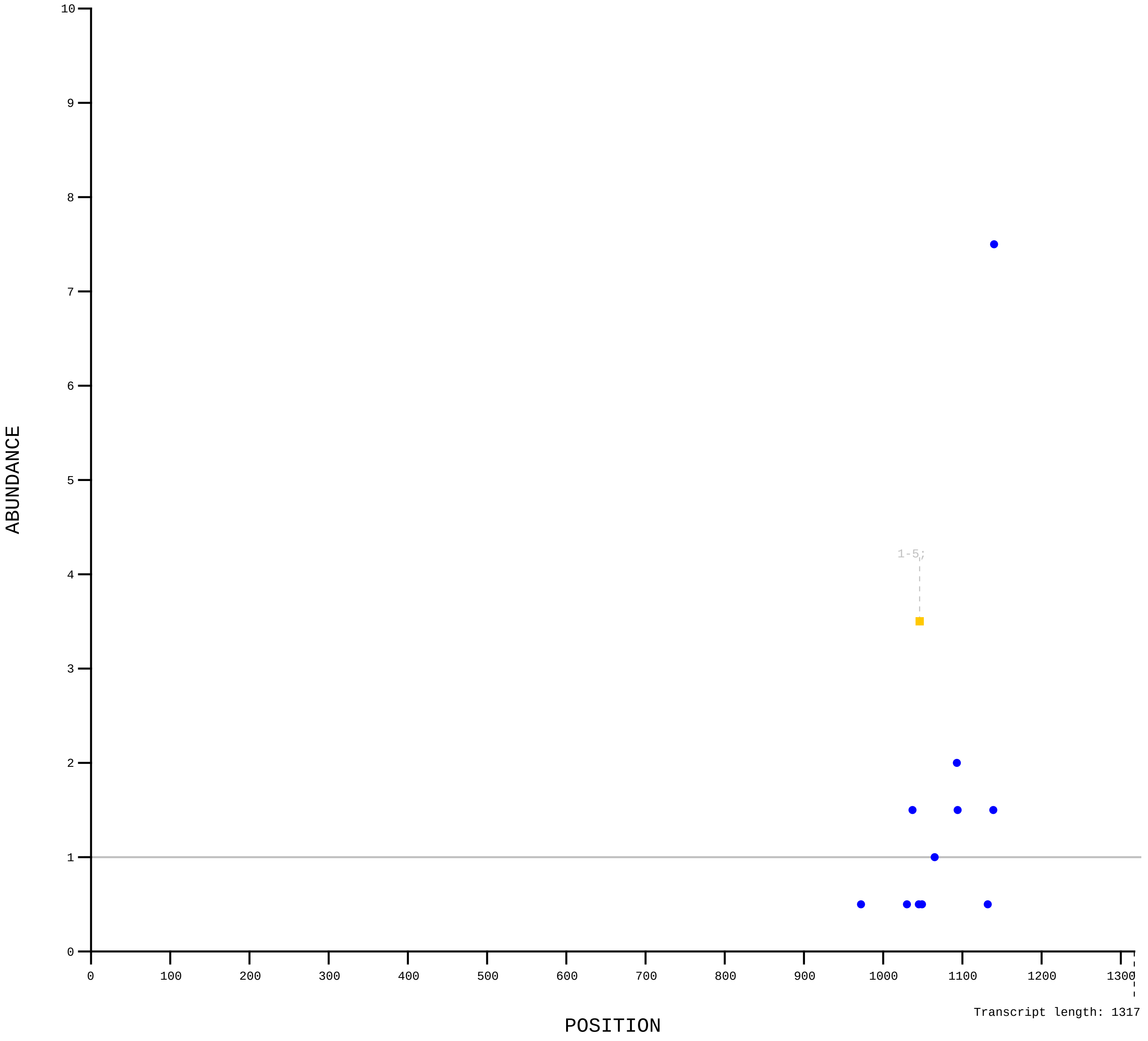


```
Category: ■0 ■1 ■2 ■3 ■4
Degradome alignment: ●    Median: ——

■2  #1  Position:1046  Abundance: 3.50(deg) 102(sRNA)
    5'     TGCACATGATTCCGCCCACA         3'  ID:
           ||||||||||| ||||| ||             Score: 2.0
    3' GGCCACGTGTACTAATGCGGGCGTGAAGCCGC 5'  p-value: 0.0

■2  #2  Position:1046  Abundance: 3.50(deg) 21(sRNA)
    5'     TGCACATGATTCTGCCCACA         3'  ID:
           ||||||||||| o|||| ||             Score: 2.5
    3' GGCCACGTGTACTAATGCGGGCGTGAAGCCGC 5'  p-value: 0.0

■2  #3  Position:1046  Abundance: 3.50(deg) 14(sRNA)
    5'     TGCACATGATTCCGCCCAC          3'  ID:
           ||||||||||| ||||| |              Score: 2.0
    3' GGCCACGTGTACTAATGCGGGCGTGAAGCCGC 5'  p-value: 0.0

■2  #4  Position:1046  Abundance: 3.50(deg) 7(sRNA)
    5'     TGCACATGATTCCGCCCACAGTG      3'  ID:
           ||||||||||| ||||| || |           Score: 4.0
    3' GGCCACGTGTACTAATGCGGGCGTGAAGCCGC 5'  p-value: 0.01

■2  #5  Position:1046  Abundance: 3.50(deg) 5(sRNA)
    5'     TGCACATGATTCCGCCCACAG        3'  ID:
           ||||||||||| ||||| ||             Score: 3.0
    3' GGCCACGTGTACTAATGCGGGCGTGAAGCCGC 5'  p-value: 0.0
```

FOXG_12452T0 | Fusarium oxysporum f. sp. lycopersici 4287 hypothetical protein (1284 nt)

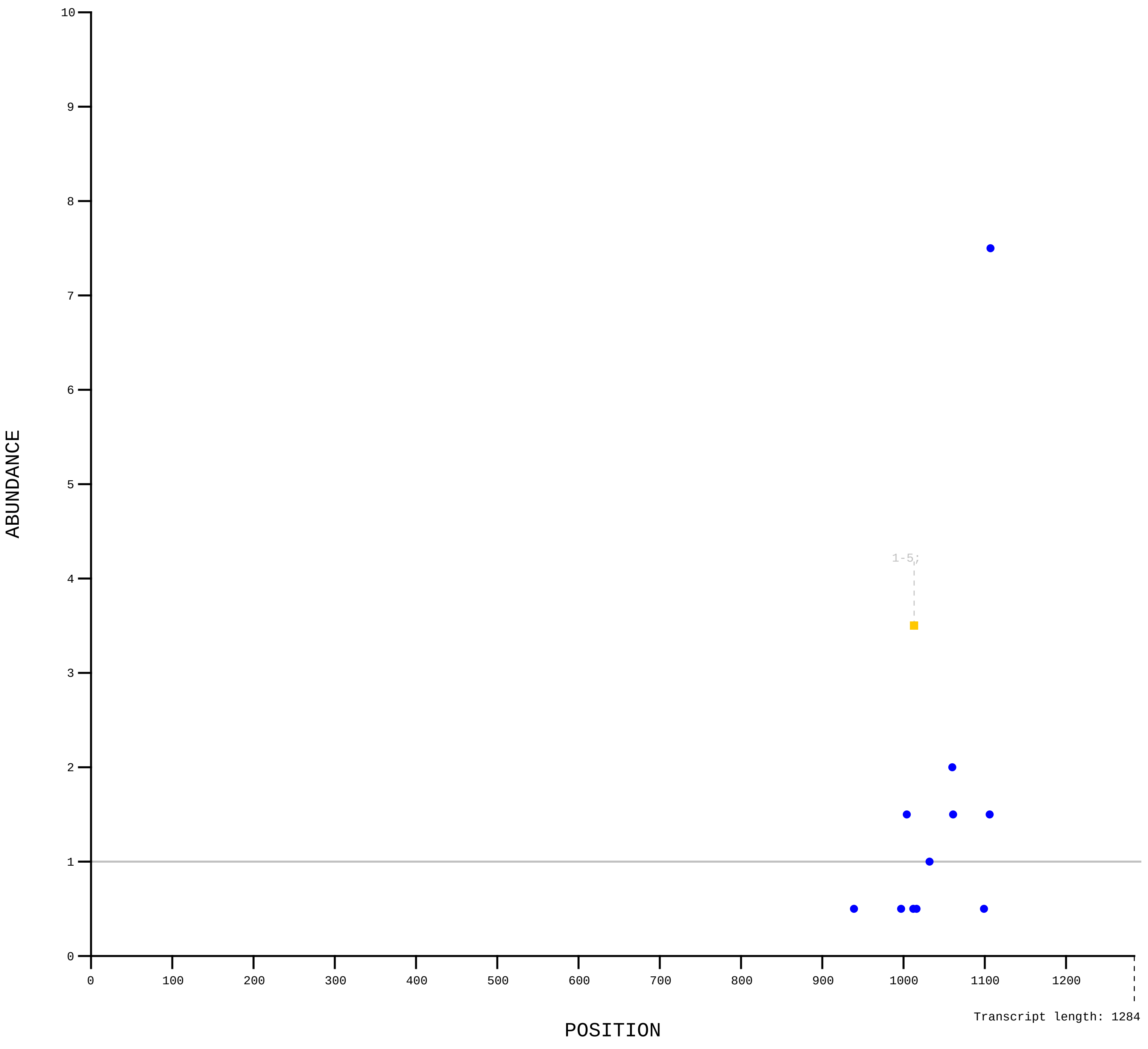


```
Category: ■0 ■1 ■2 ■3 ■4
Degradome alignment: ●    Median: ——

■2  #1  Position:1013  Abundance: 3.50(deg) 102(sRNA)
    5'     TGCACATGATTCCGCCCACA         3'  ID:
           ||||||||||| ||||| ||             Score: 2.0
    3' GGCCACGTGTACTAATGCGGGCGTGAAGCCGC 5'  p-value: 0.0

■2  #2  Position:1013  Abundance: 3.50(deg) 21(sRNA)
    5'     TGCACATGATTCTGCCCACA         3'  ID:
           ||||||||||| o|||| ||             Score: 2.5
    3' GGCCACGTGTACTAATGCGGGCGTGAAGCCGC 5'  p-value: 0.0

■2  #3  Position:1013  Abundance: 3.50(deg) 14(sRNA)
    5'     TGCACATGATTCCGCCCAC          3'  ID:
           ||||||||||| ||||| |              Score: 2.0
    3' GGCCACGTGTACTAATGCGGGCGTGAAGCCGC 5'  p-value: 0.0

■2  #4  Position:1013  Abundance: 3.50(deg) 7(sRNA)
    5'     TGCACATGATTCCGCCCACAGTG      3'  ID:
           ||||||||||| ||||| || |           Score: 4.0
    3' GGCCACGTGTACTAATGCGGGCGTGAAGCCGC 5'  p-value: 0.0

■2  #5  Position:1013  Abundance: 3.50(deg) 5(sRNA)
    5'     TGCACATGATTCCGCCCACAG        3'  ID:
           ||||||||||| ||||| ||             Score: 3.0
    3' GGCCACGTGTACTAATGCGGGCGTGAAGCCGC 5'  p-value: 0.0
```

FOXG_09465T0 | Fusarium oxysporum f. sp. lycopersici 4287 40S ribosomal protein S3 (783 nt)

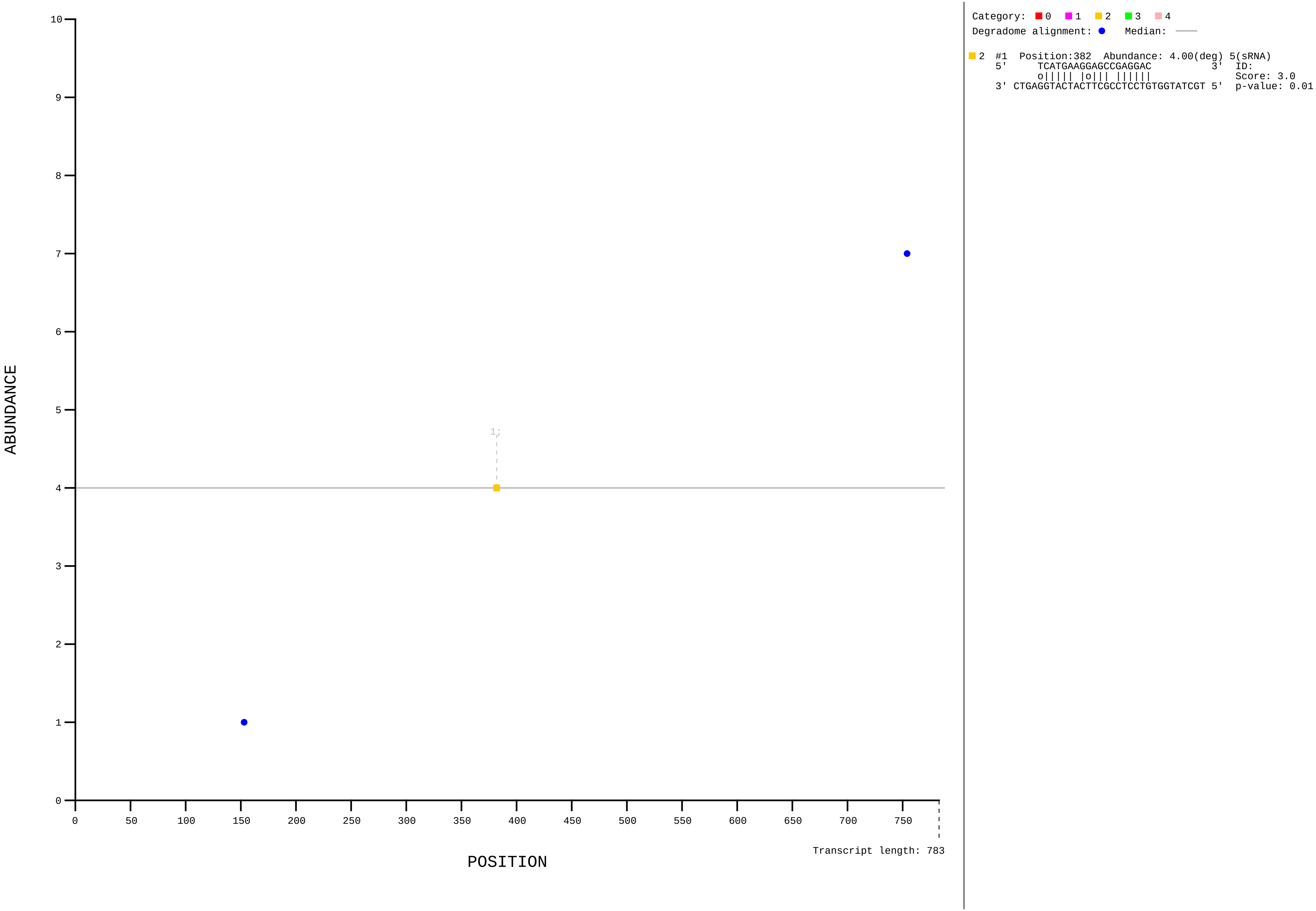

FOXG_10287T0 | Fusarium oxysporum f. sp. lycopersici 4287 damage response protein 1 (516 nt)

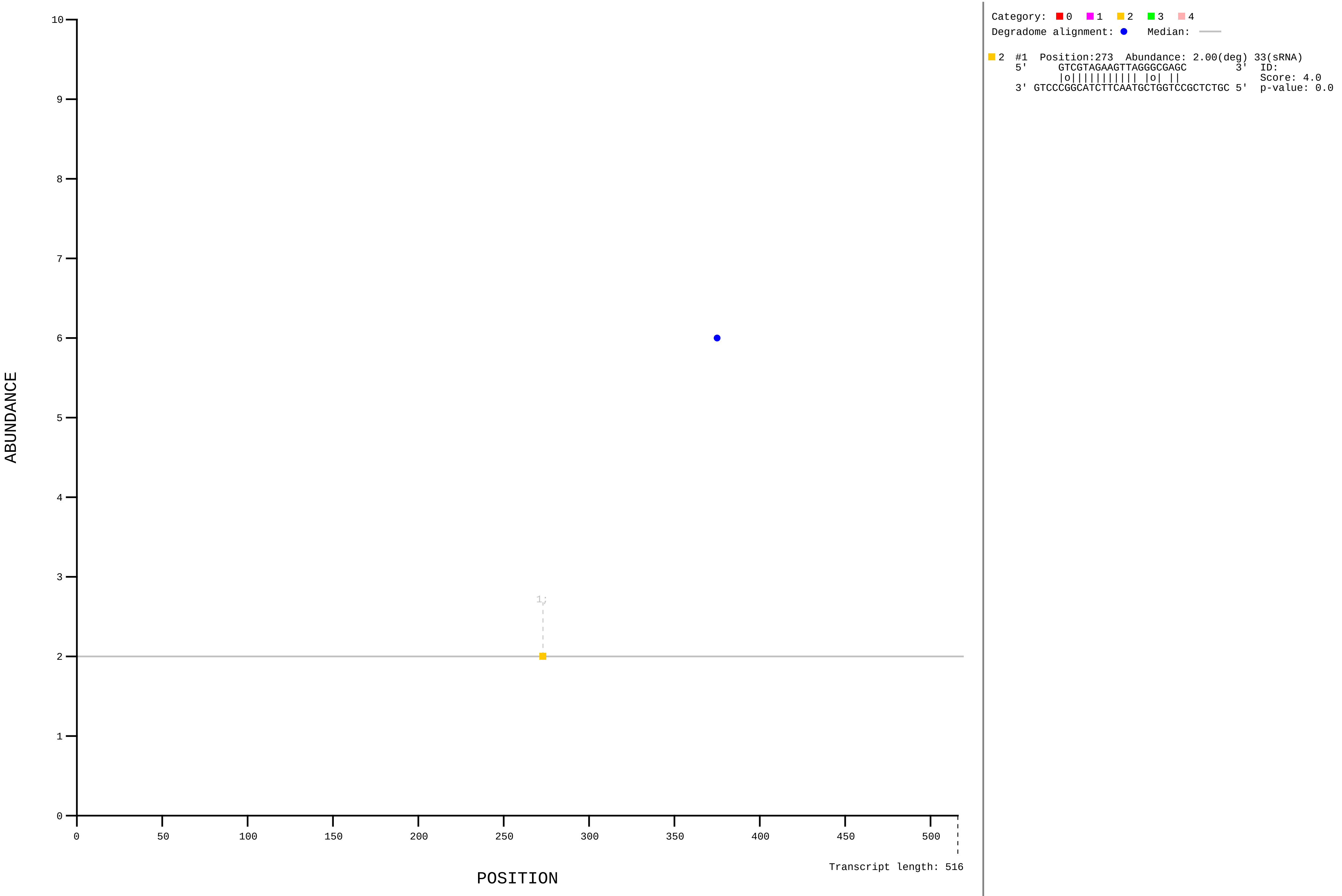

FOXG_00269T0 | Fusarium oxysporum f. sp. lycopersici 4287 verprolin (2994 nt)

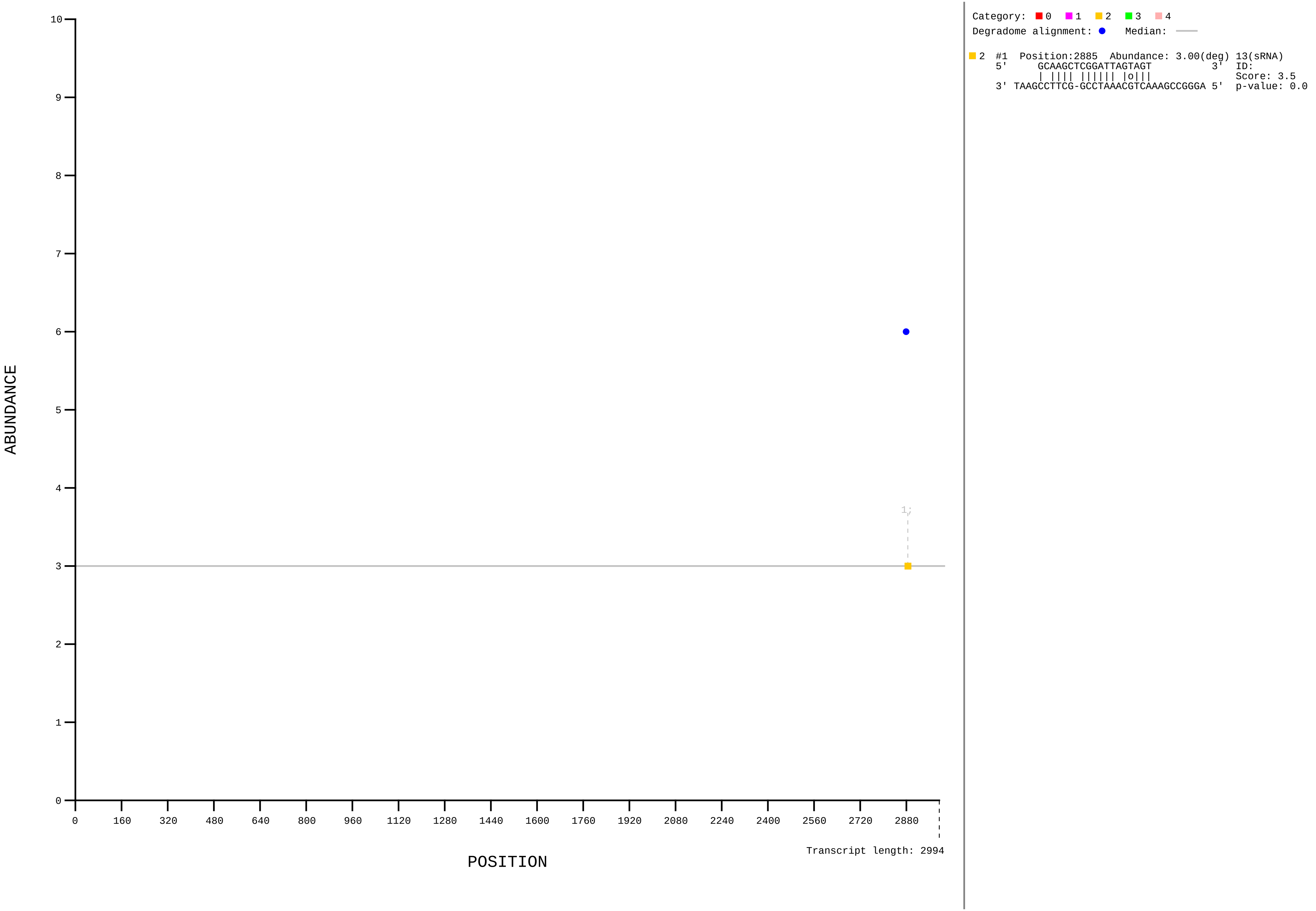

FOXG_09385T0 | Fusarium oxysporum f. sp. lycopersici 4287 Cwf15/Cwc15 cell cycle control family protein (714 nt)

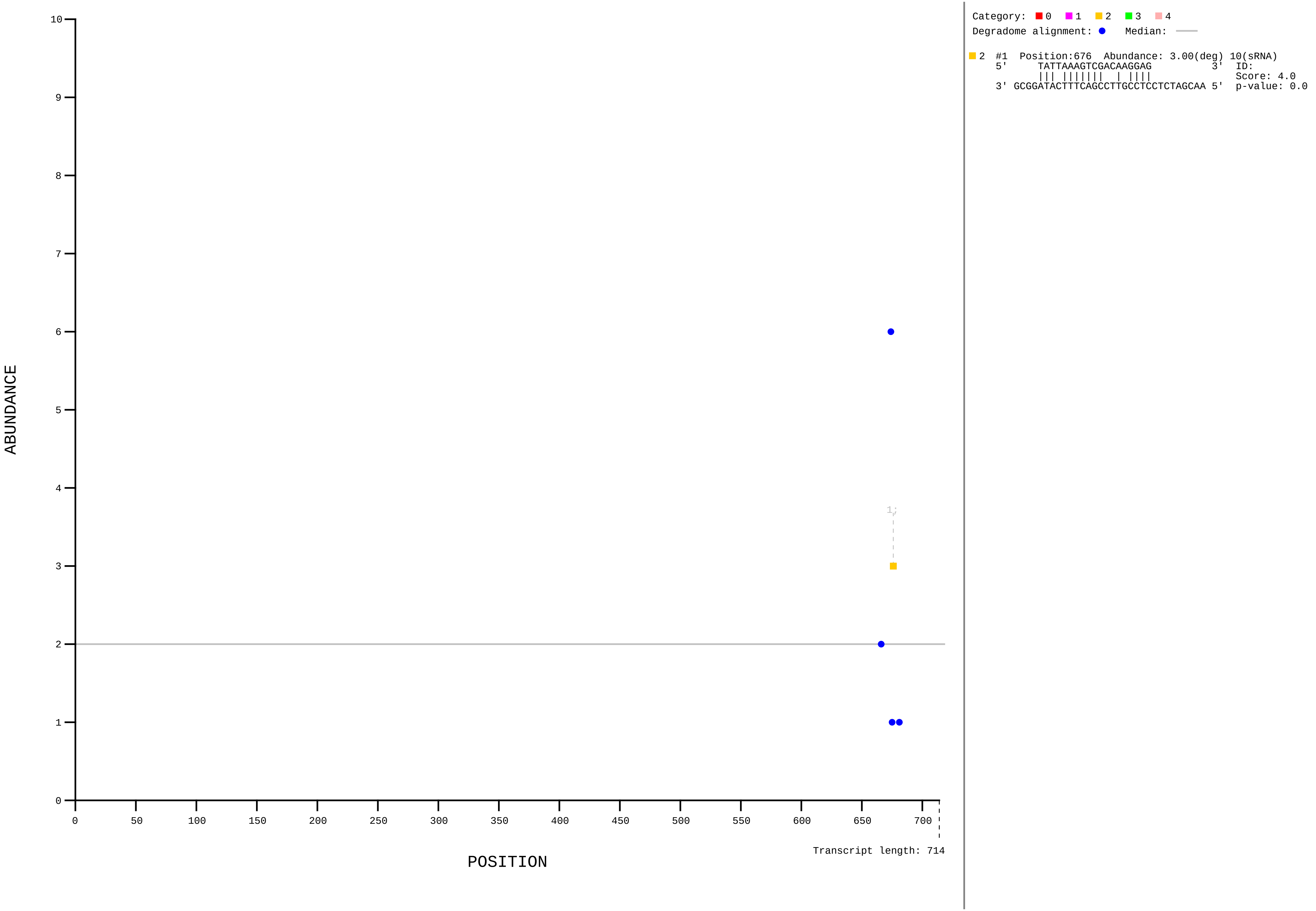

FOXG_07974T0 | Fusarium oxysporum f. sp. lycopersici 4287 hypothetical protein (1368 nt)

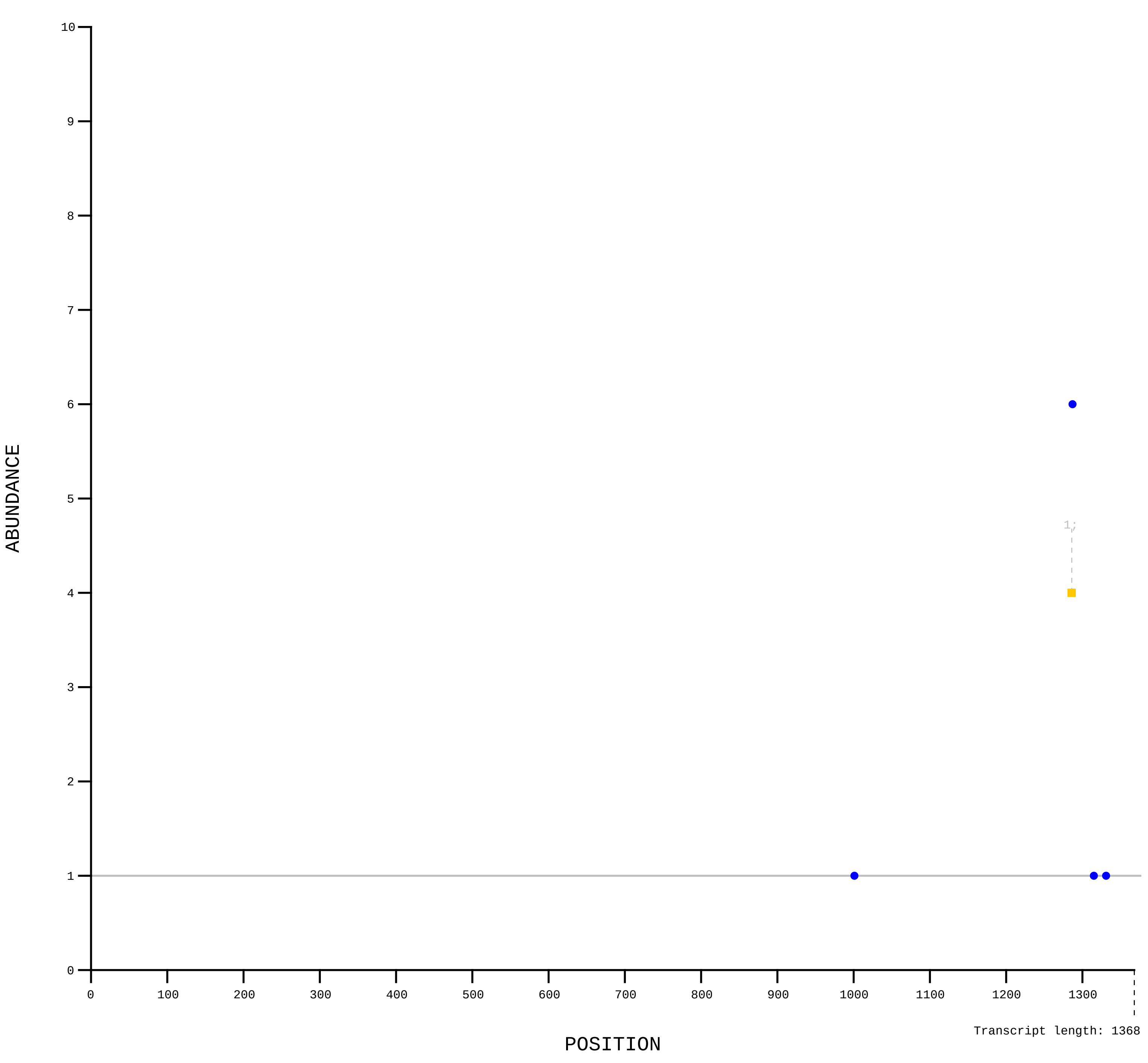

Category: 0 1 2 3 4
Degradome alignment: Median:

```
2  #1  Position:1286  Abundance: 4.00(deg) 8(sRNA)
   5'     TCCAAATTTCCAGGACTGA          3'  ID:
          |||||||||||| | ||                Score: 4.0
   3' TGGGAGGTTTAAAGGTACAGAGAGTTGTAGTA 5'  p-value: 0.02
```

FOXG_02750T0 | Fusarium oxysporum f. sp. lycopersici 4287 hypothetical protein (837 nt)

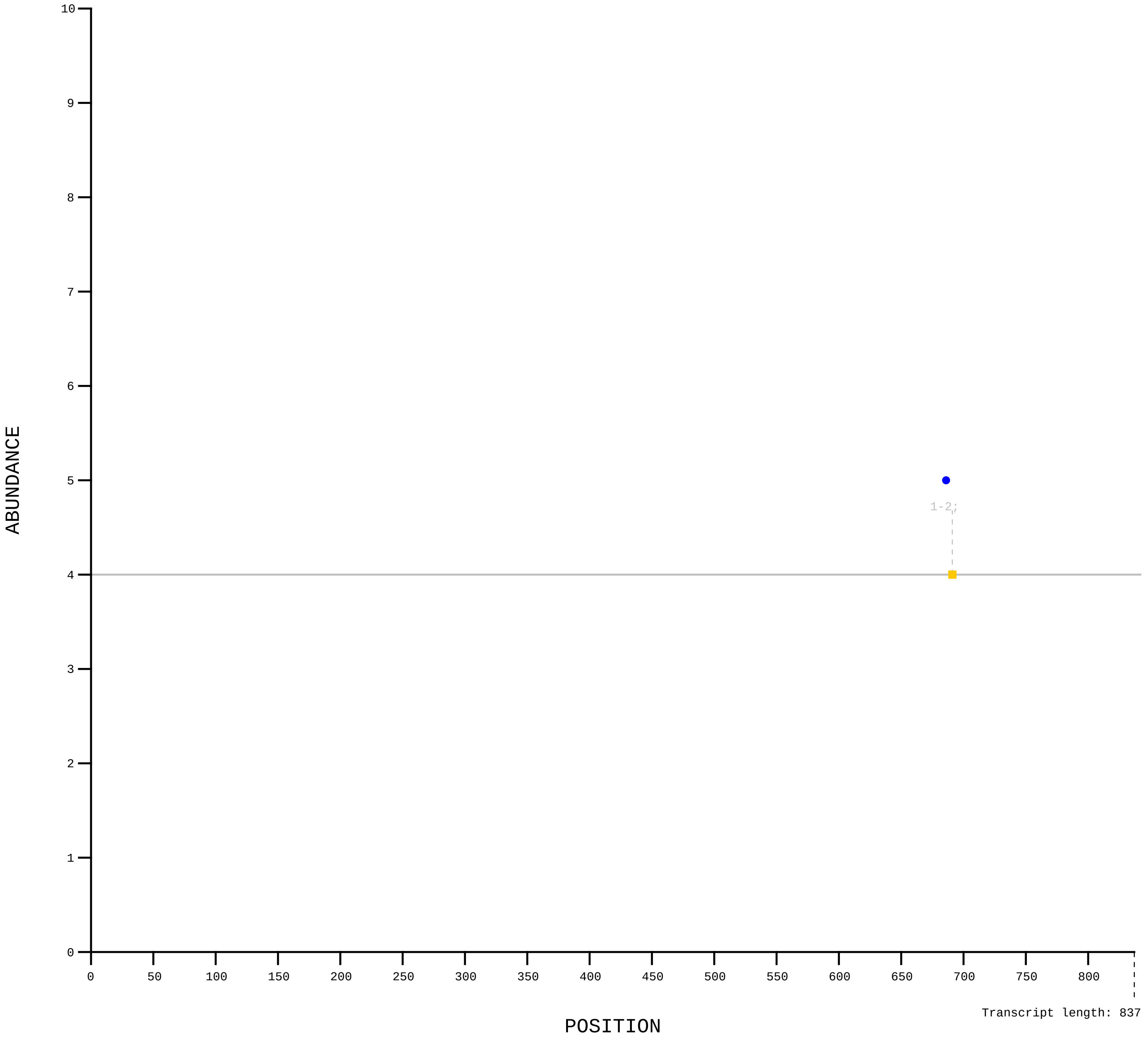


```
Category: 0 1 2 3 4
Degradome alignment:    Median:

2  #1  Position:691  Abundance: 4.00(deg) 12(sRNA)
   5'     TGAAGATTTTAGCAATGAAA         3'  ID:
          |o||| |o|||| |||| ||             Score: 4.0
   3' TCTAATTTCCAGAATCTTTACCTTCTGGTGCG 5'  p-value: 0.0

2  #2  Position:691  Abundance: 4.00(deg) 5(sRNA)
   5'     TAAGGGTCTTAGATTTTGG          3'  ID:
          |||o|||||||||  | ||              Score: 3.5
   3' TCTAATTTCCAGAATCTTTA-CCTTCTGGTGC 5'  p-value: 0.0
```

FOXG_05026T0 | Fusarium oxysporum f. sp. lycopersici 4287 hypothetical protein (1299 nt)

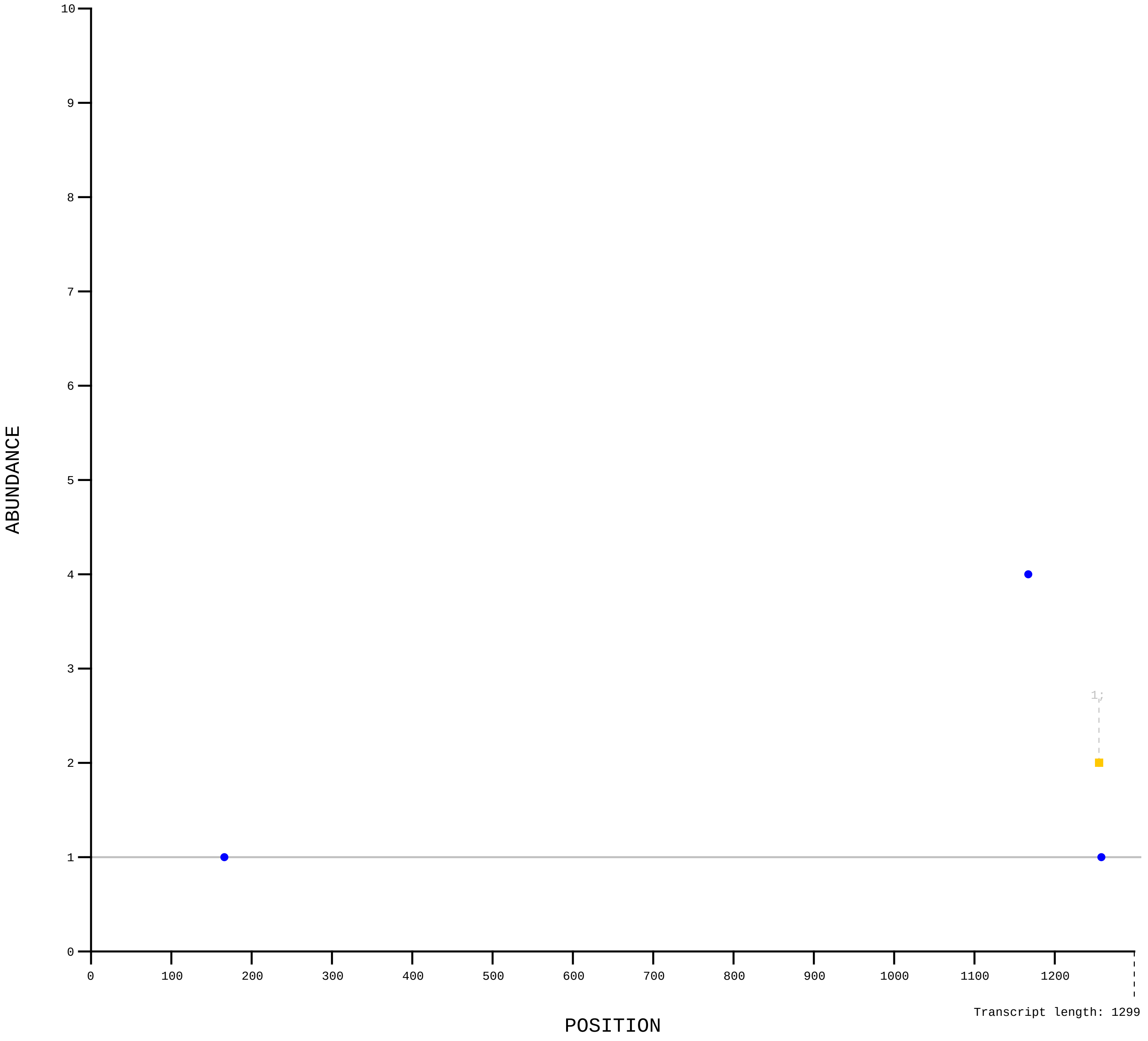

Category: 0 1 2 3 4
Degradome alignment: ● Median:

```
2  #1  Position:1255  Abundance: 2.00(deg) 5(sRNA)
   5'     CAAGCTAGAGCTAGACAACC         3'  ID:
            | || ||||||| |||| ||           Score: 4.0
   3' CTTGGGTCTATCTCGAGCTGT-GGTATGTTTT 5'  p-value: 0.0
```

FOXG_00863T0 | Fusarium oxysporum f. sp. lycopersici 4287 DNA-directed RNA polymerase N/8 kDa subu (truncated)

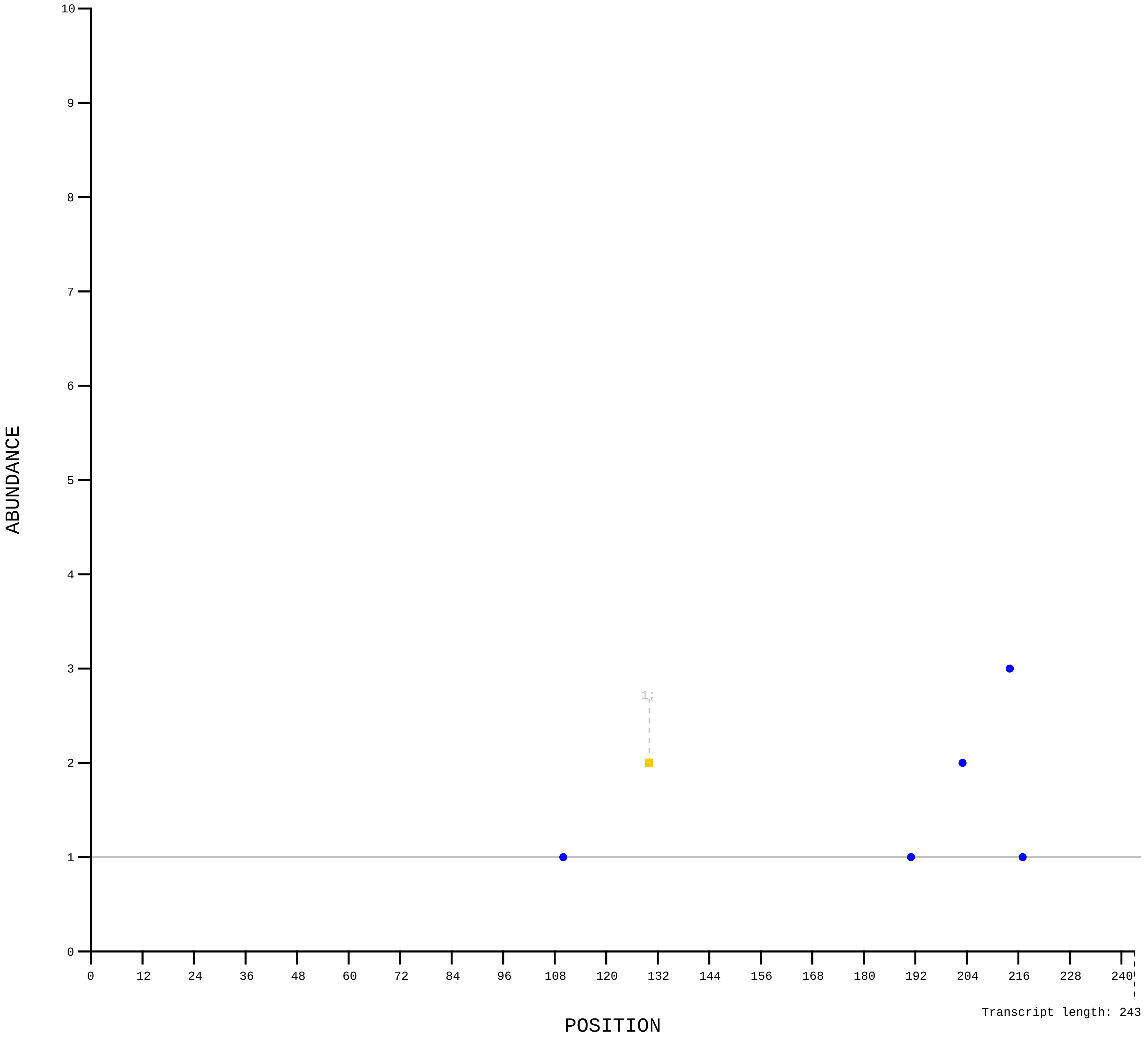

Category: 0 1 2 3 4
Degradome alignment: ● Median:

```
2  #1  Position:130  Abundance: 2.00(deg) 6(sRNA)
   5'     GGTAGCAGTATCAGATTTAGG        3'  ID:
          ||o|||||||||| | ||o||o           Score: 3.5
   3' GCCGCCGTCGTCATAG-CGAAGTCTGGCTCGA 5'  p-value: 0.0
```

FOXG_14002T0 | Fusarium oxysporum f. sp. lycopersici 4287 hypothetical protein (3153 nt) Note(s): (truncated)

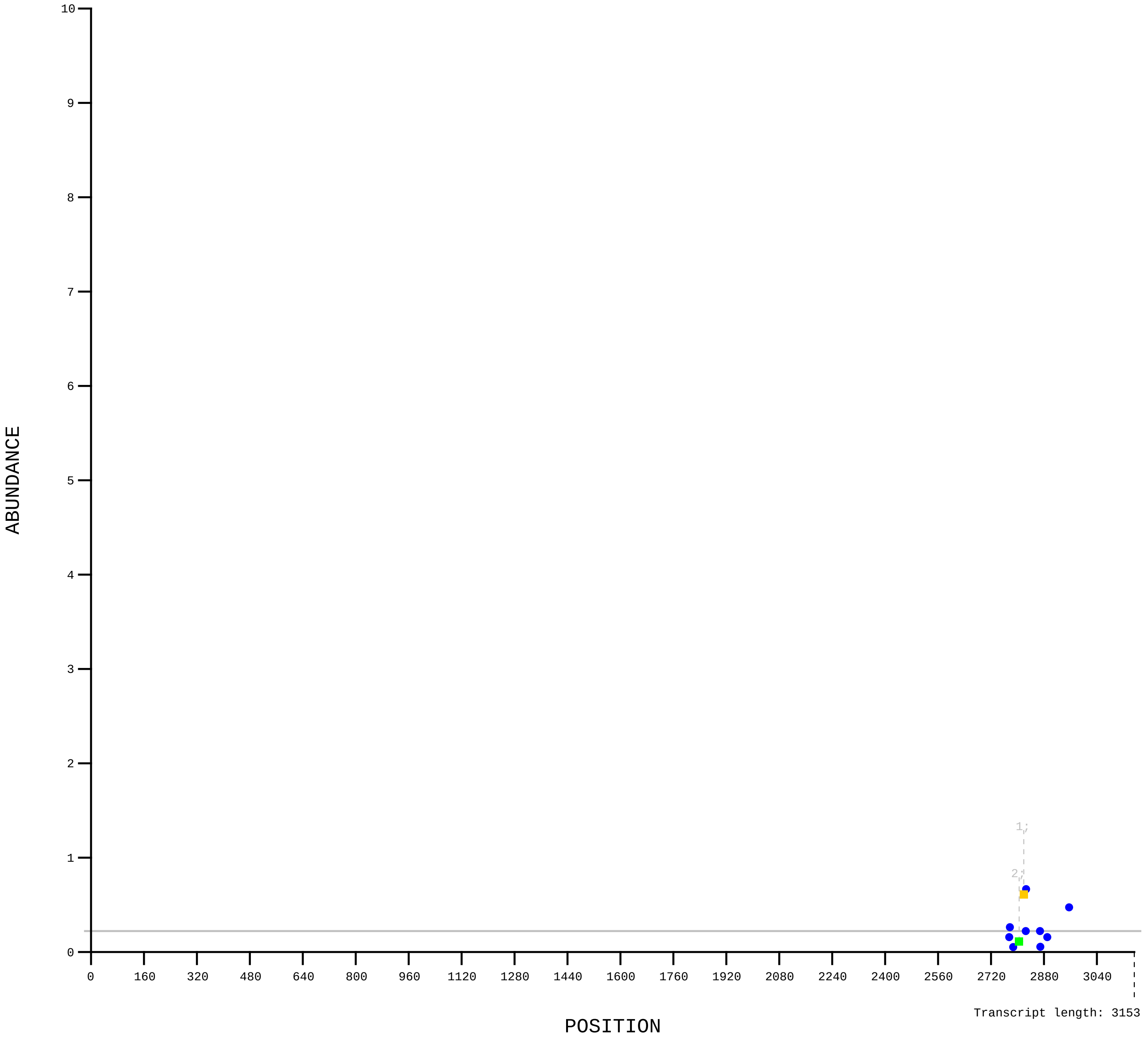

Category: 0 1 2 3 4
Degradome alignment: Median:

```
2  #1  Position:2819  Abundance: 0.61(deg) 10(sRNA)
   5'     TTTCATGATGAGATATCCA          3'  ID:
          || ||o|| ||||o| |||              Score: 4.0
   3' CAAAAATGTGCT-CTCTGTCGGTGCATCGAAT 5'  p-value: 0.03

3  #2  Position:2805  Abundance: 0.11(deg) 5(sRNA)
   5'     AGCCACGTAGCTTAAACCGGTC       3'  ID:
          ||||||||||||||||||||||           Score: 0.0
   3' TCTGTCGGTGCATCGAATTTGGCCAGACTTTC 5'  p-value: 0.0
```

FOXG_03947T0 | Fusarium oxysporum f. sp. lycopersici 4287 hypothetical protein (1143 nt)

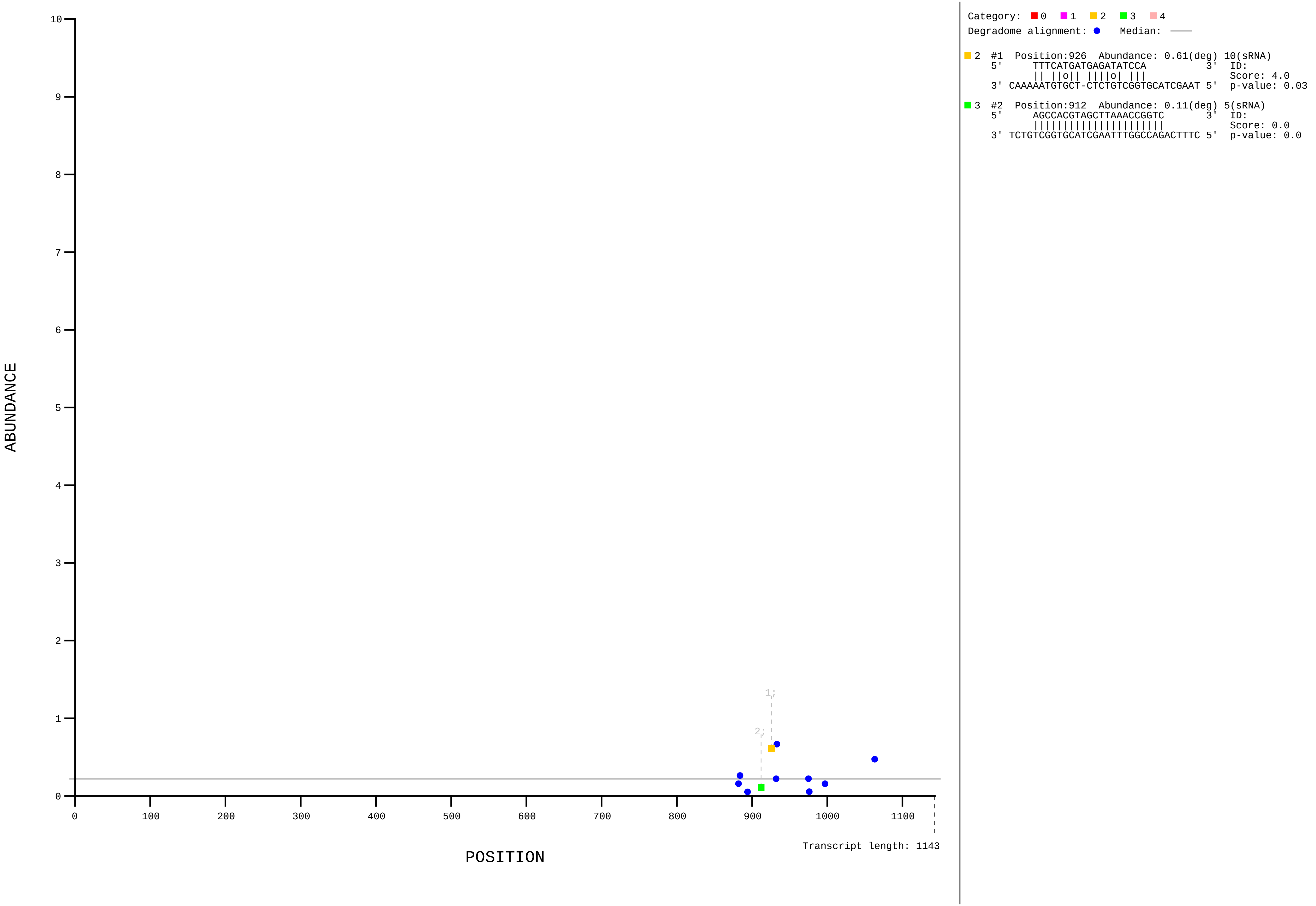

FOXG_16423T0 | Fusarium oxysporum f. sp. lycopersici 4287 hypothetical protein (4365 nt)

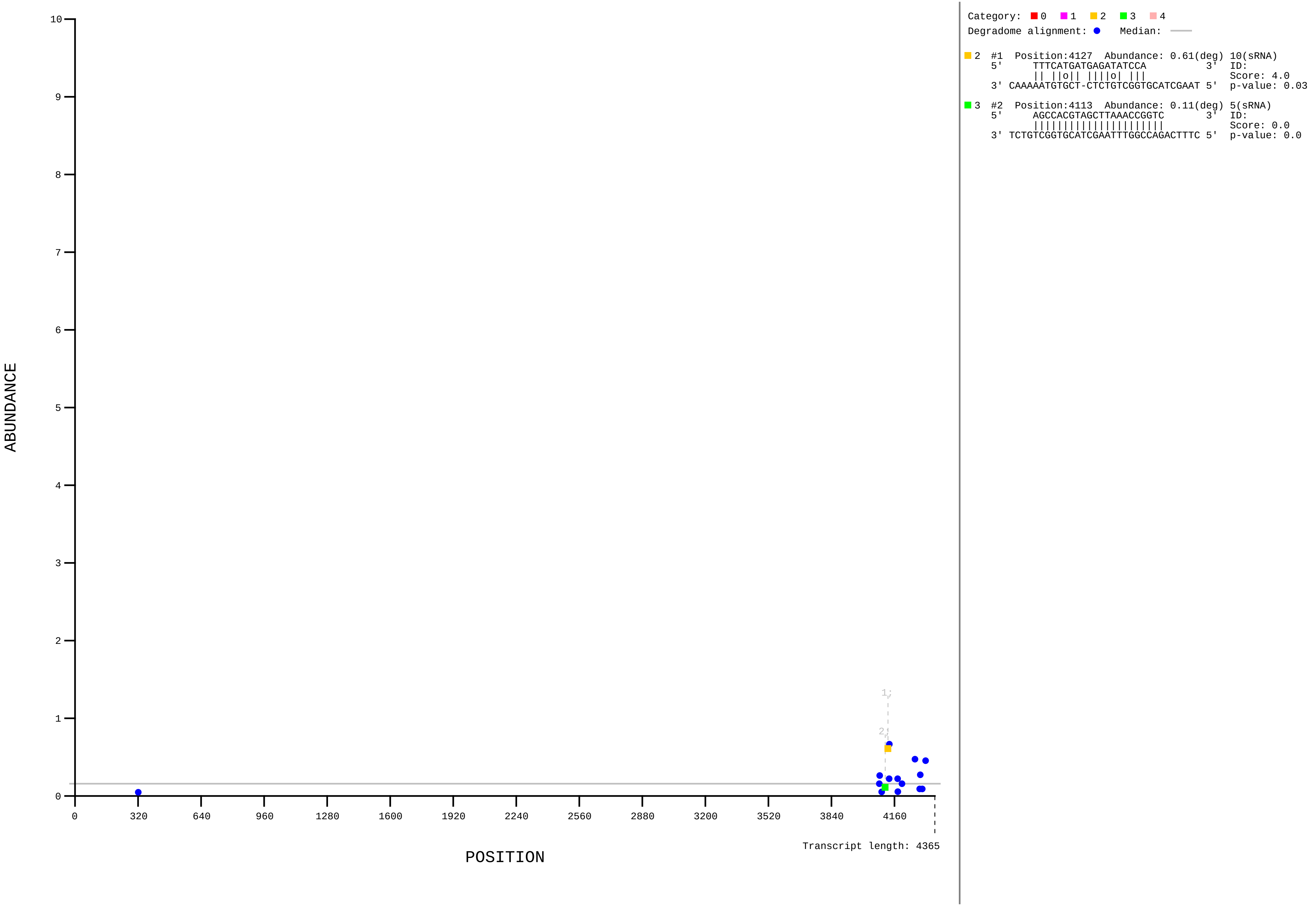

FOXG_16413T0 | Fusarium oxysporum f. sp. lycopersici 4287 hypothetical protein (5721 nt)

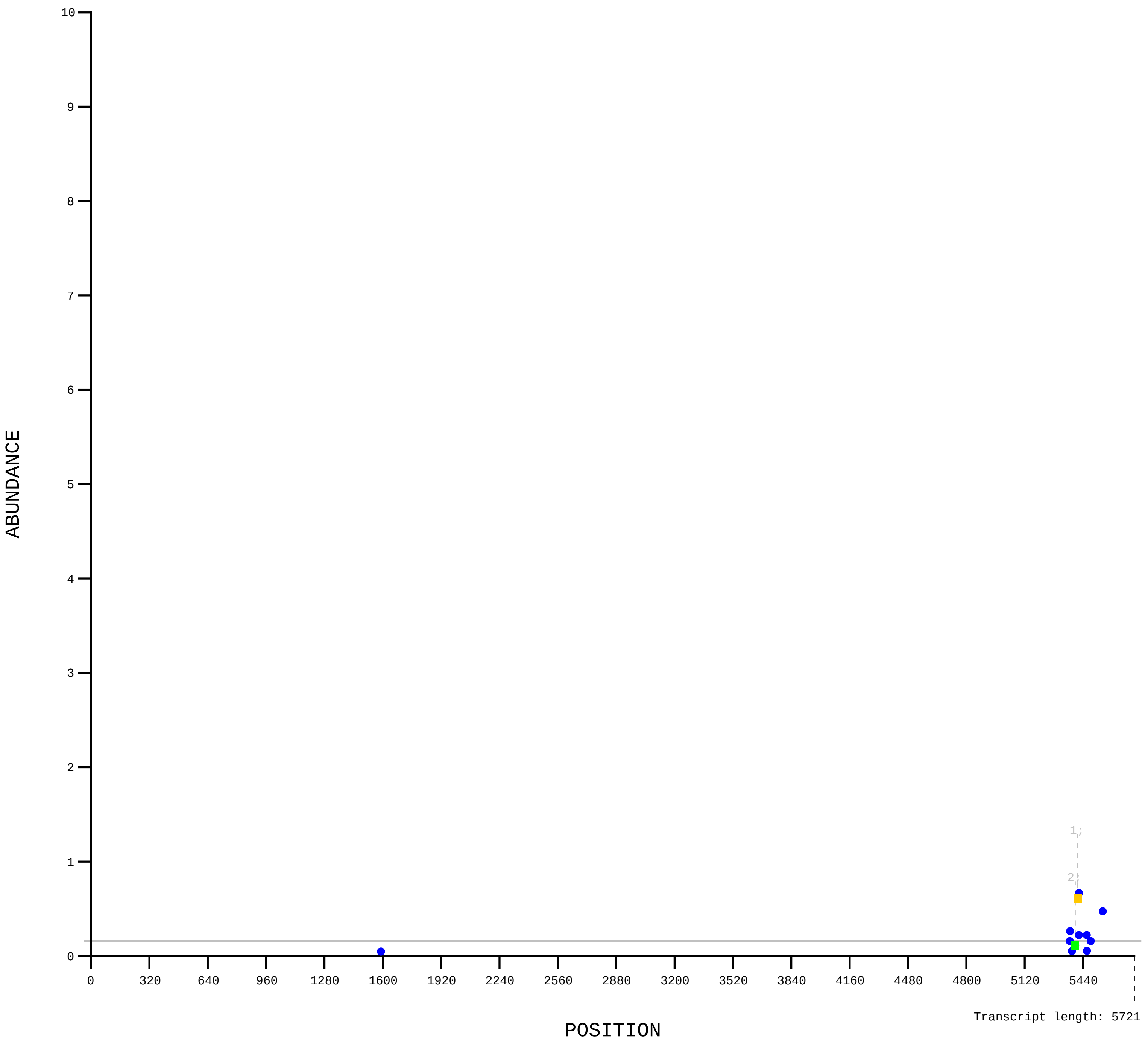


```
Category: 0 1 2 3 4
Degradome alignment:    Median:

2  #1  Position:5411  Abundance: 0.61(deg) 10(sRNA)
   5'      TTTCATGATGAGATATCCA          3'  ID:
           || ||o|| ||||o| |||              Score: 4.0
   3' CAAAAATGTGCT-CTCTGTCGGTGCATCGAAT 5'  p-value: 0.0

3  #2  Position:5397  Abundance: 0.11(deg) 5(sRNA)
   5'      AGCCACGTAGCTTAAACCGGTC       3'  ID:
           ||||||||||||||||||||||           Score: 0.0
   3' TCTGTCGGTGCATCGAATTTGGCCAGACTTTC 5'  p-value: 0.0
```

FOXG_05786T0 | Fusarium oxysporum f. sp. lycopersici 4287 hypothetical protein (5532 nt) Note(s): (truncated)

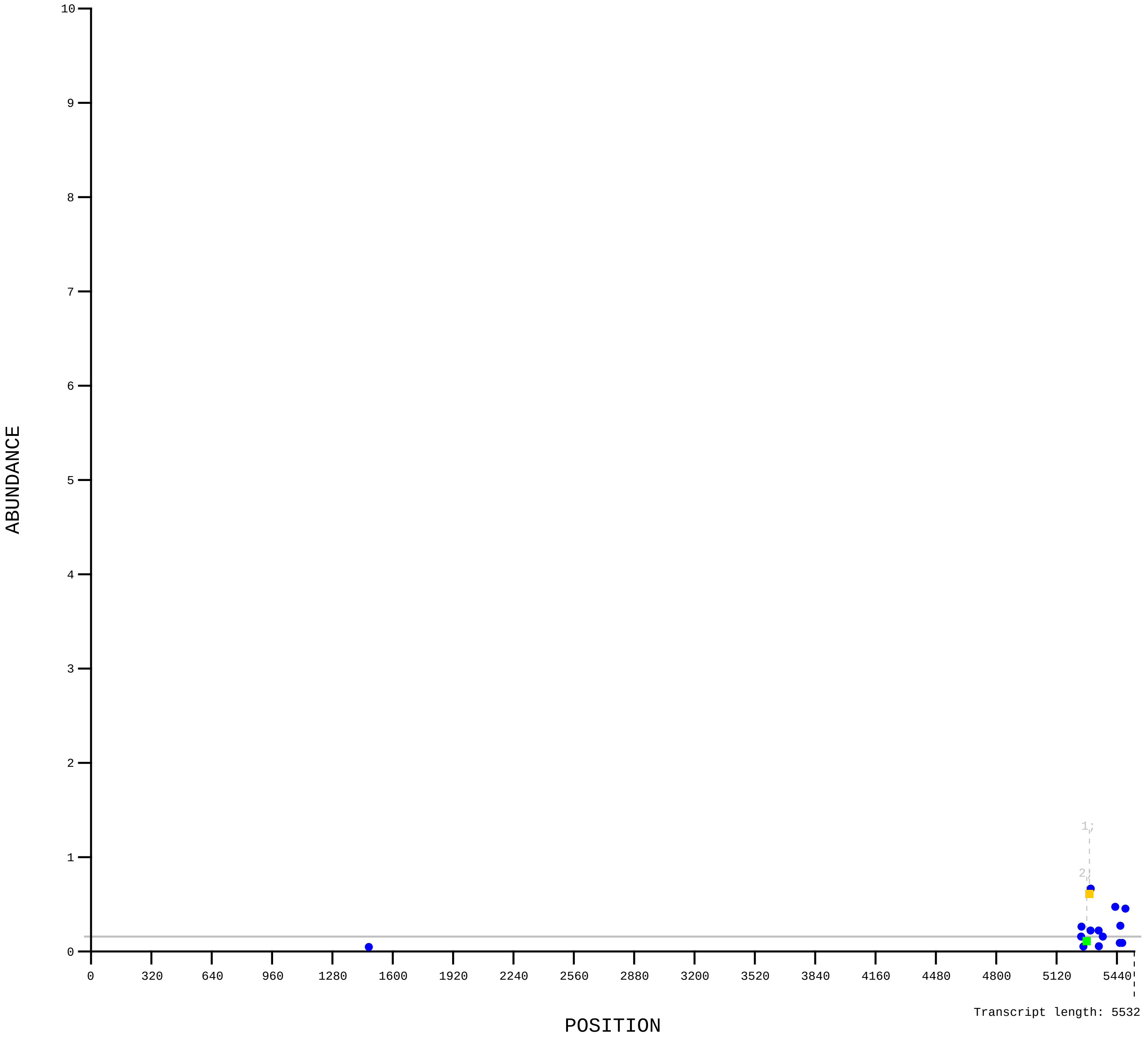

Category: 0 1 2 3 4
Degradome alignment: Median:

```
2  #1  Position:5294  Abundance: 0.61(deg) 10(sRNA)
   5'     TTTCATGATGAGATATCCA          3'  ID:
          || ||o|| ||||o| |||              Score: 4.0
   3' CAAAAATGTGCT-CTCTGTCGGTGCATCGAAT 5'  p-value: 0.0

3  #2  Position:5280  Abundance: 0.11(deg) 5(sRNA)
   5'     AGCCACGTAGCTTAAACCGGTC       3'  ID:
          ||||||||||||||||||||||           Score: 0.0
   3' TCTGTCGGTGCATCGAATTTGGCCAGACTTTC 5'  p-value: 0.0
```

FOXG_17286T0 | Fusarium oxysporum f. sp. lycopersici 4287 hypothetical protein (4365 nt) Note(s): (truncated)

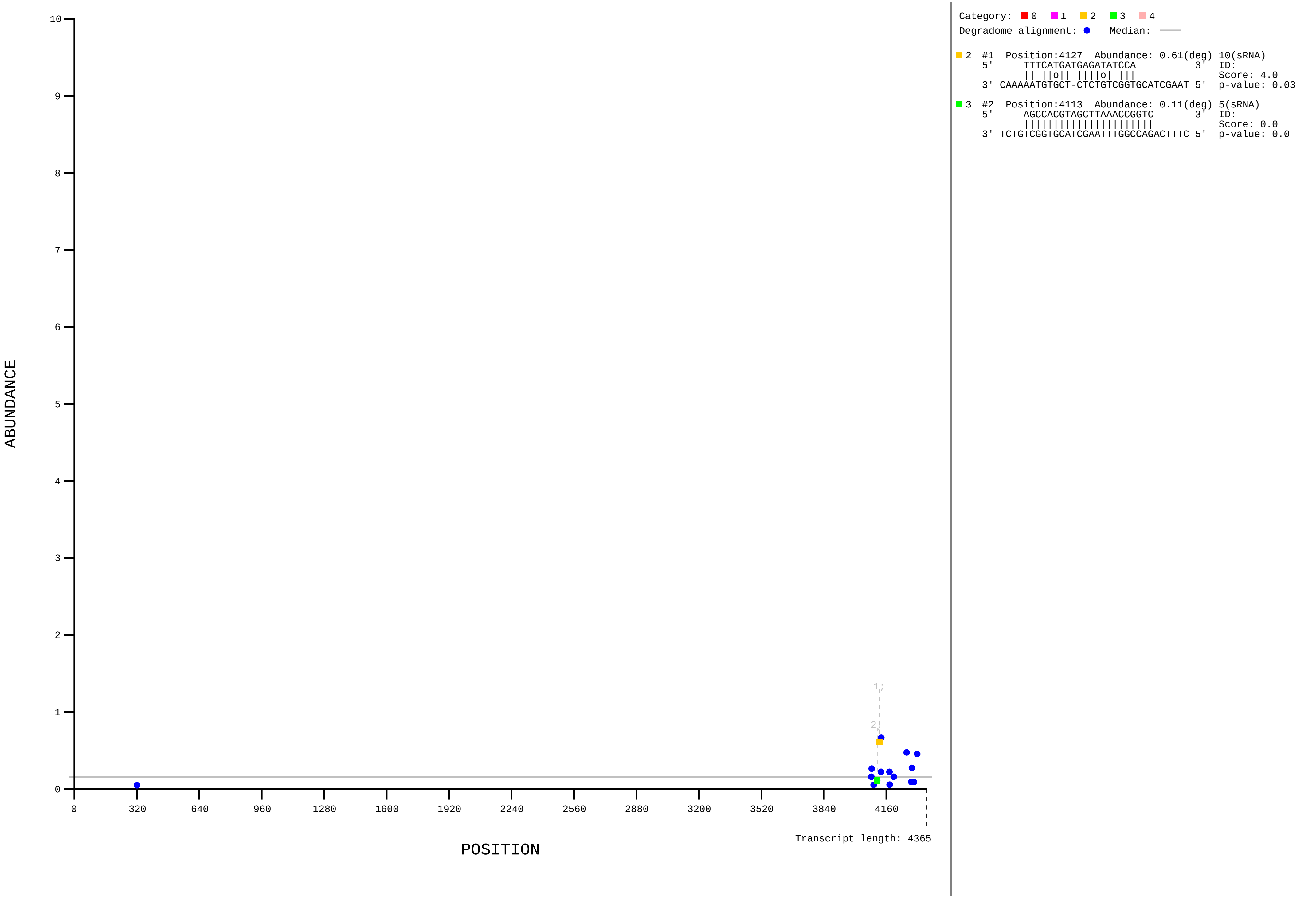

FOXG_17086T0 | Fusarium oxysporum f. sp. lycopersici 4287 hypothetical protein (3021 nt)

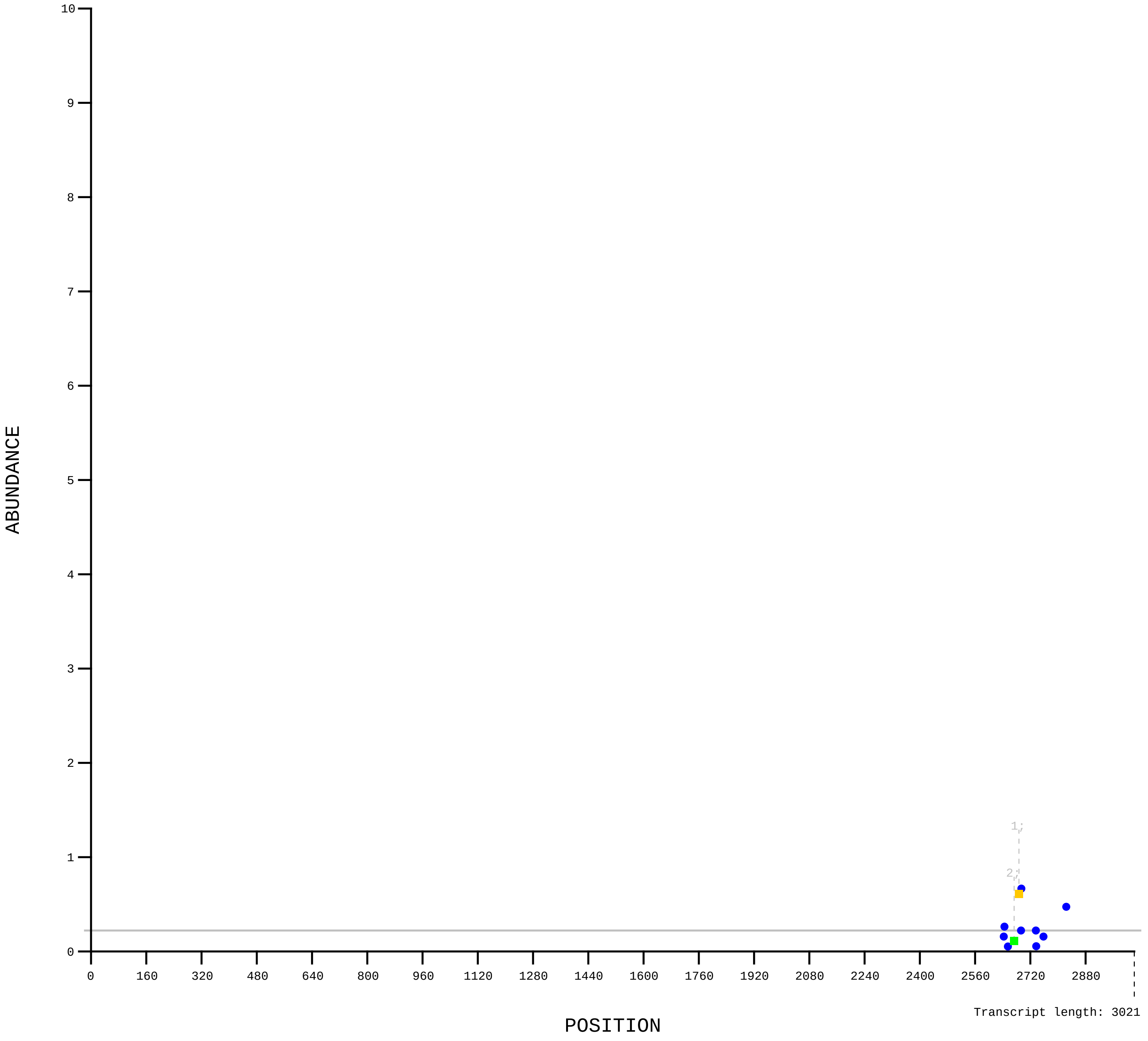

Category: 0 1 2 3 4
Degradome alignment: ● Median: —

```
2  #1  Position:2687  Abundance: 0.61(deg) 10(sRNA)
   5'     TTTCATGATGAGATATCCA          3'  ID:
          || ||o|| ||||o| |||              Score: 4.0
   3' CAAAAATGTGCT-CTCTGTCGGTGCATCGAAT 5'  p-value: 0.02

3  #2  Position:2673  Abundance: 0.11(deg) 5(sRNA)
   5'     AGCCACGTAGCTTAAACCGGTC       3'  ID:
          ||||||||||||||||||||||           Score: 0.0
   3' TCTGTCGGTGCATCGAATTTGGCCAGACTTTC 5'  p-value: 0.0
```

FOXG_16419T0 | Fusarium oxysporum f. sp. lycopersici 4287 hypothetical protein (4425 nt) Note(s): (truncated)

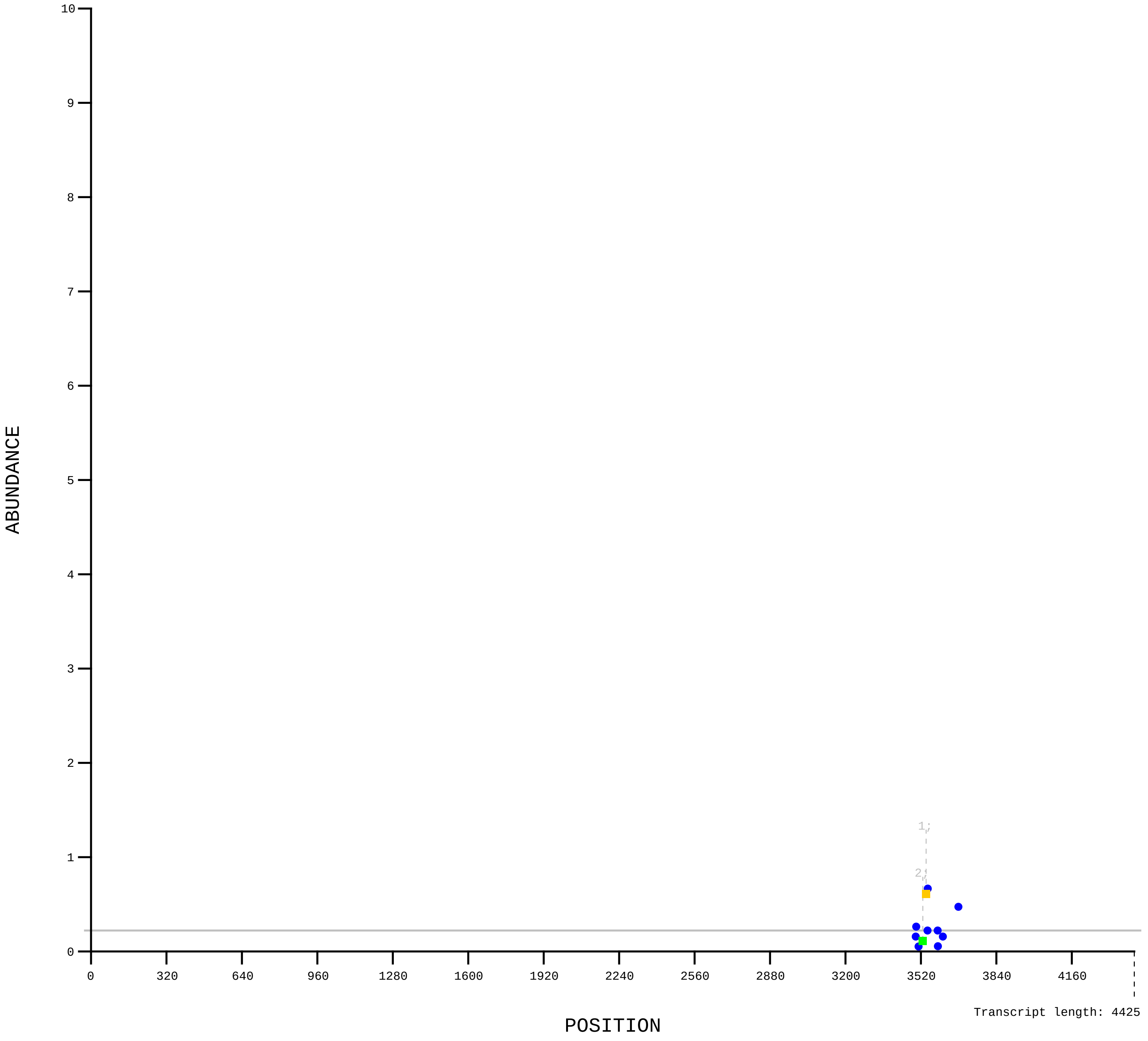

Category: 0 1 2 3 4
Degradome alignment: ● Median: —

```
2  #1  Position:3542  Abundance: 0.61(deg) 10(sRNA)
   5'     TTTCATGATGAGATATCCA          3'  ID:
          || ||o|| ||||o| |||              Score: 4.0
   3' CAAAAATGTGCT-CTCTGTCGGTGCATCGAAT 5'  p-value: 0.01

3  #2  Position:3528  Abundance: 0.11(deg) 5(sRNA)
   5'     AGCCACGTAGCTTAAACCGGTC       3'  ID:
          ||||||||||||||||||||||           Score: 0.0
   3' TCTGTCGGTGCATCGAATTTGGCCAGACTTTC 5'  p-value: 0.0
```

FOXG_14236T0 | Fusarium oxysporum f. sp. lycopersici 4287 hypothetical protein (6120 nt) Note(s): (truncated)

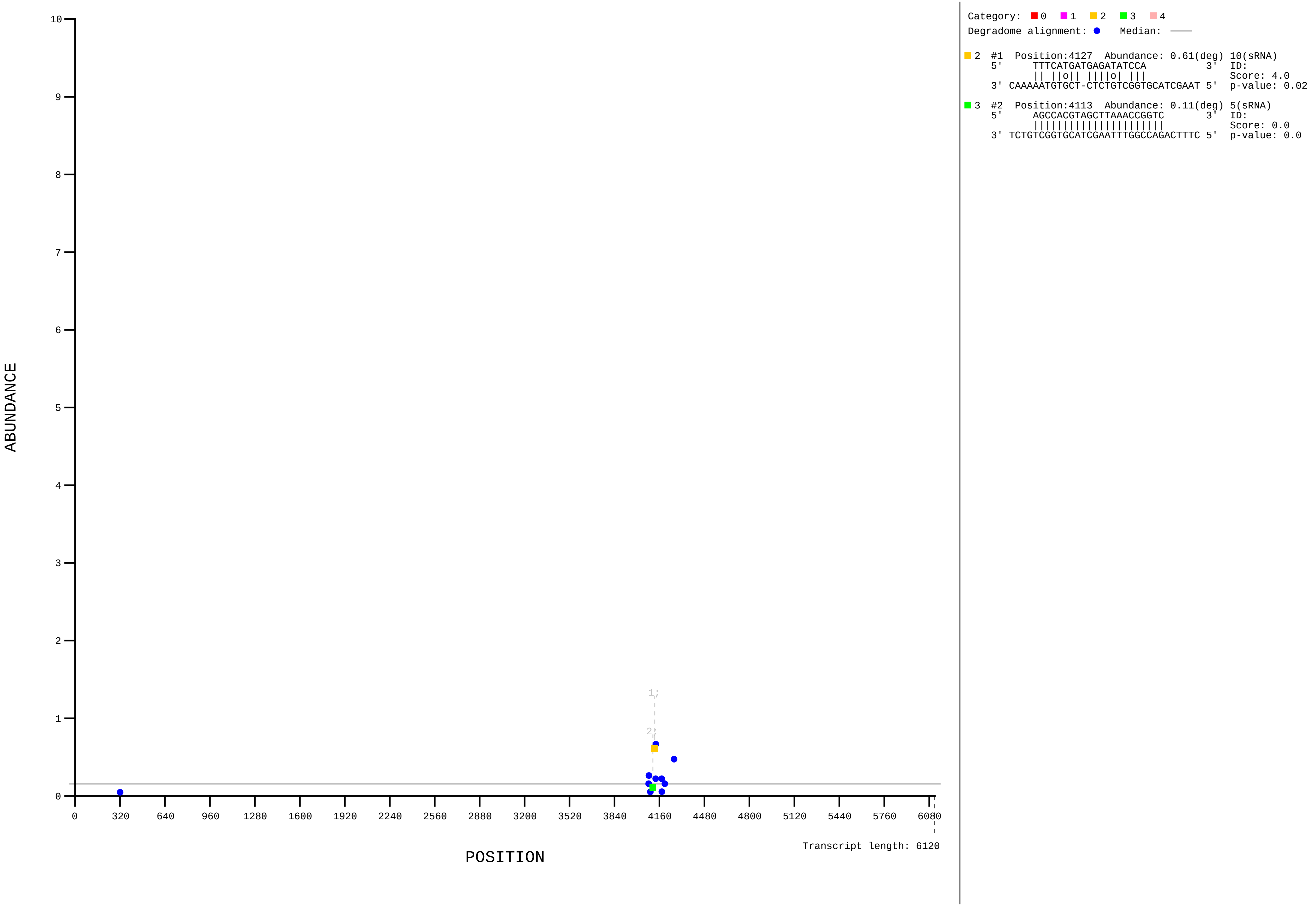

FOXG_10736T0 | Fusarium oxysporum f. sp. lycopersici 4287 hypothetical protein (5193 nt) Note(s): (truncated)

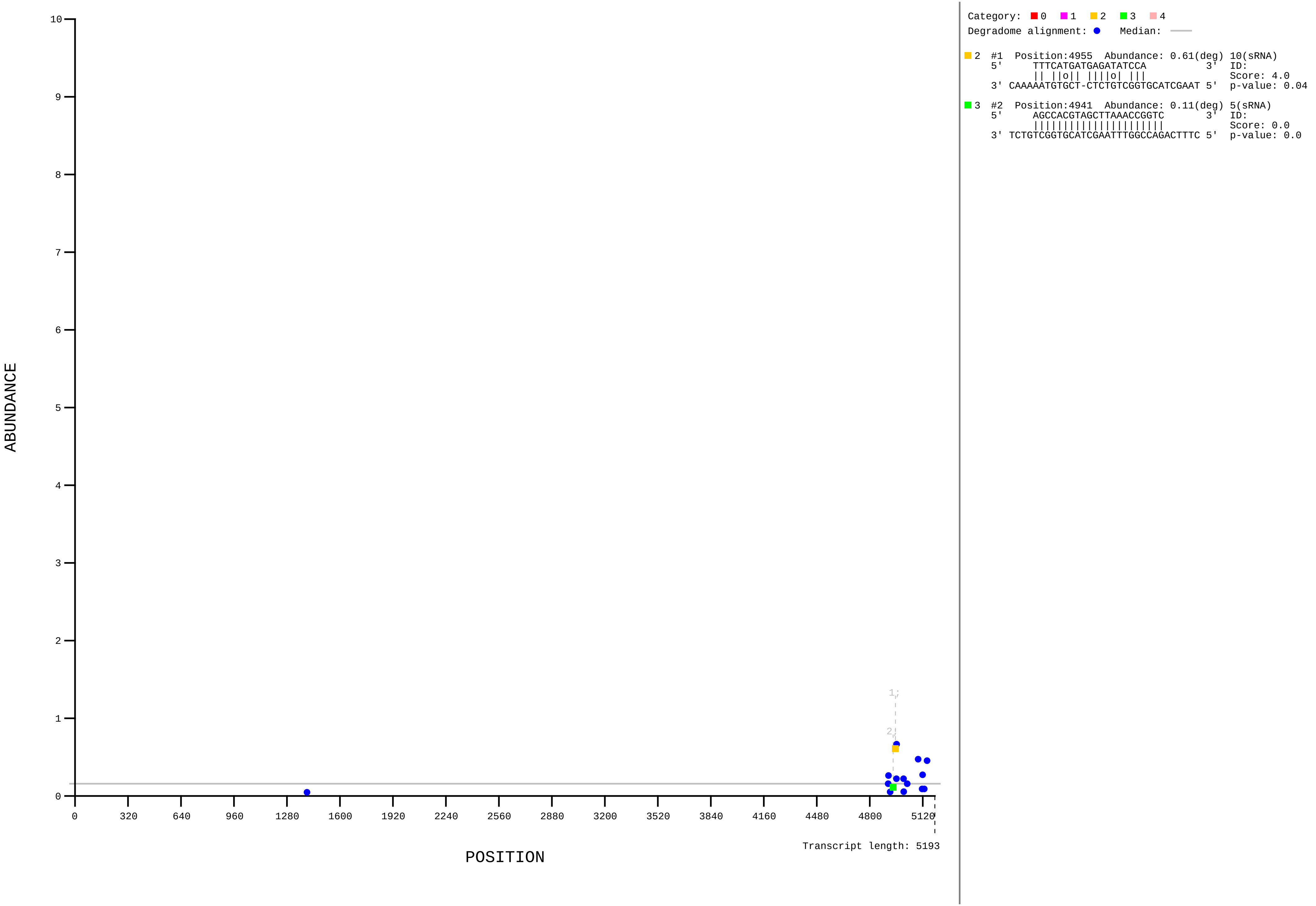

FOXG_17273T0 | Fusarium oxysporum f. sp. lycopersici 4287 hypothetical protein (4380 nt)

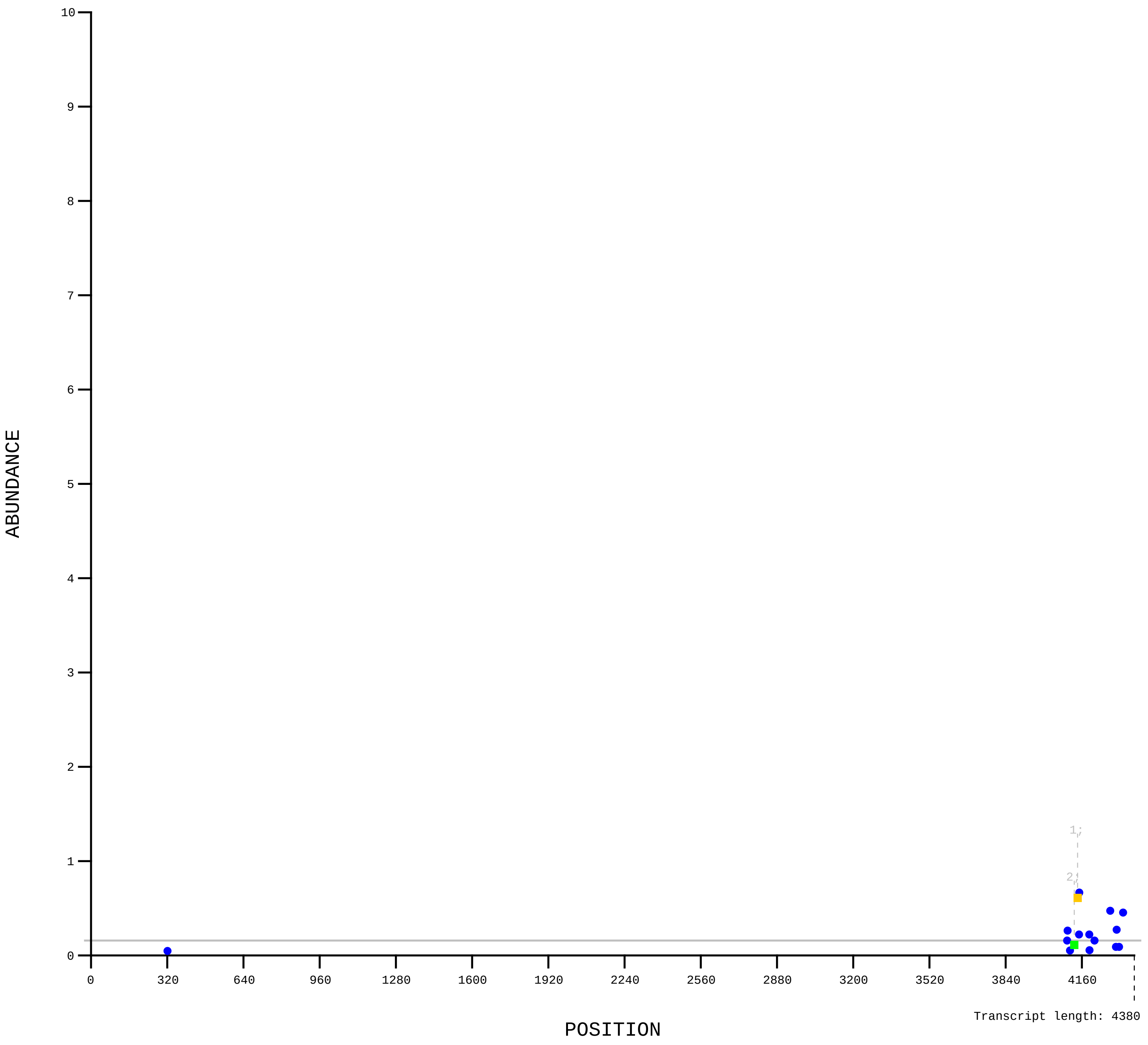

Category: 0 1 2 3 4
Degradome alignment: Median:

```
2  #1  Position:4142  Abundance: 0.61(deg) 10(sRNA)
   5'     TTTCATGATGAGATATCCA          3'  ID:
          || ||o|| ||||o| |||              Score: 4.0
   3' CAAAAATGTGCT-CTCTGTCGGTGCATCGAAT 5'  p-value: 0.02

3  #2  Position:4128  Abundance: 0.11(deg) 5(sRNA)
   5'     AGCCACGTAGCTTAAACCGGTC       3'  ID:
          ||||||||||||||||||||||           Score: 0.0
   3' TCTGTCGGTGCATCGAATTTGGCCAGACTTTC 5'  p-value: 0.0
```

FOXG_04036T0 | Fusarium oxysporum f. sp. lycopersici 4287 hypothetical protein (5544 nt)

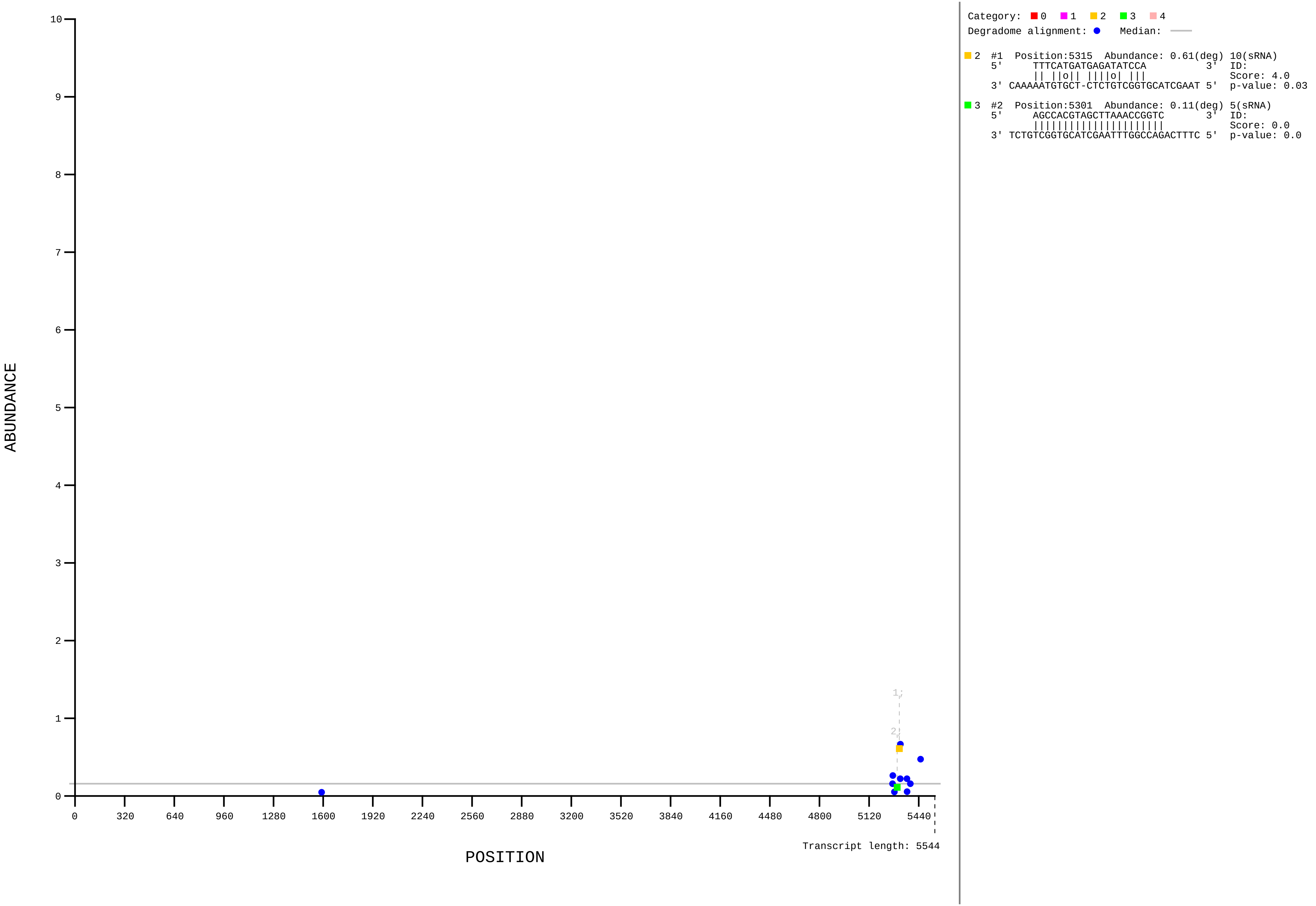

FOXG_14245T0 | Fusarium oxysporum f. sp. lycopersici 4287 hypothetical protein (5220 nt) Note(s): (truncated)

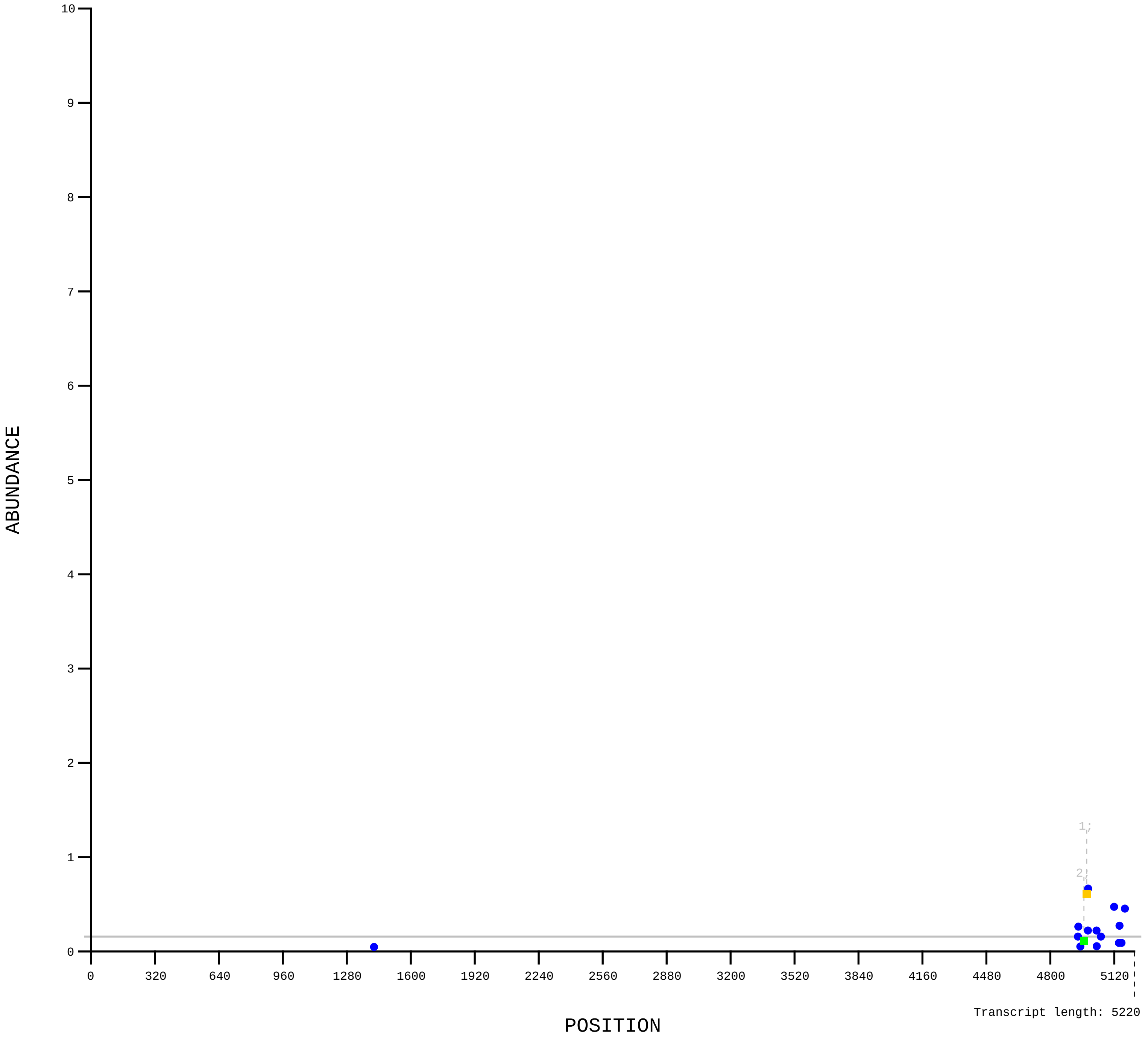

Category: 0 1 2 3 4
Degradome alignment: Median:

```
2  #1  Position:4982  Abundance: 0.61(deg) 10(sRNA)
   5'     TTTCATGATGAGATATCCA          3'  ID:
          || ||o|| ||||o| |||              Score: 4.0
   3' CAAAAATGTGCT-CTCTGTCGGTGCATCGAAT 5'  p-value: 0.01

3  #2  Position:4968  Abundance: 0.11(deg) 5(sRNA)
   5'     AGCCACGTAGCTTAAACCGGTC       3'  ID:
          ||||||||||||||||||||||           Score: 0.0
   3' TCTGTCGGTGCATCGAATTTGGCCAGACTTTC 5'  p-value: 0.0
```

FOXG_12587T0 | Fusarium oxysporum f. sp. lycopersici 4287 hypothetical protein (2925 nt) Note(s): (truncated)

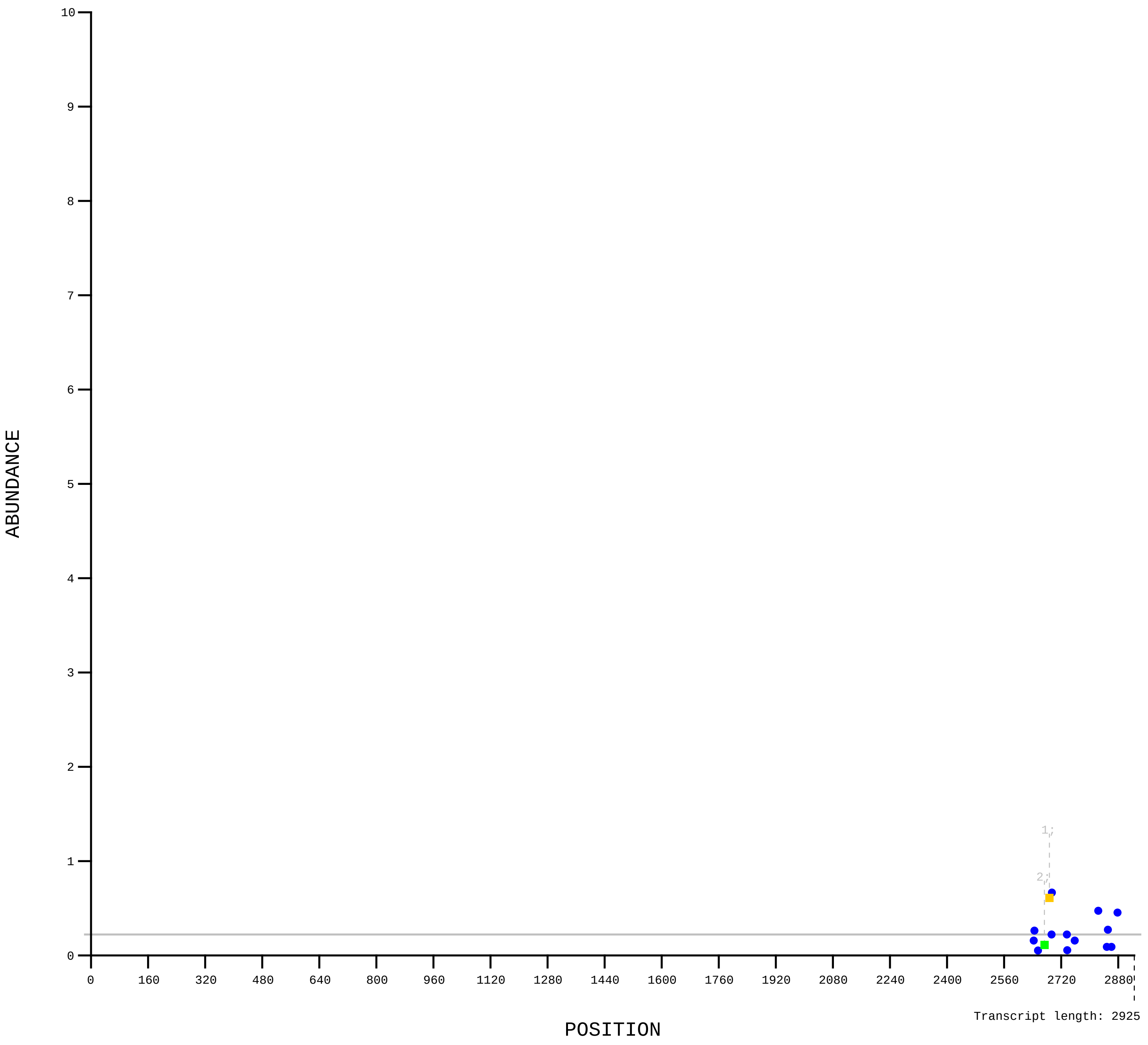


```
2  #1  Position:2687  Abundance: 0.61(deg) 10(sRNA)
   5'      TTTCATGATGAGATATCCA          3'  ID:
           || ||o|| ||||o| |||              Score: 4.0
   3' CAAAAATGTGCT-CTCTGTCGGTGCATCGAAT 5'  p-value: 0.01

3  #2  Position:2673  Abundance: 0.11(deg) 5(sRNA)
   5'      AGCCACGTAGCTTAAACCGGTC       3'  ID:
           ||||||||||||||||||||||           Score: 0.0
   3' TCTGTCGGTGCATCGAATTTGGCCAGACTTTC 5'  p-value: 0.0
```

FOXG_12537T0 | Fusarium oxysporum f. sp. lycopersici 4287 hypothetical protein (3030 nt) Note(s): (truncated)

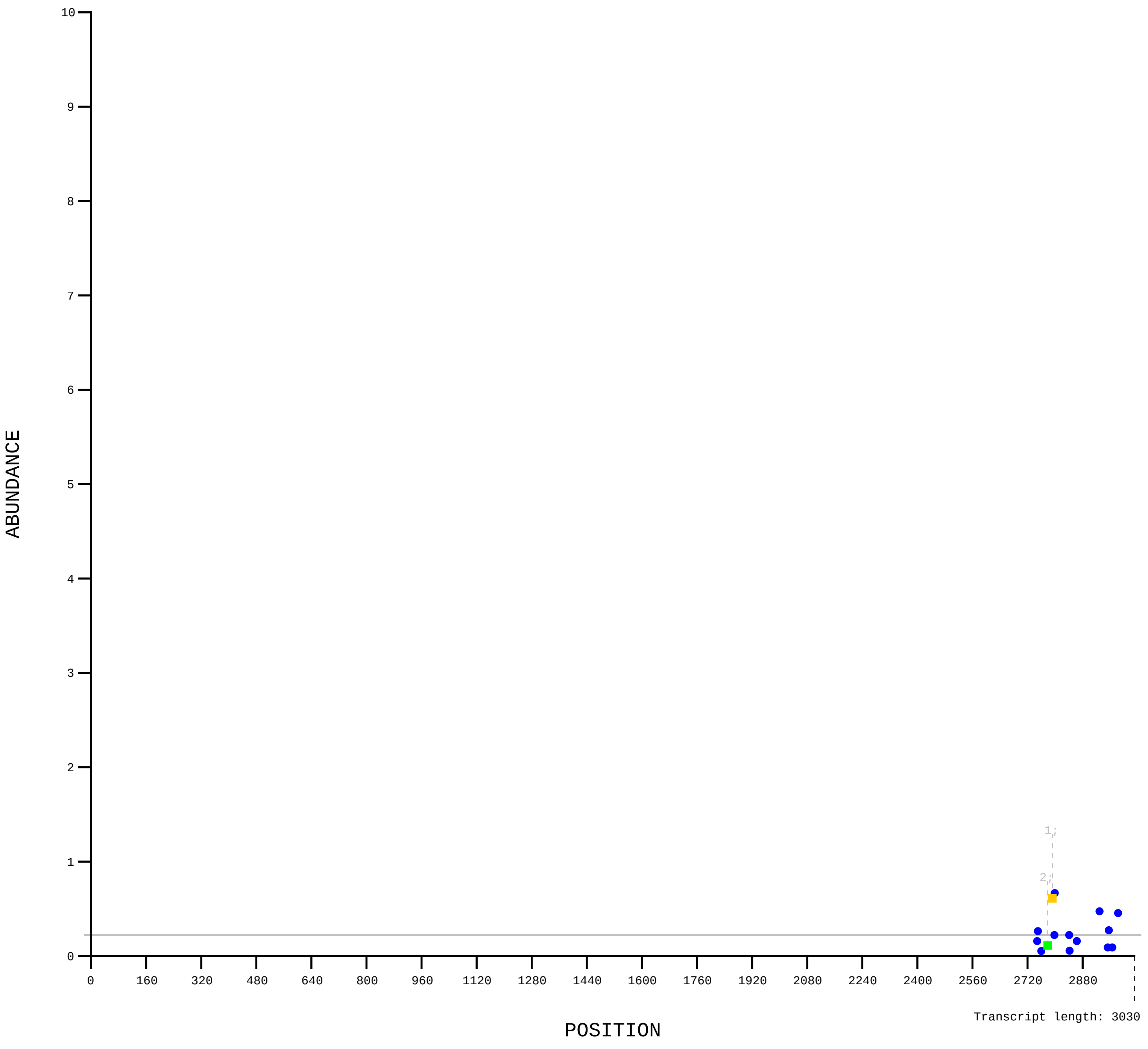


```
2  #1  Position:2792  Abundance: 0.61(deg) 10(sRNA)
   5'     TTTCATGATGAGATATCCA          3'  ID:
          || ||o|| ||||o| |||              Score: 4.0
   3' CAAAAATGTGCT-CTCTGTCGGTGCATCGAAT 5'  p-value: 0.03

3  #2  Position:2778  Abundance: 0.11(deg) 5(sRNA)
   5'     AGCCACGTAGCTTAAACCGGTC       3'  ID:
          ||||||||||||||||||||||           Score: 0.0
   3' TCTGTCGGTGCATCGAATTTGGCCAGACTTTC 5'  p-value: 0.0
```

FOXG_17427T0 | Fusarium oxysporum f. sp. lycopersici 4287 hypothetical protein (5424 nt)

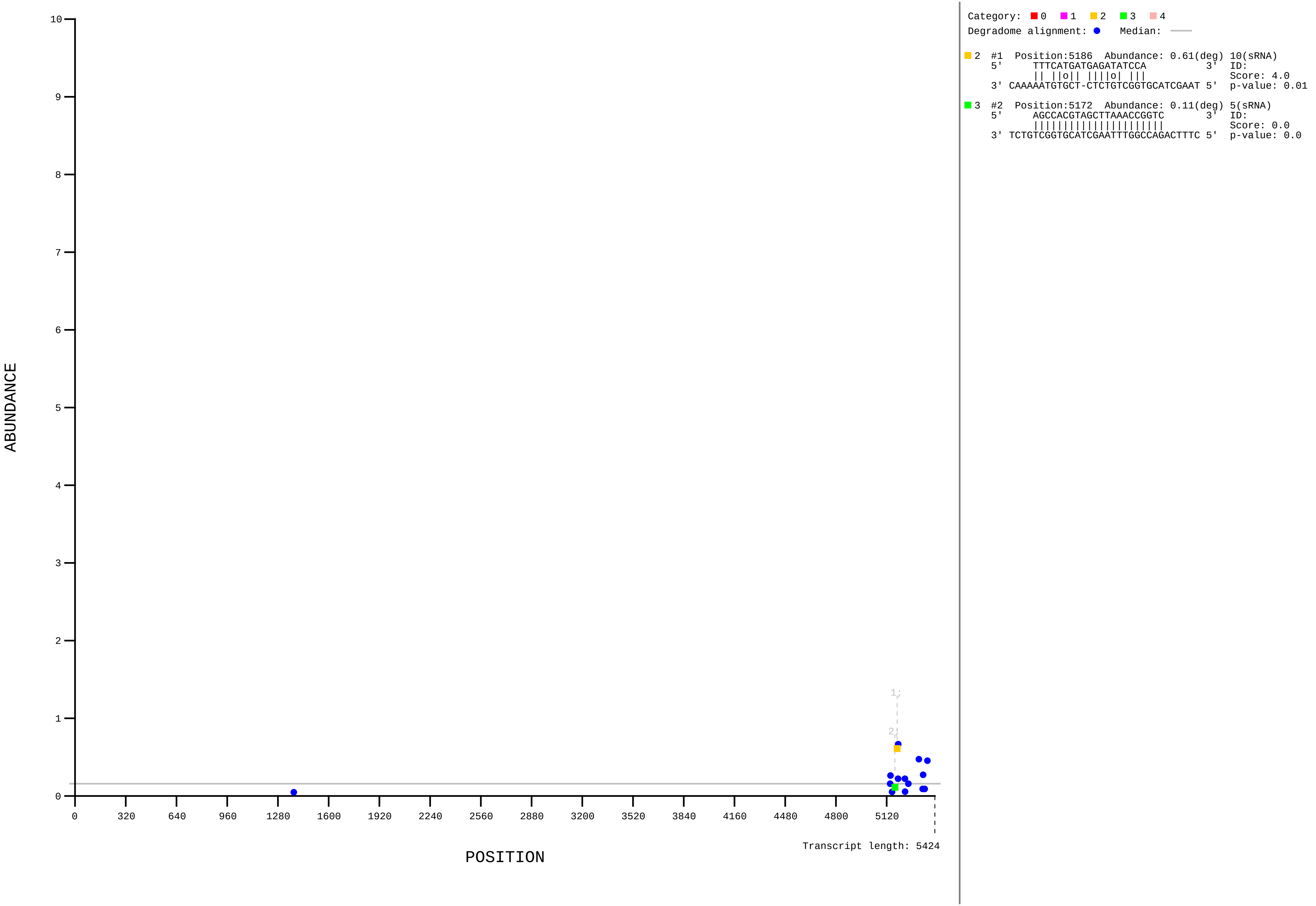

FOXG_05783T0 | Fusarium oxysporum f. sp. lycopersici 4287 hypothetical protein (5649 nt) Note(s): (truncated)

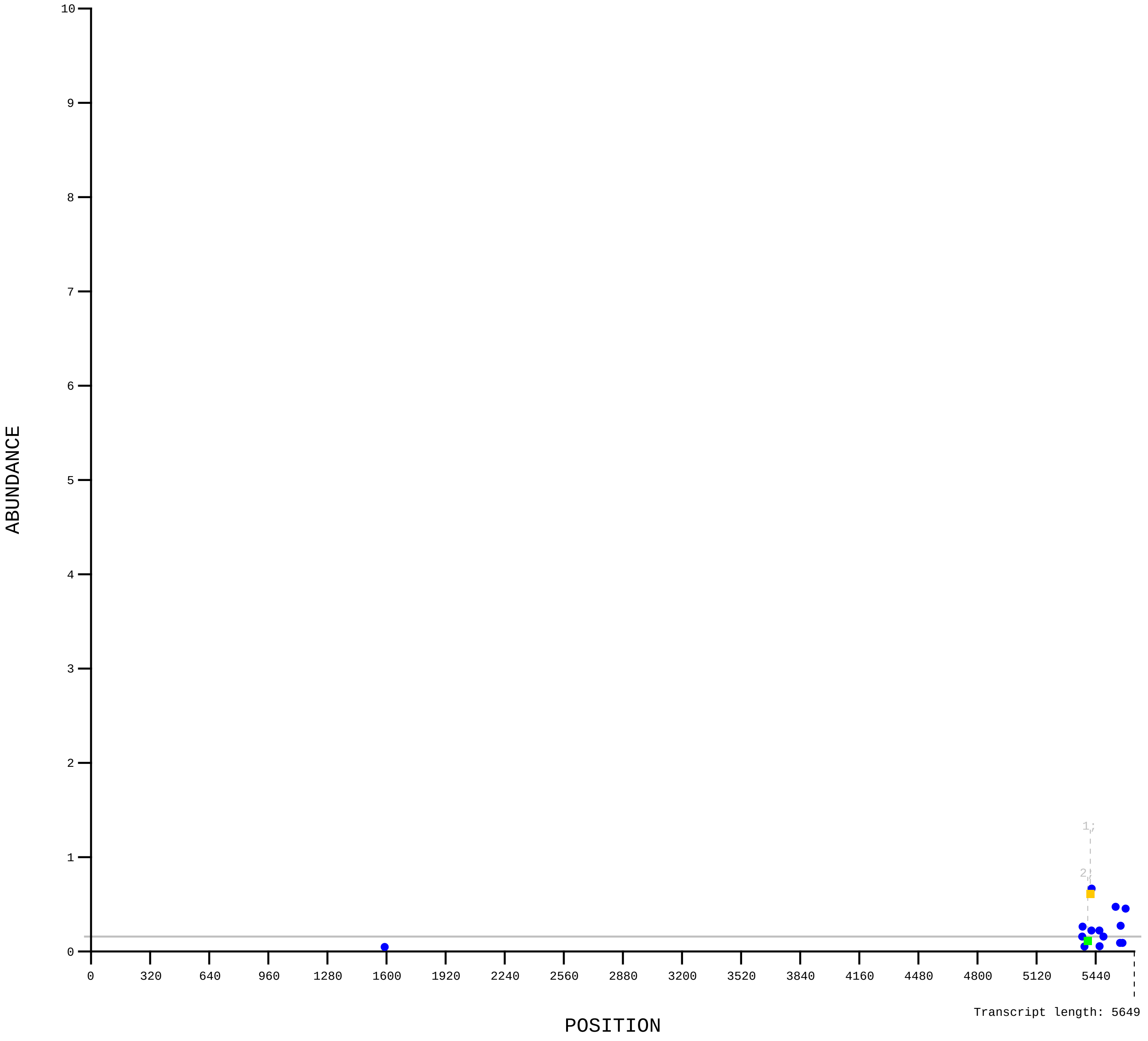

Category: 0 1 2 3 4
Degradome alignment: Median:

```
2  #1  Position:5411  Abundance: 0.61(deg) 10(sRNA)
   5'     TTTCATGATGAGATATCCA          3'  ID:
          || ||o|| ||||o| |||              Score: 4.0
   3' CAAAAATGTGCT-CTCTGTCGGTGCATCGAAT 5'  p-value: 0.01

3  #2  Position:5397  Abundance: 0.11(deg) 5(sRNA)
   5'     AGCCACGTAGCTTAAACCGGTC       3'  ID:
          ||||||||||||||||||||||           Score: 0.0
   3' TCTGTCGGTGCATCGAATTTGGCCAGACTTTC 5'  p-value: 0.0
```

FOXG_03949T0 | Fusarium oxysporum f. sp. lycopersici 4287 hypothetical protein (1074 nt)

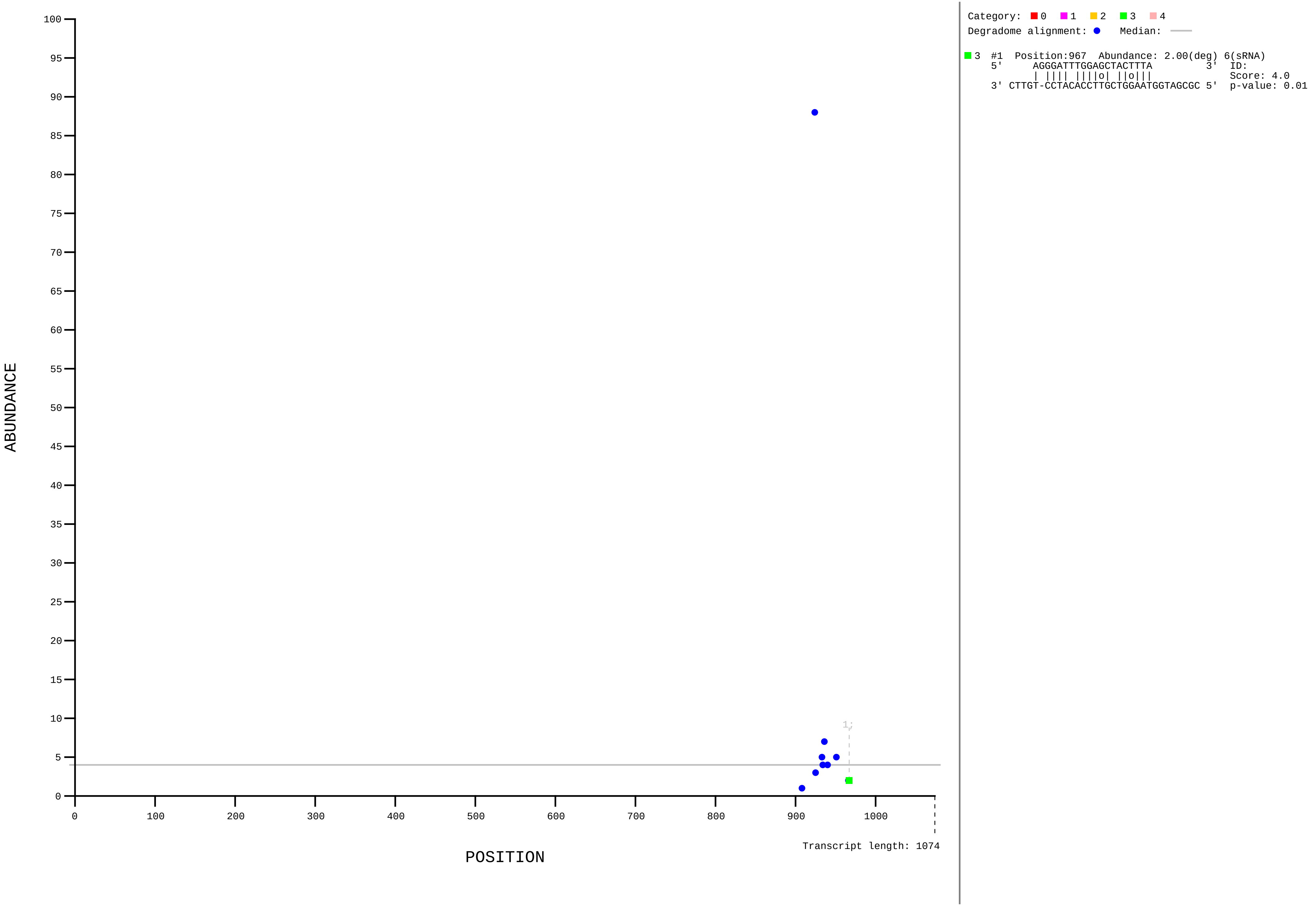

FOXG_08541T0 | Fusarium oxysporum f. sp. lycopersici 4287 hypothetical protein (1524 nt)

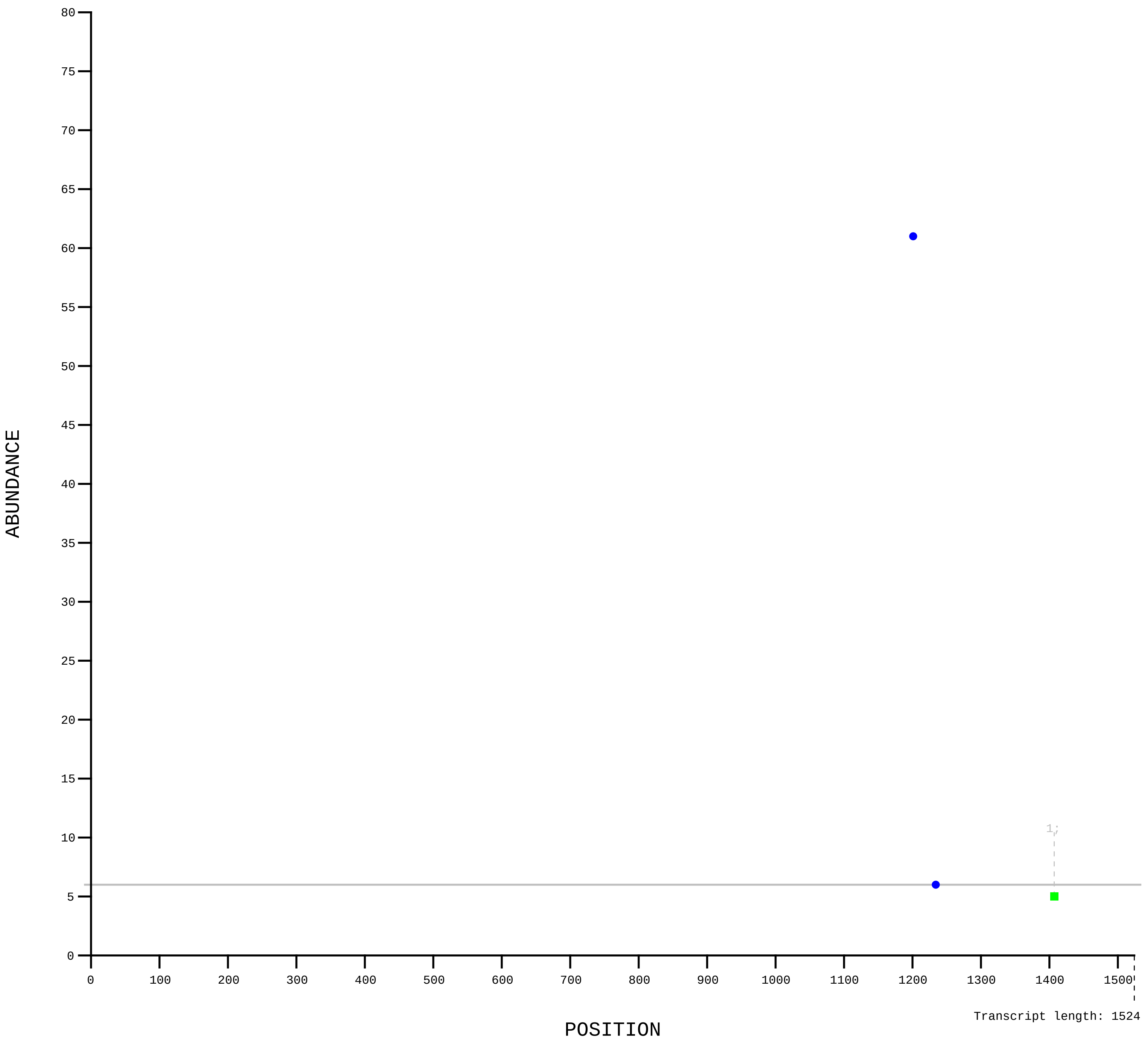

Category: 0 1 2 3 4
Degradome alignment: Median:

3 #1 Position:1407 Abundance: 5.00(deg) 8(sRNA)

```
5'     TCTATTAGTCTCTGCCACAGA        3'  ID:
       || ||o|o||||| | |||||            Score: 4.0
3' GACTAG-TAGTTAGAGAGGTTGTCTAGGATTA 5'  p-value: 0.0
```

FOXG_15666T0 | Fusarium oxysporum f. sp. lycopersici 4287 hypothetical protein (1101 nt) Note(s): (truncated)

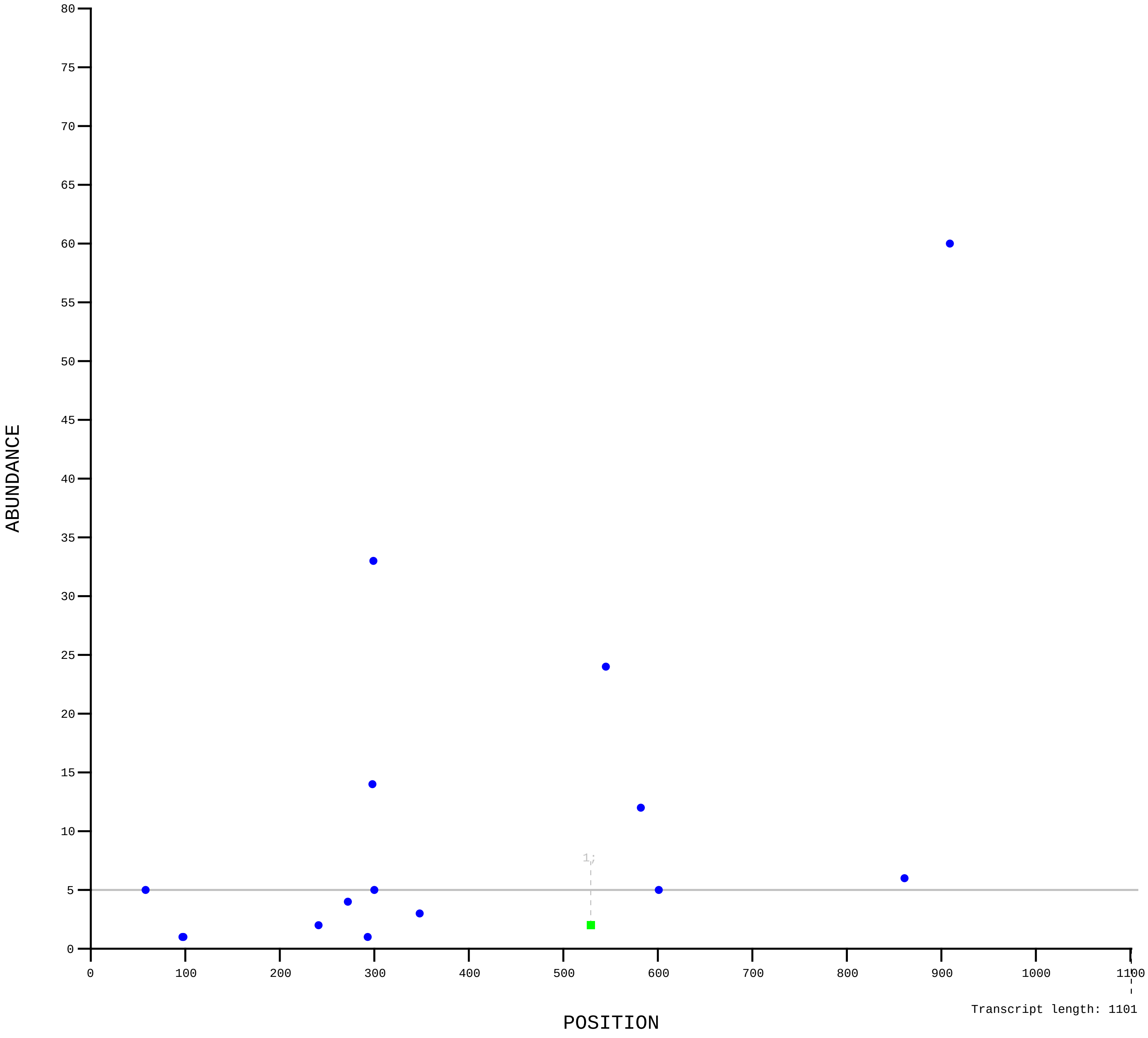

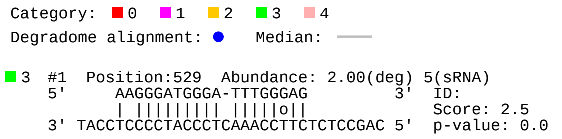

FOXG_03001T0 | Fusarium oxysporum f. sp. lycopersici 4287 hypothetical protein (1260 nt)

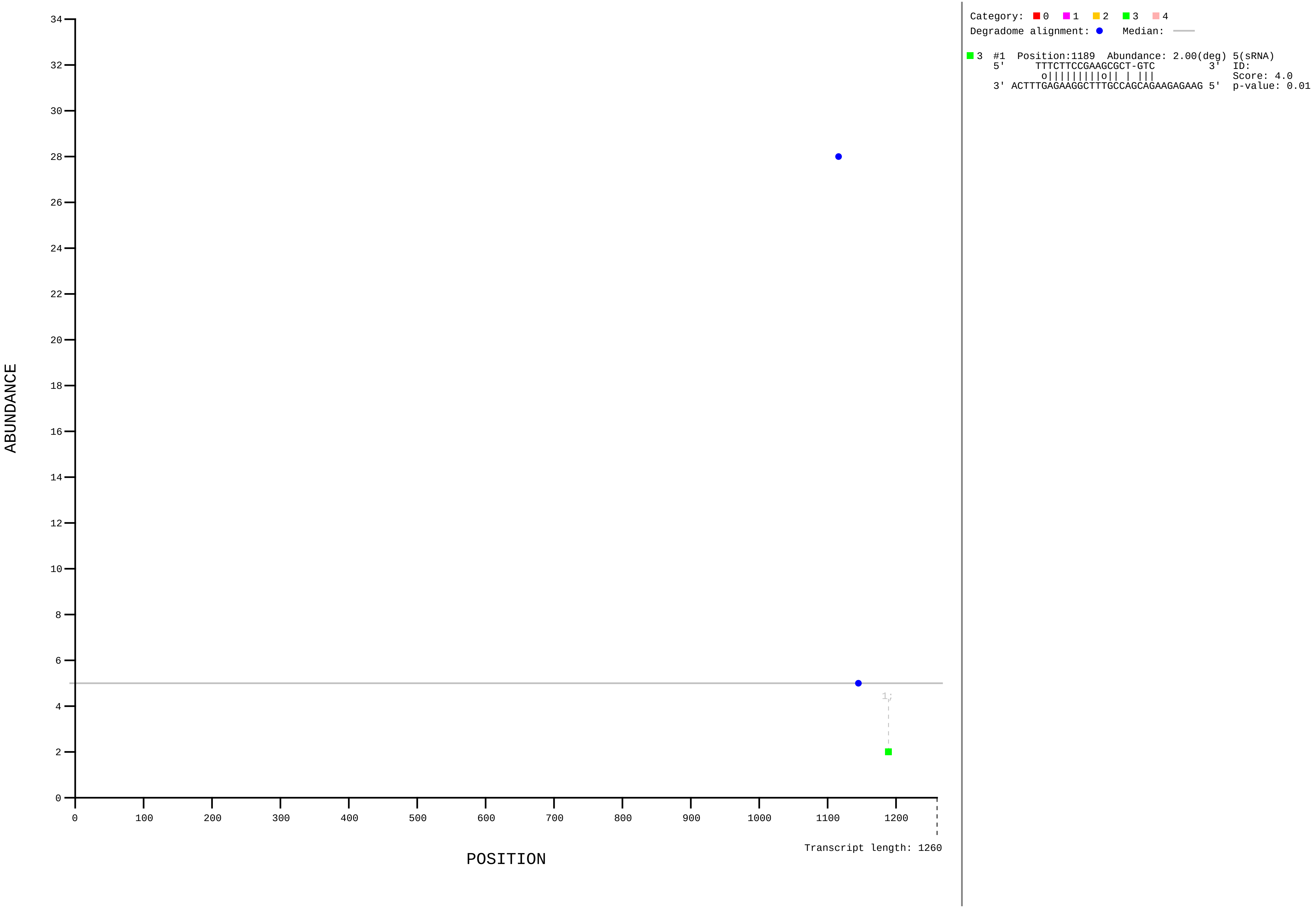

FOXG_02924T0 | Fusarium oxysporum f. sp. lycopersici 4287 acetyl-CoA hydrolase (1581 nt)

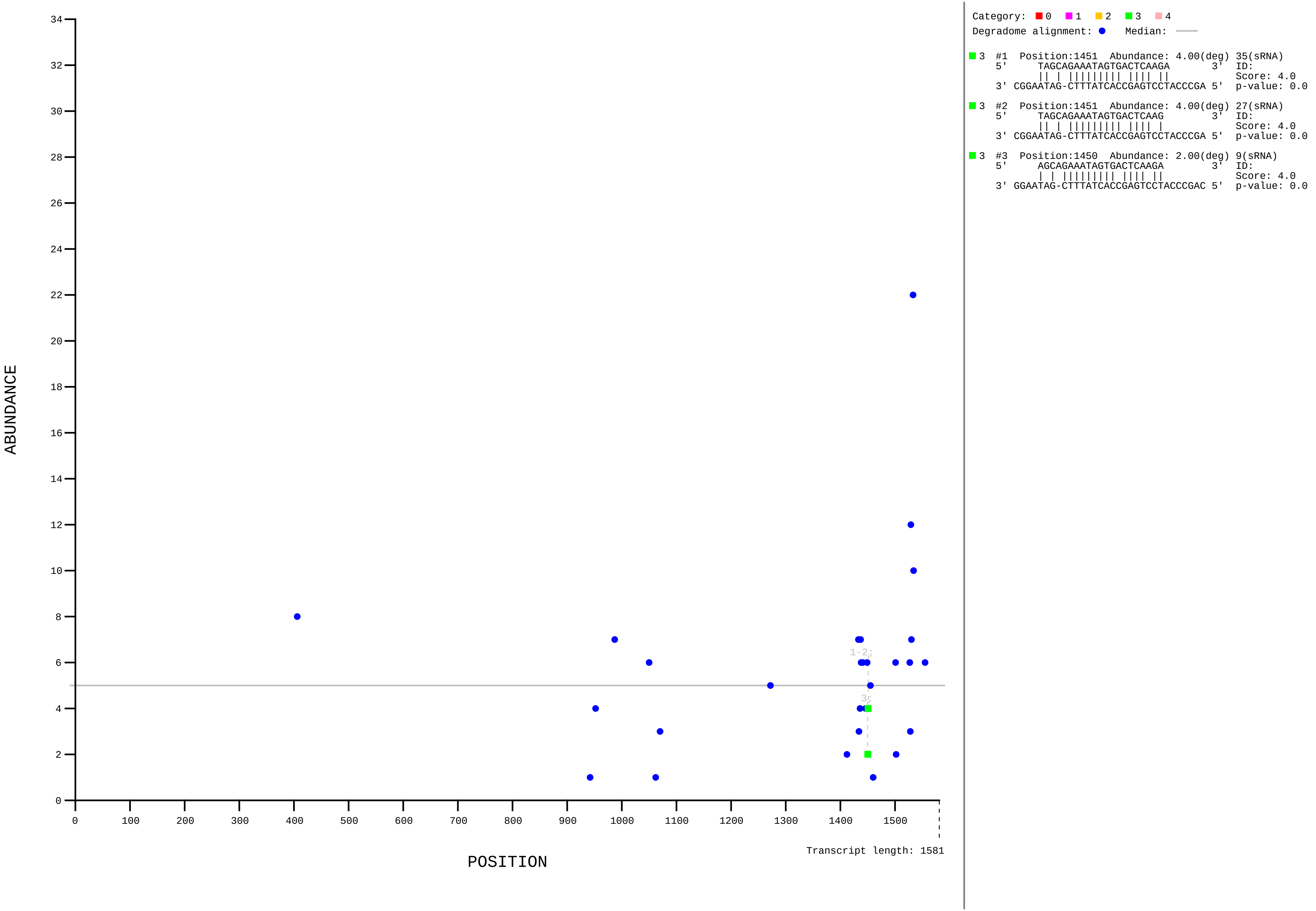

FOXG_07769T0 | Fusarium oxysporum f. sp. lycopersici 4287 hypothetical protein (1566 nt)

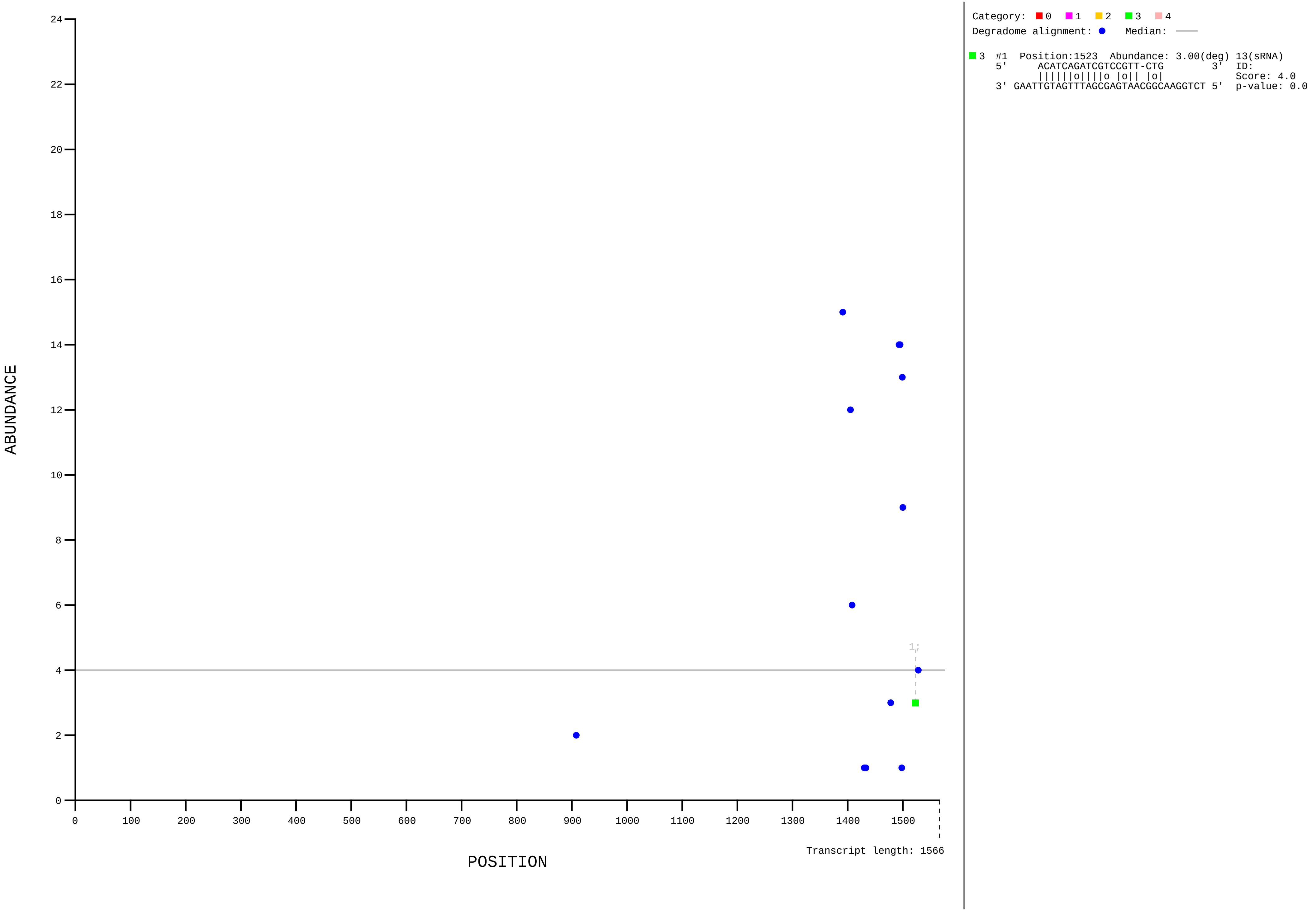

FOXG_01460T0 | Fusarium oxysporum f. sp. lycopersici 4287 AP-3 complex subunit delta (1812 nt) No (truncated)

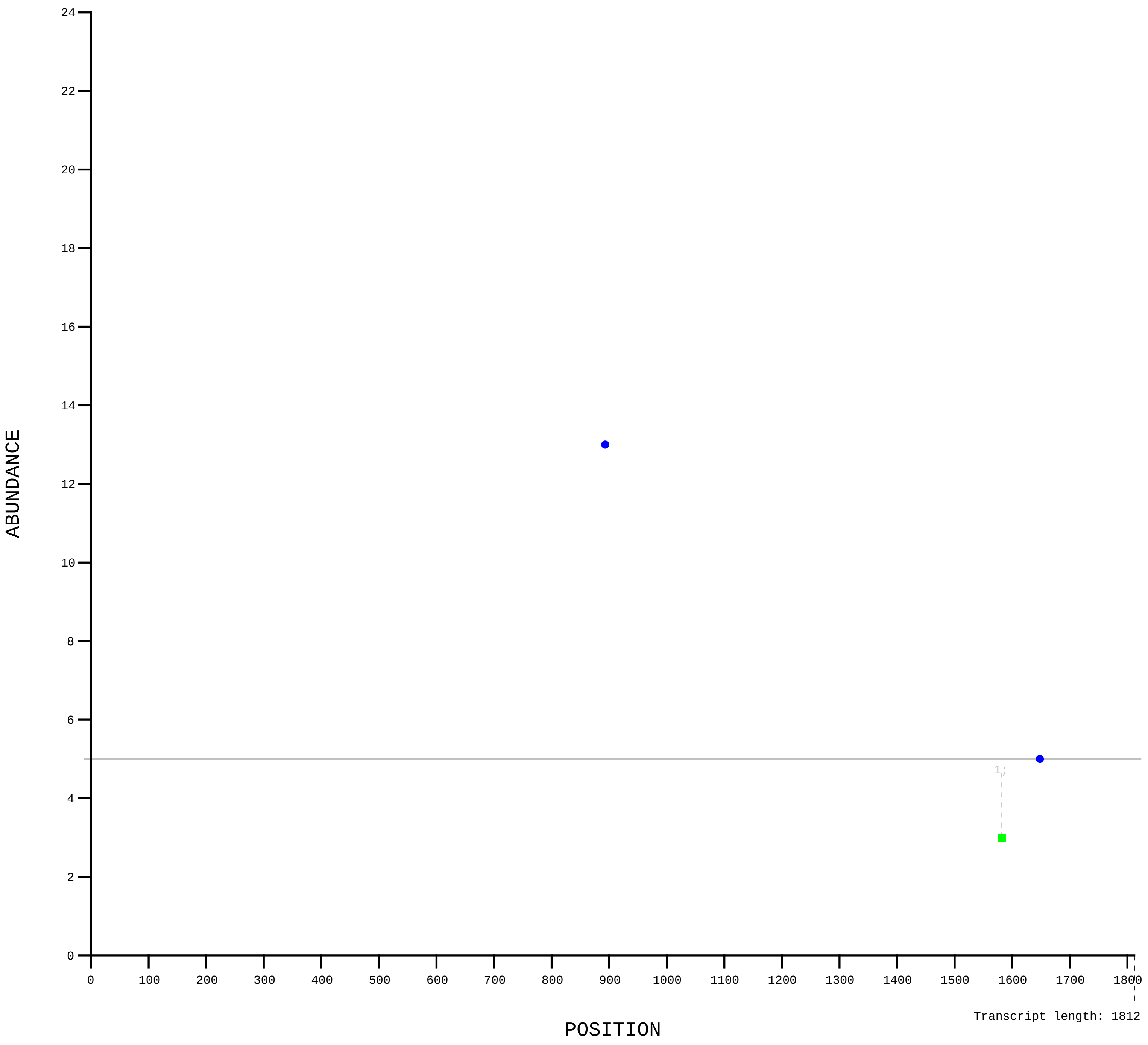

Category: 0 1 2 3 4
Degradome alignment: Median:

```
3  #1  Position:1582  Abundance: 3.00(deg) 29(sRNA)
   5'     TTCCTTCTCTGAAAACTGC          3'  ID:
          || ||||| ||| |||||               Score: 4.0
   3' AGAAAAAGAAGA-ACTGTTGACACGGGAGTTG 5'  p-value: 0.01
```

FOXG_11658T0 | Fusarium oxysporum f. sp. lycopersici 4287 hypothetical protein (900 nt)

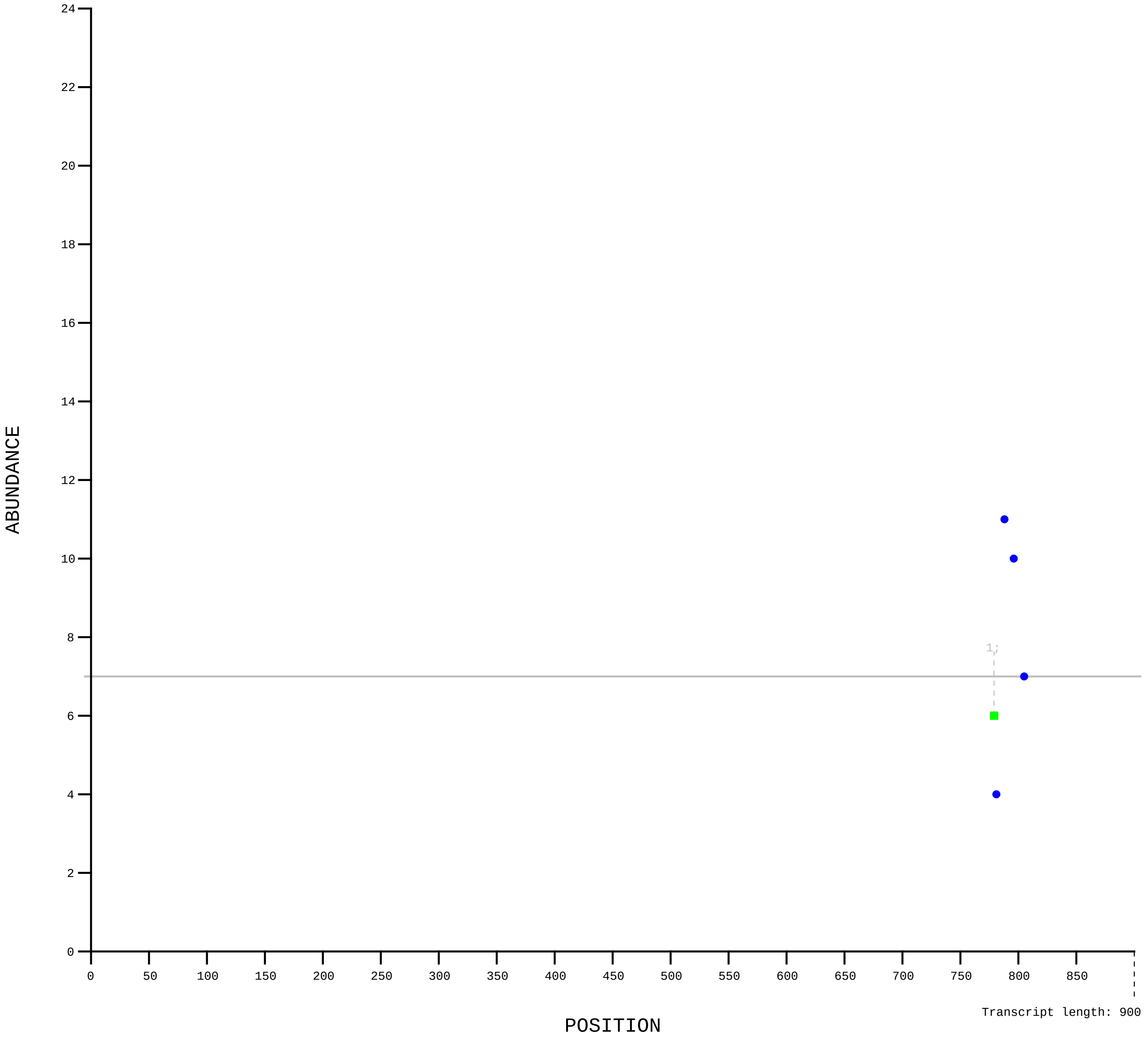

Category: 0 1 2 3 4
Degradome alignment: Median:

3 #1 Position:779 Abundance: 6.00(deg) 50(sRNA)

```
5'      TATTACTGGGGAACTTAGT          3'  ID:
        || |||||o|| ||o| ||              Score: 4.0
3' AGGCATTATGACTCCGTGGA-CAACCTTTGGA 5'  p-value: 0.0
```

FOXG_02286T0 | Fusarium oxysporum f. sp. lycopersici 4287 aspartyl aminopeptidase (1482 nt)

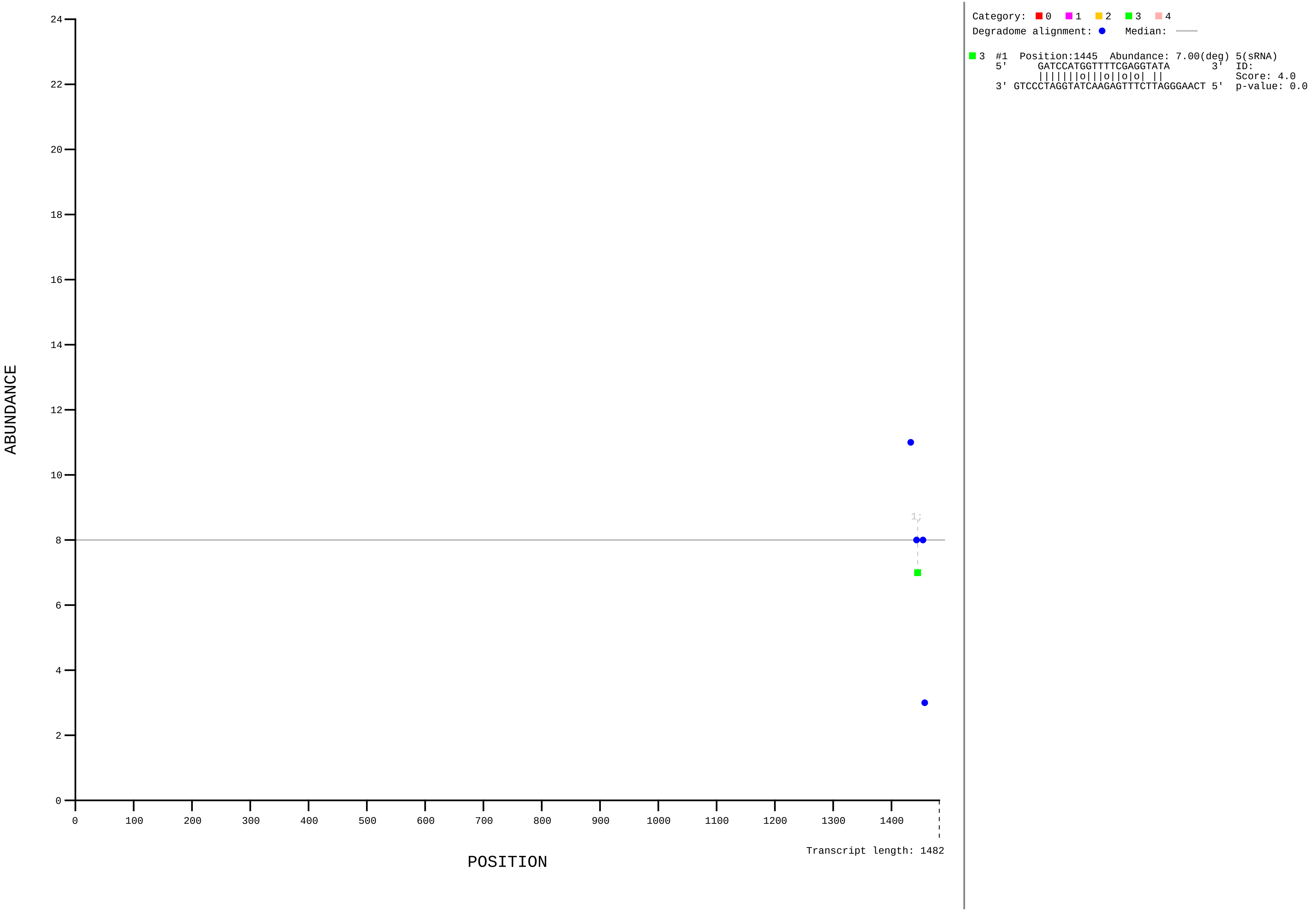

FOXG_13171T0 | Fusarium oxysporum f. sp. lycopersici 4287 hypothetical protein (1101 nt) Note(s): (truncated)

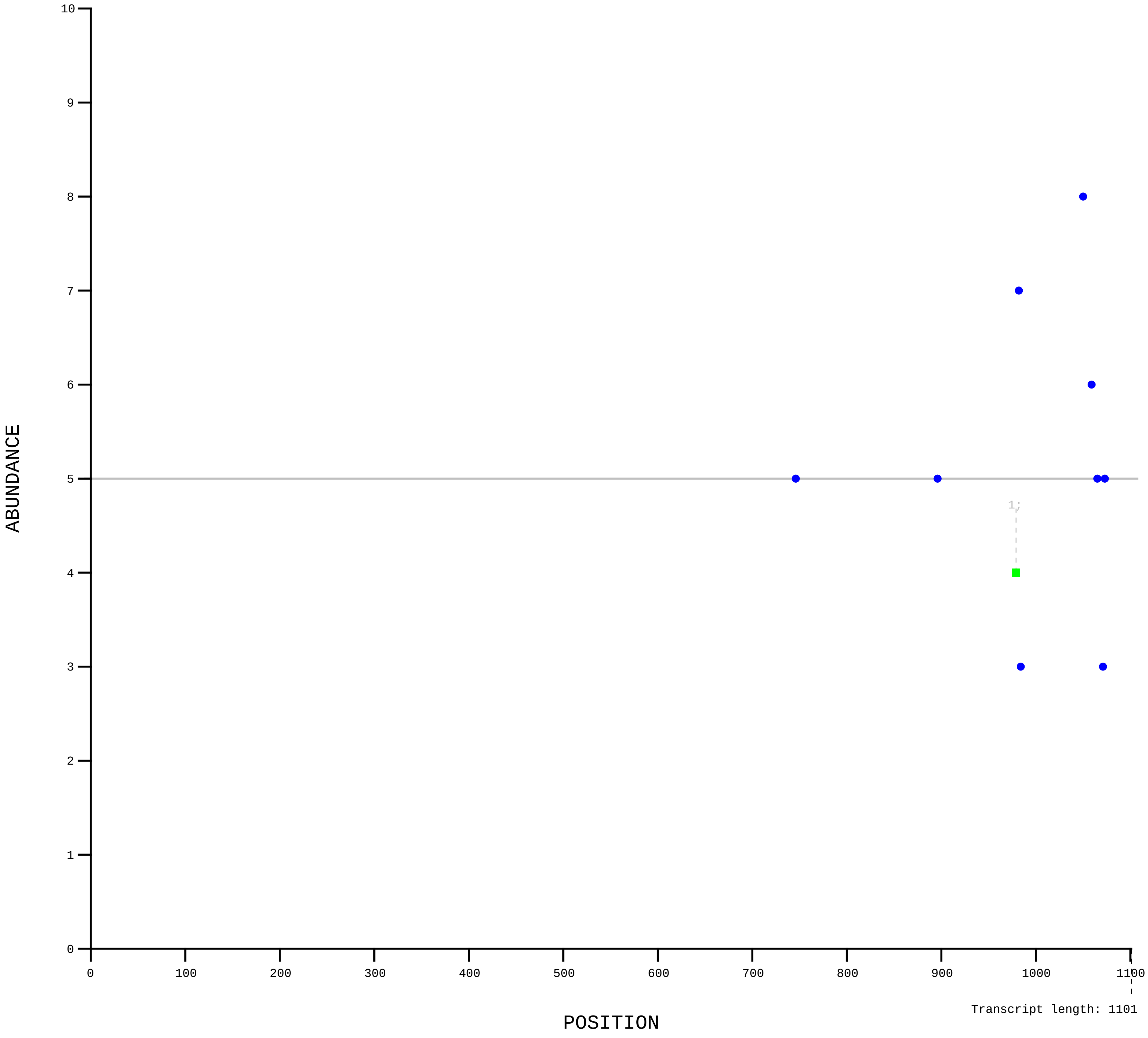

Category: 0 1 2 3 4
Degradome alignment: ● Median: —

```
3  #1  Position:979  Abundance: 4.00(deg) 25(sRNA)
   5'      TTAGAGGAGGATAGCTAGA          3'  ID:
           | |o|||||||||| o||               Score: 4.0
   3' TGGTACTTTCCTCCTATCAGTCGTCATAGCTT 5'  p-value: 0.0
```

FOXG_12791T0 | Fusarium oxysporum f. sp. lycopersici 4287 actin (1320 nt)

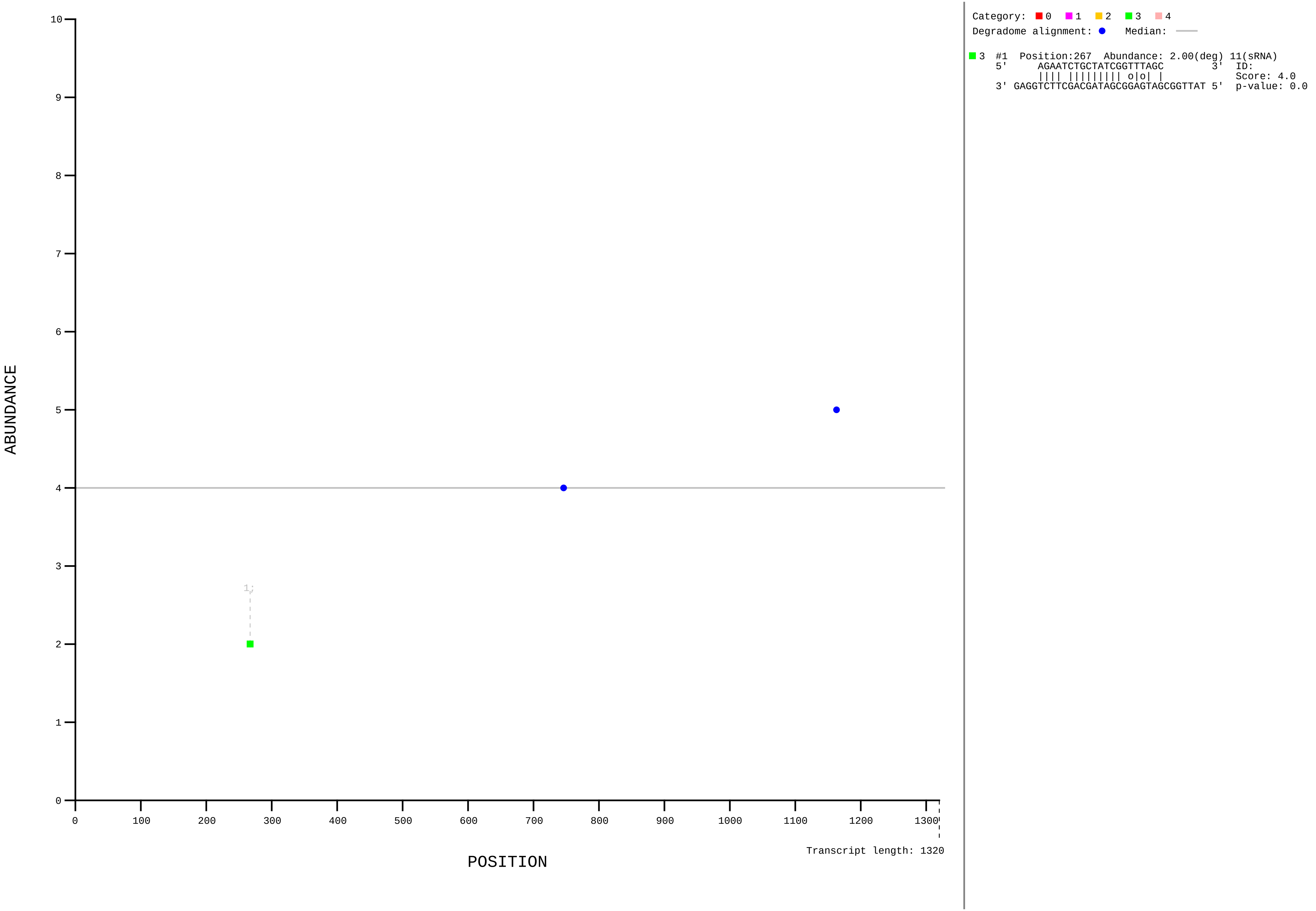

Supplement: Figure S5 — Output of PAREsnip program under stringent parameters. (PDF) [file pone.0104956.s005.pdf]
