# Supplementary material for: Exploring MicroRNA-Like Small RNAs in the Filamentous Fungus Fusarium oxysporum
Source: PLoS One. 2014 Aug 20;9(8):e104956. doi: 10.1371/journal.pone.0104956 (PMC4139310; doi:10.1371/journal.pone.0104956)
Supplement: Table S1 — MicroRNA homologs found in F. oxysporum by BLASTing against miRBase 19.0. (DOCX) [file pone.0104956.s007.docx]

**Table S1**

MicroRNA homologs found in *F. oxysporum* by BLASTing against miRBase 19.0.

| ID in this study | Sequence(5’-3’) | Abundance | Homologous to | miRDeep2 prediction | miRcheck prediction |
| --- | --- | --- | --- | --- | --- |
| fox_20nt_0011877_0000004 | TGGGCTGGGCTGGGCTGGGC | 4 | hsa-miR-3620-5p  gga-miR-1587  hsa-miR-1587  hsa-miR-4507 | Not | Not |
| fox_21nt_0025889_0000002 | TTTTACTGCTACTTATGTTCA | 2 | ath-miR5012 | Not | Not |
| fox_21nt_0091782_0000001 | TGGGCTGGGCTGGGCTGGGCA | 1 | hsa-miR-3620-5p  gga-miR-1587 | Not | Not |
