# Supplementary material for: Exploring MicroRNA-Like Small RNAs in the Filamentous Fungus Fusarium oxysporum
Source: PLoS One. 2014 Aug 20;9(8):e104956. doi: 10.1371/journal.pone.0104956 (PMC4139310; doi:10.1371/journal.pone.0104956)
Supplement: Table S2 — Results of milRNA prediction using miRDeep2 software. (DOCX) [file pone.0104956.s008.docx]

**Table S2**

Results of milRNA prediction using miRDeep2 software.

| No. | ID in this study | Sequence(5’-3’) | Abundance | Length | Precursors | miRDeep2 score | BLAST | significant randfold p-value | [consensus star sequence](file:///H:\references\degradomeFO\manuscript\milRNAout\deep2out\result_05_02_2013_t_20_15_38.html) |
| --- | --- | --- | --- | --- | --- | --- | --- | --- | --- |
| 1 | fox_23nt_0000002_0044137 | TGGATGAATCAAGCGTGGTATGA | 44137 | 23 nt | supercont2.39:99930..100017:- | 35000 | Not | yes | guaucaugcgugauucauccau |
| 2 | fox_21nt_0002335_0000032 | CGGCTGGCATAACATGAAATT | 32 | 21 nt | supercont2.1:966634..966724:- | 91 | 16s rRNA | no | cgucaguuaugccccagauca |
| 3 | fox_25nt_0004838_0000006 | CCCGAGACTGGAGATCAGATTCCCA | 6 | 25 nt | supercont2.6:1310273..1310342:+ | 9.9 | mRNA | yes | ucgagucugucguuucagucuugagucgg |
| 4 | fox_22nt_0013635_0000003 | GGGATGTCAAACCTTCGCGCCA | 3 | 22 nt | supercont2.4:585046..585099:+ | 3.8 | Not | yes | ggccaacgaggguaugacugucccc |
| 5 | fox_21nt_0023199_0000002 | TCAGACTGGACCAGAATTACT | 2 | 21 nt | supercont2.1:3459510..3459552:+ | 3.1 | mRNA | yes | uaauucugguccagucuga |
| 6 | fox_22nt_0008656_0000005 | TCGTCGTTGGGGTTGAGACGGT | 5 | 22 nt | supercont2.8:2301375..2301422:+ | 2.3 | mRNA | yes | cggcucgcccuacgacgaua |
| 7 | fox_18nt_0009482_0000002 | CTCACCGTGCAAAAGGCC | 2 | 18 nt | supercont2.24:824618..824704:+ | 2.2 | Not | yes | gccuuugugcauggauaccagggau |
| 8 | fox_18nt_0009482_0000002 | CTCACCGTGCAAAAGGCC | 2 | 18 nt | supercont2.27:127822..127908:- | 2.2 | Not | yes | gccuuugugcauggauaccagggau |
| 9 | fox_25nt_0001187_0000044 | GGATCGATAGCTCAGTGGTACGAGC | 44 | 25 nt | supercont2.19:614671..614763:- | 1.9 | Not | yes | uuguuuagauuaucagauccuu |
| 10 | fox_20nt_0003578_0000017 | TGGATTGATGACACTTCATC | 17 | 20 nt | supercont2.1:2414907..2414979:+ | 1.8 | mRNA | yes | ugagguagguugauaucauc |
| 11 | fox_20nt_0000156_0000525 | TGCGAGAGGTCCCGGGTTCA | 525 | 20 nt | supercont2.19:1154062..1154101:- | 1.2 | ncRNA | yes | auccccggccagacccuuc |
| 12 | fox_22nt_0021116_0000002 | CTGCTGTTGAAGTTGGCAAGCA | 2 | 22 nt | supercont2.48:105471..105521:- | 1.1 | mRNA | yes | cuugccaauggcagcaguaaag |
| 13 | fox_21nt_0000482_0000178 | TCATTTTTGAGATCTACATTC | 178 | 21 nt | supercont2.11:596839..596910:+ | 0.7 | snoRNA | yes | uuggauccgucguuaaucucgaca |
| 14 | fox_23nt_0008326_0000004 | TGGTGGACGTAGATTTGTATTGG | 4 | 23 nt | supercont2.4:1657832..1657885:+ | 0.4 | Not | no | agguggaaccgugucaaggcua |
| 15 | fox_23nt_0017667_0000002 | AACTGGGAGGAACAGATCAAGCA | 2 | 23 nt | supercont2.10:1148928..1148978: | 0.2 | mRNA | yes | cuugucugcccucucaguacg |
