# Supplementary material for: Exploring MicroRNA-Like Small RNAs in the Filamentous Fungus Fusarium oxysporum
Source: PLoS One. 2014 Aug 20;9(8):e104956. doi: 10.1371/journal.pone.0104956 (PMC4139310; doi:10.1371/journal.pone.0104956)
Supplement: Table S3 — Results of milRNA prediction through miRCheck pipeline. (DOCX) [file pone.0104956.s009.docx]

**Table S3**

Results of milRNA prediction through miRCheck pipeline.

| No. | ID in this study | Sequence(5’-3’) | Abundance | Length (nt) | Precursors |
| --- | --- | --- | --- | --- | --- |
| paired_1_a | fox_18nt_0000017_0003151 | CGGGATTTTCTGCAGGCC | 3151 | 18 | paired_1_a_supercont2.16_-_start_154298_stop_154367_length_70nt |
| paired_1_b | fox_23nt_0000698_0000103 | CCTGTAAAAATTCTTTCTTGGCA | 103 | 23 | paired_1_b_supercont2.18_-_start_747100_stop_747169_length_70nt |
| paired_1_c | fox_23nt_0000698_0000103 | CCTGTAAAAATTCTTTCTTGGCA | 103 | 23 | paired_1_c_supercont2.21_+_start_334973_stop_335042_length_70nt |
| paired_1_d | fox_19nt_0000983_0000060 | AGCGGGATTTTCTGCAGGC | 60 | 19 | paired_1_d_supercont2.24_+_start_719507_stop_719574_length_68nt |
| paired_1_e | fox_18nt_0002491_0000014 | TAGGAGCGGGATTTTCTA | 14 | 18 | paired_1_e_supercont2.2_-_start_4017391_stop_4017447_length_57nt |
| paired_1_f | fox_18nt_0000017_0003151 | CGGGATTTTCTGCAGGCC | 3151 | 18 | paired_1_f_supercont2.37_+_start_28923_stop_28992_length_70nt |
| paired_1_g | fox_18nt_0000017_0003151 | CGGGATTTTCTGCAGGCC | 3151 | 18 | paired_1_g_supercont2.38_+_start_15168_stop_15237_length_70nt |
| paired_2_a | fox_18nt_0000760_0000061 | TTCGCACGCGTAGGTTCG | 61 | 18 | paired_2_a_supercont2.19_+_start_319690_stop_319767_length_78nt |
| paired_2_b | fox_18nt_0001549_0000025 | GGCATTGTGTTCGCACGC | 25 | 18 | paired_2_b_supercont2.19_+_start_1054076_stop_1054135_length_60nt |
| paired_2_c | fox_18nt_0000760_0000061 | TTCGCACGCGTAGGTTCG | 61 | 18 | paired_2_c_supercont2.19_-_start_1206738_stop_1206830_length_93nt |
| paired_2_d | fox_18nt_0000760_0000061 | TTCGCACGCGTAGGTTCG | 61 | 18 | paired_2_d_supercont2.2_-_start_855092_stop_855183_length_92nt |
| paired_2_e | fox_20nt_0000990_0000073 | AAGGCATTGTGTTCGCACGC | 73 | 20 | paired_2_e_supercont2.4_-_start_994286_stop_994341_length_56nt |
| paired_3_a | fox_18nt_0000749_0000062 | CCGGTGTGGTGTATCGGT | 62 | 18 | paired_3_a_supercont2.10_-_start_1979960_stop_1980049_length_90nt |
| paired_3_b | fox_18nt_0000749_0000062 | CCGGTGTGGTGTATCGGT | 62 | 18 | paired_3_b_supercont2.13_+_start_603193_stop_603281_length_89nt |
| paired_4_a | fox_23nt_0000002_0044137 | TGGATGAATCAAGCGTGGTATGA | 44137 | 23 | paired_4_a_supercont2.39_-_start_99911_stop_100039_length_129nt |
| paired_5_a | fox_19nt_0000134_0000538 | AATCCCCGCTGTTGTATGT | 538 | 19 | paired_5_a_supercont2.12_+_start_302082_stop_302155_length_74nt |
| paired_6_a | fox_20nt_0000651_0000116 | TGTGTTGAGAAGCTAAGTTA | 116 | 20 | paired_6_a_supercont2.2_-_start_3109721_stop_3109894_length_174nt |
| paired_7_a | fox_19nt_0001135_0000051 | TCTGAAAGTTACGAGTTCG | 51 | 19 | paired_7_a_supercont2.15_-_start_647738_stop_647795_length_58nt |
| paired_8_a | fox_18nt_0002010_0000018 | TTTCGTGATGAGTTGTTT | 18 | 18 | paired_8_a_supercont2.1_+_start_1981477_stop_1981568_length_92nt |
| paired_9_a | fox_22nt_0010774_0000004 | TTGCACACTCGTTCGAGCTGCG | 4 | 22 | paired_9_a_supercont2.17_+_start_704955_stop_705057_length_103nt |
| single_1_a | fox_21nt_0003862_0000018 | TGGAAGAGATGGACGAAACGA | 18 | 21 | single_1_a_supercont2.112_-_start_3929_stop_4003_length_75nt |
| single_1_b | fox_21nt_0003862_0000018 | TGGAAGAGATGGACGAAACGA | 18 | 21 | single_1_b_supercont2.25_-_start_822740_stop_822814_length_75nt |
| single_1_c | fox_21nt_0003862_0000018 | TGGAAGAGATGGACGAAACGA | 18 | 21 | single_1_c_supercont2.28_-_start_673717_stop_673791_length_75nt |
| single_1_d | fox_21nt_0003862_0000018 | TGGAAGAGATGGACGAAACGA | 18 | 21 | single_1_d_supercont2.36_-_start_451759_stop_451833_length_75nt |
| single_1_e | fox_21nt_0003862_0000018 | TGGAAGAGATGGACGAAACGA | 18 | 21 | single_1_e_supercont2.36_-_start_480413_stop_480487_length_75nt |
| single_1_f | fox_21nt_0003862_0000018 | TGGAAGAGATGGACGAAACGA | 18 | 21 | single_1_f_supercont2.37_+_start_9334_stop_9408_length_75nt |
| single_1_g | fox_21nt_0003862_0000018 | TGGAAGAGATGGACGAAACGA | 18 | 21 | single_1_g_supercont2.44_+_start_35475_stop_35549_length_75nt |
| single_1_h | fox_21nt_0003862_0000018 | TGGAAGAGATGGACGAAACGA | 18 | 21 | single_1_h_supercont2.6_+_start_4407_stop_4481_length_75nt |
| single_1_i | fox_21nt_0003862_0000018 | TGGAAGAGATGGACGAAACGA | 18 | 21 | single_1_i_supercont2.93_-_start_7756_stop_7830_length_75nt |
| single_2_a | fox_21nt_0001822_0000041 | TAAGACTTCTGAGGAGGTGGC | 41 | 21 | single_2_a_supercont2.25_-_start_824464_stop_824534_length_71nt |
| single_2_b | fox_21nt_0001822_0000041 | TAAGACTTCTGAGGAGGTGGC | 41 | 21 | single_2_b_supercont2.28_-_start_675434_stop_675504_length_71nt |
| single_2_c | fox_21nt_0001822_0000041 | TAAGACTTCTGAGGAGGTGGC | 41 | 21 | single_2_c_supercont2.36_-_start_453476_stop_453546_length_71nt |
| single_2_d | fox_21nt_0001822_0000041 | TAAGACTTCTGAGGAGGTGGC | 41 | 21 | single_2_d_supercont2.37_+_start_7622_stop_7690_length_69nt |
| single_2_e | fox_21nt_0001822_0000041 | TAAGACTTCTGAGGAGGTGGC | 41 | 21 | single_2_e_supercont2.44_+_start_10076_stop_10144_length_69nt |
| single_2_f | fox_21nt_0001822_0000041 | TAAGACTTCTGAGGAGGTGGC | 41 | 21 | single_2_f_supercont2.44_+_start_33760_stop_33834_length_75nt |
| single_2_g | fox_21nt_0001822_0000041 | TAAGACTTCTGAGGAGGTGGC | 41 | 21 | single_2_g_supercont2.6_+_start_2695_stop_2763_length_69nt |
| single_3_a | fox_26nt_0002525_0000015 | TGCTAGGGTAGAGAATTTTTGCAGGC | 15 | 26 | single_3_a_supercont2.18_-_start_835943_stop_836012_length_70nt |
| single_3_b | fox_26nt_0002525_0000015 | TGCTAGGGTAGAGAATTTTTGCAGGC | 15 | 26 | single_3_b_supercont2.18_-_start_1095763_stop_1095832_length_70nt |
| single_3_c | fox_26nt_0002525_0000015 | TGCTAGGGTAGAGAATTTTTGCAGGC | 15 | 26 | single_3_c_supercont2.19_+_start_778436_stop_778505_length_70nt |
| single_3_d | fox_26nt_0002525_0000015 | TGCTAGGGTAGAGAATTTTTGCAGGC | 15 | 26 | single_3_d_supercont2.21_+_start_247051_stop_247120_length_70nt |
| single_3_e | fox_26nt_0002525_0000015 | TGCTAGGGTAGAGAATTTTTGCAGGC | 15 | 26 | single_3_e_supercont2.24_+_start_290342_stop_290411_length_70nt |
| single_3_f | fox_26nt_0002525_0000015 | TGCTAGGGTAGAGAATTTTTGCAGGC | 15 | 26 | single_3_f_supercont2.41_+_start_248664_stop_248733_length_70nt |
| single_3_g | fox_26nt_0002525_0000015 | TGCTAGGGTAGAGAATTTTTGCAGGC | 15 | 26 | single_3_g_supercont2.43_+_start_171922_stop_171991_length_70nt |
| single_4_a | fox_19nt_0000004_0016753 | TCCGGTATGGTGTAGTGGC | 16753 | 19 | single_4_a_supercont2.3_-_start_3100467_stop_3100674_length_208nt |
| single_5_a | fox_23nt_0000219_0000403 | TCCGTAGTATAGTGGTCAGTATG | 403 | 23 | single_5_a_supercont2.14_-_start_769284_stop_769380_length_97nt |
| single_6_a | fox_21nt_0000621_0000137 | AGGCGTCTGTTCGAATCAGGC | 137 | 21 | single_6_a_supercont2.1_-_start_2159158_stop_2159298_length_141nt |
| single_7_a | fox_21nt_0001030_0000077 | TGAGACCCGGGTTCAATTCCC | 77 | 21 | single_7_a_supercont2.19_+_start_73416_stop_73517_length_102nt |
| single_8_a | fox_18nt_0000715_0000066 | TGGTTAGGACGTTGGATT | 66 | 18 | single_8_a_supercont2.15_-_start_889475_stop_889547_length_73nt |
| single_9_a | fox_19nt_0001126_0000052 | GCTTCCGTAGCTCAGTTGG | 52 | 19 | single_9_a_supercont2.6_-_start_1979709_stop_1979861_length_153nt |
| single_10_a | fox_24nt_0001324_0000041 | GGATCGATAGCTCAGCGGTAGAGC | 41 | 24 | single_10_a_supercont2.15_+_start_613829_stop_613913_length_85nt |
| single_11_a | fox_21nt_0002639_0000028 | GGTTCCGTGGTCTAGTTGGTT | 28 | 21 | single_11_a_supercont2.5_+_start_2446474_stop_2446531_length_58nt |
| single_12_a | fox_22nt_0003930_0000015 | AGAGTCGATGACTGGTTGTGGA | 15 | 22 | single_12_a_supercont2.15_-_start_22334_stop_22516_length_183nt |
| single_13_a | fox_20nt_0004882_0000012 | GCGTAATTGGTTTAGTGGTA | 12 | 20 | single_13_a_supercont2.5_+_start_1142170_stop_1142227_length_58nt |
| single_14_a | fox_18nt_0003512_0000009 | CTGCGGATTCTGATTCCG | 9 | 18 | single_14_a_supercont2.15_-_start_876025_stop_876134_length_110nt |
| single_15_a | fox_18nt_0004002_0000007 | ATTACTGGAGACGATGAG | 7 | 18 | single_15_a_supercont2.10_-_start_61798_stop_62019_length_222nt |
| single_16_a | fox_20nt_0008317_0000006 | TTTGCAGACGACTTAGCTGA | 6 | 20 | single_16_a_supercont2.10_-_start_2204577_stop_2204692_length_116nt |
| single_17_a | fox_22nt_0009745_0000005 | CAGAATACTGACACTCCTGCAG | 5 | 22 | single_17_a_supercont2.1_-_start_3532633_stop_3532689_length_57nt |
| single_18_a | fox_23nt_0007016_0000005 | TGTCCAAGCGTTGGGACTACGGT | 5 | 23 | single_18_a_supercont2.14_-_start_1383104_stop_1383174_length_71nt |
| single_19_a | fox_20nt_0013911_0000003 | TGGACATCTTGGAAGACACC | 3 | 20 | single_19_a_supercont2.8_+_start_277370_stop_277456_length_87nt |
| single_20_a | fox_23nt_0011634_0000003 | GATTGTAGTAGAATCTAGCGGCA | 3 | 23 | single_20_a_supercont2.5_+_start_1105144_stop_1105212_length_69nt |
| single_21_a | fox_21nt_0016987_0000003 | TGAGAACTGAATACCAAGCCT | 3 | 21 | single_21_a_supercont2.4_-_start_1656933_stop_1657047_length_115nt |
| single_22_a | fox_22nt_0015592_0000003 | GGAGAACTTTATGCCAAAGACT | 3 | 22 | single_22_a_supercont2.3_+_start_1613066_stop_1613130_length_65nt |
| single_23_a | fox_28nt_0007465_0000003 | TGGACTACTCACTACTAGTTGGCATACA | 3 | 28 | single_23_a_supercont2.17_+_start_271398_stop_271502_length_105nt |
| single_24_a | fox_20nt_0018250_0000003 | GAAGACTAGGAATGATGAAC | 3 | 20 | single_24_a_supercont2.15_-_start_1074968_stop_1075055_length_88nt |
| single_25_a | fox_21nt_0018041_0000003 | GTAGATACTTTTTGCGATGGT | 3 | 21 | single_25_a_supercont2.15_-_start_1066113_stop_1066319_length_207nt |
| single_26_a | fox_23nt_0012620_0000003 | GCTGTGACGGAAGAGTGTAGGGT | 3 | 23 | single_26_a_supercont2.13_-_start_782949_stop_783025_length_77nt |
