# Supplementary material for: Exploring MicroRNA-Like Small RNAs in the Filamentous Fungus Fusarium oxysporum
Source: PLoS One. 2014 Aug 20;9(8):e104956. doi: 10.1371/journal.pone.0104956 (PMC4139310; doi:10.1371/journal.pone.0104956)
Supplement: Table S4 — Target prediction of fox-milRNAs using psRNATarget online. (DOCX) [file pone.0104956.s010.docx]

**Table S4**

Target prediction of fox-milRNAs using psRNATarget online.

| milRNAs | Targets | Exp | UPE | miRNA  start..end | Target  start..end | miRNA_aligned_fragment | Target_aligned_fragment | Inhibition^a^ | Target_Desc. |
| --- | --- | --- | --- | --- | --- | --- | --- | --- | --- |
| fox_milR_1a,b,c,d,e,f,g | FOXG_04240T0 | 3 | 20.3 | 1..22 | 493..514 | UGCUAGGGUAGAGAAUUUUUGC | GUCAAAGUUCUCUACGCUAGCA | Cleavage | hypothetical protein (825 nt) |
| fox_milR_2a,b | FOXG_03470T0 | 3 | 23.8 | 1..23 | 230..252 | GACAACGUGGCCGAGUGGUUAAG | CUGAAGCUCUCGGCGACGUUGUC | Translation | glycine cleavage system H protein (519 nt) |
| fox_milR_3b | FOXG_04910T0 | 3 | 23.2 | 1..27 | 2084..2110 | CCGGUGUGGUGUAUCGGUUAUCAUUCC | GUAACGGUAACCGCUACACCGCACCCG | Cleavage | hypothetical protein (2952 nt) |
| fox_milR_4 | FOXG_09739T0 | 1.5 | 22.4 | 1..20 | 135..154 | UGGAUGAAUCAAGCGUGGUA | UAUCACGCUUGAUUCGUUCA | Cleavage | quinate permease (1617 nt) |
| fox_milR_4 | FOXG_08812T0 | 2.5 | 16.7 | 1..21 | 218..238 | UGGAUGAAUCAAGCGUGGUAU | AUACCACGCUUAAAUUAUCCA | Translation | hypothetical protein (378 nt) |
| fox_milR_4 | FOXG_08797T0 | 2.5 | 7.4 | 1..20 | 68..87 | UGGAUGAAUCAAGCGUGGUA | UACCACGCUUCAAUCAUCUA | Translation | hypothetical protein (825 nt) |
| fox_milR_6 | FOXG_00067T0 | 3 | 18.5 | 1..24 | 3202..3225 | GUUCCGUGGUCUAGUUGGUUAUGG | CCAGAACCAGCUAGUCCACGUAAC | Translation | hypothetical protein (3945 nt) |
| fox_milR_7 | FOXG_16460T0 | 3 | 20.0 | 1..20 | 1100..1119 | CUUCCGUAGUAUAGUGGUCA | UGCUCACUAUAUUGCGGAGG | Cleavage | hypothetical protein (1212 nt) |
| fox_milR_8 | FOXG_05730T0 | 2 | 18.2 | 1..20 | 170..189 | CUUGAGACCCGGGUUCAAUU | CGUUGAAUCCGGGUCUCAAG | Cleavage | hypothetical protein (867 nt) |
| fox_milR_8 | FOXG_15485T0  FOXG_15486T0 | 2.5 | 17.4 | 1..20 | 1433..1452 | CUUGAGACCCGGGUUCAAUU | AAUGGAACUGGGGUCUCAAG | Translation | hypothetical protein (1644 nt) |
