# Supplementary material for: Exploring MicroRNA-Like Small RNAs in the Filamentous Fungus Fusarium oxysporum
Source: PLoS One. 2014 Aug 20;9(8):e104956. doi: 10.1371/journal.pone.0104956 (PMC4139310; doi:10.1371/journal.pone.0104956)
Supplement: Table S5 — Conservation analysis of fox-milRNA precursors in closely related Fusarium species. The precursor sequences of milRNAs were BLAST against related genomes. Alignment length more than 95% was calculated. (DOCX) [file pone.0104956.s011.docx]

**Table S5**

Conservation analysis of fox-milRNA precursors in closely related *Fusarium* species.

| **Fusarium species^a^** | **Family (member)** | | | | | | | |
| --- | --- | --- | --- | --- | --- | --- | --- | --- |
|  | milRNA_1 (7) | milRNA_2 (5) | milRNA_3 (2) | milRNA_4 (1) | milRNA_5 (1) | milRNA_6 (1) | milRNA_7 (1) | milRNA_8 (1) |
| *Fusarium graminearum* | 0 | 8 | 0 | 0 | 0 | 0 | 0 | 0 |
| *Fusarium verticillioides* | 0 | 7 | 3 | 0 | 0 | 0 | 1 | 0 |
| *Fusarium oxysporum cl57* | 12 | 7 | 3 | 1 | 1 | 1 | 1 | 1 |
| *Fusarium oxysporum cotton* | 25 | 7 | 3 | 1 | 1 | 1 | 1 | 1 |
| *Fusarium oxysporum f. sp. melonis 26406* | 22 | 7 | 3 | 1 | 1 | 1 | 1 | 1 |
| *Fusarium oxysporum fo47* | 12 | 7 | 3 | 1 | 1 | 1 | 1 | 1 |
| *Fusarium oxysporum fo5176* | 19 | 7 | 3 | 1 | 1 | 1 | 1 | 0 |
| *Fusarium oxysporum hdv247* | 39 | 7 | 3 | 1 | 1 | 1 | 1 | 1 |
| *Fusarium oxysporum ii5* | 9 | 7 | 3 | 1 | 1 | 1 | 1 | 1 |
| *Fusarium oxysporum mn25* | 8 | 7 | 3 | 1 | 1 | 1 | 1 | 1 |
| *Fusarium oxysporum nrrl32931* | 11 | 7 | 3 | 1 | 1 | 1 | 1 | 1 |
| *Fusarium oxysporum phw808* | 27 | 9 | 3 | 1 | 1 | 1 | 1 | 1 |
| *Fusarium oxysporum phw815* | 42 | 7 | 3 | 0 | 1 | 1 | 1 | 1 |

^a^Supercontigs were download from <http://www.broadinstitute.org/> (Fusarium Comparative Sequencing Project, Broad Institute of Harvard and MIT).
