# Supplementary material for: Exploring MicroRNA-Like Small RNAs in the Filamentous Fungus Fusarium oxysporum
Source: PLoS One. 2014 Aug 20;9(8):e104956. doi: 10.1371/journal.pone.0104956 (PMC4139310; doi:10.1371/journal.pone.0104956)
Supplement: Table S6 — Primers used for RT-PCR experiments in this study. (DOCX) [file pone.0104956.s012.docx]

**Table S6**

Primers used for RT-PCR experiments in this study.

| ID | Primer sequence(5’-3’) | Length (nt) |
| --- | --- | --- |
| Fox_miRNA_1-a,b,c,d,e,f,g | GCTAGGGTAGAGAATTTTTGCAG | 23 |
| Fox_miRNA_2-a,b | CAACGTGGCCGAGTGGTTAAG | 21 |
| Fox_miRNA_2-c,d,e | CATTGTGTTCGCACGCGTAGG | 21 |
| Fox_miRNA_3-a | GTGTGGTGTATCGGTTTATCATTC | 24 |
| Fox_miRNA_3-b | GTGTGGTGTATCGGTTATCATTC | 23 |
| Fox_miRNA_4 | TGGATGAATCAAGCGTGGTATG | 22 |
| Fox_miRNA_5 | TCCGGTATGGTGTAGTGGC | 19 |
| Fox_miRNA_6 | GTTCCGTGGTCTAGTTGGTTATG | 23 |
| Fox_miRNA_7 | CCGTAGTATAGTGGTCAGTATGC | 23 |
| Fox_miRNA_8 | CTTGAGACCCGGGTTCAATTCC | 22 |
| Fox_sR_1a,b | CATCACCACCCTATGACGGA | 20 |
| Fox_sR_2a,b,c | CAGGACTGAATGCTTTATC | 19 |
| Fox_sR_3 | CGCAGAAGGTCCCGAGTTC | 19 |
| Reverse primer | GCTGTCAACGATACGCTACGTAACGGCATGACAGTGTTTTTTTTTTTTTTTTTTTTTTTTVN | 62 |
| Downstream universal primer | GCTGTCAACGATACGCTACGTAACG | 25 |
